# Histological and molecular glioblastoma, IDH-wildtype: a real-world landscape using the 2021 WHO classification of central nervous system tumors

**DOI:** 10.3389/fonc.2023.1200815

**Published:** 2023-07-06

**Authors:** Xiaopeng Guo, Lingui Gu, Yilin Li, Zhiyao Zheng, Wenlin Chen, Yaning Wang, Yuekun Wang, Hao Xing, Yixin Shi, Delin Liu, Tianrui Yang, Yu Xia, Junlin Li, Jiaming Wu, Kun Zhang, Tingyu Liang, Hai Wang, Qianshu Liu, Shanmu Jin, Tian Qu, Siying Guo, Huanzhang Li, Yu Wang, Wenbin Ma

**Affiliations:** ^1^ Department of Neurosurgery, Center for Malignant Brain Tumors, National Glioma MDT Alliance, Peking Union Medical College Hospital, Chinese Academy of Medical Sciences and Peking Union Medical College, Beijing, China; ^2^ China Anti-Cancer Association Specialty Committee of Glioma, Peking Union Medical College Hospital, Beijing, China; ^3^ ’4 + 4’ Medical Doctor Program, Chinese Academy of Medical Sciences and Peking Union Medical College, Beijing, China; ^4^ Department of Neurosurgery, Beijing Tiantan Hospital, Capital Medical University, Beijing, China; ^5^ Research Unit of Accurate Diagnosis, Treatment, and Translational Medicine of Brain Tumors (No.2019RU011), Chinese Academy of Medical Sciences, Beijing, China; ^6^ Eight-year Medical Doctor Program, Chinese Academy of Medical Sciences and Peking Union Medical College, Beijing, China

**Keywords:** glioblastoma, histological glioblastoma, molecular glioblastoma, molecular alteration, chromosome copy number variation

## Abstract

**Introduction:**

Glioblastoma (GBM), the most lethal primary brain malignancy, is divided into histological (hist-GBM) and molecular (mol-GBM) subtypes according to the 2021 World Health Organization classification of central nervous system tumors. This study aimed to characterize the clinical, radiological, molecular, and survival features of GBM under the current classification scheme and explore survival determinants.

**Methods:**

We re-examined the genetic alterations of IDH-wildtype diffuse gliomas at our institute from 2011 to 2022, and enrolled GBMs for analysis after re-classification. Univariable and multivariable analyses were used to identify survival determinants.

**Results:**

Among 209 IDH-wildtype gliomas, 191 were GBMs, including 146 hist-GBMs (76%) and 45 mol-GBMs (24%). Patients with mol-GBMs were younger, less likely to develop preoperative motor dysfunction, and more likely to develop epilepsy than hist-GBMs. Mol-GBMs exhibited lower radiographic incidences of contrast enhancement and intratumoral necrosis. Common molecular features included copy-number changes in chromosomes 1, 7, 9, 10, and 19, as well as alterations in EGFR, TERT, CDKN2A/B, and PTEN, with distinct patterns observed between the two subtypes. The median overall survival (mOS) of GMB was 12.6 months. Mol-GBMs had a higher mOS than hist-GBMs, although not statistically significant (15.6 vs. 11.4 months, p=0.17). Older age, male sex, tumor involvement of deep brain structure or functional area, and genetic alterations in CDK4, CDK6, CIC, FGFR3, KMT5B, and MYB were predictors for a worse prognosis, while MGMT promoter methylation, maximal tumor resection, and treatment based on the Stupp protocol were predictive for better survival.

**Conclusion:**

The definition of GBM and its clinical, radiological, molecular, and prognostic characteristics have been altered under the current classification.

## 1 Introduction

Glioblastoma (GBM), the most common and lethal primary malignant brain tumor worldwide, is associated with a dismal prognosis (1, 2). Despite continuous efforts to improve glioblastoma-related outcomes, the median overall survival (mOS) is only 16 months, and the real-world 5-year survival rate is 6.8% (3). Moreover, glioblastomas invariably recur as aggressive, therapy-resistant relapses, highlighting the need for more effective treatment strategies (4, 5). Before the publication of the fifth edition of the World Health Organization (WHO) classification of central nervous system (CNS) tumors, known as the “WHO CNS5 classification” in June 2021, GBM classification primarily relied on histology. The traditional hallmarks of GBM were astrocytomas with the features of central pseudo-palisading necrosis and abnormal microvascular proliferation under the microscope (6).

Advancements in molecular testing have improved our understanding of the molecular categorization of gliomas, leading to a debate regarding whether GBM should be divided into isocitrate dehydrogenase (IDH)-mutant and IDH-wildtype subcategories (7, 8). IDH-mutant gliomas differ fundamentally from IDH-wildtype gliomas in terms of metabolism, epigenetics, biological behavior, aggressive infiltration, vulnerable population, and response to therapy (9–12). The Consortium to Inform Molecular and Practical Approaches to CNS Tumor Taxonomy (cIMPACT-NOW) proposed that IDH-wildtype diffuse gliomas that contain specific-altered genes strongly suggest a poor prognosis similar to GBM, even if the tumor exhibits low-grade histological features (11). Hence, in the WHO CNS5 classification scheme, molecular information and histological features have been incorporated for an integrated diagnosis that has substantially changed the overall classification of gliomas (13–15). Notably, one of the most significant changes in adult-type diffuse gliomas is the reclassification of ‘GBM, IDH-mutant, WHO grade 4’ as ‘Astrocytoma, IDH-mutant, WHO grade 4’ (10). GBM are now defined as diffuse, astrocytic gliomas that are IDH-wildtype and H3-wildtype, exhibiting one or more of the following histological or genetic features: microvascular proliferation, necrosis, telomerase reverse transcriptase (*TERT*) promoter mutation, epidermal growth factor receptor (*EGFR*) gene amplification, a concomitant gain of chromosome 7 and loss of chromosome 10 (+7/-10) (CNS WHO grade 4) (14, 16, 17). Histological diagnosis of GBM was determined by the presence of necrosis or microvascular proliferation, which is referred to as histological GBM (hist-GBM). IDH-wildtype diffuse astrocytic tumors without the histological features of GBM, which would have otherwise been classified as grade 2 or 3, are considered as molecular GBM (mol-GBM, WHO grade 4) if they harbor any of the following molecular abnormalities: TERT promoter mutation, EGFR amplification, or chromosomal + 7/−10 copy changes. An emerging concern is whether the results from previous studies fit GBMs as defined by the current classification system. It is also unclear whether the low-histological-grade mol-GBMs share the same clinical, radiological, pathological, molecular, and prognostic characteristics as hist-GBMs. Accumulating prospective biomarker alterations for GBM have been confirmed to have major consequences on the management of GBM, such as O-6-methylguanine-DNA methyltransferase (*MGMT*) promoter methylation, phosphatase and tension homolog (*PTEN*) loss, and histone methyltransferase *KMT5B* alteration (alias *SUV420H1*) (18–20). However, these gene alterations were not included in the current classification system for subtype categorization of gliomas due to the limited availability of evidence-based data in real-world settings(16, 21).

The present study aimed to comprehensively describe the clinical presentations, radiological features, pathological characteristics, and molecular alterations of GBMs according to the updated WHO CNS5 classification, as well as to assess patient survival and clinical determinants, and compare the aforementioned biological characteristics between hist-GBMs and mol-GBMs. To achieve these objectives, we collected and analyzed data from GBM patients treated at our institute over 11 years. In addition, we utilized public datasets to validate our findings, with the ultimate goal of establishing a more solid foundation for clinical recognition and decision-making of hist-GBMs and mol-GBMs.

## 2 Methods

### 2.1 Study participants

Patients with IDH-wildtype and H3-wildtype diffuse gliomas who underwent surgical tumor resection at Peking Union Medical College Hospital Neurosurgery between January 2011 and January 2022 were retrospectively screened. Patients’ tumors were defined as GBM (WHO grade 4), anaplastic astrocytomas (WHO grade 3), and diffuse astrocytomas (WHO grade 2) by the WHO CNS4 classification, while were recently re-classified as GBM, diffuse gliomas (NOS), or certain types of pediatric-type diffuse gliomas using the WHO CNS5 classification. In this analysis, only patients with GBMs as defined by the WHO CNS5 classification were enrolled and further analyzed.

The present study was approved by the institutional review board at Peking Union Medical College Hospital. Written informed consent was obtained from every subject.

### 2.2 Definition of histological and molecular glioblastomas

Hist-GBMs were defined as IDH-wildtype and H3-wildtype high-grade diffuse gliomas with microvascular proliferation and/or intratumoral necrosis. Mol-GBMs were defined as histological WHO grade 2-3 *IDH*-wildtype and *H3*-wildtype diffuse gliomas with *TERT* promoter mutation, *EGFR* amplification, or +7/-10 chromosome variations. When neither of the aforementioned criteria was met, the tumor was classified as diffuse gliomas (NEC) or pediatric-type diffuse glioma, including MYB- or MYBL1-altered low-grade diffuse astrocytoma and H3-wildtype and IDH-wildtype pediatric-type high-grade diffuse glioma.

### 2.3 Data acquisition

Clinical information, radiological characteristics, and histological data were extracted from the electronic medical record (**Supplementary Table 1**). Sixty molecular markers of interest, including gene alterations and chromosome copy number variations, were analyzed using the next-generation sequencing method, the PCR-based assay, and the fluorescence *in situ* hybridization. These markers were chosen to differentiate subtypes of diffuse gliomas or to predict patient prognosis.

### 2.4 Targeted sequencing

Genomic DNA from formalin-fixed paraffin-embedded (FFPE) tissue was isolated using the DNA FFPE Tissue Kit (Qiagen #56404). A custom glioma next-generation sequencing (NGS) panel was designed to investigate detailed molecular alterations in these patients. The 60-gene NGS panel contains some interested well-known genes and recently described genes that were associated with the diagnosis, grading, and treatment responses of gliomas, including *IDH1*, *IDH2*, *H3F3A*, *HIST1H3B*, *HIST1H3C*, *EGFR*, *TERT*, *CDKN2A/B*, *CDK4*, *CDK6*, *CIC*, *FGFR3*, *MYB*, *MYBL1*, *KMT5B*, *MGMT*, *PTEN*, chromosome copy number variations, etc (**Supplementary Table 1**). According to the manufacturer’s specifications, The DNA library was constructed by DNA repair (NEBNext^®^ FFPE DNA Repair Mix, #M6630S), DNA fragmentation and end-repair (TIANSeq Fragment/Repair/Tailing Module, #NG301), and hybridization (KAPA HyperCapture Reagent Kit, #09075828001). Among all of the 209 FFPE tissues that had been acquired and sent to NGS test, only 78 cases had adequate tissue and passed the quality control and were analyzed for the molecular part in this article. After the quality assessment, the library was sequenced with the Illumina platform and the mean read depth was >500X. GRCH38 was used as the reference genome and subsequent analyses.

### 2.5 Validation dataset

RNA-sequencing and clinicopathological data of GBMs were downloaded from the Chinese Glioma Genome Atlas (CGGA; http://www.cgga.org.cn/; Dataset ID: mRNAseq_693, mRNAseq_325) to obtain a validation dataset. A total of 280 patients who were diagnosed with hist-GBMs according to the WHO CNS5 classification were enrolled. Information included were gender, age, OS, and data of molecular markers. The optimal cutoff values of the expression level of each parameter were calculated using the “surv_cutpoint” function of the “survminer” R package, and the patients were consequently divided into the high and low expression groups for validation.

### 2.6 Statistical analyses

Clinical, radiological, and pathological data expressed as categorical variables were shown as numbers and percentages, and continuous variables were presented as the means ± standard deviations or medians plus interquartile range according to data distribution. Comparisons of categorical variables were performed using the chi-squared test. Student’s t-test was used to assess the differences between normally distributed continuous variables, while the Mann-Whitney U test was used for variables that failed the normality test. For most parameters, all patients were included in the analysis. However, only the patients with available and complete data were enrolled for analysis of several variables. *p*<0.05 indicated statistical significance. The Sankey diagram was used to visualize the changes in tumor classifications using the WHO CNS4 and the WHO CNS5 classification systems. The waterfall chart was performed to illustrate the GBM samples’ molecular alterations and IHC expressions. Median OS and the 95% confidence interval (95%CI) were calculated for hist-GBMs and mol-GBMs and patient subgroups with distinct clinical, radiological, or molecular features. Kaplan-Meier curves were drawn to illustrate the OS, and a log-rank *p*<0.05 indicated a significant difference in survival. Comprehensive sub-analyses were performed by using both the uni- and multi-variable hazard ratio (HR) models to evaluate the associations between biological variables and OS. The above analyses were performed for all glioblastomas, hist-GBMs, and mol-GBMs. To further validate the study’s results, all variables significantly associated with patient OS were included in the validation set analysis, where univariate subgroup analysis was performed for each factor using the RNA-sequencing data. SPSS Statistics (version 26.0, IBM, USA) was used to conduct the data analyses, and RStudio (PBC & Certified B Corp.^®^, USA) was used to generate graphs.

## 3 Results

### 3.1 Changes in the classification of IDH-wildtype diffuse gliomas

**Figure 1:**
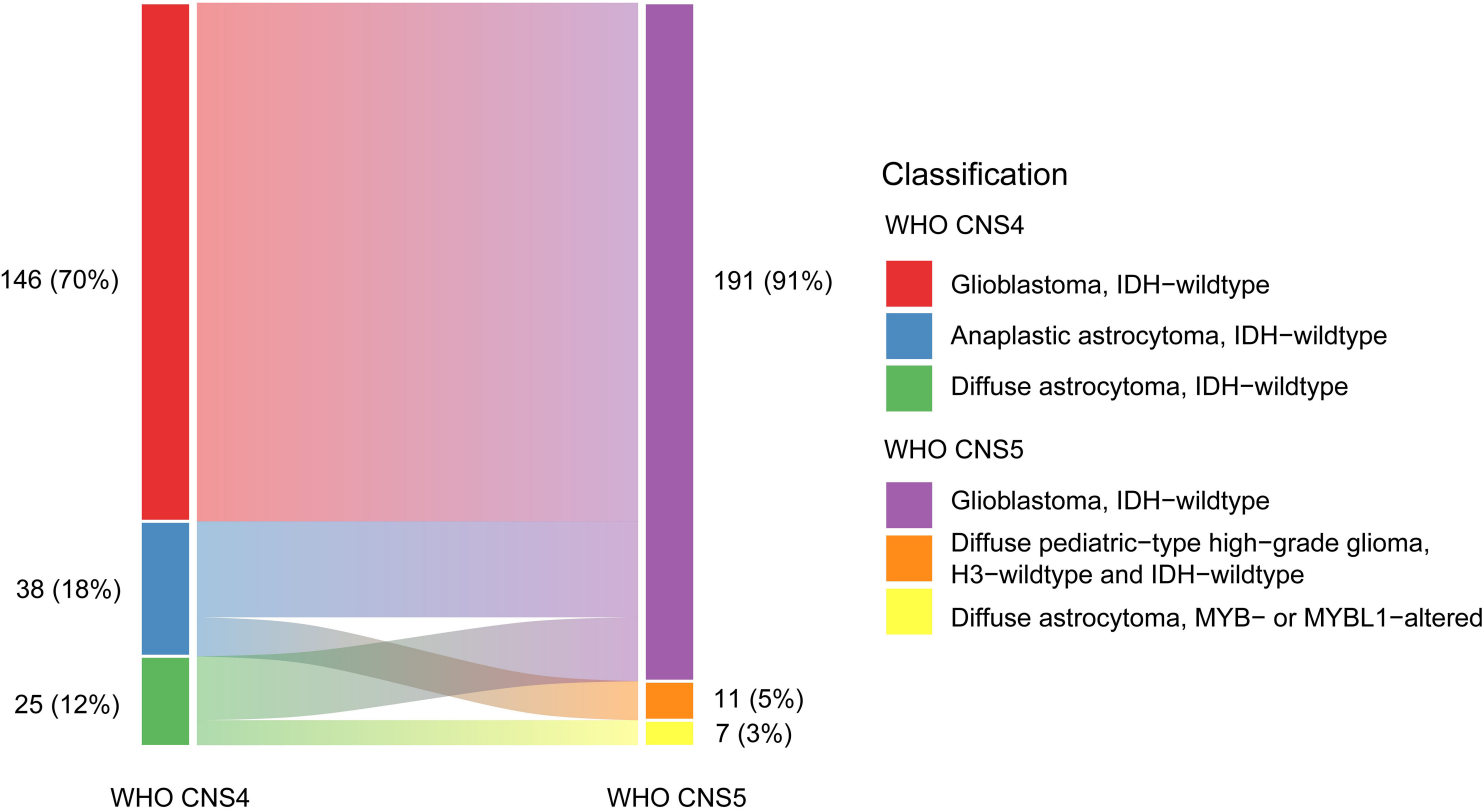
Changes of subtypes of IDH-wildtype diffuse gliomas from the WHO CNS4 to the WHO CNS5 classification. Each rectangle represents a subtype of IDH-wildtype diffuse gliomas. The blocks on the left include glioblastomas (WHO grade 4), anaplastic astrocytomas (WHO grade 3), and diffuse astrocytoma (WHO grade 2) classified using the WHO CNS4 classification system. The blocks on the right include glioblastomas, H3-wildtype and IDH-wildtype diffuse pediatric-type high-grade gliomas, and *MYB*- or *MYBL1*-altered diffuse pediatric-type astrocytomas classified using the WHO CNS5 classification system.

Among 209 IDH-wildtype diffuse gliomas (146 GBM, 38 anaplastic astrocytomas, and 25 diffuse astrocytomas) according to the WHO CNS4 classification, 191 (91%) were reclassified as GBM (146 hist-GBMs and 45 mol-GBMs), 11 (5%) as H3-wildtype and IDH-wildtype pediatric-type high-grade diffuse gliomas, and 7 (3%) as MYB/MYBL1-altered pediatric-type low-grade diffuse astrocytomas based on the WHO CNS5 classification system (**Figure 1**). Out of 38 IDH-wildtype anaplastic astrocytomas (71%) and 25 diffuse astrocytomas (72%), 27 (71%) and 18 (72%) respectively were reclassified as mol-GBMs due to the presence of key genetic features. The number of molGBMs with one, two, three genetic alterations were 11 (24.4%), 18 (40.0%), and 16 (35.6%), respectively. The remaining IDH-wildtype anaplastic astrocytomas (29%) and diffuse astrocytomas (28%) were reclassified as pediatric-type diffuse high-grade and low-grade gliomas, respectively.

### 3.2 Clinical presentations

The average age of GBM patients was 55.5 years, with males comprising 52.4% of all samples (**Table 1**). At diagnosis, 67.5% of patients exhibited neurological symptoms, with motor dysfunction (37.2%) and aphasia (21.5%) being the most common. Intracranial hypertension was found in 48.2% of patients, while 20.9% had a history of epilepsy. The median duration of the disease before admission was 5 weeks, and the median Karnofsky performance scale was 80. The majority of patients underwent gross total resection (61.8%; 36.1% for those invading deep brain structures), and 64.1% received the standard Stupp treatment protocol following surgery.

**Table 1:** Clinical, radiological, and pathological features of patients with histological or molecular glioblastomas.

	All Glioblastomas (n=191)	Histological Glioblastomas (n=146)	Molecular Glioblastomas (n=45)	P Value
Age at diagnosis, year	55.5 ± 14.7	56.9 ± 14.1	50.8 ± 15.8	**0.015**
Age ≥ 60, n/%	80, 41.9%	66, 45.2%	14, 31.1%	0.094
Age ≥ 70, n/%	31, 16.2%	27, 18.5%	4, 8.9%	0.127
Male, n/%	100, 52.4%	75, 51.4%	25, 55.6%	0.623
Body mass index, kg/m^2^	23.8 ± 3.3	23.8 ± 3.3	23.8 ± 3.2	0.910
Neurologic impairment, n/%	129, 67.5%	102, 69.9%	27, 60.0%	0.217
Motor dysfunction, n/%	71, 37.2%	63, 43.2%	8, 17.8%	**0.002**
Sensory dysfunction, n/%	16, 8.4%	11, 7.5%	5, 11.1%	0.653
Visual field defect, n/%	16, 8.4%	13, 8.9%	3, 6.7%	0.868
Aphasia, n/%	41, 21.5%	33, 22.6%	8, 17.8%	0.491
Intracranial hypertension, n/%	92, 48.2%	70, 47.9%	22, 48.9%	0.912
Epilepsy, n/%	40, 20.9%	25, 17.1%	15, 33.3%	**0.019**
Memory deterioration, n/%	19, 9.9%	15, 10.3%	4, 8.9%	1.000
Changes in personality, n/%	7, 3.7%	6, 4.1%	1, 2.2%	0.892
Disease duration before admission, week	5 (2, 13)	4 (2, 12)	6 (3, 24)	0.188
Baseline Karnofsky performance scale, n	80 (80, 95)	80 (80, 95)	90 (80, 95)	0.496
Extent of surgical resection				
Gross total resection, n/%	118, 61.8%	98, 67.1%	20, 44.4%	**0.006**
Subtotal resection, n/%	34, 17.8%	20, 13.7%	14, 31.1%	**0.008**
Biopsy, n/%	39, 20.4%	28, 19.2%	11, 24.4%	0.444
Postoperative treatment*				
With the Stupp protocol, %	75/117, 64.1%	58/90, 64.4%	17/27, 63.0%	0.888
Without the Stupp protocol, %	42/117, 35.9%	32/90, 35.6%	10/27, 37.0%	0.888
Tumor location (if involved) *				
Frontal lobe, n/%	75, 46.3%	56, 43.4%	19, 57.6%	0.145
Temporal lobe, n/%	68, 42.0%	52, 40.3%	16, 48.5%	0.396
Parietal lobe, n/%	53, 32.7%	43, 26.5%	10, 30.3%	0.741
Occipital lobe, n/%	30, 18.5%	26, 20.2%	4, 12.1%	0.289
Insular lobe/thalamus/callosum, n/%	36, 22.2%	28, 21.7%	8, 24.2%	0.754
Multiple lobes involved, n/%	85, 52.5%	64, 49.6%	21, 63.6%	0.150
Subtentorial, n/%	2, 1.2%	2, 1.6%	0, 0%	1.000
Tumor connection to the functional regions*				
No, n/%	72, 44.4%	58, 45.0%	14, 42.4%	0.794
Yes, n/%	90, 55.6%	71, 55.0%	19, 57.6%	0.794
Motor cortex and tract, n/%	50, 30.9%	43, 33.3%	7, 21.2%	0.179
Sensory cortex and tract, n/%	31, 19.1%	21, 16.3%	10, 30.3%	0.068
Language area and tract, n/%	18, 11.1%	16, 12.4%	2, 6.1%	0.469
Visual pathway, n/%	14, 8.6%	11, 8.5%	3, 9.1%	1.000
T1-weighted image*				
Hypointensity, n/%	103/63.6%	81, 62.8%	22, 66.7%	0.680
Mixed (hypo-, iso-, and hyperintensity), n/%	57/35.2%	47, 36.4%	10, 30.3%	0.510
Isointensity, n/%	1/0.6%	0, 0%	1, 3.0%	0.204
Hyperintensity, n/%	1/0.6%	1, 0.8%	0, 0%	1.000
T2-weighted image*				
Hyperintensity, n/%	85/52.5%	66, 51.2%	19, 57.6%	0.510
Mixed (hypo-, iso-, and hyperintensity), n/%	76/46.9%	63, 48.8%	13, 39.4%	0.332
Isointensity, n/%	1/0.6%	0, 0%	1, 3.0%	0.204
Hypointensity, n/%	0/0%	0, 0%	0, 0%	1.000
Multiple tumors, n/%*	30, 18.5%	25, 19.4%	5, 15.2%	0.577
Contrast enhancement, n/%*	149/92.0%	123, 95.3%	26, 78.8%	**0.006**
Intratumoral necrosis, n/%*	131/80.9%	110, 85.3%	21, 63.6%	**0.005**
Maximal tumor diameter, cm*	4.1 ± 1.7	4.2 ± 1.7	3.6 ± 1.5	0.092
Edema extending from tumor margin, cm*	1.8 (1.0, 2.6)	2.0 (1.1, 2.6)	1.1 (0.6, 2.7)	0.167
Maximal diameter of necrosis, cm*	2.1 (0.8, 3.5)	2.3 (1.0, 3.7)	1.0 (0, 2.9)	**0.002**
Pathology under the microscope				
Ki-67 index, %, median, interquartiles	30 (15, 50)	30 (20, 50)	10 (3, 25)	**<0.001**
WHO grade 4, np/nt, %	146/191, 76.4%	146/146, 100%	0/45, 0%	**<0.001**
WHO grade 3, np/nt, %	27/191, 14.1%	0/146, 0%	27/45, 60.0%	**<0.001**
WHO grade 2, np/nt, %	18/191, 9.4%	0/146, 0%	18/45, 40.0%	**<0.001**
Immunohistochemical presentations				
ATRX expression, np/nt, %	83/91, 91.2%	68/74, 91.9%	15/17, 88.2%	0.996
TP53 expression, np/nt, %	80/147, 54.4%	67/119, 56.3%	13/28, 46.4%	0.345
GFAP expression, np/nt, %	166/172, 96.5%	132/137, 96.4%	34/35, 97.1%	1.000
Olig2 expression, np/nt, %	105/112, 93.8%	82/88, 93.2%	23/24, 95.8%	1.000
S-100 expression, np/nt, %	151/155, 97.4%	121/124, 97.6%	30/31, 96.8%	1.000
Molecular alterations				
* MGMT* promoter methylation, np/nt, %	52/145, 35.9%	40/110, 36.4%	12/35, 34.3%	0.823
* TERT* promoter mutation, np/nt, %	136/182, 74.7%	100/140, 71.4%	36/42, 85.7%	0.507
* EGFR* amplification, np/nt, %	46/80, 57.5%	27/45, 60.0%	19/35, 54.3%	0.785
Chromosome- 7 gain/10 loss, np/nt, %	73/77, 94.8%	38/41, 92.7%	35/36, 97.2%	0.703
* CDKN2A/B* homozygous deletion, np/nt, %	47/68, 69.1%	27/37, 73.0%	20/31, 64.5%	0.452

*In this section, analysis was based on the patients who had complete follow-up data of the postoperative treatments. The bold values in the "P value" column indicated significant P values (<0.05).

Compared with hist-GBMs, patients with mol-GBMs were diagnosed at a younger age (50.8 ± 15.8 vs. 56.9 ± 14.1 years, *p*=0.015), had a lower incidence of motor dysfunction (17.8% vs. 43.2%, *p*=0.002), a higher incidence of epilepsy (33.3% vs. 17.1%, *p*=0.019) before admission, and a lower rate of gross total resection (44.4% vs. 67.1%, *p*=0.006).

### 3.3 Radiological features

The frontal (46.3%) and temporal (42.0%) lobes were the most frequently affected regions, and 52.5% of tumors invaded more than one lobe (**Table 1**). 22.2% of included GBMs invaded deep structures (thalamus or callosum), and two were originated from the subtentorial region. Functional brain regions were involved in 55.6% of patients, with the motor cortex and tract being the most affected (30.9%). GBM appeared hypo-intensive (63.6%) or mixed (35.2%) on T1-weighed images and hyper-intensive (52.5%) or mixed (46.9%) on T2-weighed images. The average maximal tumor diameter was 4.1 ± 1.7 cm. Most tumors exhibited contrast-enhanced (92.0%) and intratumoral necrosis (80.9%).

Compared with hist-GBMs, mol-GBMs were less likely to have contrast-enhancement (78.8% vs. 95.3%, *p*=0.006) and intratumoral necrosis (63.6% vs. 85.3%, *p*=0.005), and the median diameter of necrosis inside mol-GBMs was smaller (1.0 vs. 2.3cm, *p*=0.002).

### 3.4 Under-the-microscope pathological characteristics

The median ki-67 index was 30% for all GBMs (**Table 1**). Hist-GBMs had a higher median ki-67 index compared to mol-GBMs (30% vs. 10%, *p*<0.001). Among mol-GBMs, 60% were upgraded from WHO grade 3 IDH-wildtype anaplastic astrocytomas, and the other 40% originated from WHO grade 2 IDH-wildtype diffuse astrocytomas. No differences in immunohistochemical expressions were found between hist-GBMs and mol-GBMs.

### 3.5 Genetic alterations and IHC expression

**Figure 2:**
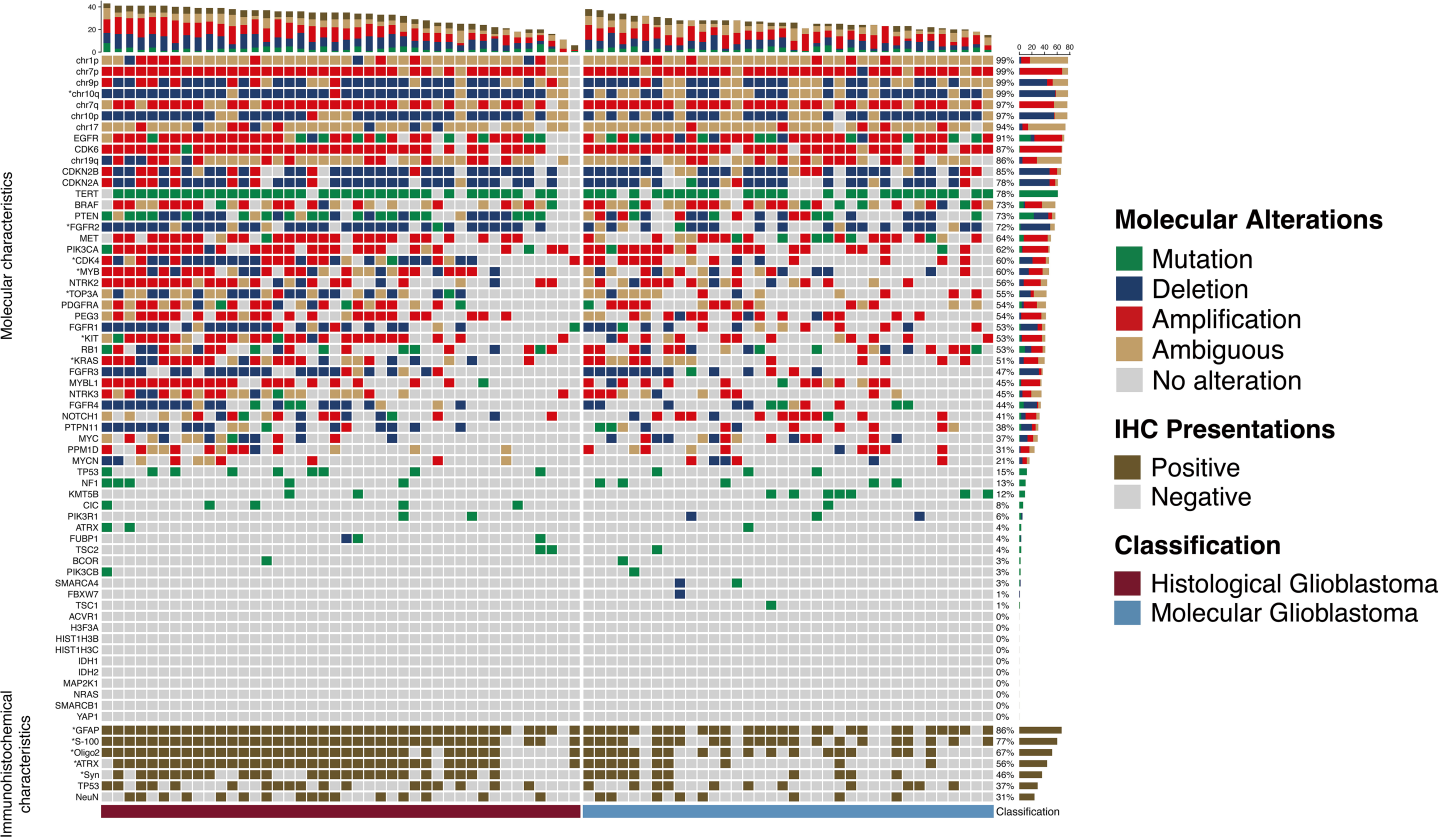
Molecular alterations and immunohistochemical presentations of histological and molecular glioblastomas. Molecular alterations and immunohistochemical presentations of histological and molecular glioblastomas. Each column represents an individual patient, and the classification of tumors is displayed at the bottom. Each row indicates a genetic or immunohistochemical parameter, and these parameters are listed from top to bottom based on the frequency of genetic alterations and immunohistochemical expressions. Genetic mutation is shown as green, deletion is shown as blue, amplification is shown as red, ambiguous is shown as yellow, and positive immunohistochemical staining is shown as brown. The frequencies of mutation, deletion, and amplification of each gene are shown in the right histogram. Differences in molecular alterations and immunohistochemical expressions are compared, and an asterisk (*) to the left indicates a p<0.05.

Mutation was considered the main type of genetic alteration in *TERT* promotor, *TOP3A*, and *BRAF*; while deletions were predominantly observed in chr-10p/10q/9p, *CDKN2A*/B, *PTEN*, *PTPN11*, and *FGFR1/2/4.* Amplification was most commonly seen in chr-7p/q, *EGFR*, *CDK6/4*, *MYB/MYBL1*, *MET*, *PIK3CA*, *NTRK2*, *PDGFRA*, *PEG3*, *KIT*, *NOTCH1*, *PPM1D*, RB1, and *KRAS* (**Figure 2**). Immunohistochemistry results indicated that GFAP, S-100, Olig-2, and ATRX were positive in more than 90% of glioblastomas (**Table 1**).

Compared with mol-GBMs, hist-GBMs had higher rates of genetic alterations in *PTEN*, *TOP3A*, *CDK4*, *MYB*, *KIT*, *KRAS*, and *NTRK3* (**Supplementary Table 2**).

### 3.6 Overall survival of glioblastomas as defined by the WHO CNS5 classification system

**Figure 3:**
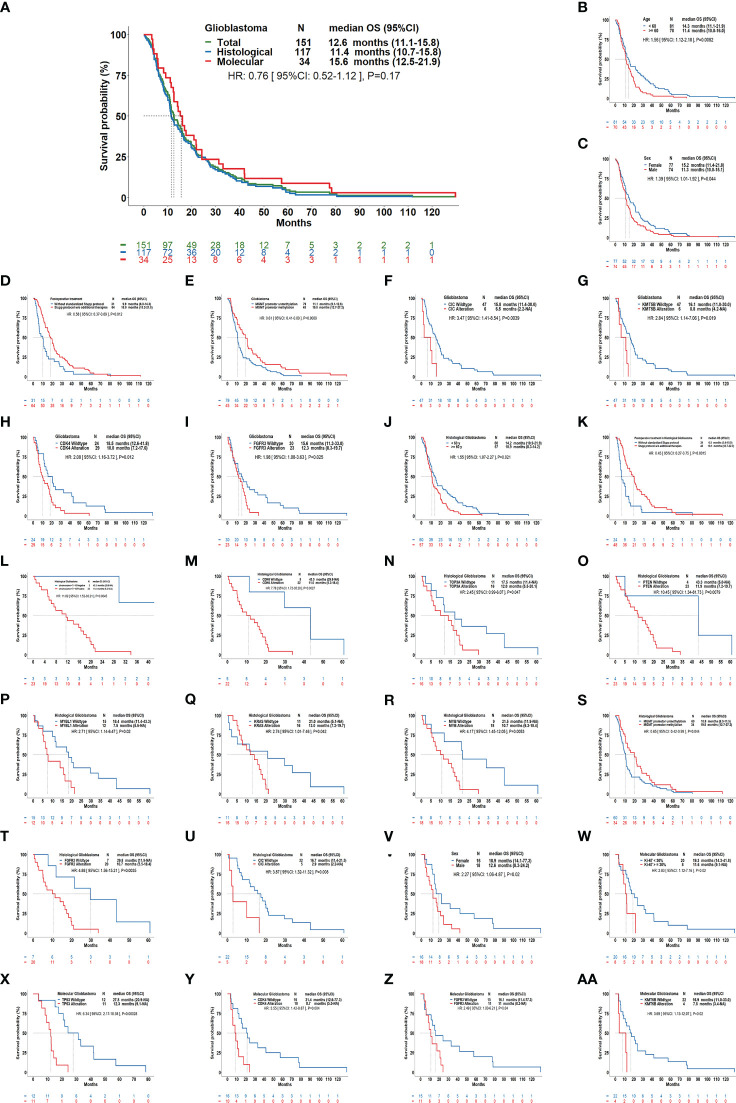
Overall survival of patients with glioblastomas using the current classification scheme and its clinical relevance. **(A)**: Kaplan–Meier curves showing the overall survival (OS) of molecular glioblastomas vs. histological glioblastomas (median OS: 11.4 months vs. 15.6 months, hazard ratio [HR]: 0.76, *P*=0.17), and the OS curve for all glioblastomas (median OS: 12.6 months). In all glioblastomas **(B-I)**, age ≥60 years, males, postoperative treatment without Stupp protocol, *MGMT* promotor unmethylation, alteration of CIC, *KMT5B*, *CDK4*, *FGFR3* were correlated with a shorter OS. In histological glioblastomas **(J-U)**, age ≥60 years, postoperative treatment without standardized Stupp protocol, having chromosome 7 gain and chromosome 10 loss, alteration of *CDK6*, *TOP3A*, *PTEN*, *MYBL1*, *KRAS*, *MYB*, *FGFR2, CIC, MGMT*, promotor unmethylation were correlated with a shorter OS. In molecular glioblastomas **(V–AA)**, male sex, ki-67≥30%, alteration of *TP53*, *CDK4*, *FGFR3*, and *KMT5B* were correlated with a shorter OS.

Our results showed that the median OS for all GBMs was 12.6 months (95%CI: 11.1-15.8); it was 15.6 months (95%CI: 12.5-21.9) for mol-GBMs and 11.4 months (95%CI: 10.7-15.8) for hist-GBMs (**Figure 3**). However, despite that mol-GBMs showed better survival, no significant difference was found between the two subtypes (HR=0.76, 95%CI: 0.52-1.12; *p*=0.17). Additionally, the median OS of patients with pediatric-type diffuse gliomas (the other IDH-wildtype gliomas in this study) was 55.1 months, significantly longer than that of patients with mol-GBMs (*p*=0.006).

Patients aged ≥60 years (11.4 vs. 14.3, months; *p*=0.008), male patients (11.3 vs. 15.2, months; *p*=0.044), and those who did not undergo the standard Stupp treatment protocol (9.9 vs. 18.0, months; *p*=0.012) had a shorter OS than the others. MGMT promotor unmethylation, FGFR3 alteration, CIC alteration, KMT5B alteration, and CDK4 alteration were molecular features that predicted a shorter OS in GBM (*p*<0.05).

### 3.7 Clinical correlations of OS in histological and molecular glioblastomas

In hist-GBMs, older age (≥60 years), not receiving the Stupp treatment protocol, +7/-10 chromosome copy-number changes, *MGMT* unmethylation, and alterations in *CDK6*, *TOP3A*, *PTEN*, *MYB*, *MYBL1*, *KRAS*, *FGFR2*, and *CIC* were significantly correlated with a shorter OS. In mol-GBMs, male sex, ki-67 ≥30%, and alterations in *TP53*, *CDK4*, *FGFR3*, and *KMT5B* were significantly correlated with a shorter OS (**Figure 3**). No other molecular correlations with OS were found (**Supplementary Figures 1–3**).

Multivariable regression analyses showed that older age, male sex, tumor involvement of functional brain areas and deep brain structures, and alterations in *CDK4*, *CDK6*, and *KMT5B* were predictive of a decreased OS, while maximal surgical resection as compared to biopsy, treatment with the Stupp protocol, and *MGMT* promoter methylation was predictive of a prolonged OS (**Table 2**). *CIC* and *MYB* alterations were predictive of a decreased OS in hist-GBMs. Presentation of motor dysfunction before surgery, a higher ki-67 index, *TP53* alteration, and *FGFR3* alteration were predictive of a decreased OS in mol-GBMs.

**Table 2:** Independent clinical, radiological, and pathological factors associated with overall survival of patients with glioblastomas.

	All Glioblastomas		Histological Glioblastomas		Molecular Glioblastomas	
	HR (95% CI)	P Value	HR (95% CI)	P Value	HR (95% CI)	P Value
Age at diagnosis	1.017 (1.004, 1.030)	**0.010**	1.015 (1.000, 1.030)	**0.045**		
Male	1.482 (1.045, 2.103)	**0.027**			3.930 (1.574, 9.808)	**0.003**
Preoperative motor dysfunction					4.167 (1.523, 11.398)	**0.005**
Insular lobe/thalamus/callosum involvement	2.073 (1.352, 3.179)	**0.001**	2.286 (1.406, 3.717)	**0.001**		
Tumor related to the functional region	1.877 (1.296, 2.719)	**0.001**	1.567 (1.050, 2.339)	**0.028**	4.678 (1.500, 14.591)	**0.008**
Extent of resection						
Biopsy	reference		reference		reference	
Subtotal resection	0.390 (0.232, 0.655)	**<0.001**	0.417 (0.235, 0.863)	**0.016**	0.175 (0.055, 0.563)	**0.003**
Gross total resection	0.348 (0.226, 0.535)	**<0.001**	0.374 (0.217, 0.636)	**<0.001**	0.108 (0.033, 0.349)	**<0.001**
Postoperative treatment						
Without standardized Stupp protocol	reference		reference			
Stupp protocol w/o additional therapies	0.577 (0.373, 0.891)	**0.013**	0.450 (0.272, 0.746)	**0.002**		
Ki-67 index					1.028 (1.002, 1.054)	**0.036**
*p53* alteration					8.970 (2.353, 34.199)	**0.001**
*MGMT* promoter methylation	0.596 (0.359, 0.989)	**0.045**				
*CDK4* alteration	2.366 (1.280, 4.373)	**0.006**			5.964 (1.836, 19.370)	**0.003**
*CDK6* alteration	25.813 (1.645, 405.012)	**0.021**	15026 (171.311, 1.3x10^6)	**<0.001**		
*CIC* alteration			6.112 (1.209, 30.901)	**0.029**		
*FGFR3* alteration					4.265 (1.230, 14.786)	**0.022**
*KMT5B* alteration	3.452 (1.294, 9.207)	**0.013**			21.293 (3.447, 131.521)	**0.001**
*MYB* alteration			415.989 (17.899, 9668.181)	**<0.001**		

The bold values in the "P value" column indicated significant P values (<0.05).

### 3.8 Validation of the clinical correlations of OS using the CGGA dataset

**Figure 4:**
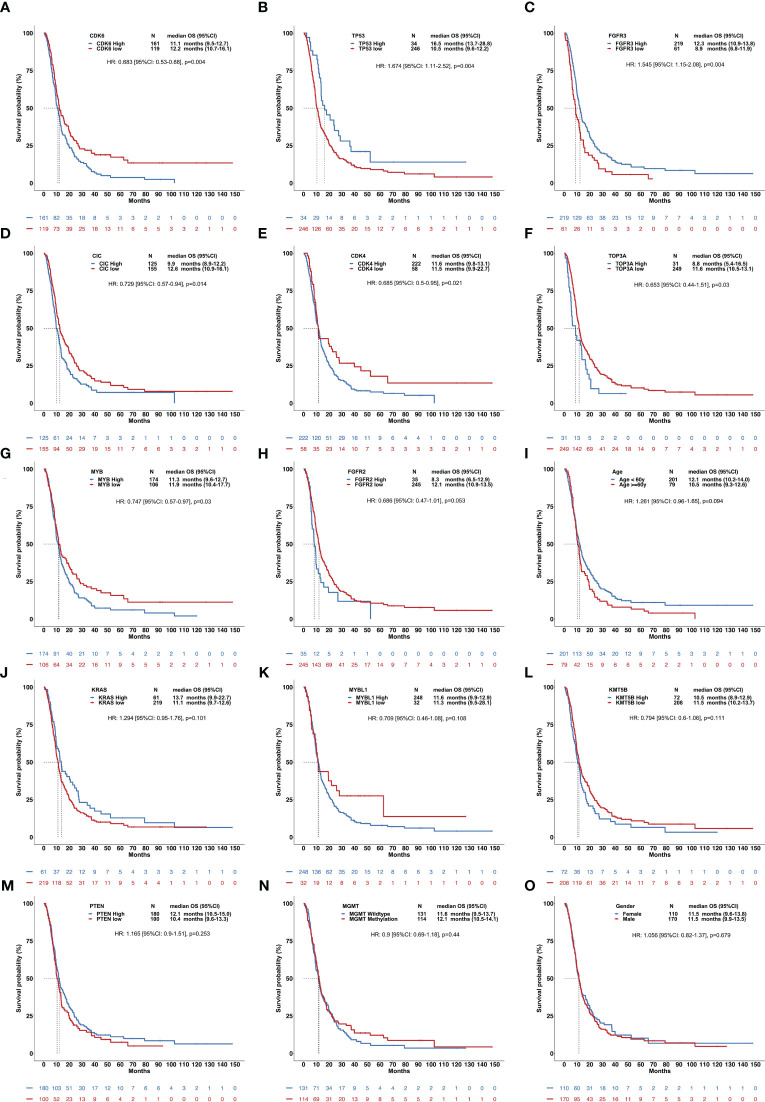
Clinical relevance of overall survival of patients with histological glioblastomas using the validation set. This figure included 15 factors showing the influence of gene expression and other clinical parameters on the OS **(A-O)**, including *CDK6*, *TP53*, *FGFR3*, *CIC*, *CDK4*, *TOP3A*, *MYB*, *FGFR2*, age, *KRAS*, *MYBL1*, *KMT5B*, *PTEN*, *MGMT* methylation, and sex.

In the RNA-sequencing validation set, high expression in *CDK6*, *CIC*, *CDK4*, *TOP3A*, and *MYB* and low expression in *TP53* and *FGFR3* were all significantly correlated with a shorter OS in hist-GBMs (**Figure 4**).

## 4 Discussion

Since the release of the 5^th^ edition of the WHO classification of CNS tumors, the categorization of adult-type gliomas has been altered drastically. Among primary CNS tumors, GBM has undergone the greatest changes. In the present study, over 70% of previously defined grade 2-3 IDH-wildtype diffuse gliomas were re-classified as grade 4 GBMs due to specific molecule alterations, while the remaining 30% of them were re-classified into other subtypes. Furthermore, the previously recognized IDH-mutant GBM have been re-classified as astrocytoma, WHO grade 4. Overall, the composition of GBMs nowadays differs greatly from earlier ones in that all IDH-mutant astrocytomas are excluded, and a large number of WHO grade 2-3 IDH-wildtype gliomas with specific molecular features are involved (14). Consequently, previous conclusions on GBM’s clinical, radiographic, molecular, and prognostic features might no longer be compatible with the current clinical practice. Additionally, since mol-GBMs has become a newly defined group of tumors, increasing uncertainty about its diagnostic and therapeutic principles has emerged (22). Hence, it is imperative and crucial to reassess the characteristics of GBMs in the context of the updated classification. In this study, we conducted genetic analyses on IDH-wildtype diffuse gliomas resected at our institute. A total of 191 GBMs defined by the WHO CNS5 classification were identified and included in the study. The study also monitored the survival rates of the patients and investigated potential correlations between biological parameters and their prognoses. To examine the molecular changes in glioma patients, we developed and utilized a specialized NGS panel to compare molecular and histological characteristics of GBMs. The objective of this study was to provide valuable insights for the clinical treatment of this novel subset of mol-GBMs.

Based on this new classification, current GBMs were diagnosed at a younger age and were more predominant in male patients. Also, more than two-thirds were accompanied by neurological deficits, particularly motor dysfunction, which differed slightly from the prior studies (23, 24). Older age, especially in hist-GBMs, and male sex, particularly in mol-GBMs, were found to be independently associated with patient survival. Preoperative motor dysfunction also served as a negative prognostic factor in mol-GBMs. Individuals with mol-GBMs were diagnosed 6 years earlier than those with hist-GBMs. Patients with mol-GBMs had a lower incidence of motor dysfunction and higher incidence of preoperative seizures than those with hist-GBMs, which might be due to the aggressive behavior of the latter, tending to compress and invade surrounding tissue rapidly, thus speeding up the onset of neurological symptoms while delaying the development of epilepsy (25, 26). It is well known that IDH mutation is more common in low-grade gliomas (LGGs) with a better prognosis than high-grade gliomas and that epilepsy is significantly correlated with LGGs (27). Although mol-GBMs are IDH-wildtype, they are histologically classified as relatively-low-grade. Certain molecular features, other than IDH mutation, may correlate with seizures in mol-GBMs; however, further investigation is needed. In our analysis, a higher number of patients with hist-GBMs than those with mol-GBMs received gross total resection. This might be due to the lack of angiogenic presentations and intratumoral necrosis, making it challenging to clearly recognize the tumor border of mol-GBMs during surgery. Another explanation might be that the postoperative radiological evaluation of the residual tumor/edema is difficult for mol-GBMs, since many of these tumors do not show radiologically contrast-enhancement, increasing the possibility of misinterpreting gross total resection as subtotal resection. For both subtypes, the greater the extent of tumor resection, the longer the OS. Although the Stupp treatment protocol was predictive of a better prognosis for hist-GBMs, our result showed it was not a significant protective factor for survival in mol-GBMs. The optimal treatment strategy for this new subtype of GBMs remains to be determined and requires further clinical trials.

The frontal and temporal lobes are the most commonly involved areas in GBM, they are the largest brain lobes (28). In the present study, tumor involvement of functional brain regions was found to be an independent predictor of a worse prognosis, while the motor cortex/tract was the most affected functional area, consistent with existing literature (29). Involvement of the insular lobe, thalamus, and callosum also predicted a shortened OS, especially in hist-GBMs. The pathological findings contradict the imaging findings of central nervous system necrosis. While we observed imaging enhancement and necrosis in the mol-GBMs, their pathological findings did not show necrosis. It has been noted that the imaging results did not perfectly match the pathology findings. The presentations of T1WI and T2WI of GBMs using the current classification shared many similarities with the previous classification (30). Previous studies have found that contrast enhancement in mol-GBMs is a marker of aggressiveness (17). In our study, we found that the rates of contrast enhancement and intratumoral necrosis were lower, and the maximal diameter of intratumoral necrosis was shorter in mol-GBMs, which might be due to their relatively low histological grade.

The identification of specific biomarkers plays a crucial role in determining the prognosis of a medical condition and selecting the most suitable therapeutic approach (31). The occurrence of IDH mutation is an early event in the development of glioma and has significant consequences for glioma progression and treatment response. The *IDH* gene has emerged as a promising molecular biomarker for predicting the response to TMZ treatment and has been validated as a standalone favorable prognostic indicator in patients with GBMs (32). Histone H3s mutations may drive pediatric GBM (33). Copy-number change in certain chromosomes, or chromosomal instability, is one of the hallmarks of GBMs (34). In our cohort, changes in chromosomes, especially chr-1, 7, 9, and 10, occurred in over 90% of GBMs. Besides, alterations of *EGFR*, *TERT*, *CDK6*, *PTEN*, *BRAF*, *FGFR2*, and *CDKN2A/B* were found in over 70% of GBMs. The current classification for GBM is mainly based on *IDH*, *EGFR*, *TERT*, and chromosome copy-number changes. Giulia et al. have underscored the significance of isolated *TERT* promoter mutation in glioma and its correlation with improved prognosis, amidst the plethora of molecules under investigation (35). As our understanding of molecular pathogenesis evolves, additional biomarkers may be added to improve these tumors’ classification, survival prediction, and targeted therapy.

The rates of alterations of *PTEN*, *CDK4*, *MYB*, *TOP3A*, *KIT*, *KRAS*, and *NTRK3* were higher in hist-GBMs than in mol-GBMs. Previous studies have reported that *PTEN* alteration is associated with therapeutic resistance in GBMs (36). *CDK4*/*6*, which regulates the cell cycle, are an important factor for the tumorigenesis of GBMs (37). *MYB* is a transcription factor from the myeloblastosis family linked to the development and progression of GBMs (38). *TOP3A* levels are higher in GBMs compared to LGGs (39). *KIT* is elevated at the time of both the first diagnosis and the recurrence of GBMs (40). *KRAS* alteration is identified as an oncogene for GBMs, and its over-expression has a crucial role in glioma cell growth and proliferation (41). *NTRK1-3* genes have been identified as potential driver mutation partners in GBMs (42). Approximately 12% of mol-GBM in this cohort showed no nuclear ATRX staining. Although this is not identical to our concept, ATRX expression was reported in other publications, showing that not all GBMs expressed ATRX (43). Previous studies reported that ATRX-deficient GBM cells show enhanced sensitivity to irradiation(44). The differences in molecular alterations between hist-GBMs and mol-GBMs revealed that this might be the key biological mechanism behind their clinical disparities.

Few studies assessed the real-world survival of GBM patients based on the WHO CNS5 classification (17, 45, 46). In this cohort, the median OS of GBMs was 12.6 months, which is shorter than 16 months calculated using the WHO CNS4 classification (47). The shorter OS may be partly due to the exclusion of IDH-mutant astrocytomas with relatively better survival and the introduction of mol-GBMs that are more infiltrative and less responsive to TMZ chemotherapy. In addition, whether mol-GBMs with a relatively low histological grade have the same OS as hist-GBMs with necrosis and/or microvascular proliferation are less known. In our cohort, the median OS of mol-GBMs was slightly higher than that of hist-GBMs, but the difference was not statistically significant. This finding is consistent with the reports by Ramos-Fresnedo (17) and Grogan (45) but not Ostrom (46), and might be explained by the small sample size in this study. However, since the OS curve of mol-GBMs with only 34 patients remained above the hist-GBMs curve, the non-significant OS difference might be false-negative if the sample size was increased.

The WHO CNS5 classification places greater emphasis on molecular markers than ever before. Beyond *IDH* mutation*, EGFR* amplification*, TERT* promotor mutation, and +7/-10 chromosome copy-number variations, it is important to find additional molecular changes associated with the prognosis of GBMs that could assist in distinguishing further subgroups for a more individualized treatment (22). In addition to *MGMT* promoter methylation being linked to positive outcomes, alterations of *CDK4/6*, *CIC*, *FGFR3*, *MYB*, *TP53*, and *KMT5B* were strongly related to a worse OS in mol-GBMs, hist-GBMs, or both, which is consistent with the previous studies (20, 48–50). These findings provided a valuable hint for further exploration of these molecular alterations as potential categorization markers or therapeutic targets in GBMs.

Subsequently, we attempted to utilize the TCGA, CGGA, and GEO datasets to confirm our findings. However, since the release of the WHO CNS5 classification was too recent, we could not find any available dataset of DNA sequencing for validation, so we finally used the RNA-sequencing dataset from the CGGA to access the associations between the expression levels of genes and the prognosis of GBMs. The results revealed that the expression levels of *CIC*, *CDK4*, *CDK6*, *TP53*, *FGFR3*, *TOP3A*, and *MYB* might impact the survival of patients with hist-GBMs, thus validating our results from the perspective of the expression of gene alterations.

The current investigation exhibits certain constraints. The screening of IDH-wildtype gliomas during a certain period was incomplete due to unsuccessful DNA extraction from some FFPE samples, which experienced DNA degradation over an extended period. Consequently, a selection bias was inevitable. Furthermore, it is noteworthy that the present study was conducted solely on a cohort of Asian patients, thereby constraining the generalizability of the findings to a broader international population. To enhance the validity of the findings of this study, it is recommended that future collaborations across multiple international centers be pursued, to broaden the scope of sample collection. Additionally, the outcomes were not verified utilizing DNA-sequencing information because of the inadequacy of present public datasets.

## 5 Conclusions

According to the WHO CNS5 classification, GBMs are a new comprehensive entity that encompasses both hist-GBMs (histologically grade 4) and mol-GBMs (histologically grade 2-3). Clinicians should update their diagnostic and therapeutic approaches to this lethal brain malignancy, as the clinical, radiological, molecular, and prognostic characteristics of GBMs have significantly changed after the release of the current WHO classification. Mol-GBMs, a newly identified GBM subtype, lacks sufficient research and clinical awareness, despite having distinct genetic features that may lead to different clinical manifestations. To explore additional targeted therapies, it is necessary to incorporate more molecular features into the current WHO classification.

## Data availability statement

The data presented in the study are deposited in the figshare repository (DOI: 10.6084/m9.figshare.23593203). This data can be found here: https://figshare.com/articles/dataset/Data_for_the_article_Histological_and_molecular_glioblastoma_IDH-wildtype_A_real-world_landscape_using_the_2021_WHO_classification_of_central_nervous_system_tumors_published_in_Frontiers_in_Oncology/23593203.

## Ethics statement

The studies involving human participants were reviewed and approved by Ethics Review Committee of Peking Union Medical College Hospital. The patients/participants provided their written informed consent to participate in this study.

## Author contributions

Conceptualization: XG and WC. Methodology: XG, LG, YL, and ZZ. Formal analysis and investigation: XG, LG, YL, ZZ, WC, YNW, YKW, HX, YS, DL, YX, TY, JL, JW, TL, HW, XZ, QL, SJ, TQ, SG, HL, YG, CW, KZ, YW, and WM. Writing—original draft preparation: XG, LG, YL, and ZZ. Writing—review and editing: YW and WM. Supervision: YW and WM. All authors provided meaningful contributions to the manuscript. All authors read and approved the final manuscript.
