# Supplementary material for: Histological and molecular glioblastoma, IDH-wildtype: a real-world landscape using the 2021 WHO classification of central nervous system tumors
Source: Front Oncol. 2023 Jul 6;13:1200815. doi: 10.3389/fonc.2023.1200815 (PMC10358772; doi:10.3389/fonc.2023.1200815)
Supplement: Supplementary file 1 [file Image_1.pdf]

Supplementary Figure 1. Kaplan–Meier curves showing no significant effects of certain genetic and immunohistochemical alterations on the overall survival of patients with glioblastomas (1-39). Since the numbers of patients divided into two groups (e.g. FGFR1 Wildtype/FGFR1 Alteration) using different parameters varied, we only enrolled the parameters using which the number of patients in either group was above 3. Parameters used in this section were shown as follows.

(1) Ki-67 1
(2) NeuN 2
(3) Syn 3
(4) Oligo2 4
(5) ATRX 5
(6) S-100 6
(7) GAFP 7
(8) TERT promotor mutation 8
(9) EGFR Amplification 9
(10) CDKN2A/B homozygous deletion 10
(11) Chromosome- 7 gain/10 loss 11
(12) TP53 12
(13) BRAF 13
(14) CDK6 14
(15) FGFR1 15
(16) FGFR2 16
(17) FGFR4 17
(18) KIT 18
(19) KRAS 19
(20) MET 20
(21) MYB 21
(22) MYBL1 22
(23) MYC 23
(24) MYCN 24
(25) NF1 25
(26) NOTCH1 26
(27) NTRK2 27
(28) NTRK3 28
(29) PDGFRA 29
(30) PEG3 30
(31) PIK3CA 31
(32) PPM1D 32
(33) PTEN 33
(34) PTPN11 34
(35) RB1 35
(36) TOP3A 36
(37) PIK3R1 37
(38) TSC2 38
(39) chr17 39

(1)

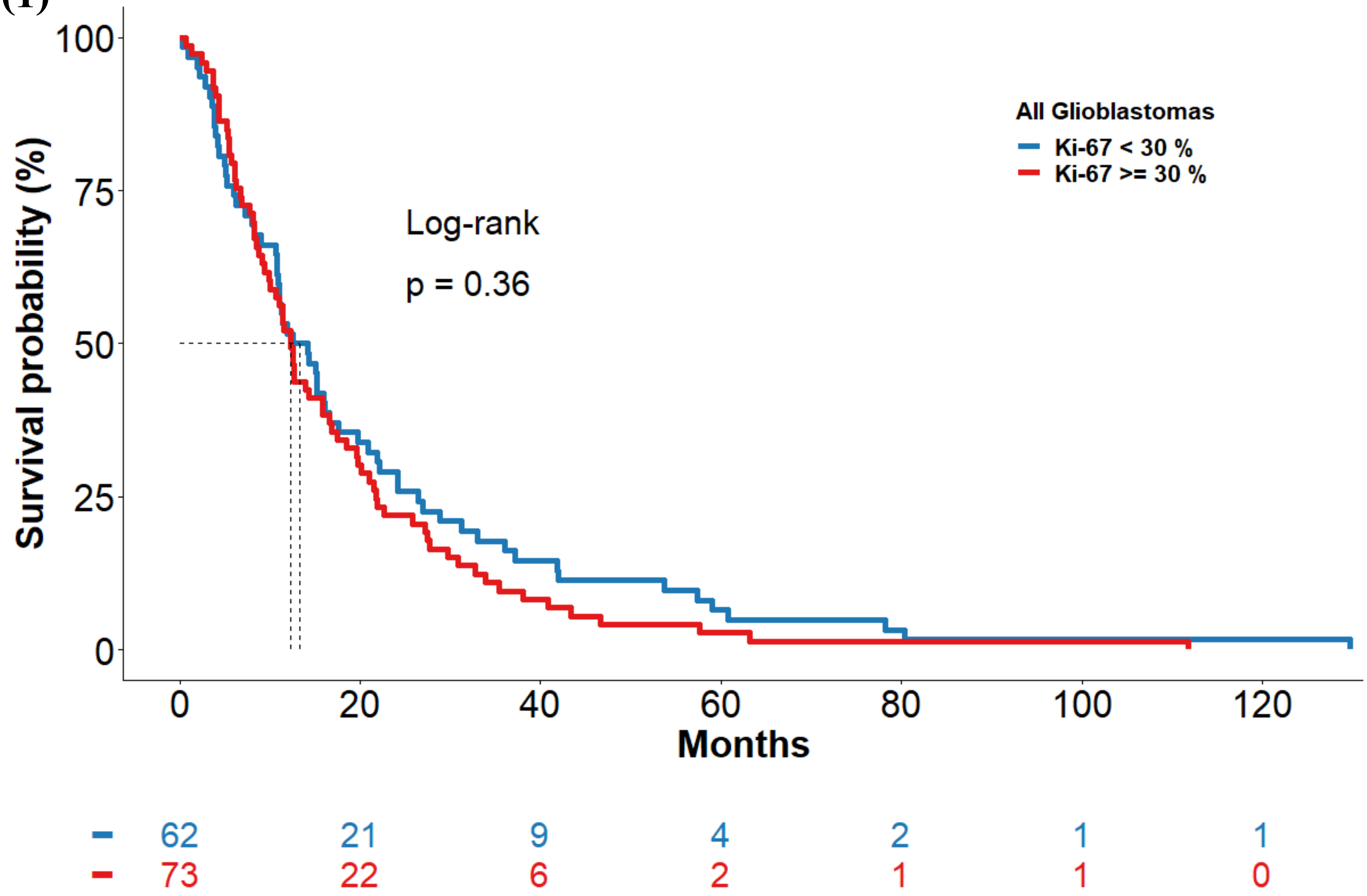

Survival probability (%)
100
75
50
25
0
Log-rank
p = 0.36
All Glioblastomas
Ki-67 < 30 %
Ki-67 >= 30 %
0
20
40
60
80
100
120
Months
62 21 9 4 2 1 1
73 22 6 2 1 1 0

(2)

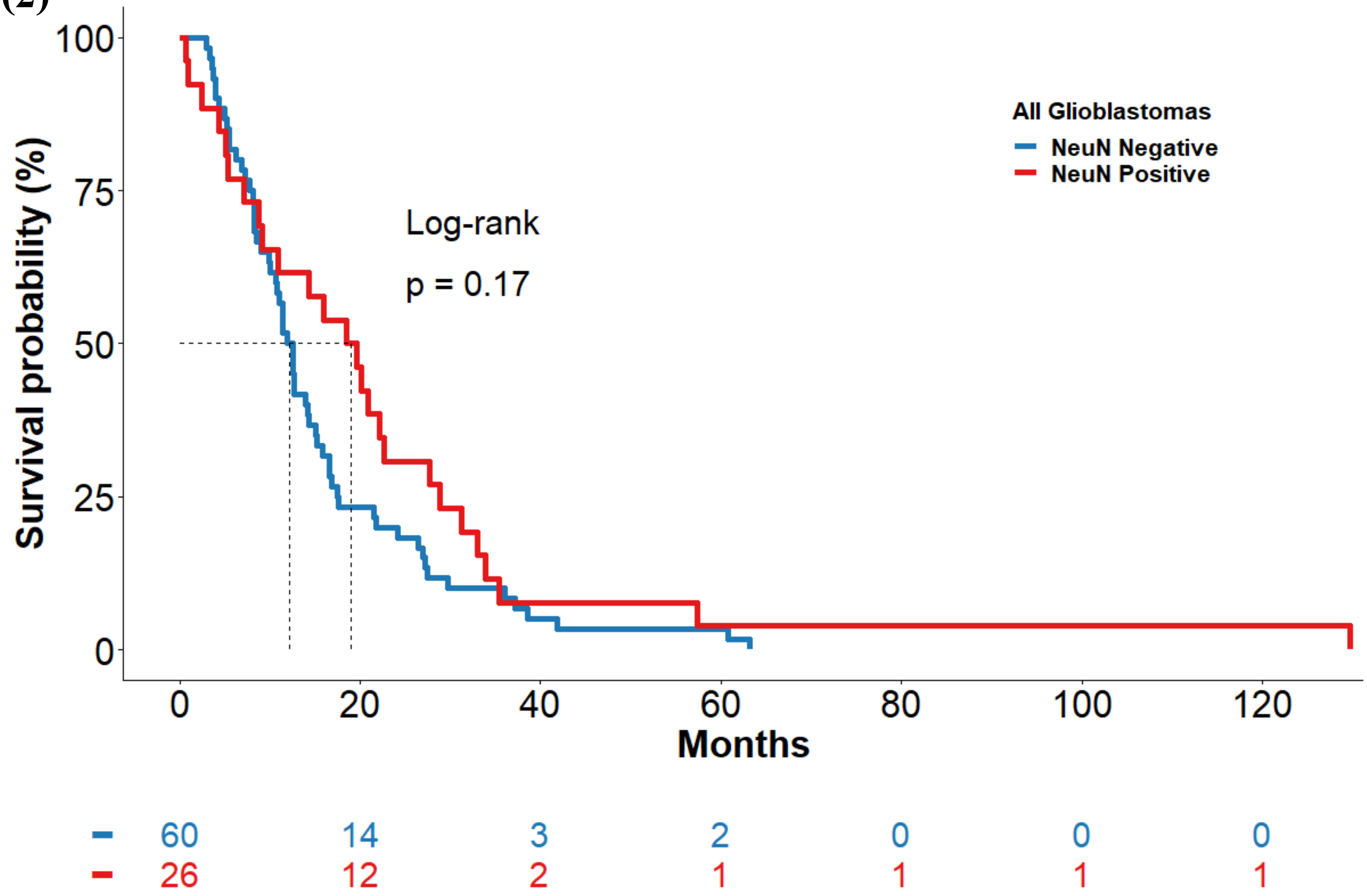
Survival probability (%)
100
75
50
25
0
Log-rank
p = 0.17
All Glioblastomas
NeuN Negative
NeuN Positive
0
20
40
60
80
100
120
Months
60
14
3
2
0
0
0
26
12
2
1
1
1
1

(3)

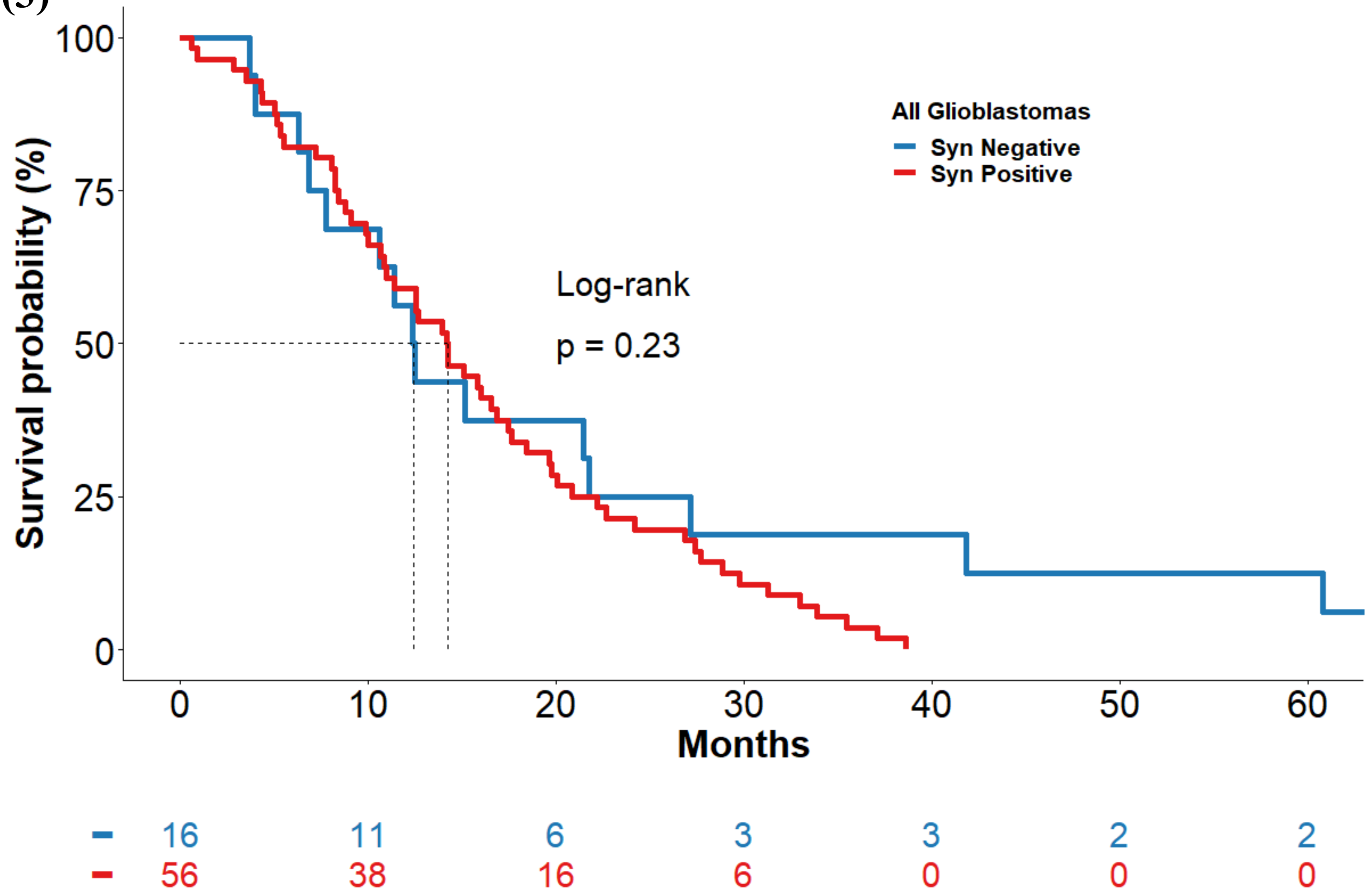

Survival probability (%)
100
75
50
25
0
Log-rank
p = 0.23
All Glioblastomas
Syn Negative
Syn Positive
0
10
20
30
40
50
60
Months
16 11 6 3 3 2 2
56 38 16 6 0 0 0

(4)

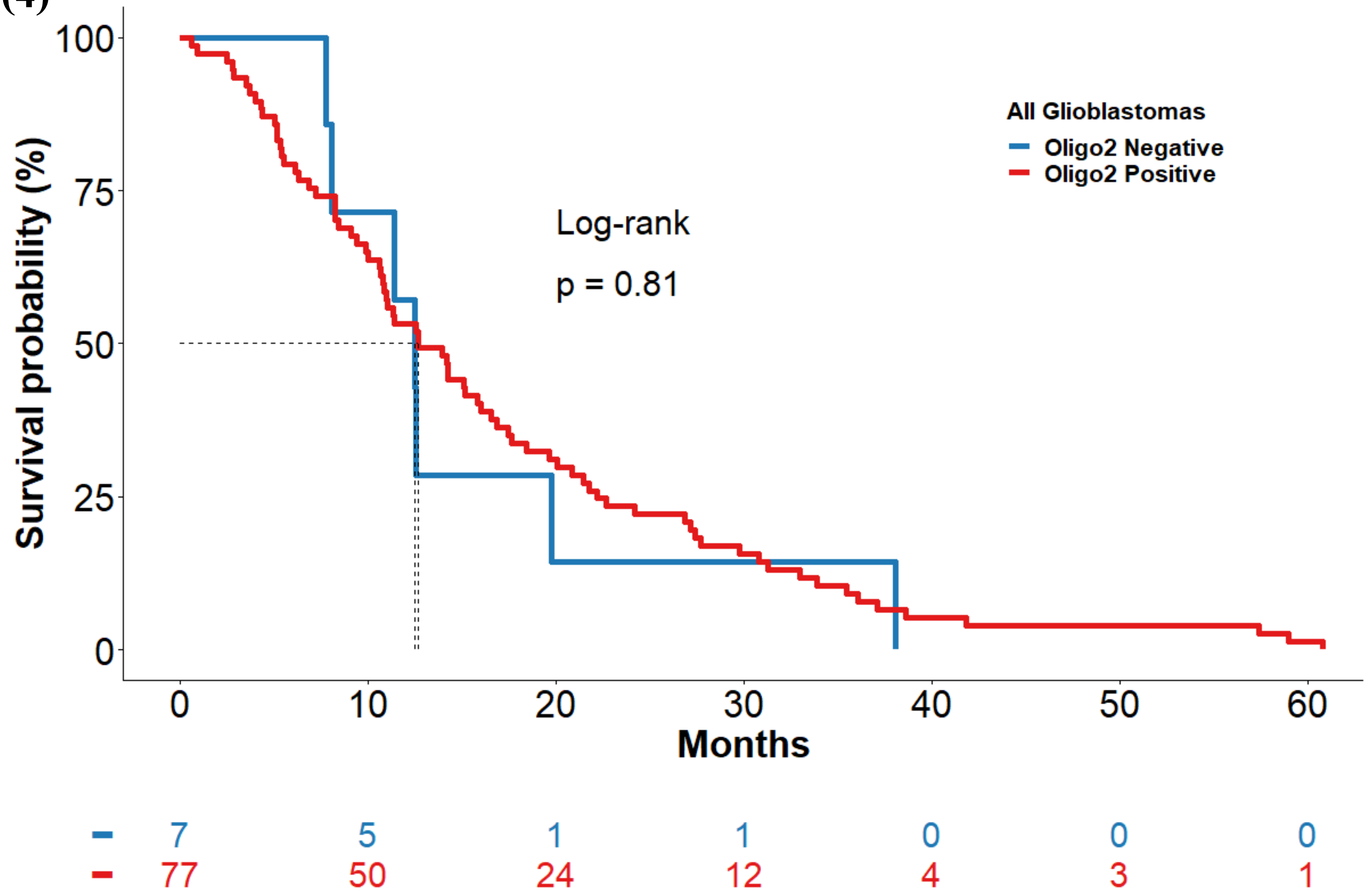
Survival probability (%)
100
75
50
25
0
Log-rank
p = 0.81
All Glioblastomas
Oligo2 Negative
Oligo2 Positive
0
10
20
30
40
50
60
Months
7 5 1 1 0 0 0
77 50 24 12 4 3 1

(5)

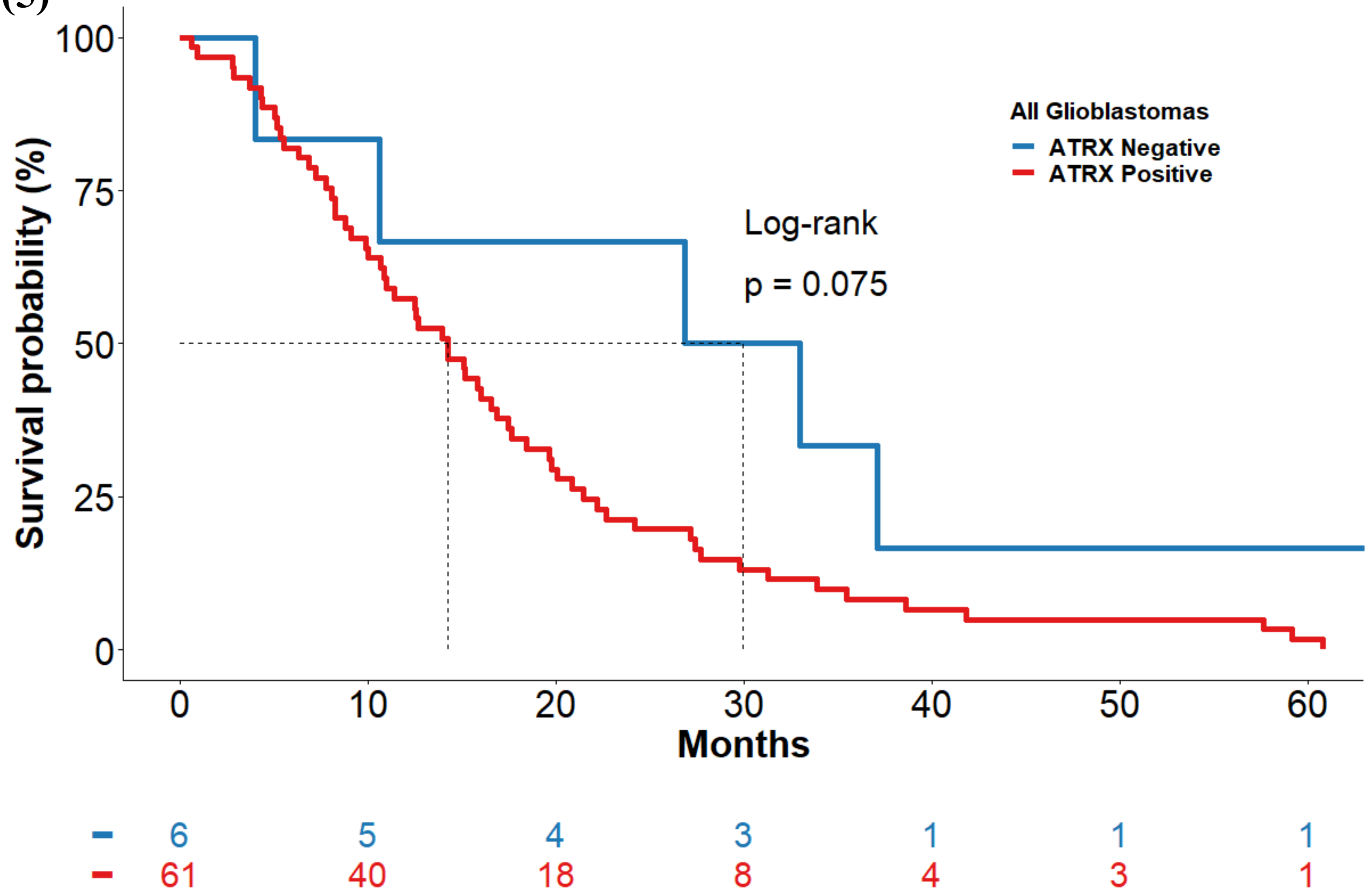

100
75
50
25
0
Survival probability (%)
All Glioblastomas
ATRX Negative
ATRX Positive
Log-rank
p = 0.075
0
10
20
30
40
50
60
Months
6 5 4 3 1 1 1
61 40 18 8 4 3 1

(6)

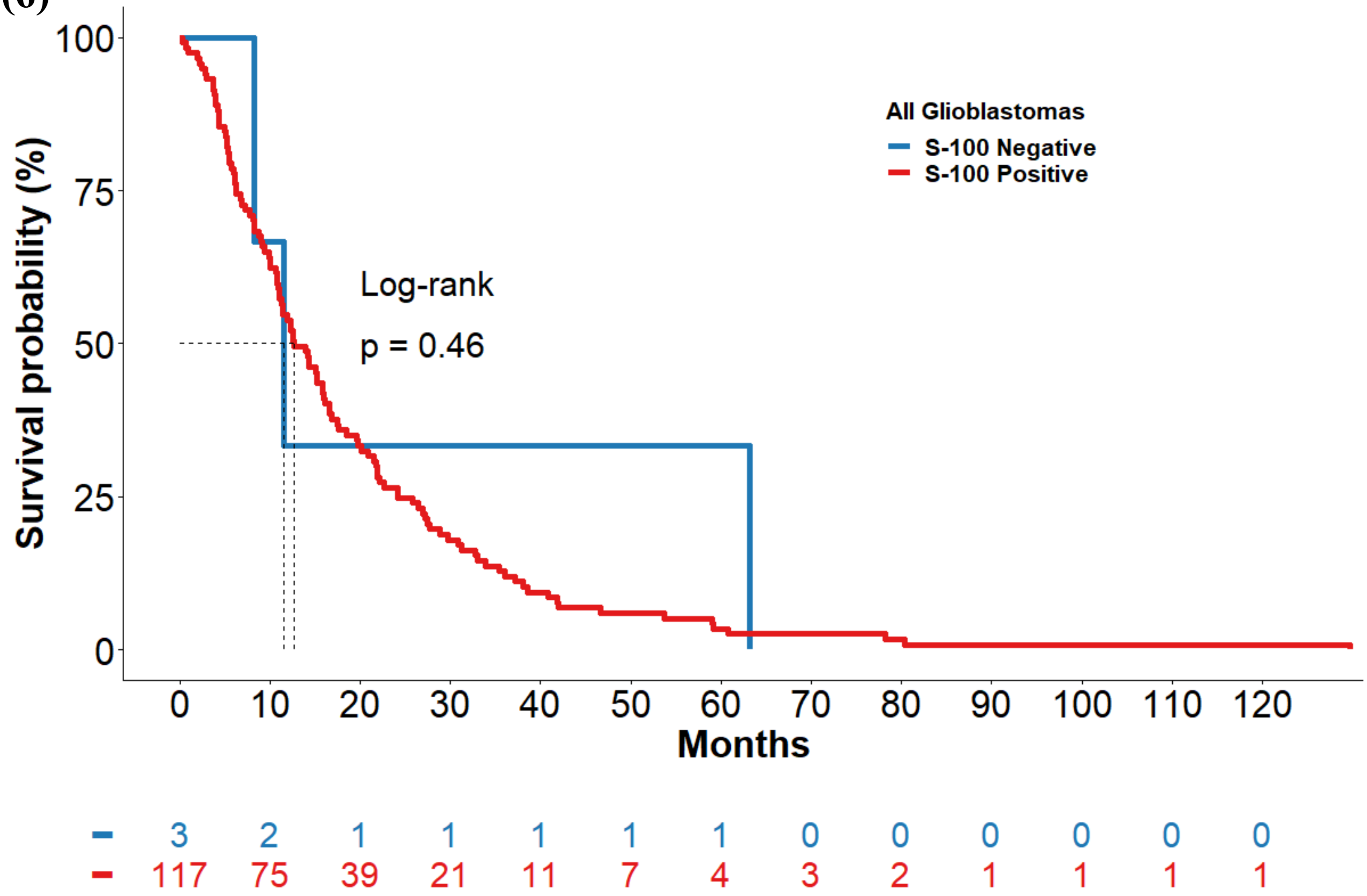

Survival probability (%)
100
75
50
25
0
Log-rank
p = 0.46
All Glioblastomas
S-100 Negative
S-100 Positive
0
10
20
30
40
50
60
70
80
90
100
110
120
Months
3 2 1 1 1 1 1 0 0 0 0 0 0
117 75 39 21 11 7 4 3 2 1 1 1 1

(7)

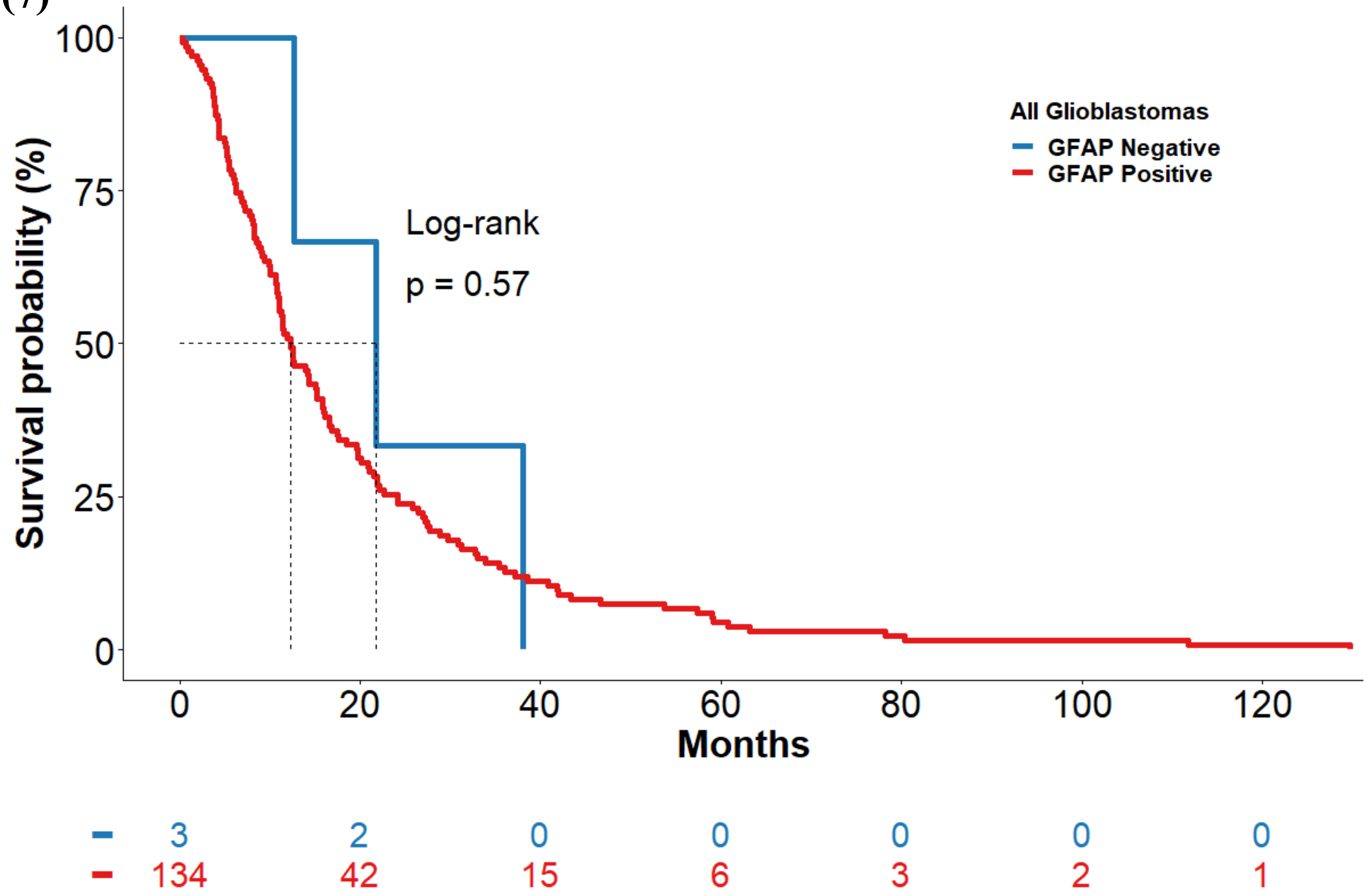
Survival probability (%)
100
75
50
25
0
Log-rank
p = 0.57
All Glioblastomas
GFAP Negative
GFAP Positive
0
20
40
60
80
100
120
Months
3 2 0 0 0 0 0
134 42 15 6 3 2 1

(8)

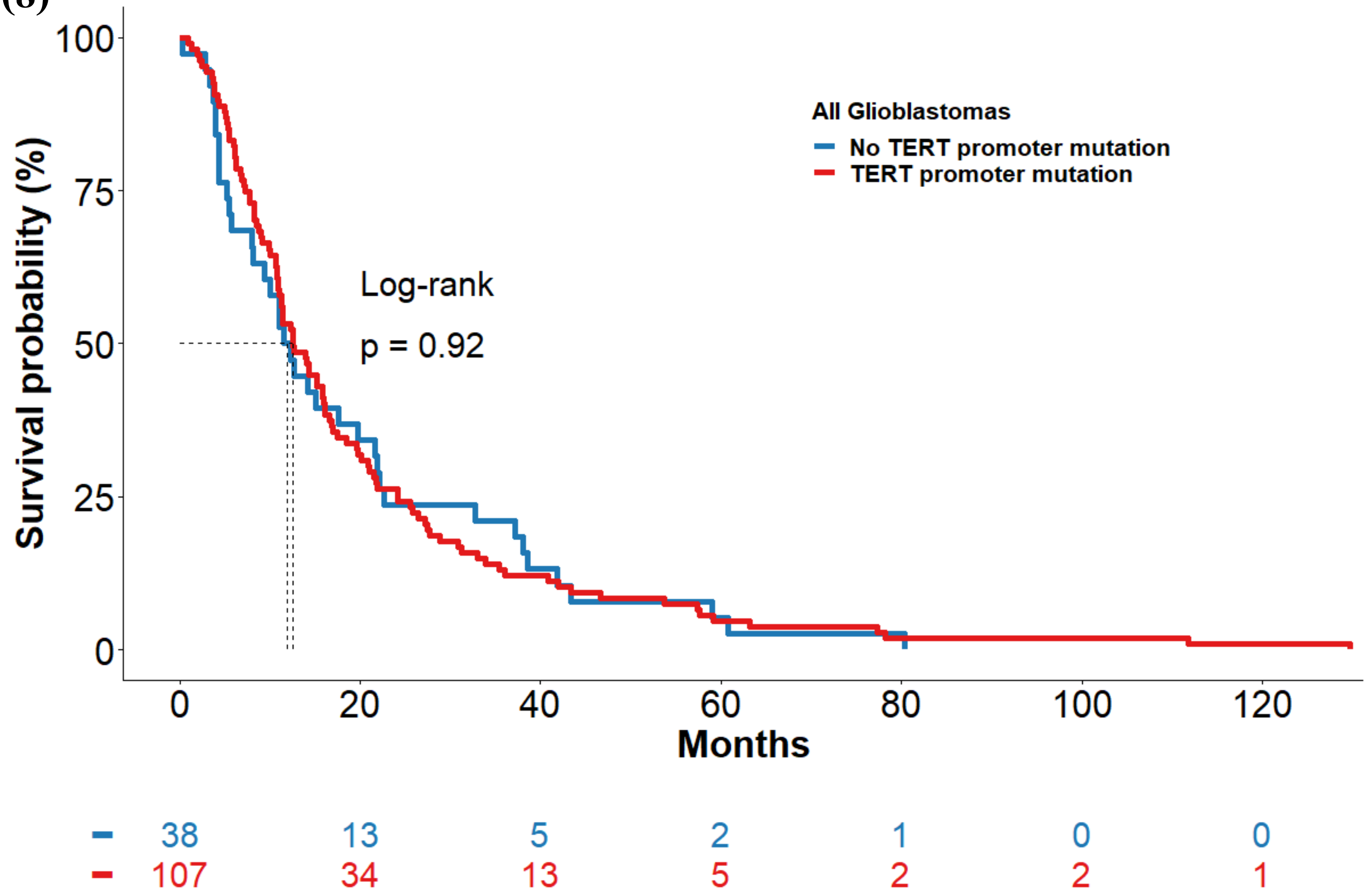

Survival probability (%)
100
75
50
25
0
Log-rank
p = 0.92
All Glioblastomas
No TERT promoter mutation
TERT promoter mutation
0
20
40
60
80
100
120
Months
38 13 5 2 1 0 0
107 34 13 5 2 2 1

(9)

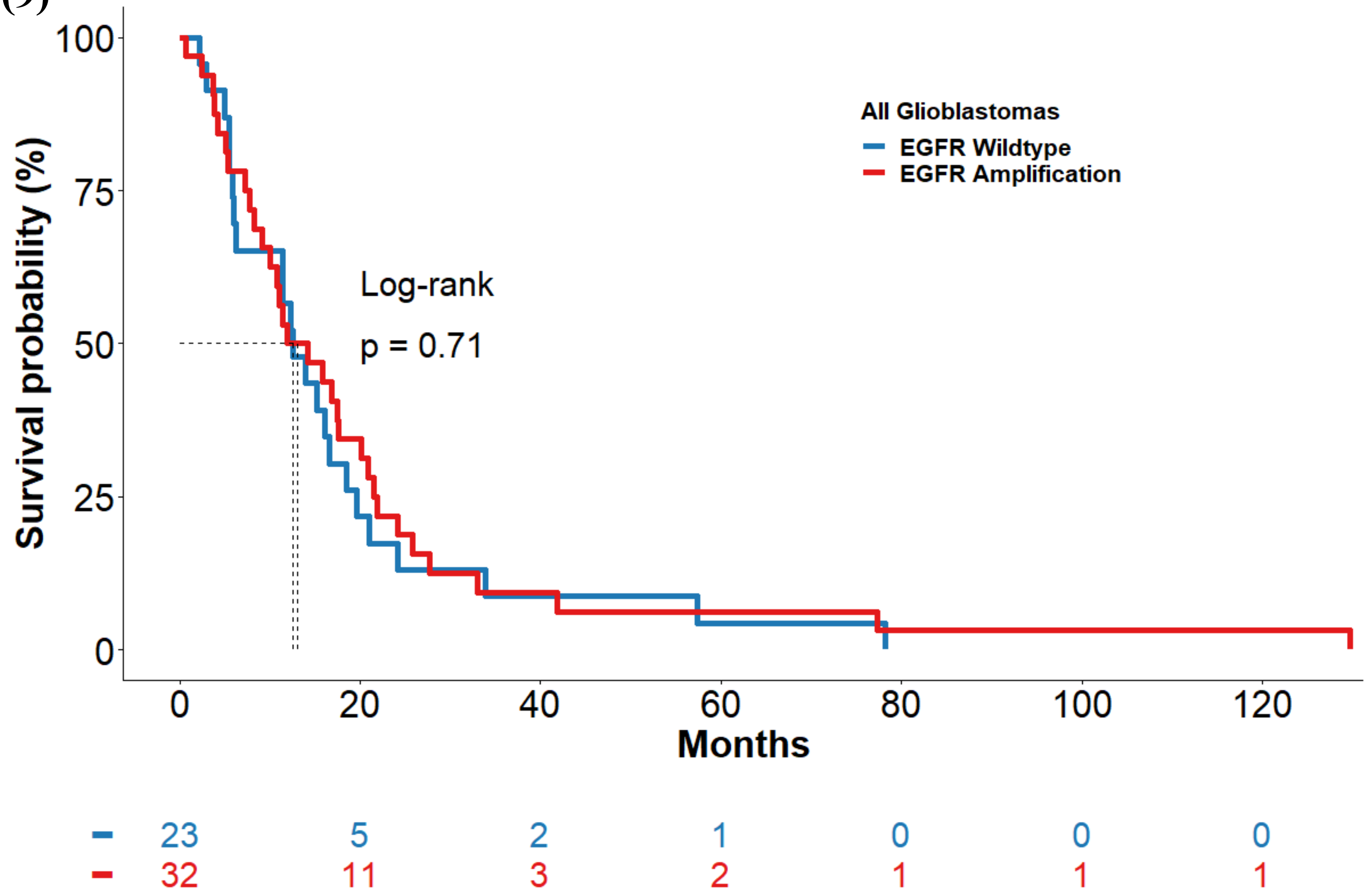

All Glioblastomas
EGFR Wildtype
EGFR Amplification
Log-rank
p = 0.71
Survival probability (%)
100
75
50
25
0
0
20
40
60
80
100
120
Months
23 5 2 1 0 0 0
32 11 3 2 1 1 1

(10)

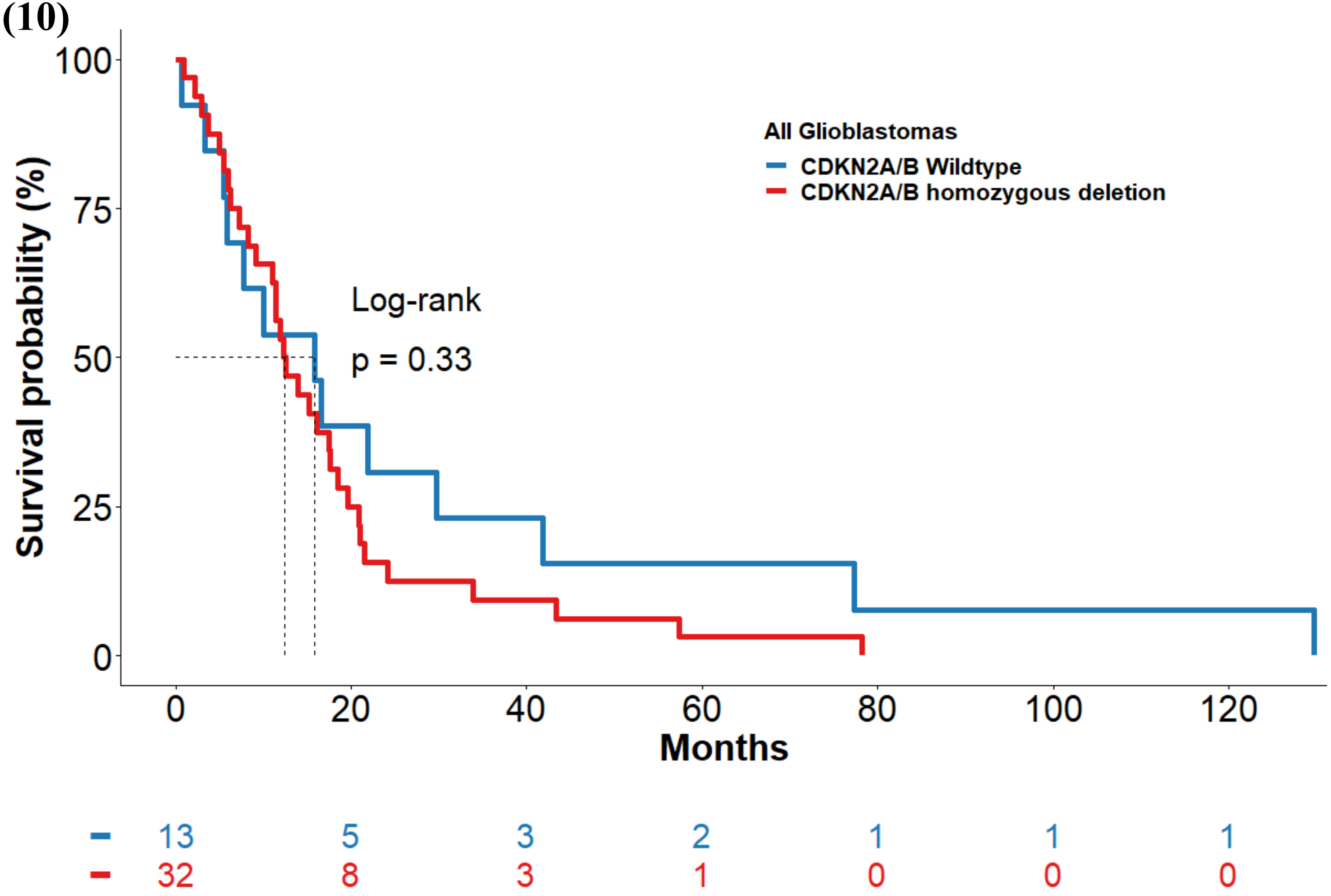

Survival probability (%)
100
75
50
25
0
0
20
40
60
80
100
120
Months
All Glioblastomas
CDKN2A/B Wildtype
CDKN2A/B homozygous deletion
Log-rank
p = 0.33
13 5 3 2 1 1 1
32 8 3 1 0 0 0

(11)

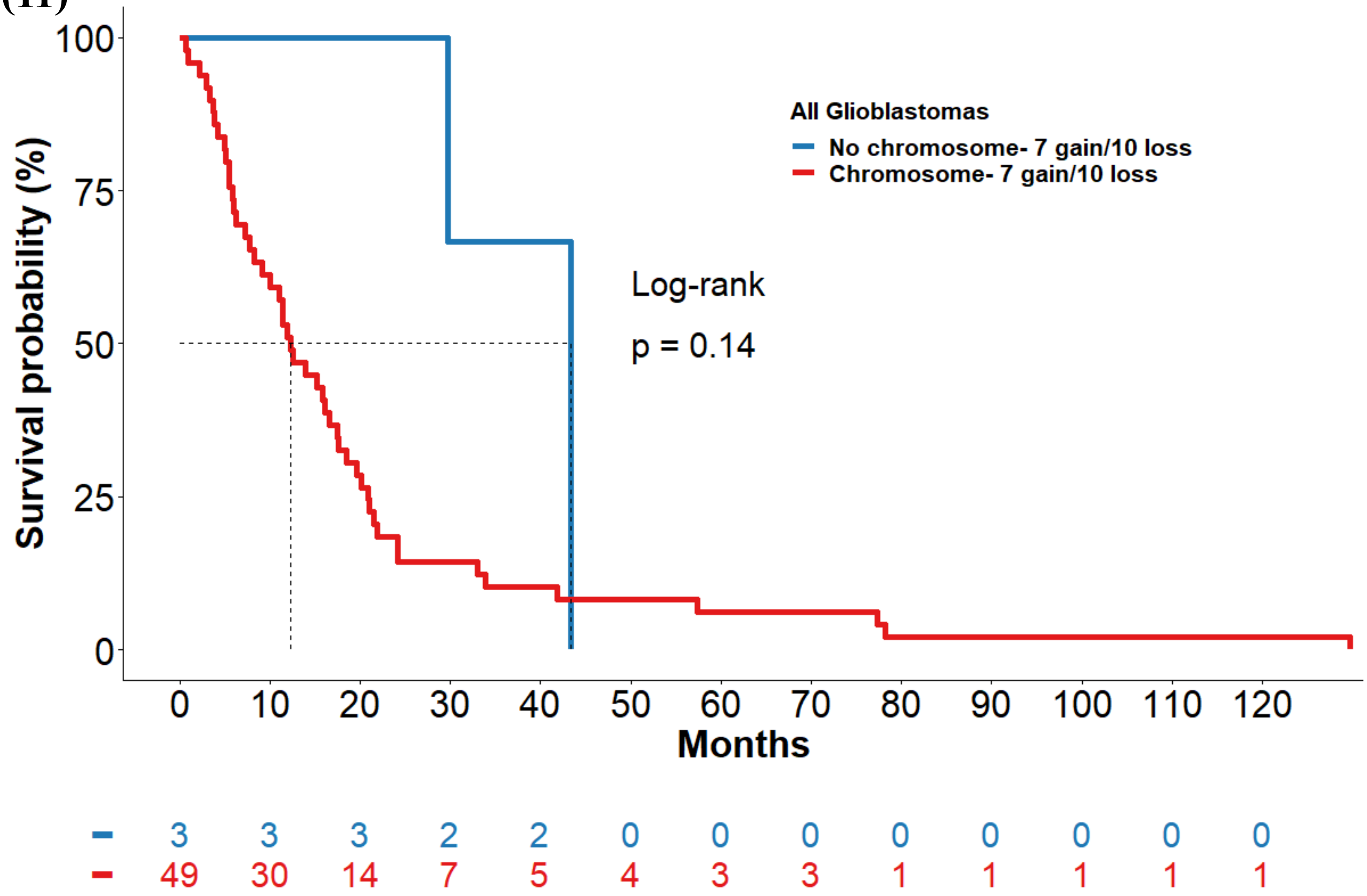
Survival probability (%)
100
75
50
25
0
All Glioblastomas
No chromosome- 7 gain/10 loss
Chromosome- 7 gain/10 loss
Log-rank
p = 0.14
0 10 20 30 40 50 60 70 80 90 100 110 120
Months
3 3 3 2 2 0 0 0 0 0 0 0 0
49 30 14 7 5 4 3 3 1 1 1 1 1

(12)

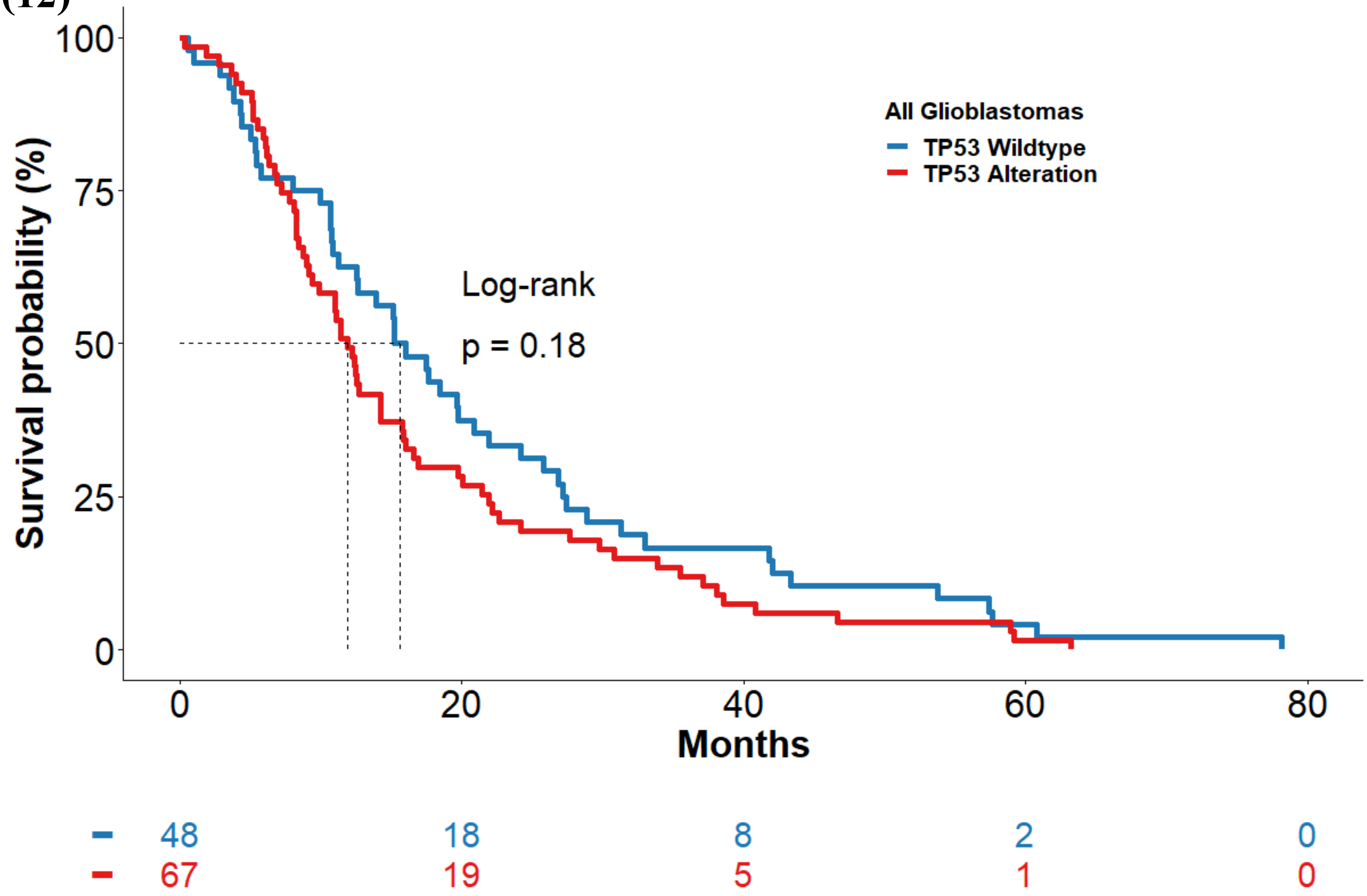

All Glioblastomas
TP53 Wildtype
TP53 Alteration
Survival probability (%)
100
75
50
25
0
Log-rank
p = 0.18
0
20
40
60
80
Months
48
18
8
2
0
67
19
5
1
0

(13)

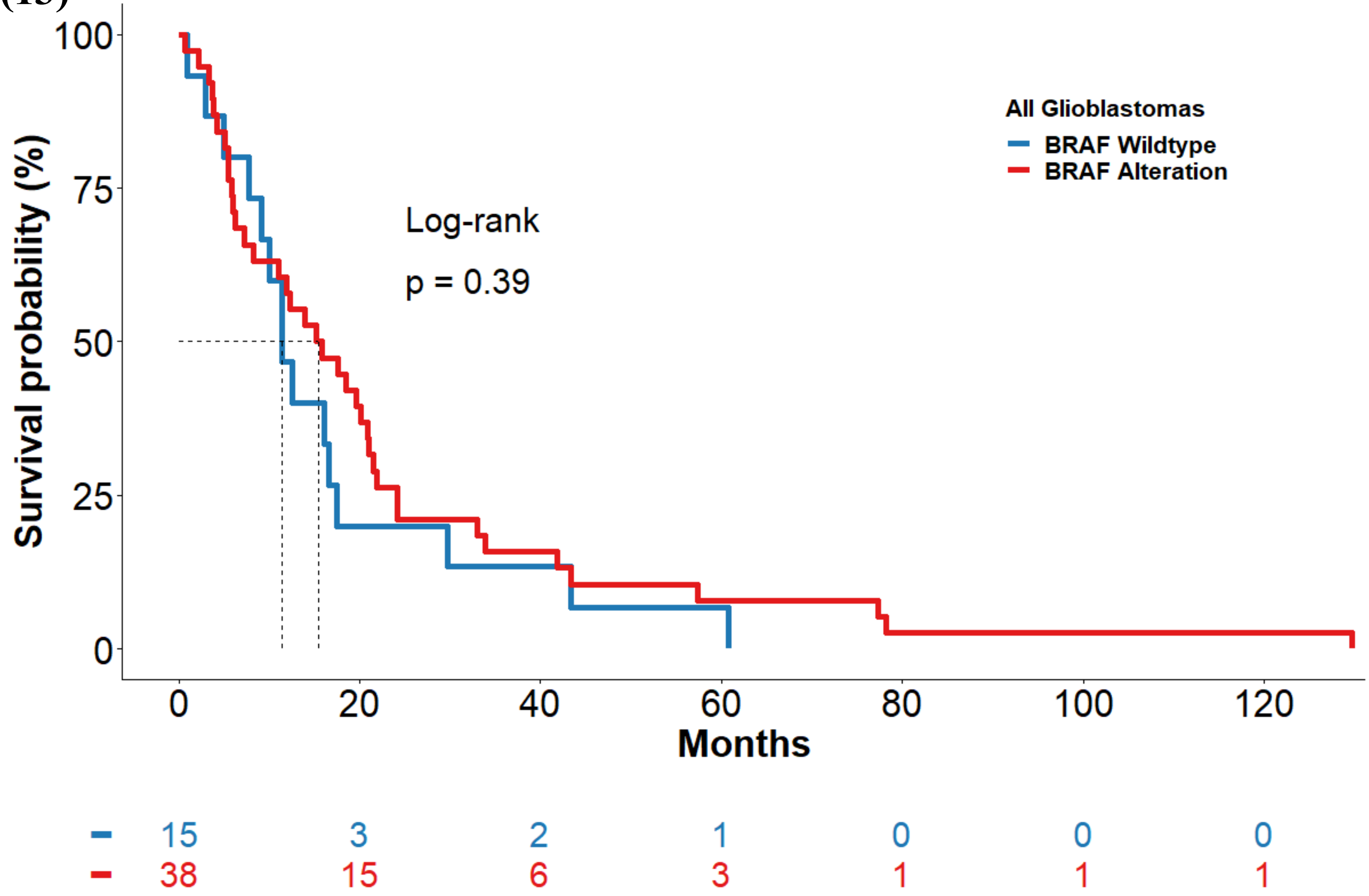

Survival probability (%)
100
75
50
25
0
Log-rank
p = 0.39
All Glioblastomas
BRAF Wildtype
BRAF Alteration
0
20
40
60
80
100
120
Months
15 3 2 1 0 0 0
38 15 6 3 1 1 1

(14)

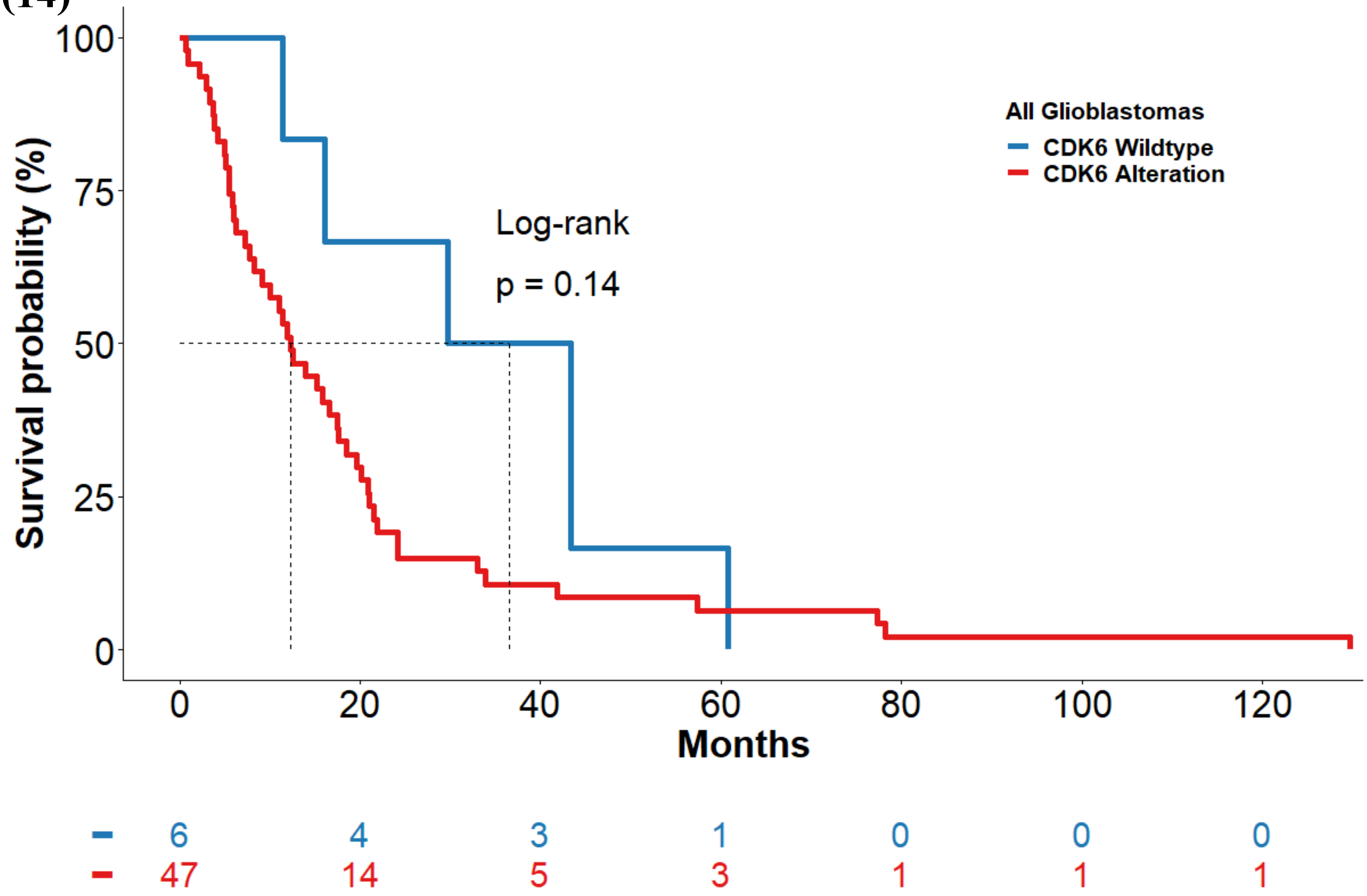

All Glioblastomas
CDK6 Wildtype
CDK6 Alteration
Survival probability (%)
100
75
50
25
0
Log-rank
p = 0.14
0
20
40
60
80
100
120
Months
6
4
3
1
0
0
0
47
14
5
3
1
1
1

(15)

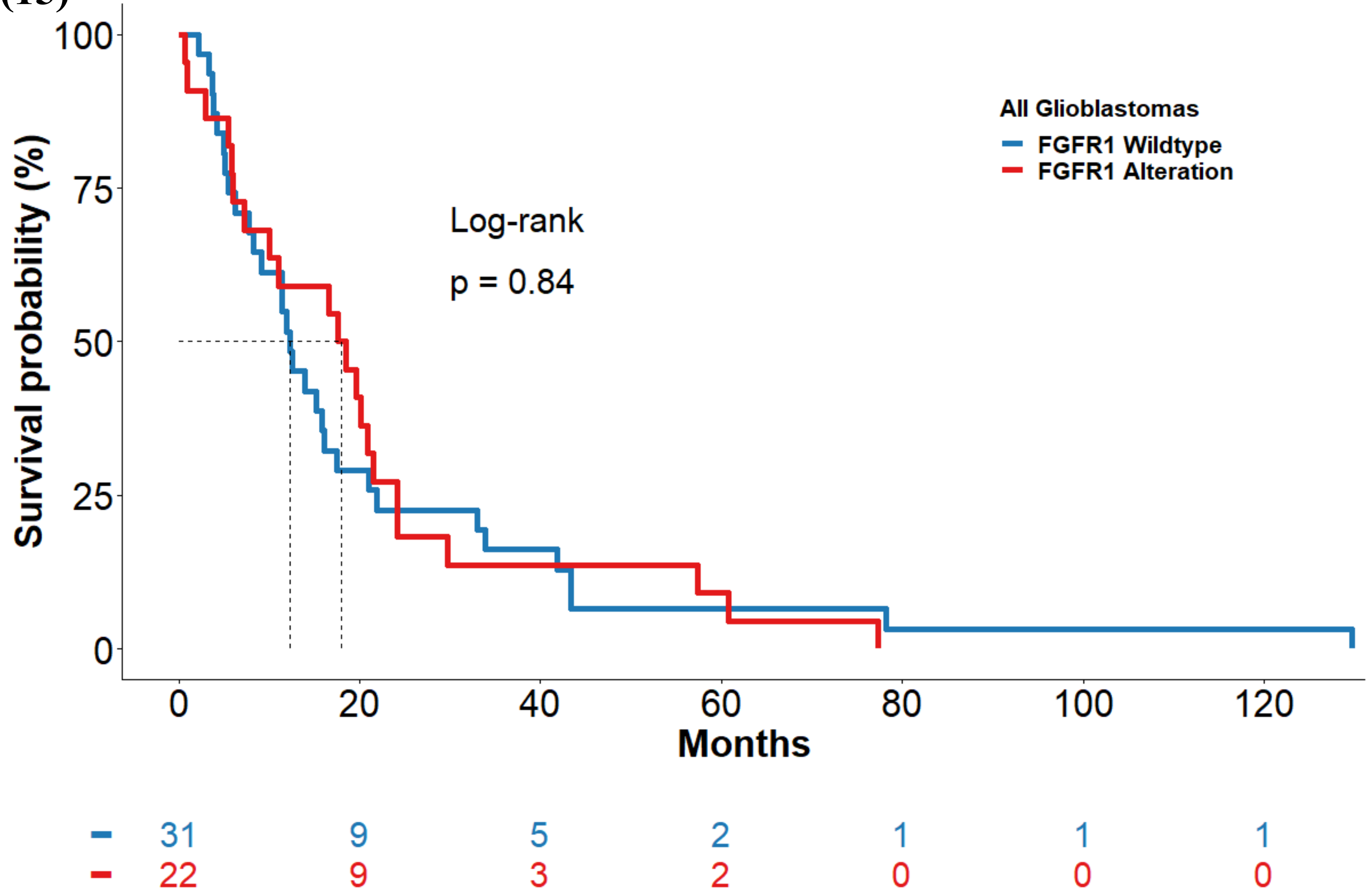

Survival probability (%)
100
75
50
25
0
Log-rank
p = 0.84
All Glioblastomas
FGFR1 Wildtype
FGFR1 Alteration
0
20
40
60
80
100
120
Months
31
9
5
2
1
1
1
22
9
3
2
0
0
0

(16)

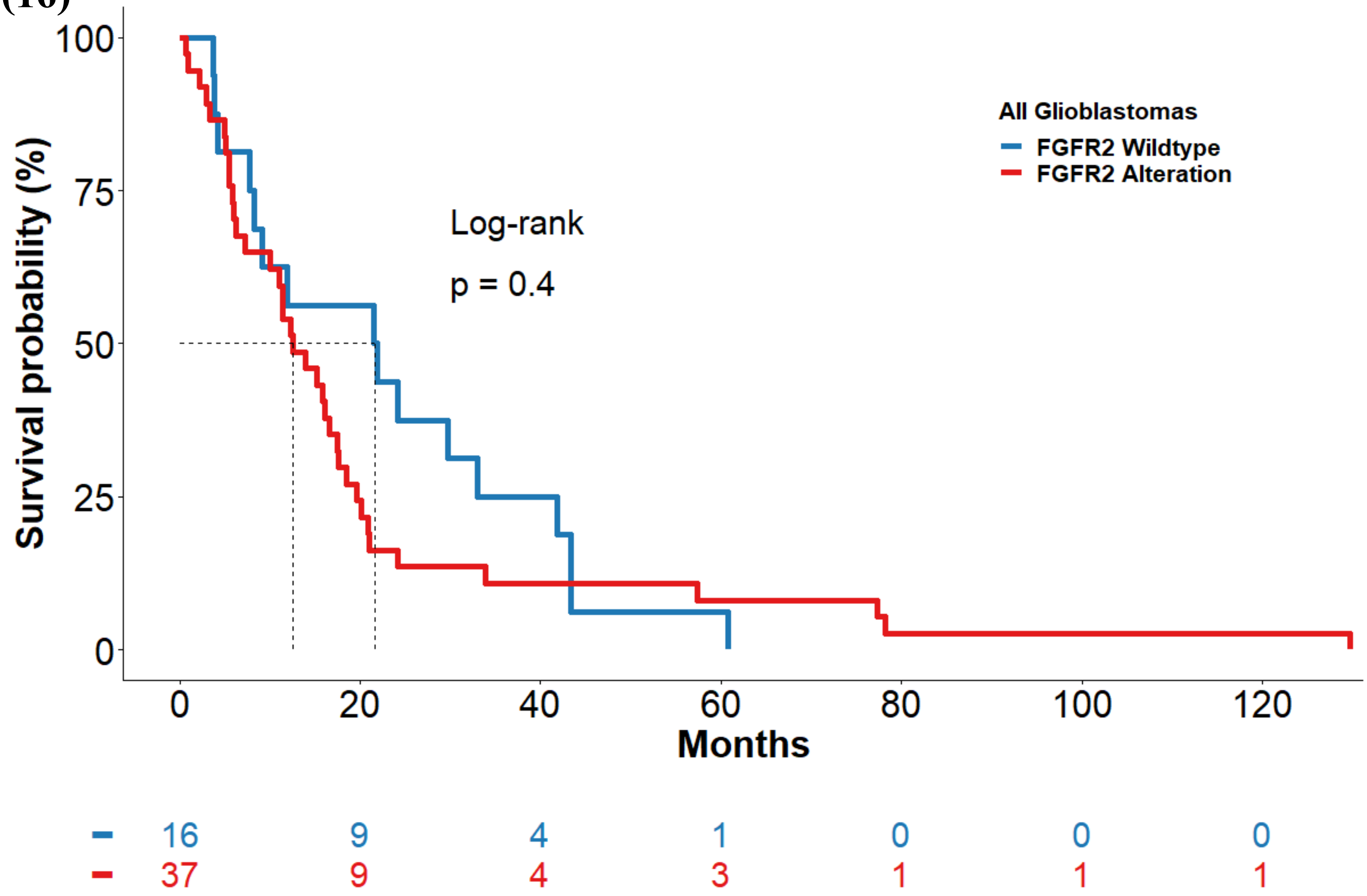

Survival probability (%)
100
75
50
25
0
Log-rank
p = 0.4
All Glioblastomas
FGFR2 Wildtype
FGFR2 Alteration
0
20
40
60
80
100
120
Months
16
9
4
1
0
0
0
37
9
4
3
1
1
1

(17)

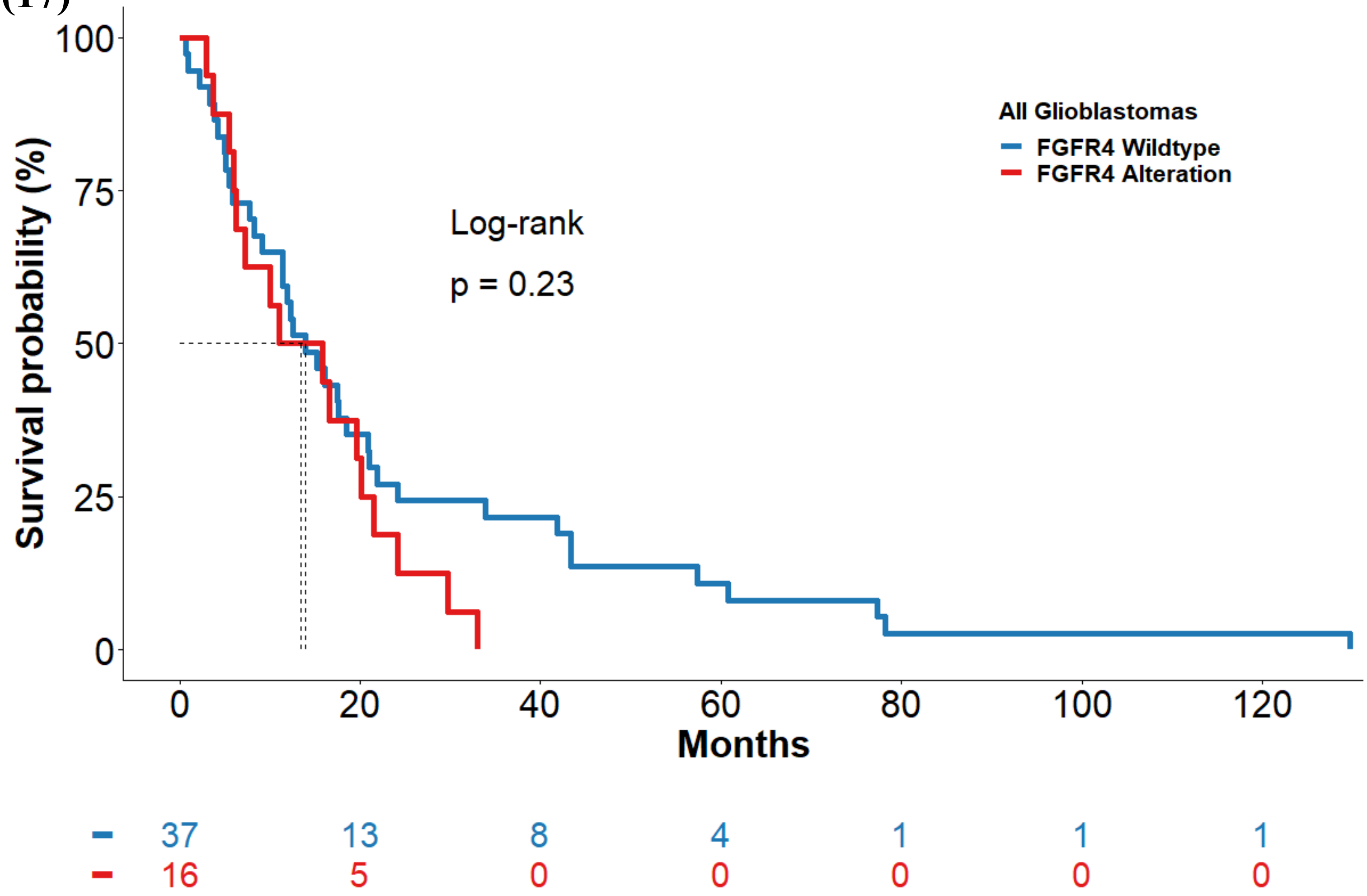
Survival probability (%)
100
75
50
25
0
Log-rank
p = 0.23
All Glioblastomas
FGFR4 Wildtype
FGFR4 Alteration
0
20
40
60
80
100
120
Months
37 13 8 4 1 1 1
16 5 0 0 0 0 0

(18)

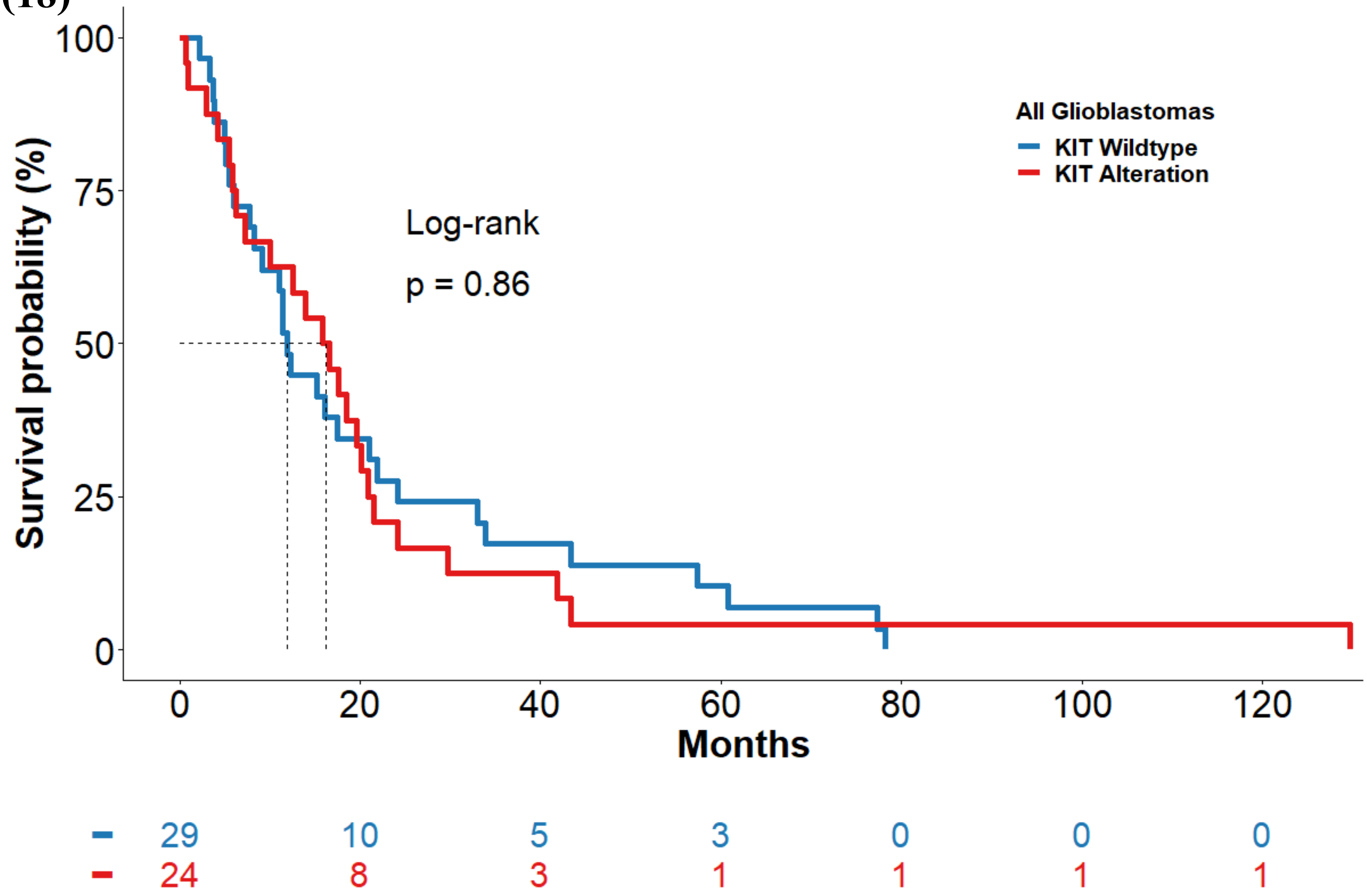

Survival probability (%)
100
75
50
25
0
Log-rank
p = 0.86
All Glioblastomas
KIT Wildtype
KIT Alteration
0
20
40
60
80
100
120
Months
29
10
5
3
0
0
0
24
8
3
1
1
1
1

(19)

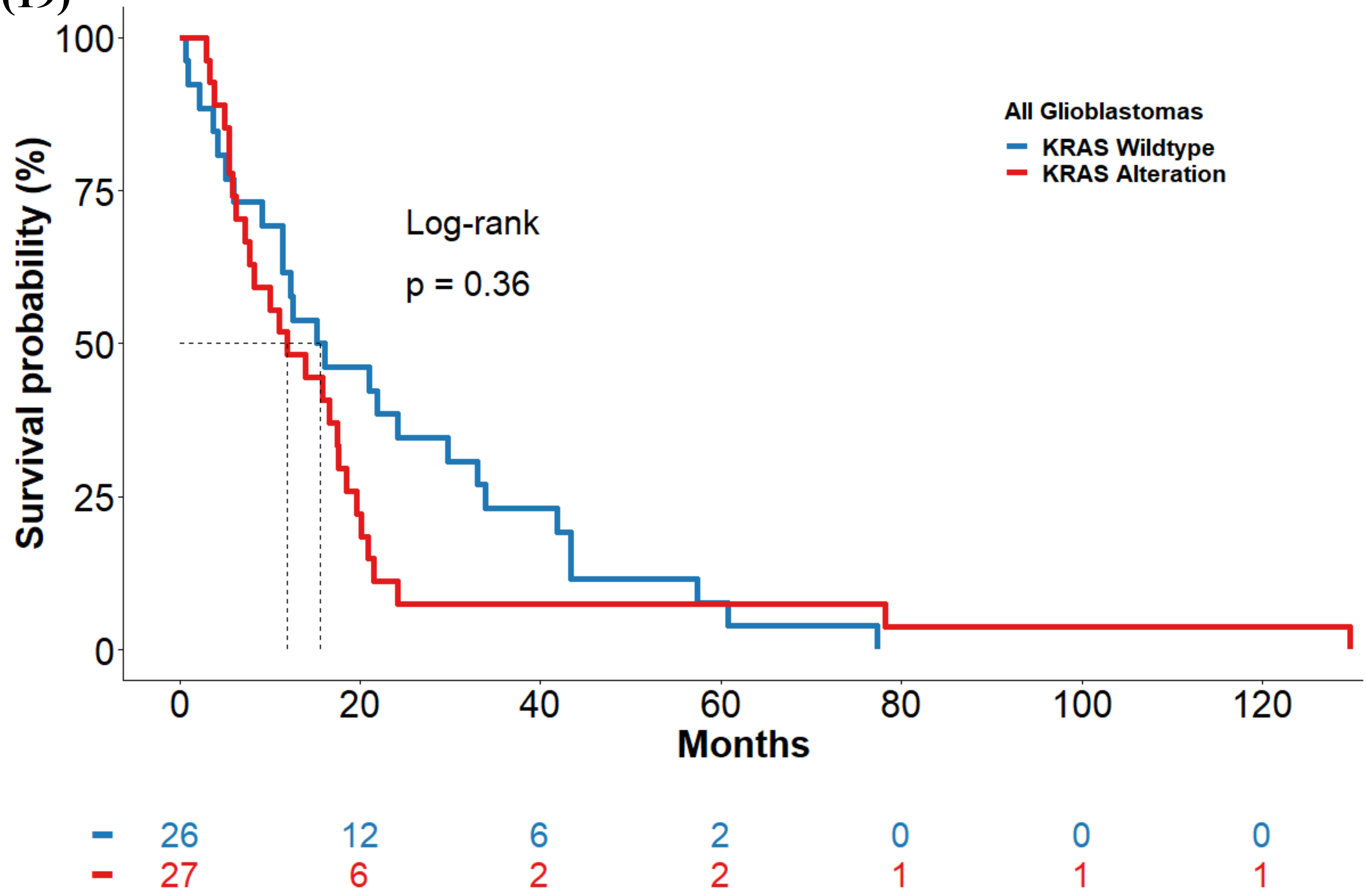

100
75
50
25
0
Survival probability (%)
Log-rank
p = 0.36
All Glioblastomas
KRAS Wildtype
KRAS Alteration
0
20
40
60
80
100
120
Months
26 12 6 2 0 0 0
27 6 2 2 1 1 1

(20)

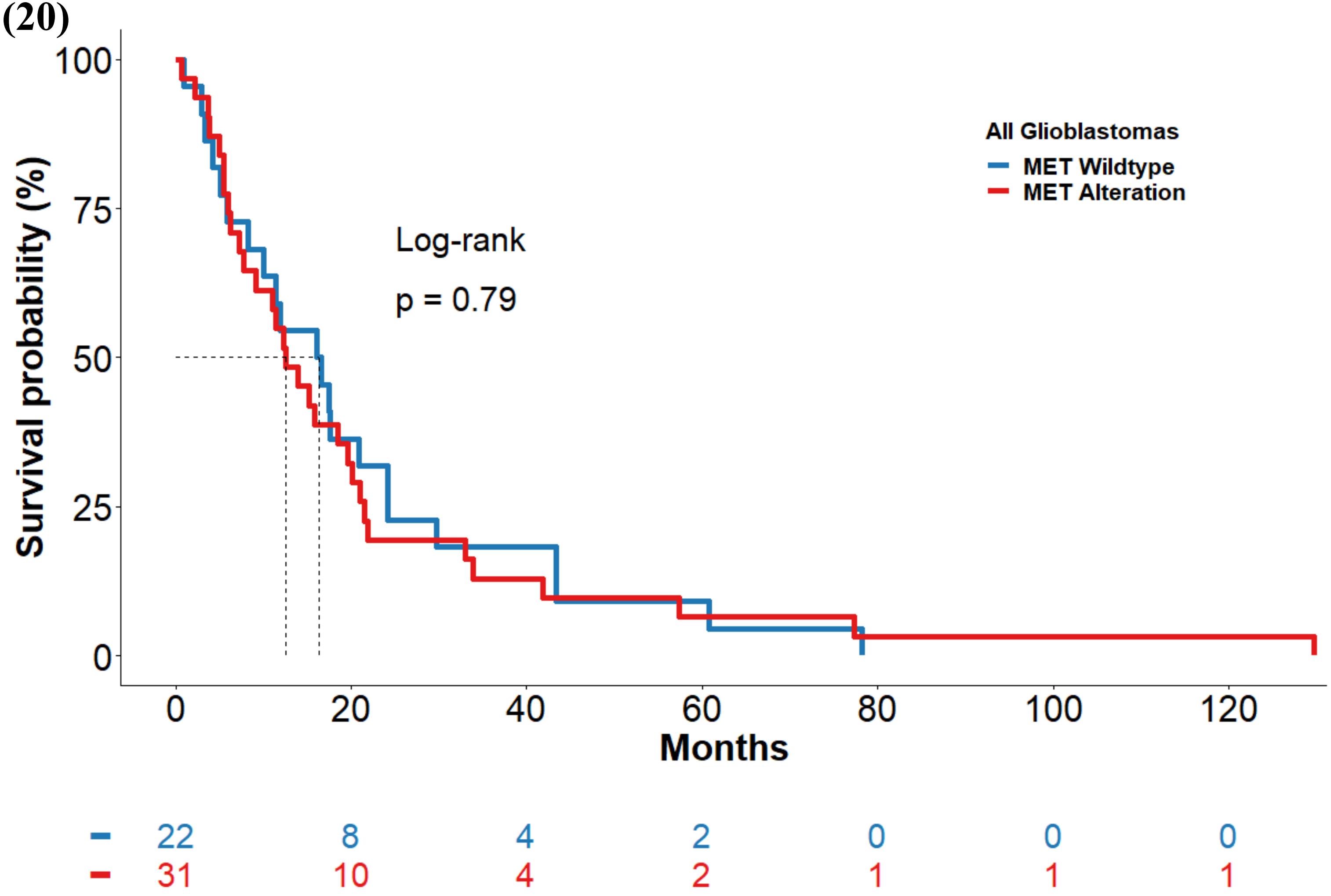

All Glioblastomas
MET Wildtype
MET Alteration
Log-rank
p = 0.79
Survival probability (%)
100
75
50
25
0
0
20
40
60
80
100
120
Months
22 8 4 2 0 0 0
31 10 4 2 1 1 1

(21)

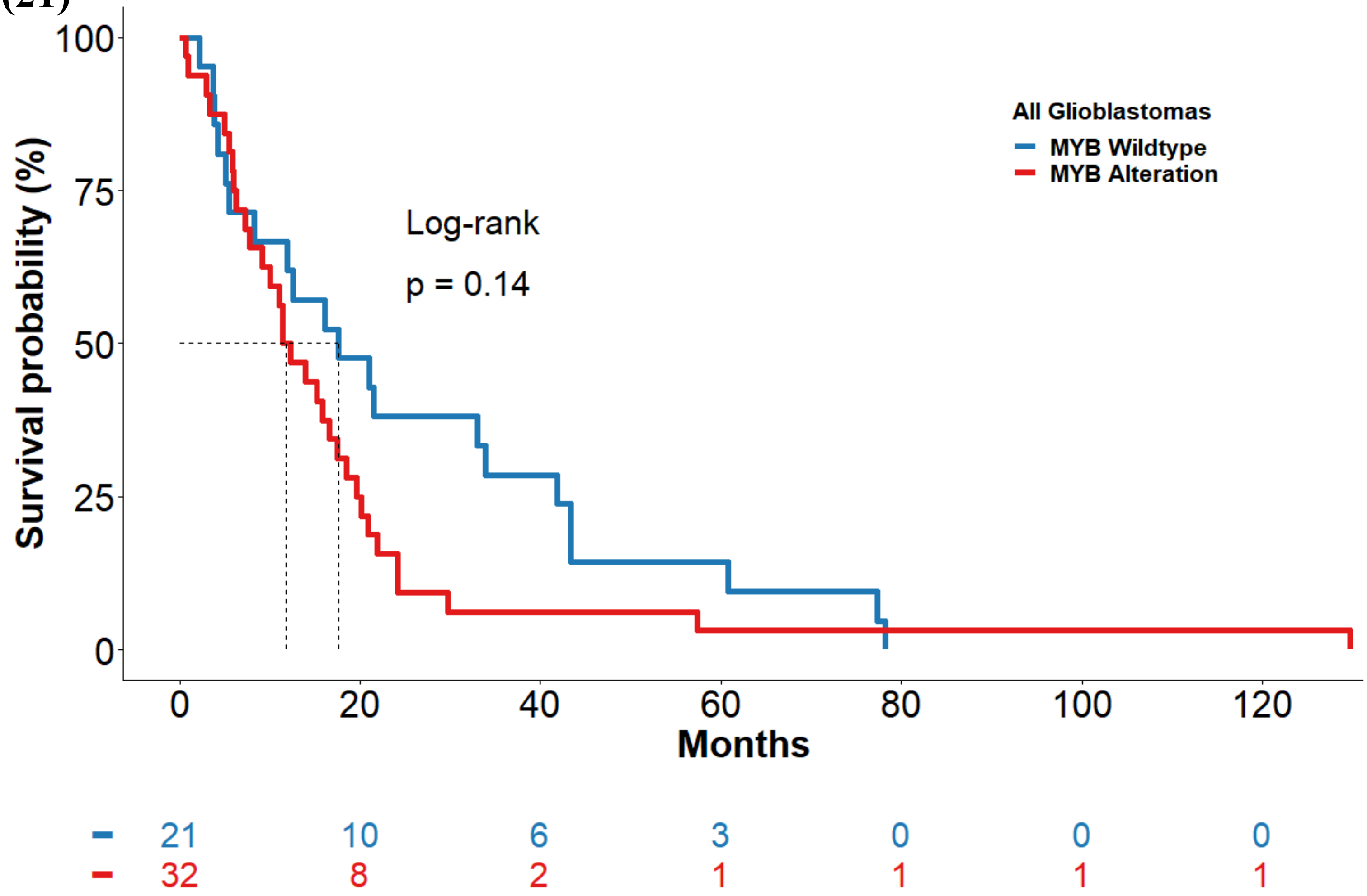

Survival probability (%)
100
75
50
25
0
Log-rank
p = 0.14
All Glioblastomas
MYB Wildtype
MYB Alteration
0
20
40
60
80
100
120
Months
21
10
6
3
0
0
0
32
8
2
1
1
1
1

(22)

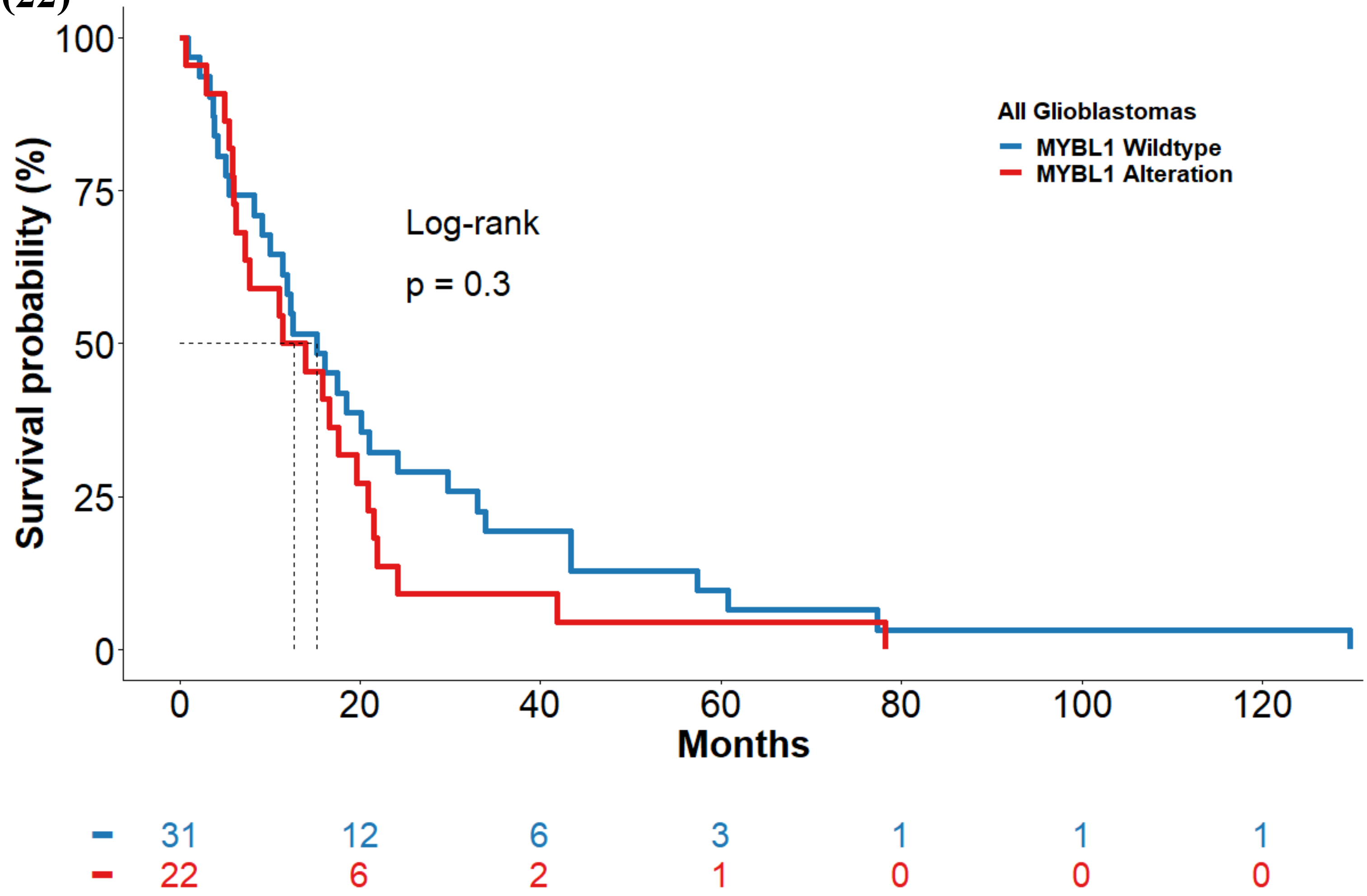

100
75
50
25
0
Survival probability (%)
Log-rank
p = 0.3
All Glioblastomas
MYBL1 Wildtype
MYBL1 Alteration
0
20
40
60
80
100
120
Months
31 12 6 3 1 1 1
22 6 2 1 0 0 0

(23)

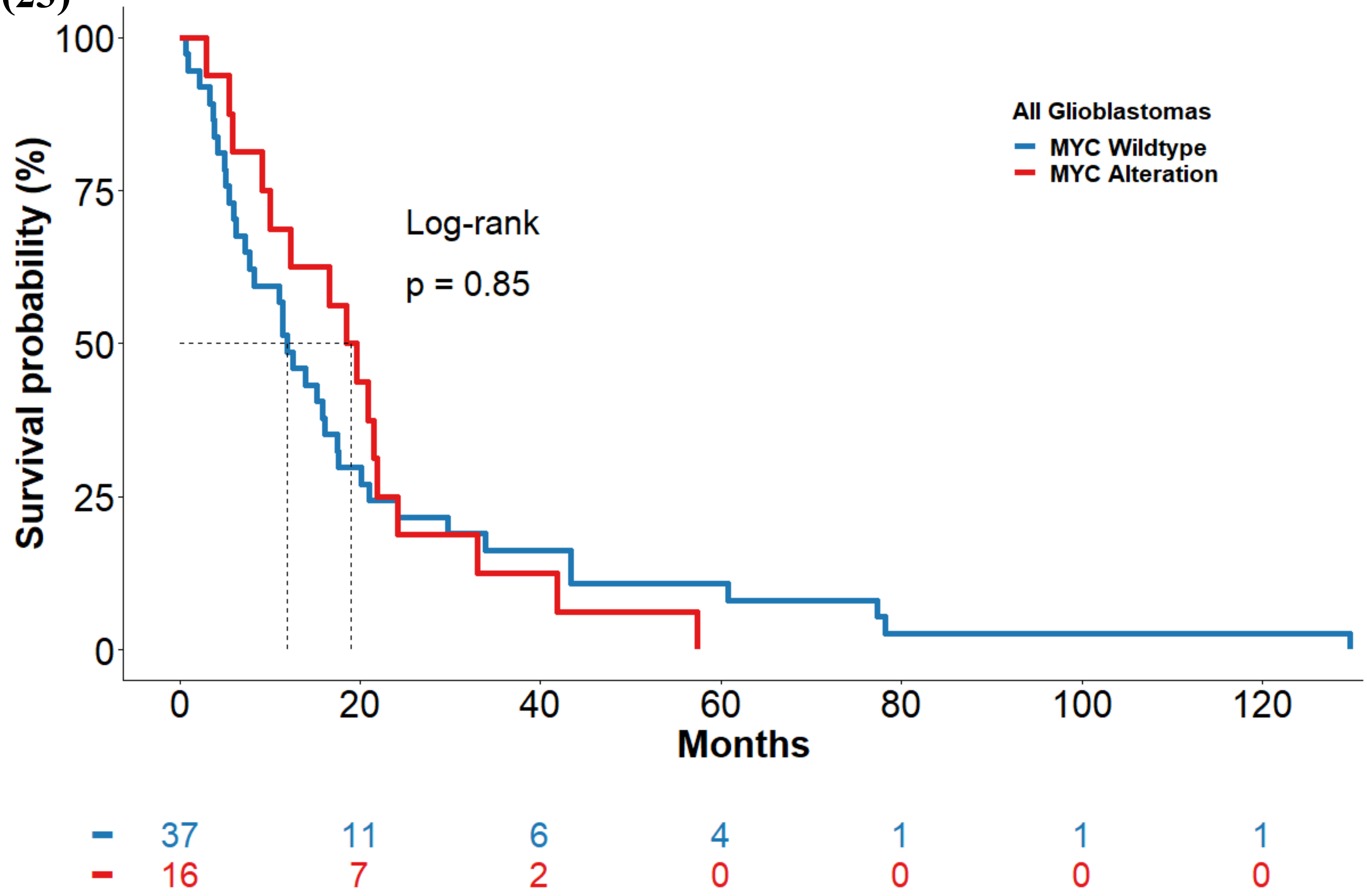

Survival probability (%)
100
75
50
25
0
Log-rank
p = 0.85
All Glioblastomas
MYC Wildtype
MYC Alteration
0
20
40
60
80
100
120
Months
37
11
6
4
1
1
1
16
7
2
0
0
0
0

(24)

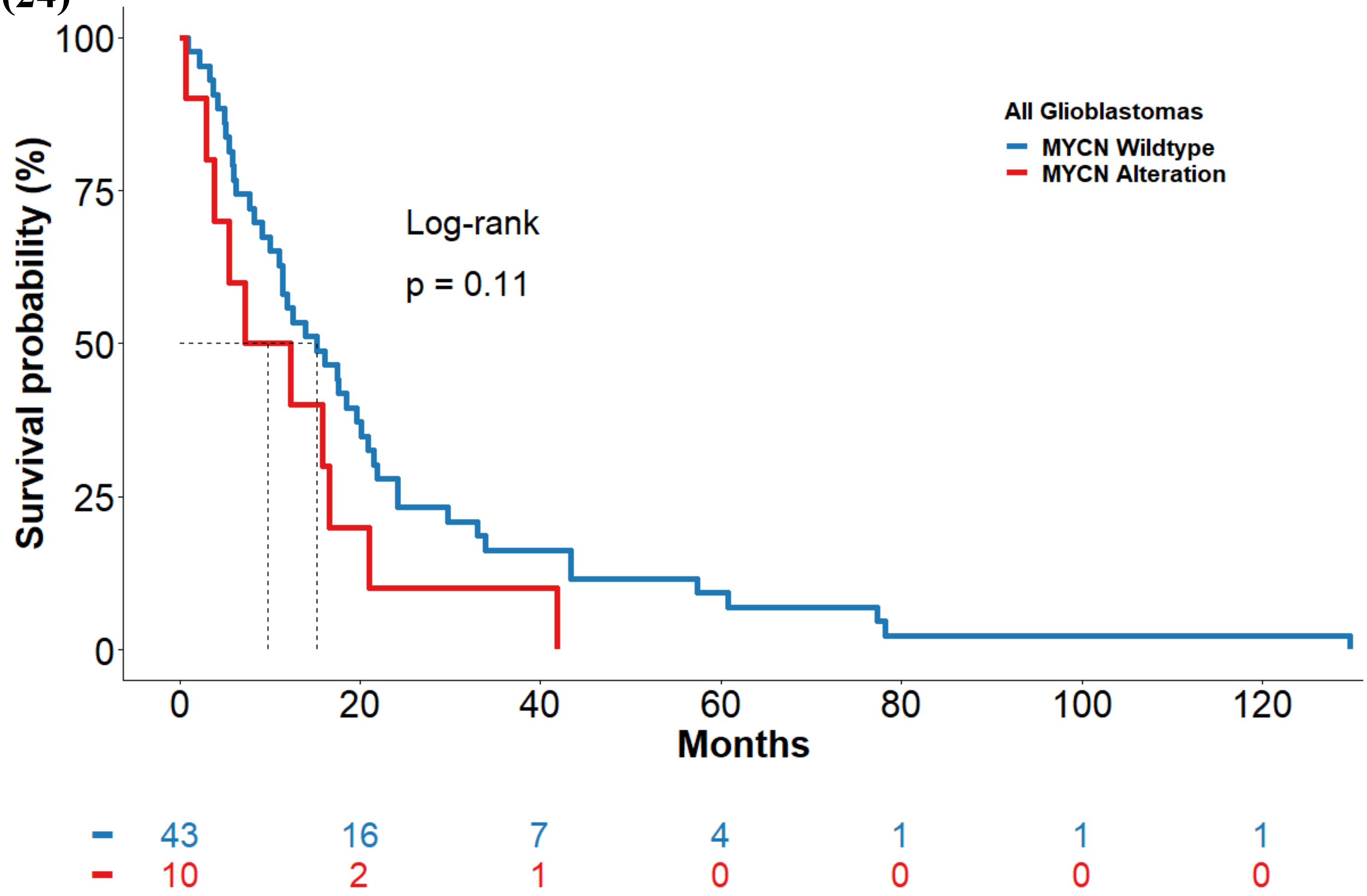
100
75
50
25
0
Survival probability (%)
Log-rank
p = 0.11
All Glioblastomas
MYCN Wildtype
MYCN Alteration
0
20
40
60
80
100
120
Months
43
16
7
4
1
1
1
10
2
1
0
0
0
0

(25)

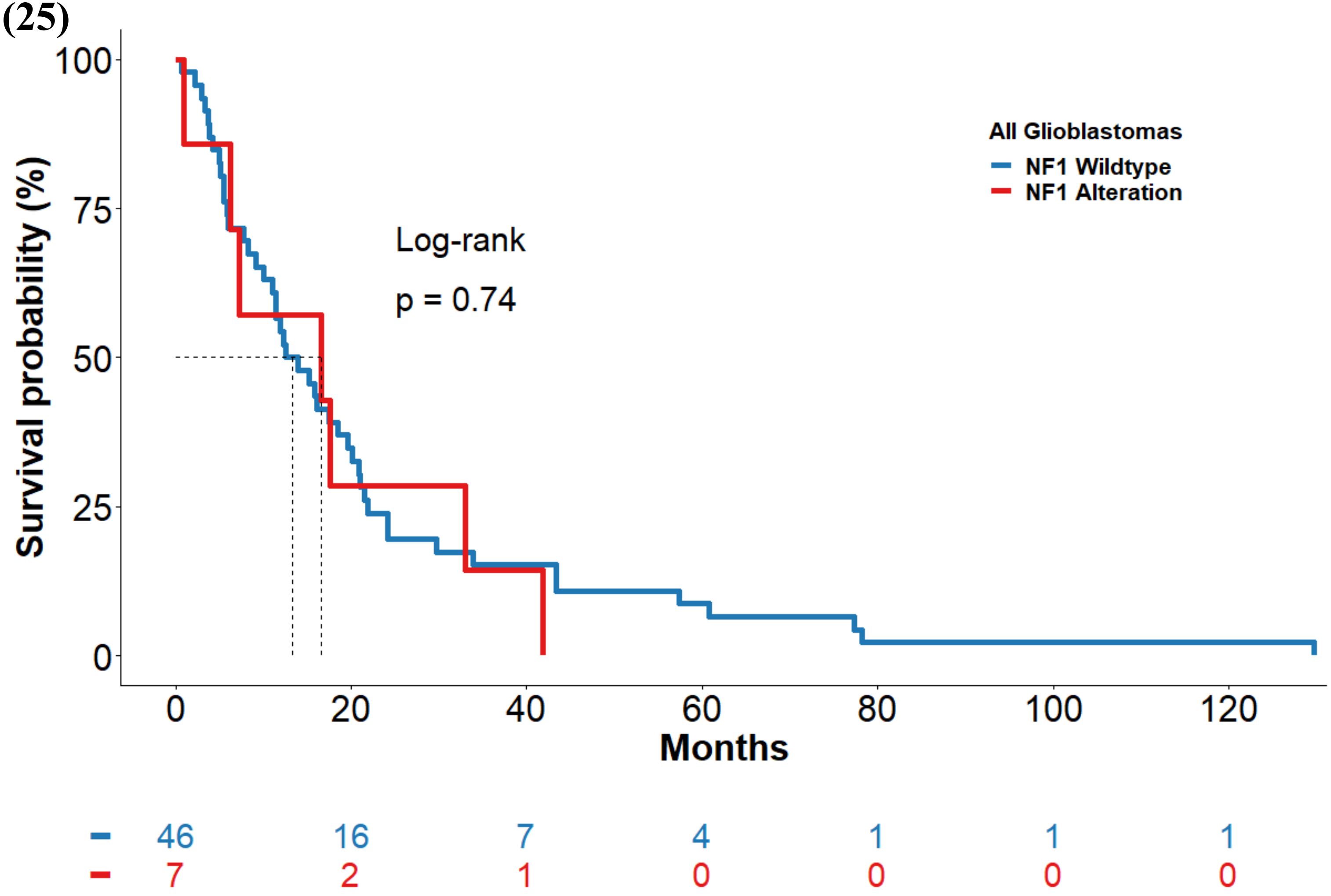
Survival probability (%)
100
75
50
25
0
Log-rank
p = 0.74
All Glioblastomas
NF1 Wildtype
NF1 Alteration
0
20
40
60
80
100
120
Months
46 16 7 4 1 1 1
7 2 1 0 0 0 0

(26)

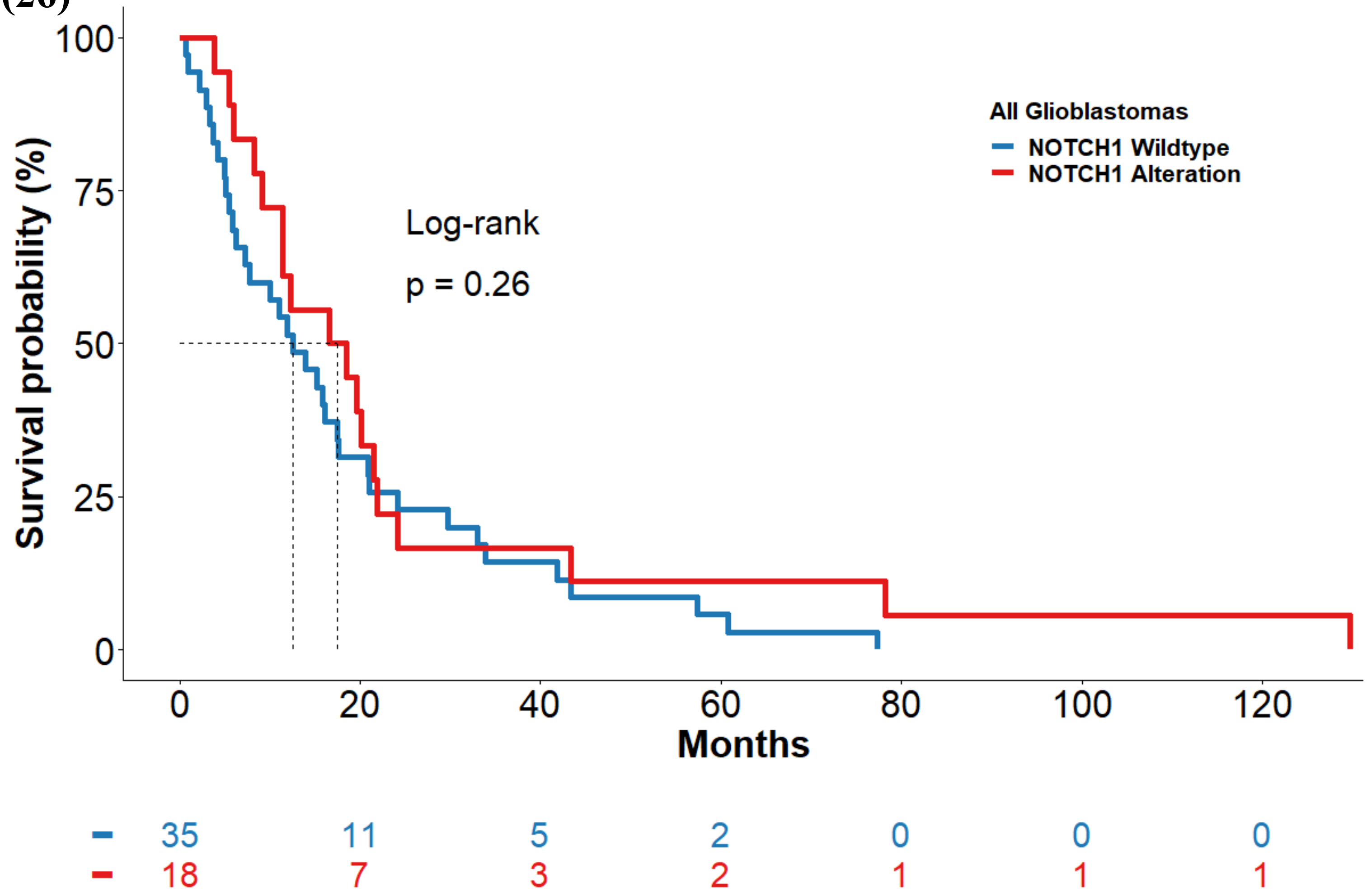
Survival probability (%)
100
75
50
25
0
Log-rank
p = 0.26
All Glioblastomas
NOTCH1 Wildtype
NOTCH1 Alteration
0
20
40
60
80
100
120
Months
35
11
5
2
0
0
0
18
7
3
2
1
1
1

(27)

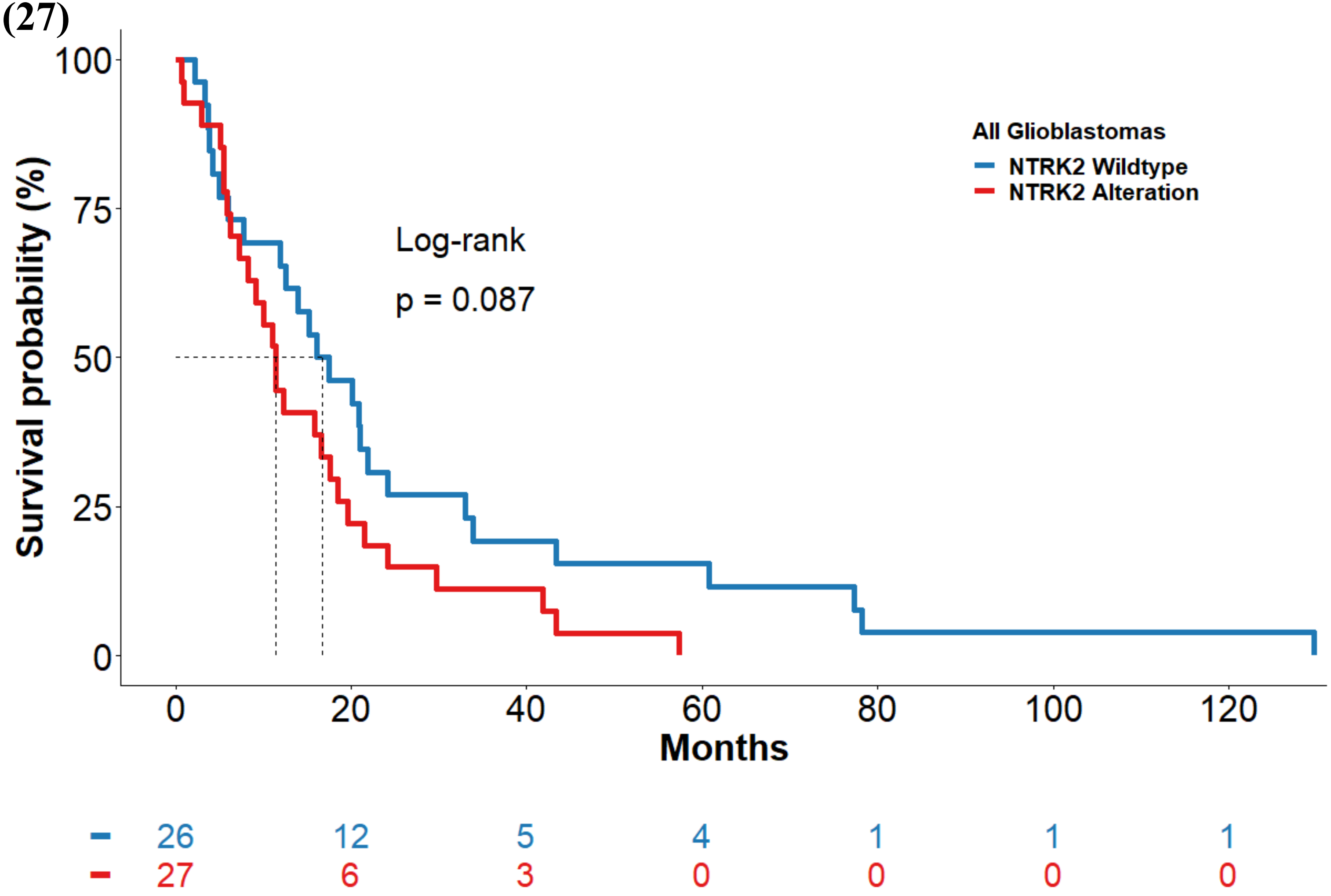

Survival probability (%)
100
75
50
25
0
Log-rank
p = 0.087
All Glioblastomas
NTRK2 Wildtype
NTRK2 Alteration
0
20
40
60
80
100
120
Months
26
12
5
4
1
1
1
27
6
3
0
0
0
0

(28)

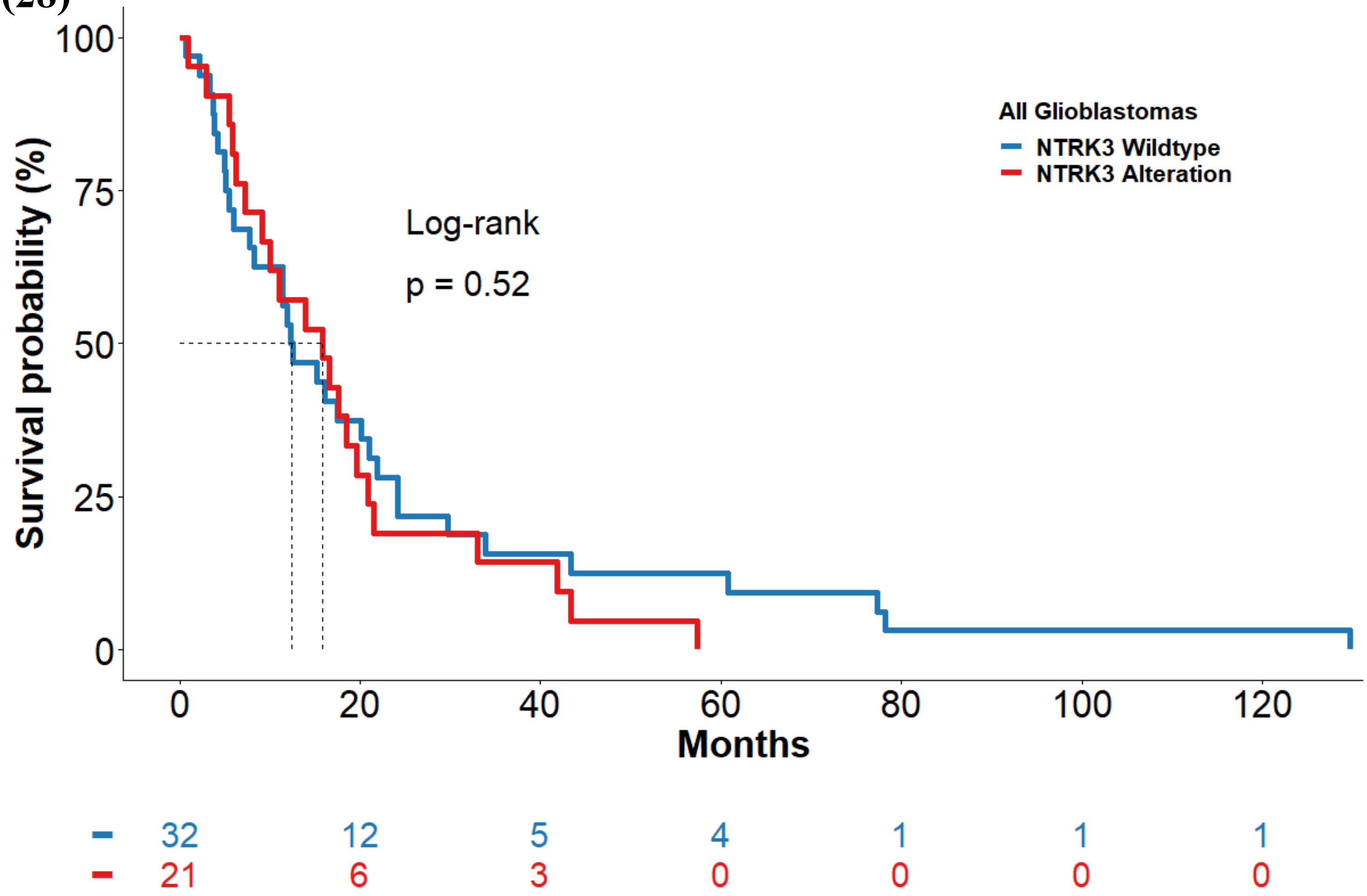
All Glioblastomas
NTRK3 Wildtype
NTRK3 Alteration
Log-rank
p = 0.52
Survival probability (%)
100
75
50
25
0
0
20
40
60
80
100
120
Months
32 12 5 4 1 1 1
21 6 3 0 0 0 0

(29)

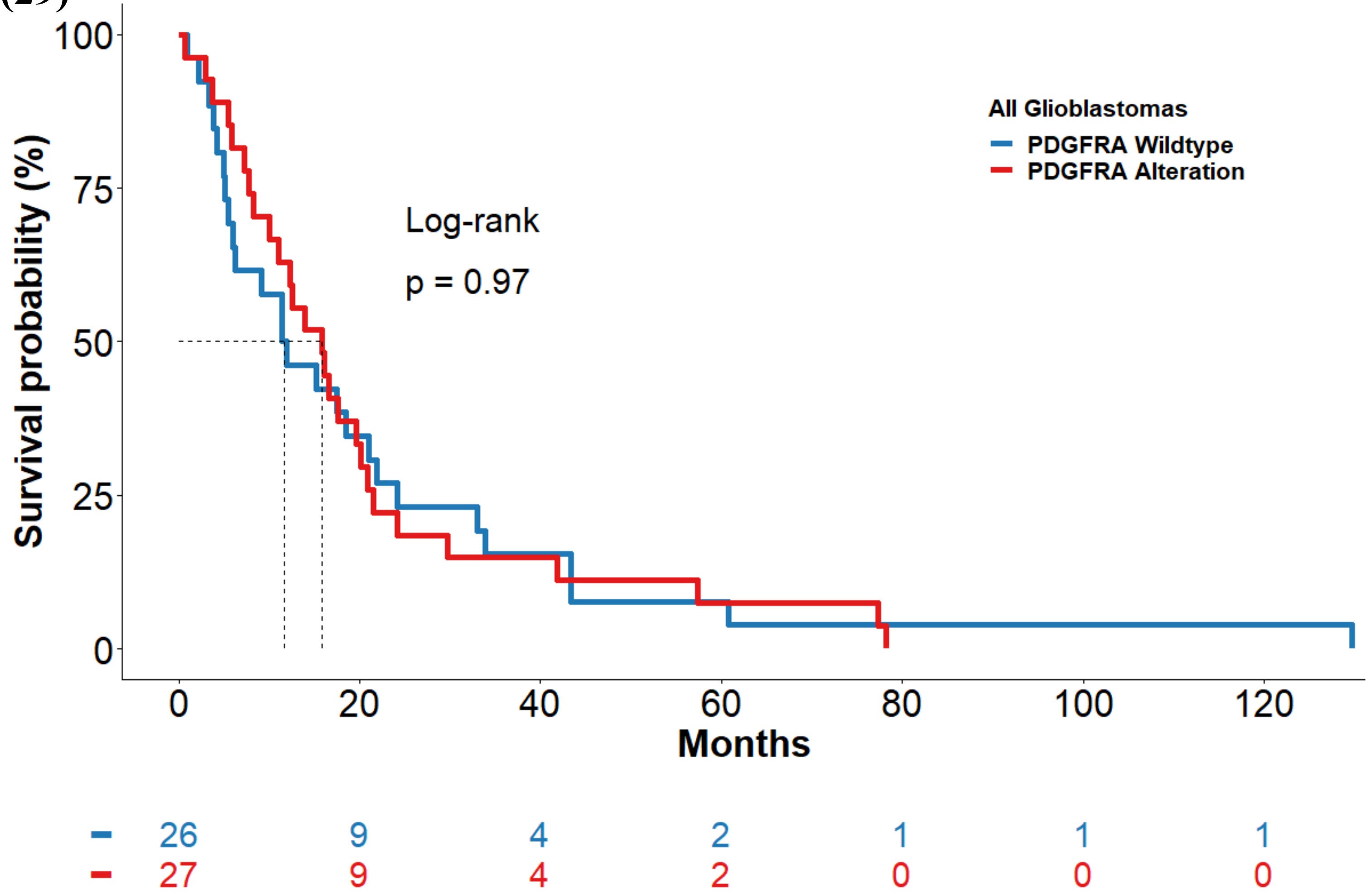

Survival probability (%)
100
75
50
25
0
Log-rank
p = 0.97
All Glioblastomas
PDGFRA Wildtype
PDGFRA Alteration
0
20
40
60
80
100
120
Months
26 9 4 2 1 1 1
27 9 4 2 0 0 0

(30)

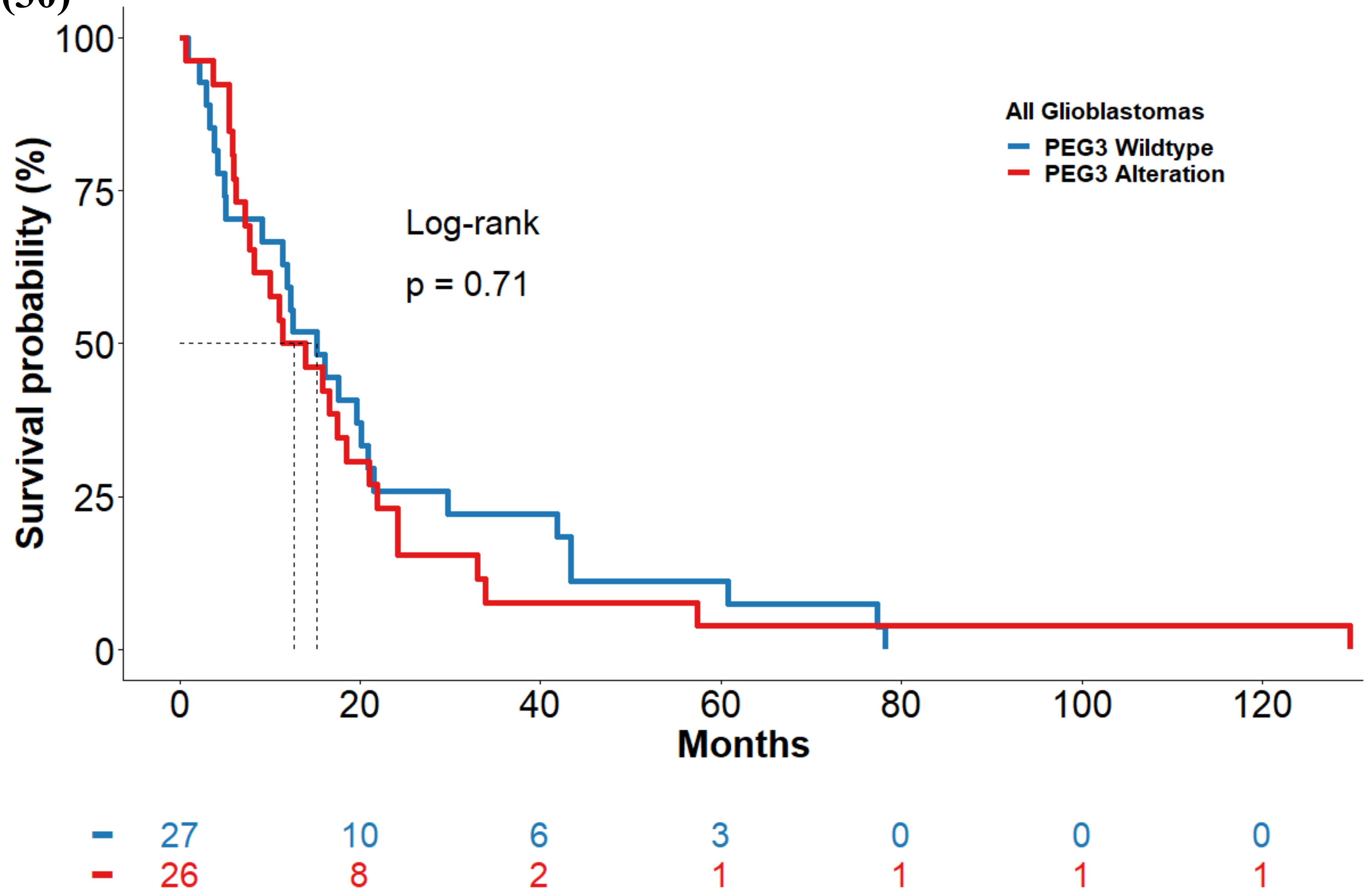

Survival probability (%)
100
75
50
25
0
Log-rank
p = 0.71
All Glioblastomas
PEG3 Wildtype
PEG3 Alteration
0
20
40
60
80
100
120
Months
27
10
6
3
0
0
0
26
8
2
1
1
1
1

(31)

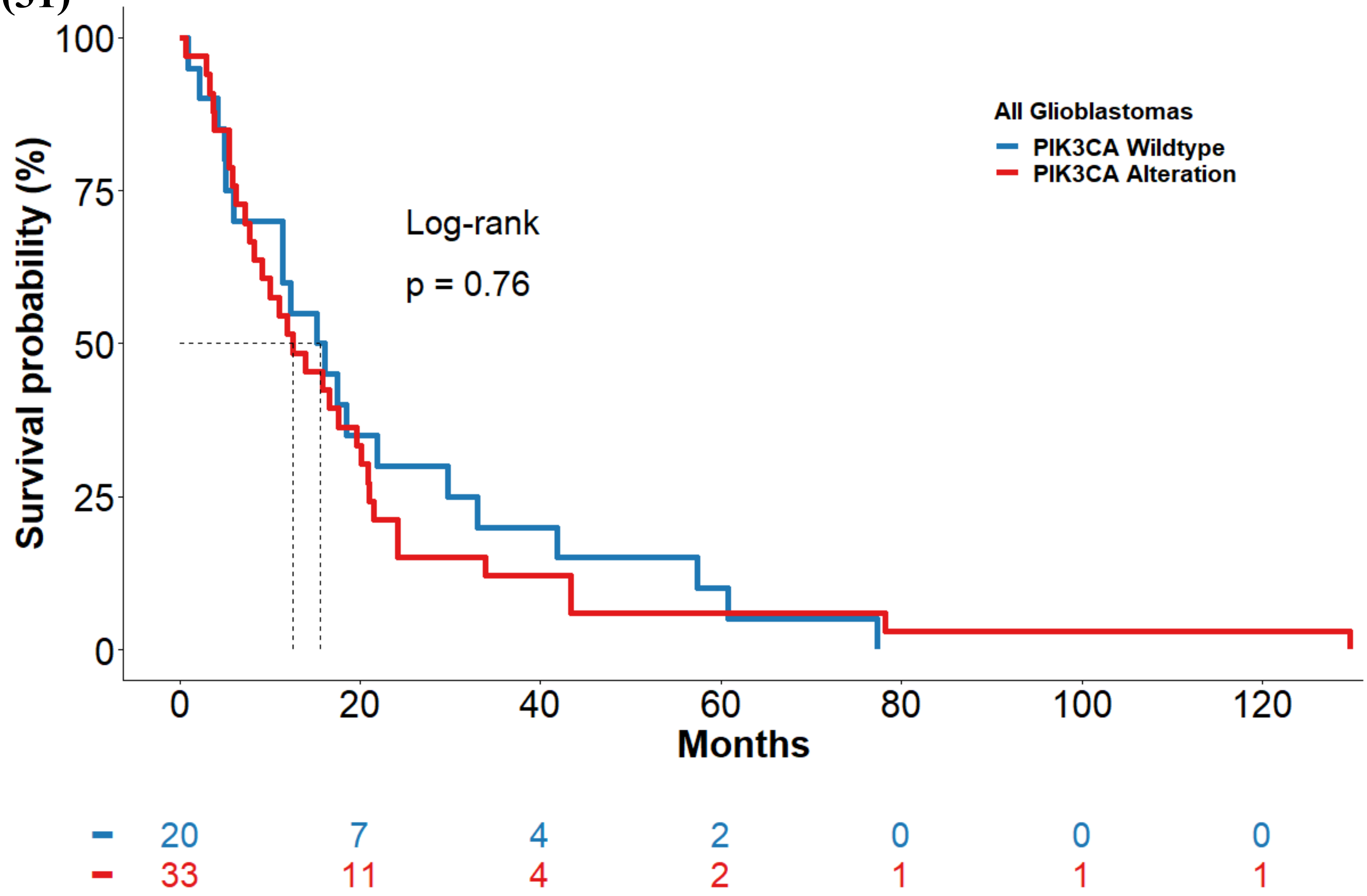

Survival probability (%)
100
75
50
25
0
Log-rank
p = 0.76
All Glioblastomas
PIK3CA Wildtype
PIK3CA Alteration
0
20
40
60
80
100
120
Months
20 7 4 2 0 0 0
33 11 4 2 1 1 1

(32)

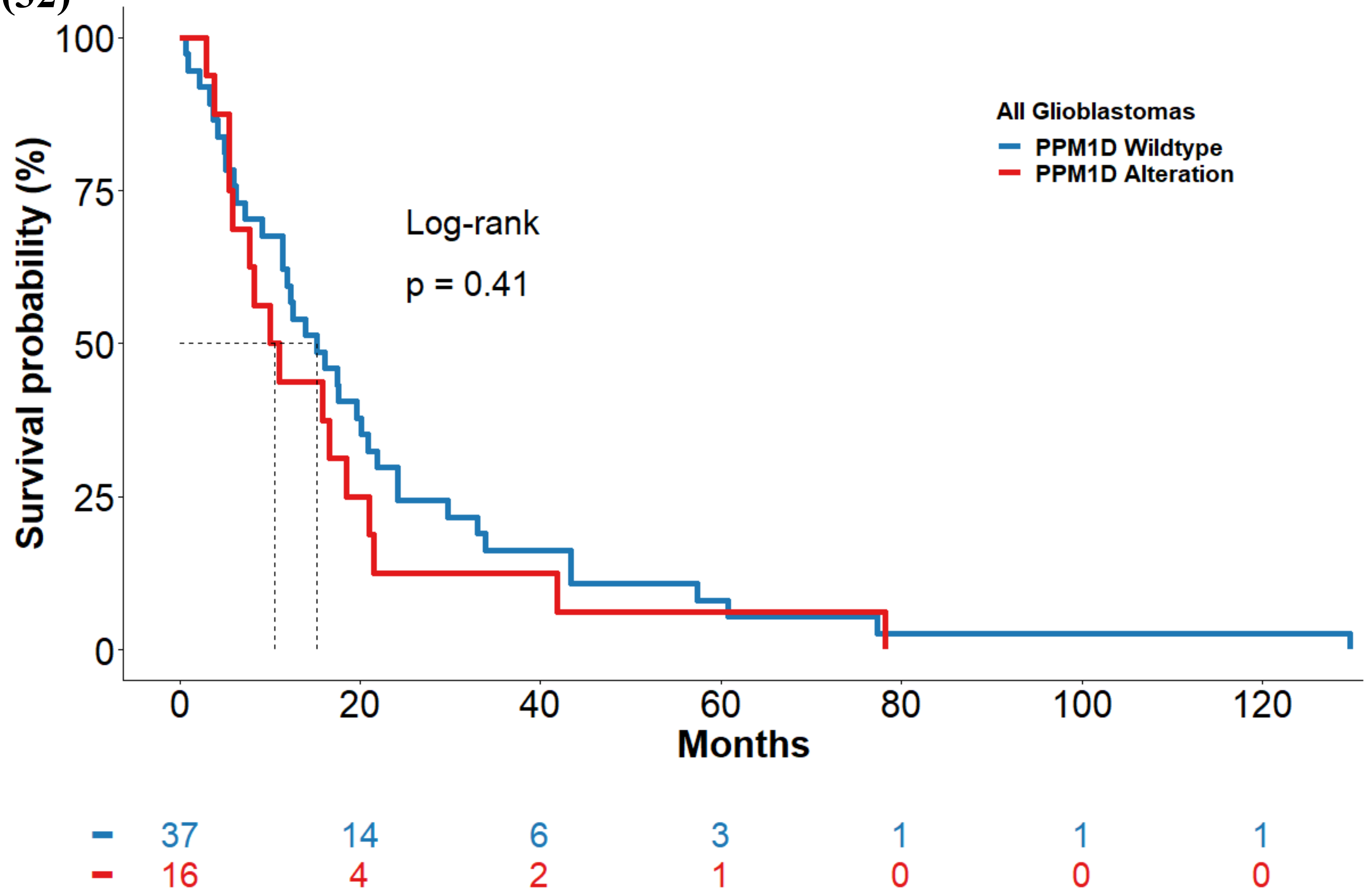
Survival probability (%)
100
75
50
25
0
Log-rank
p = 0.41
All Glioblastomas
PPM1D Wildtype
PPM1D Alteration
0
20
40
60
80
100
120
Months
37 14 6 3 1 1 1
16 4 2 1 0 0 0

(33)

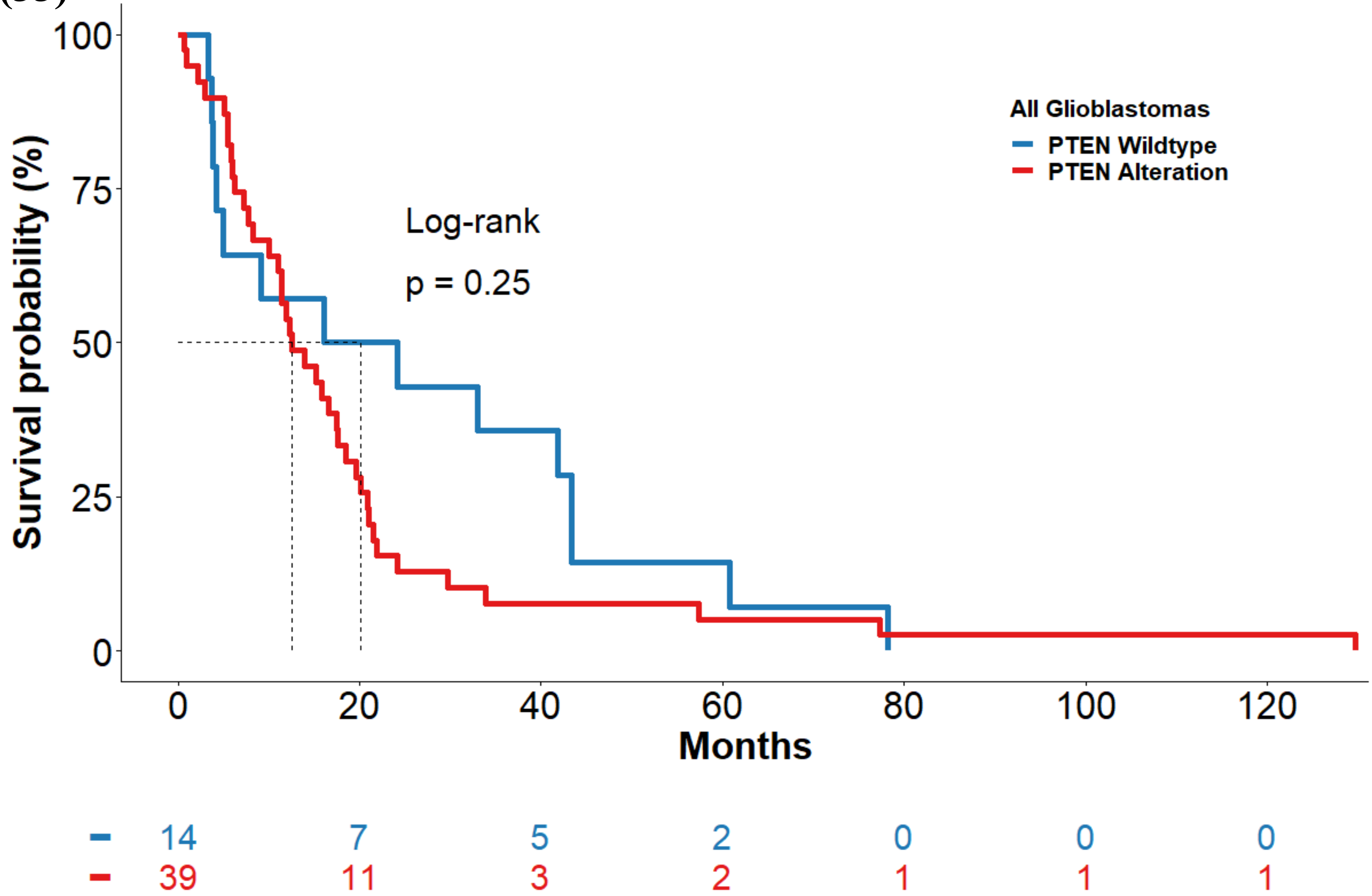

Survival probability (%)
100
75
50
25
0
Log-rank
p = 0.25
All Glioblastomas
PTEN Wildtype
PTEN Alteration
0
20
40
60
80
100
120
Months
14 7 5 2 0 0 0
39 11 3 2 1 1 1

(34)

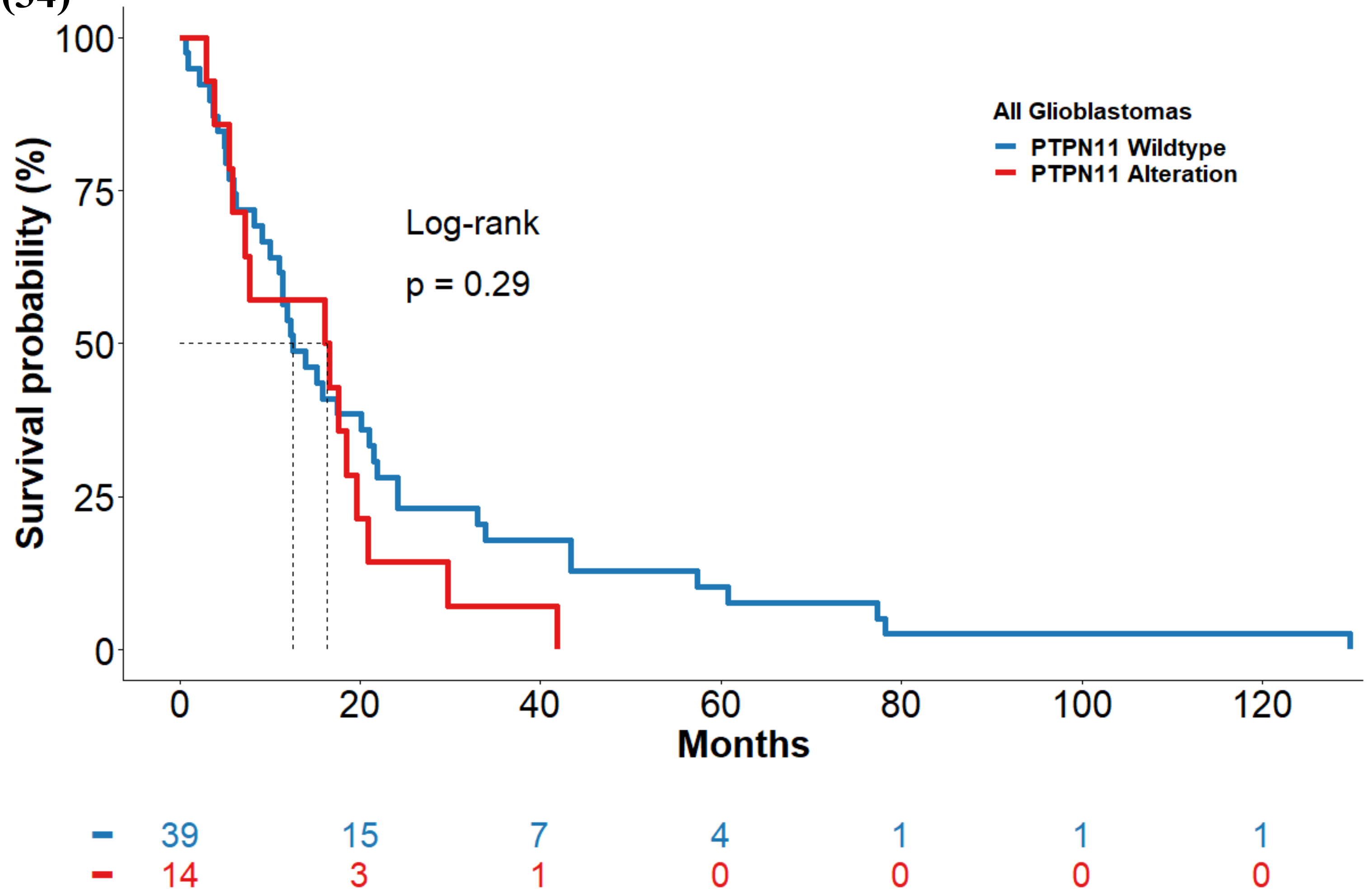

100
75
50
25
0
Survival probability (%)
Log-rank
p = 0.29
All Glioblastomas
PTPN11 Wildtype
PTPN11 Alteration
0
20
40
60
80
100
120
Months
39
15
7
4
1
1
1
14
3
1
0
0
0
0

(35)

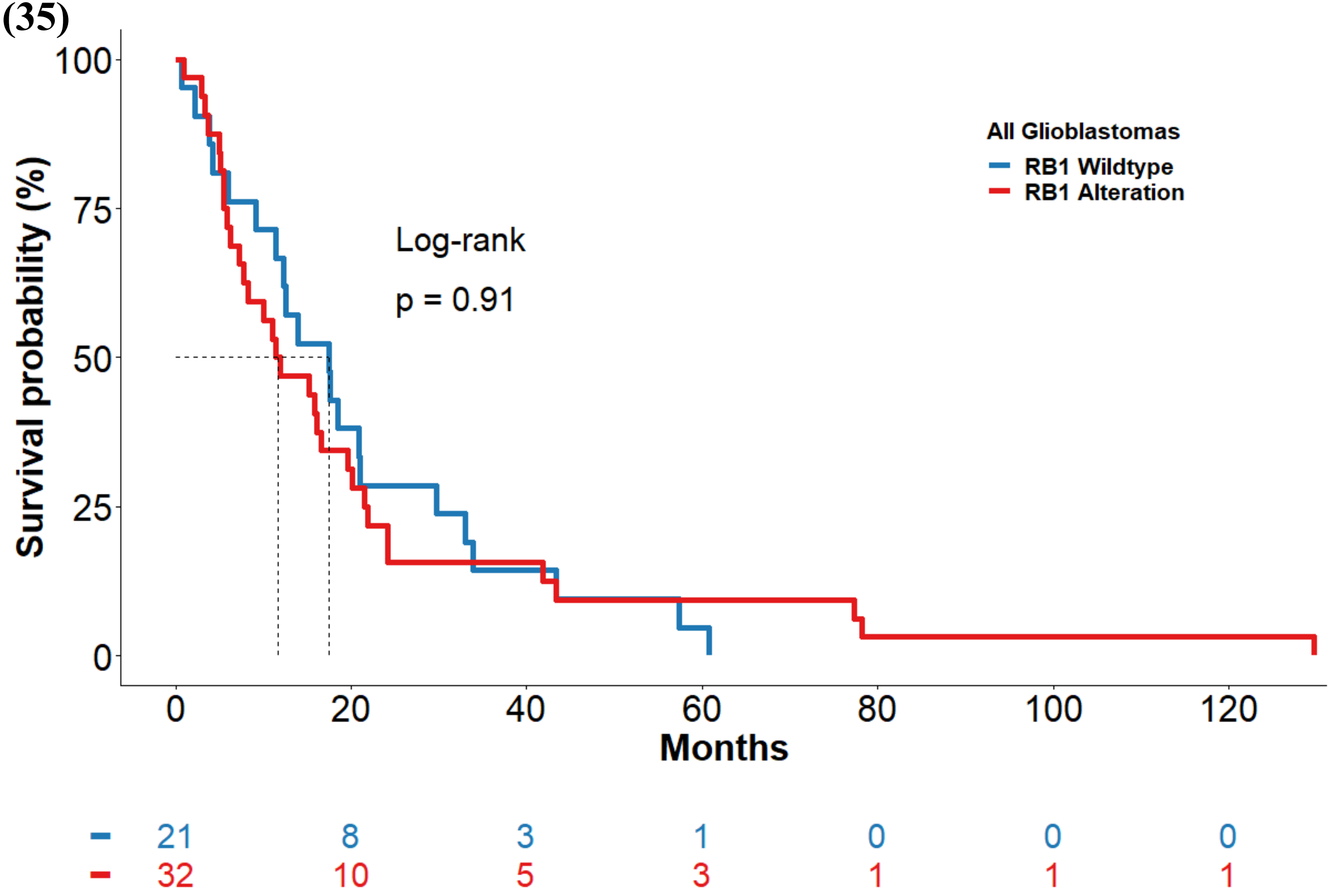

100
75
50
25
0
Survival probability (%)
Log-rank
p = 0.91
All Glioblastomas
RB1 Wildtype
RB1 Alteration
0
20
40
60
80
100
120
Months
21
8
3
1
0
0
0
32
10
5
3
1
1
1

(36)

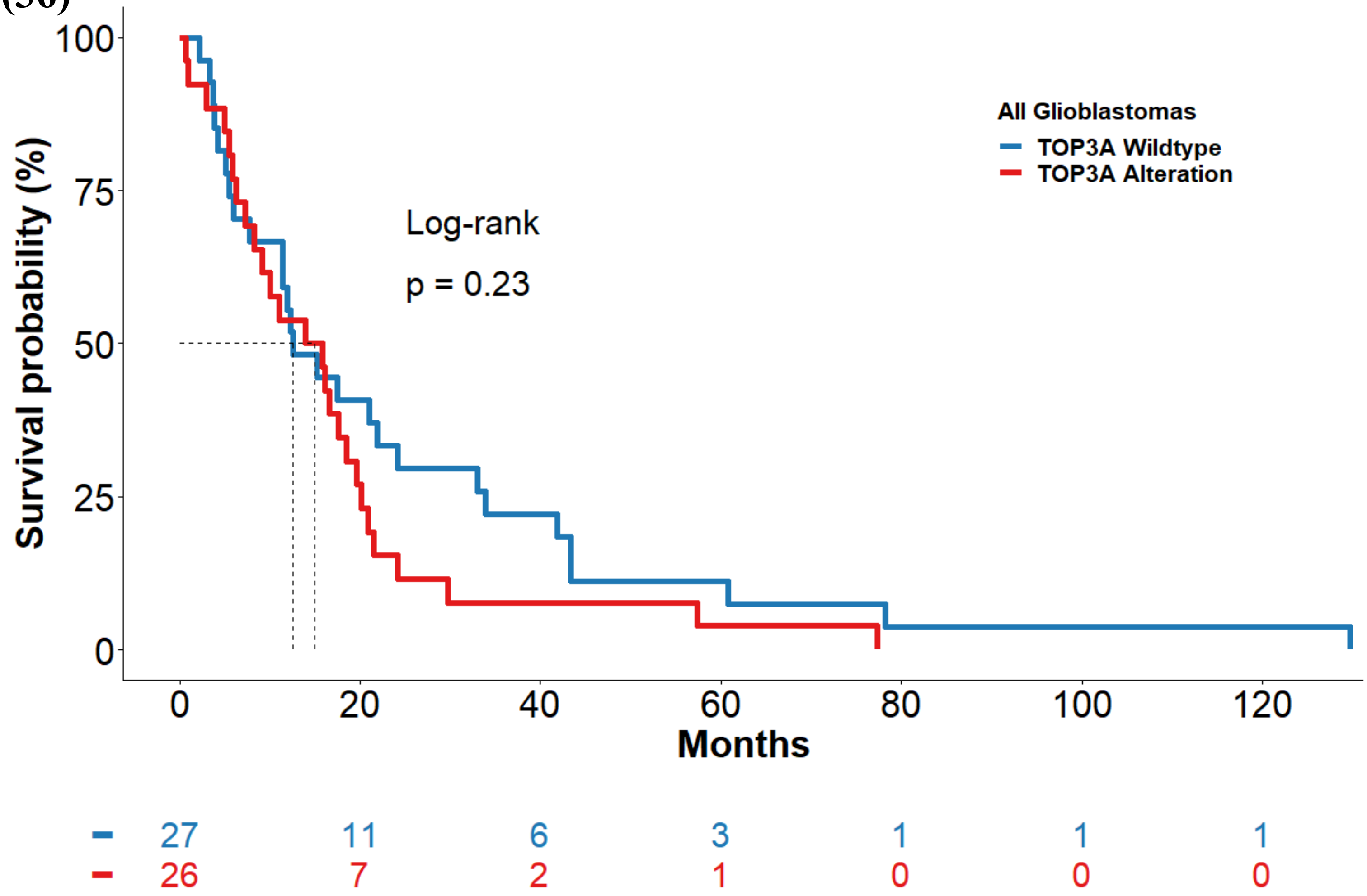

Survival probability (%)
100
75
50
25
0
Log-rank
p = 0.23
All Glioblastomas
TOP3A Wildtype
TOP3A Alteration
0
20
40
60
80
100
120
Months
27 11 6 3 1 1 1
26 7 2 1 0 0 0

(37)

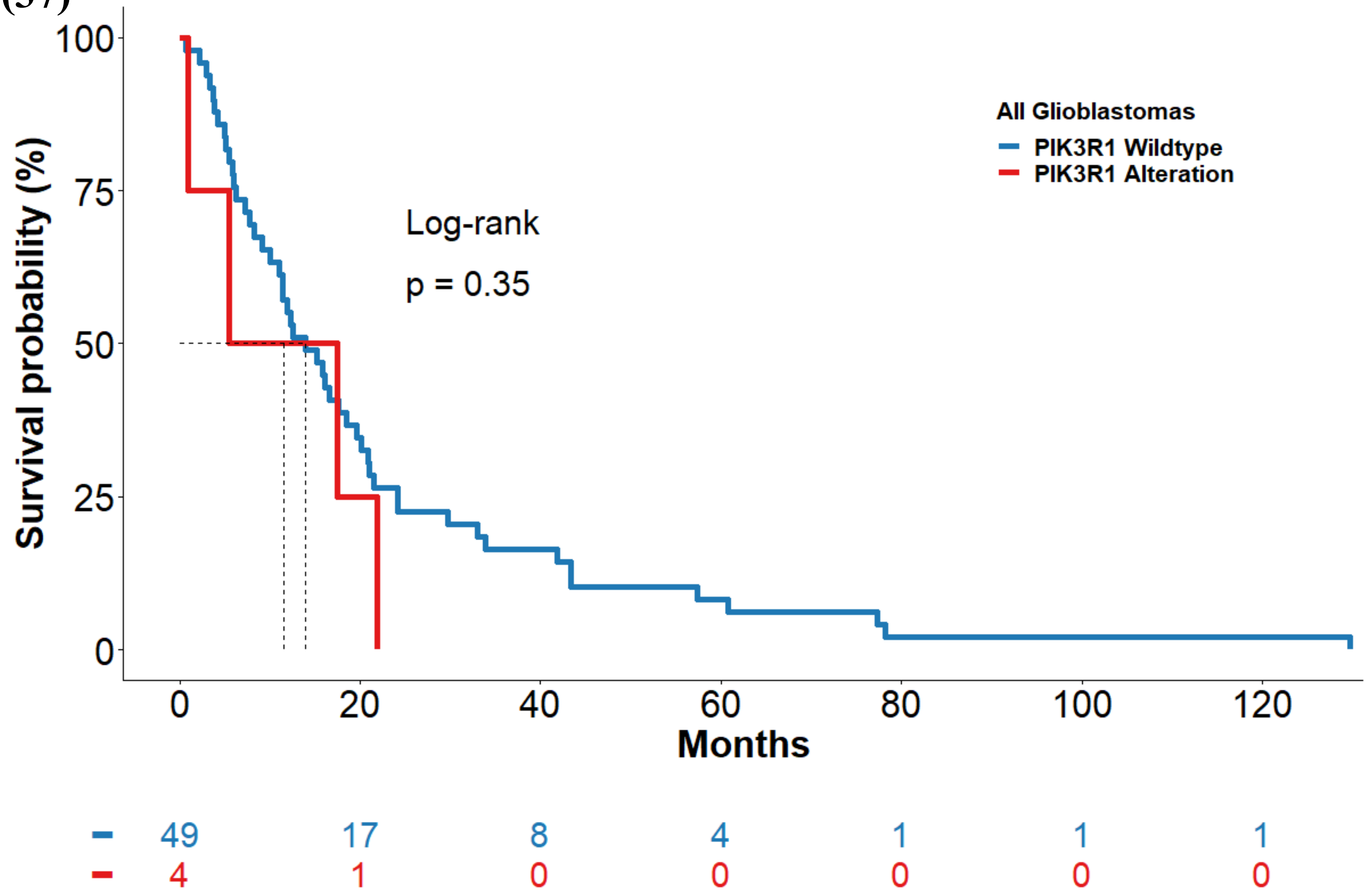

All Glioblastomas
PIK3R1 Wildtype
PIK3R1 Alteration
Log-rank
p = 0.35
Survival probability (%)
100
75
50
25
0
0
20
40
60
80
100
120
Months
49
17
8
4
1
1
1
4
1
0
0
0
0
0

(38)

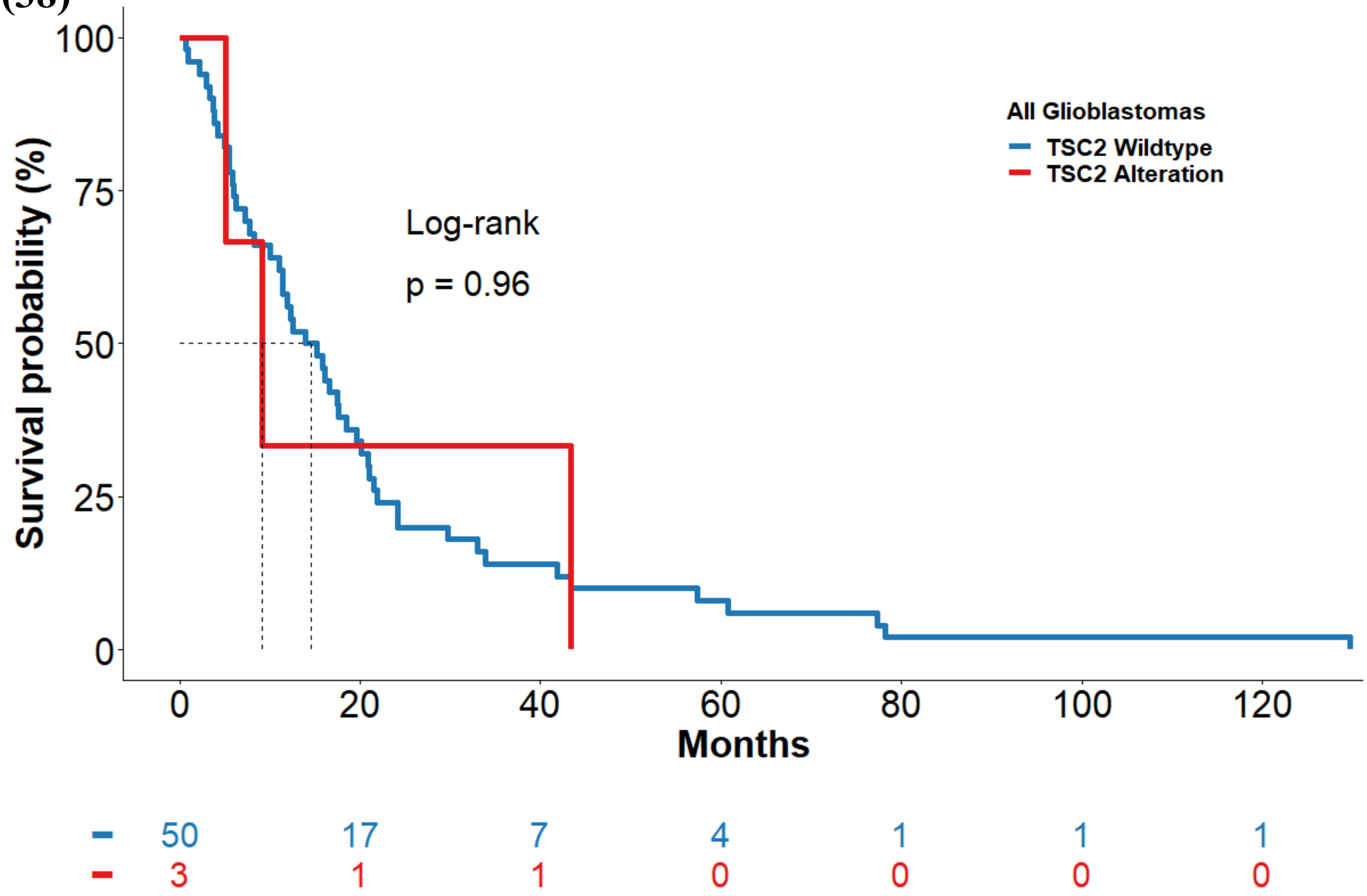
All Glioblastomas
TSC2 Wildtype
TSC2 Alteration
Log-rank
p = 0.96
Survival probability (%)
100
75
50
25
0
0
20
40
60
80
100
120
Months
50 17 7 4 1 1 1
3 1 1 0 0 0 0

(39)

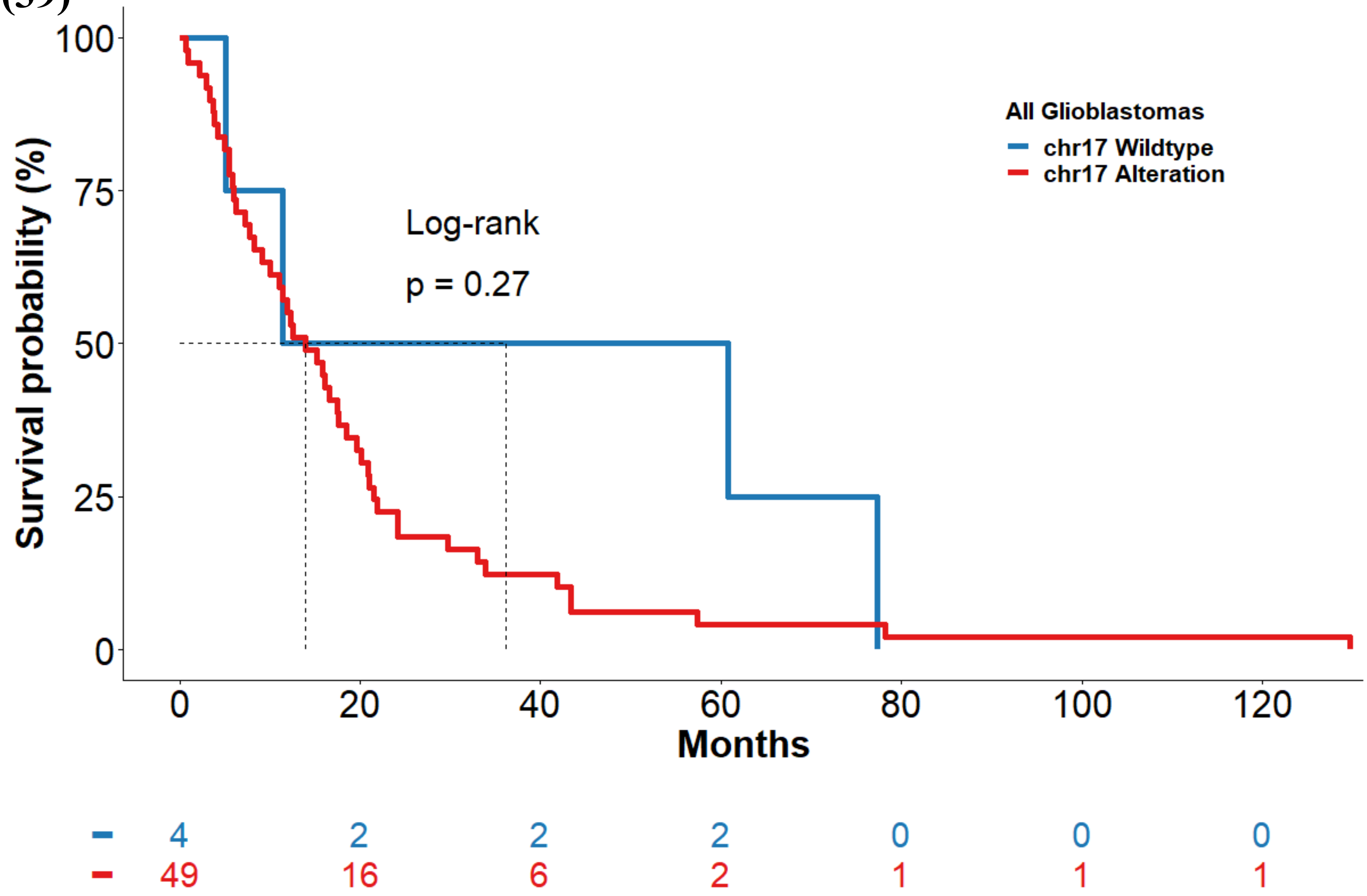

All Glioblastomas
chr17 Wildtype
chr17 Alteration
Log-rank
p = 0.27
Survival probability (%)
100
75
50
25
0
0
20
40
60
80
100
120
Months
4 2 2 2 0 0 0
49 16 6 2 1 1 1
