# Supplementary material for: Histological and molecular glioblastoma, IDH-wildtype: a real-world landscape using the 2021 WHO classification of central nervous system tumors
Source: Front Oncol. 2023 Jul 6;13:1200815. doi: 10.3389/fonc.2023.1200815 (PMC10358772; doi:10.3389/fonc.2023.1200815)
Supplement: Supplementary file 2 [file Image_2.pdf]

Supplementary Figure 2. Kaplan–Meier curves showing no significant effects of certain genetic and immunohistochemical alterations on the overall survival of patients with histological glioblastomas (1-30). Since the numbers of patients divided into two groups (e.g. FGFR1 Wildtype/FGFR1 Alteration) using different parameters varied, we only enrolled the parameters using which the number of patients in either group was above 3. Parameters used in this section were shown as follows.

(1) Sex 1
(2) Ki-67 2
(3) ATRX 3
(4) TP53 4
(5) Oligo2 5
(6) NeuN 6
(7) Syn 7
(8) TERT promotor mutation 8
(9) EGFR Amplification 9
(10) CDKN2A/B homozygous deletion 10
(11) BRAF 11
(12) CDK4 12
(13) FGFR1 13
(14) FGFR3 14
(15) FGFR4 15
(16) KIT 16
(17) MET 17
(18) MYC 18
(19) MYCN 19
(20) NF1 20
(21) NOTCH1 21
(22) NTRK2 22
(23) NTRK3 23
(24) PDGFRA 24
(25) PEG3 25
(26) PIK3CA 26
(27) PPM1D 27
(28) PTPN11 28
(29) RB1 29
(30) chr17 30

(1)

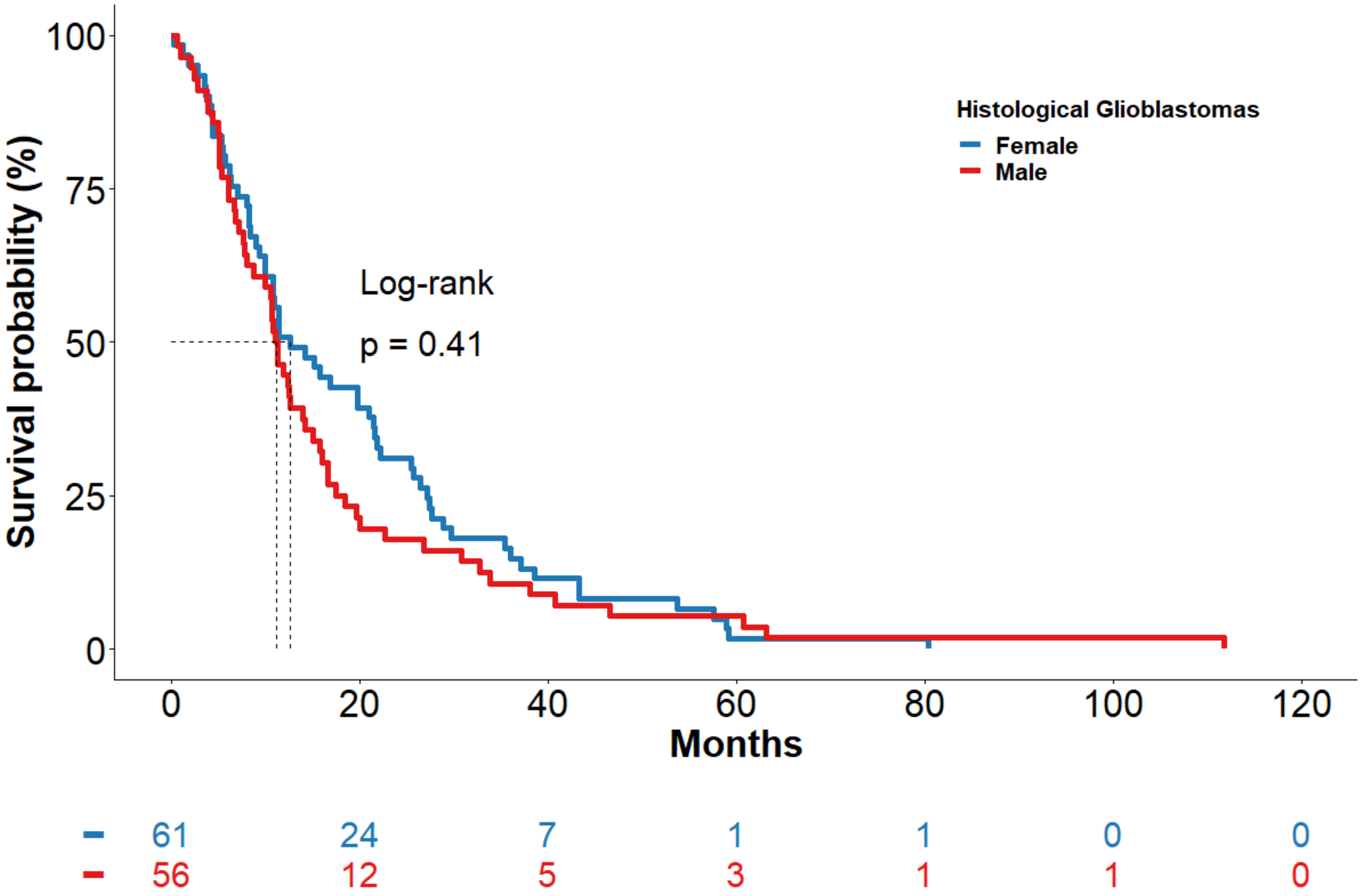

Survival probability (%)
100
75
50
25
0
Log-rank
p = 0.41
Histological Glioblastomas
Female
Male
0
20
40
60
80
100
120
Months
61 24 7 1 1 0 0
56 12 5 3 1 1 0

(2)

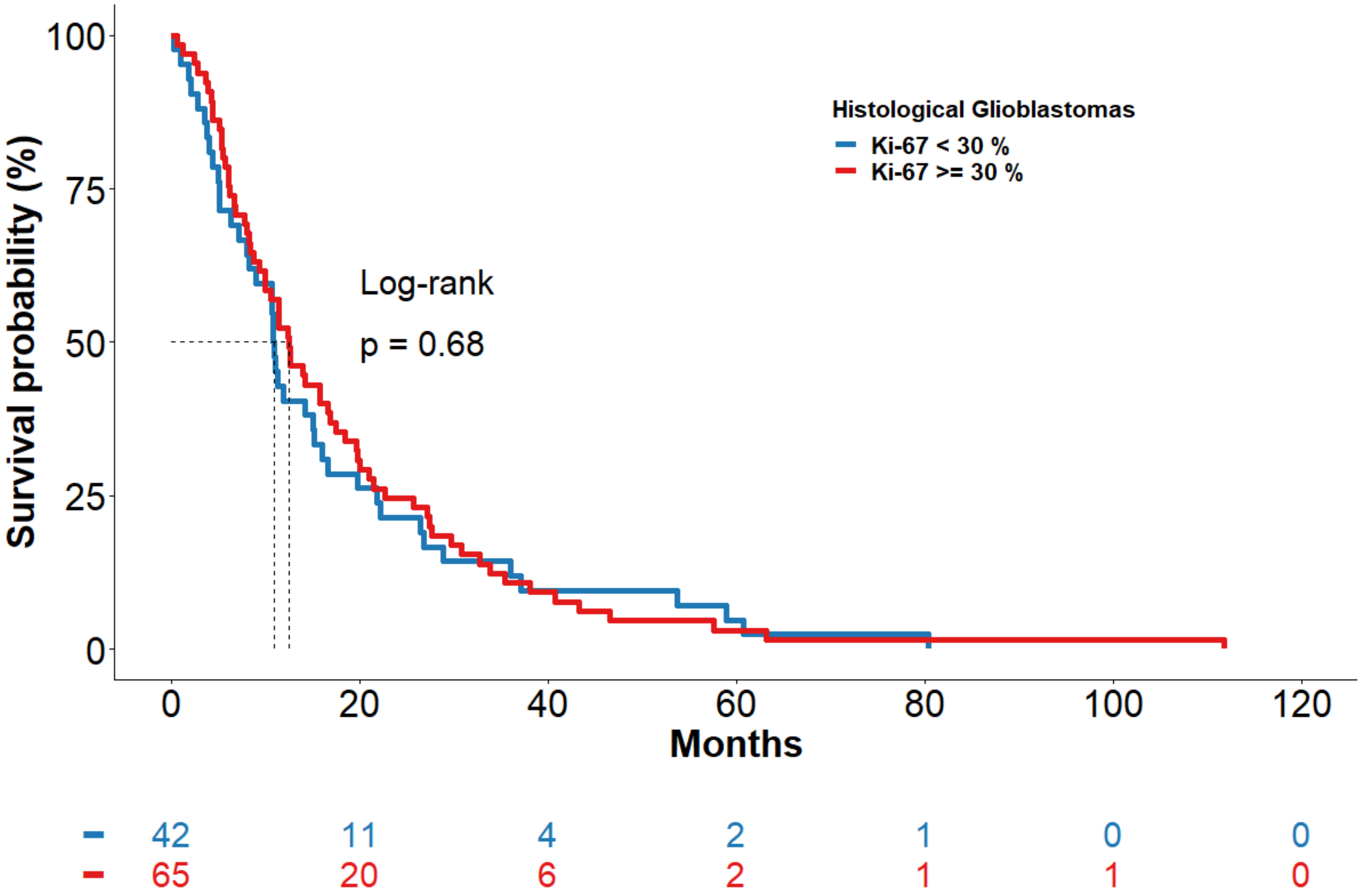
Survival probability (%)
100
75
50
25
0
Histological Glioblastomas
Ki-67 < 30 %
Ki-67 >= 30 %
Log-rank
p = 0.68
0
20
40
60
80
100
120
Months
42 11 4 2 1 0 0
65 20 6 2 1 1 0

(3)

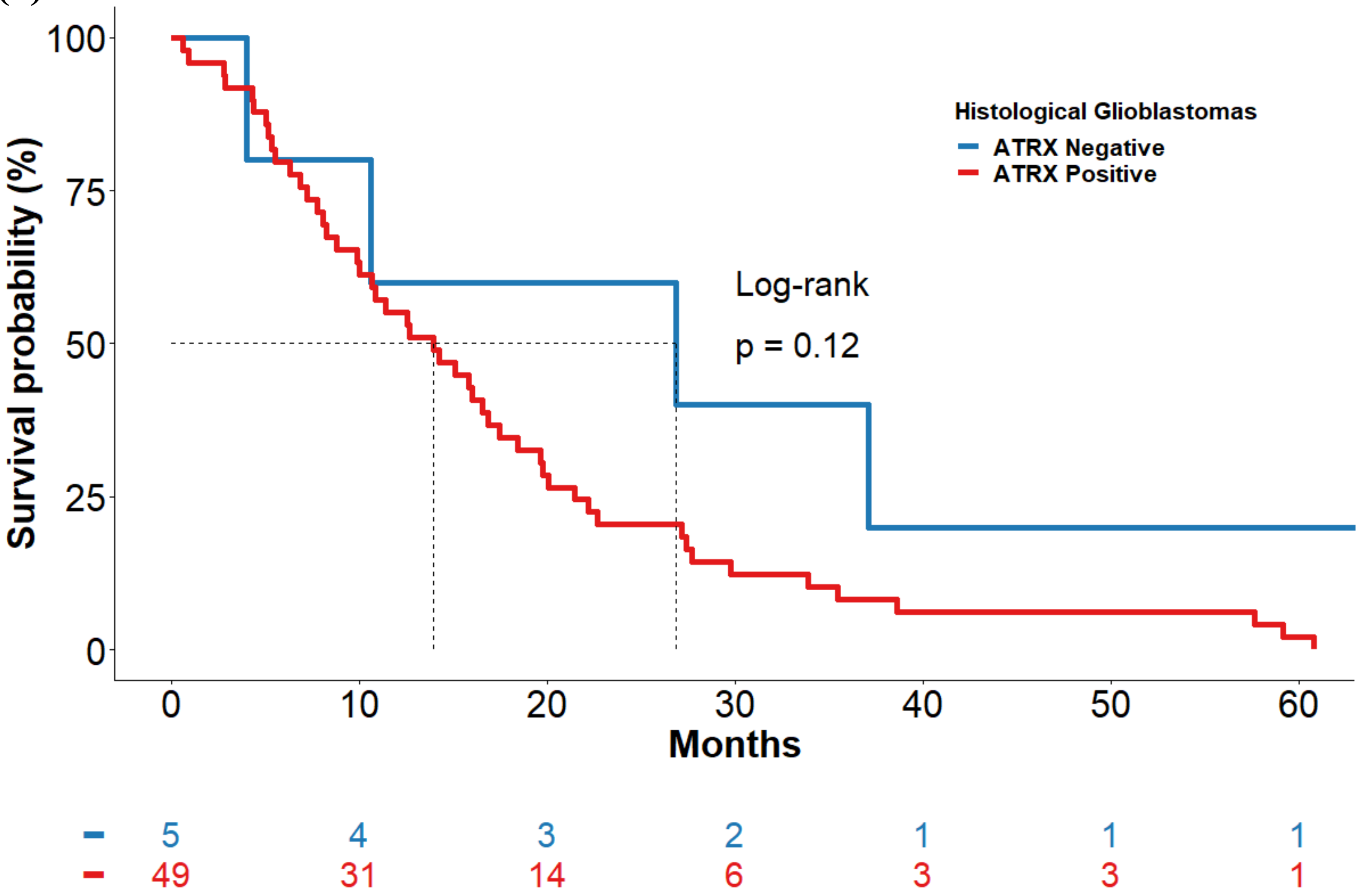

Survival probability (%)
100
75
50
25
0
Histological Glioblastomas
ATRX Negative
ATRX Positive
Log-rank
p = 0.12
0
10
20
30
40
50
60
Months
5 4 3 2 1 1 1
49 31 14 6 3 3 1

(4)

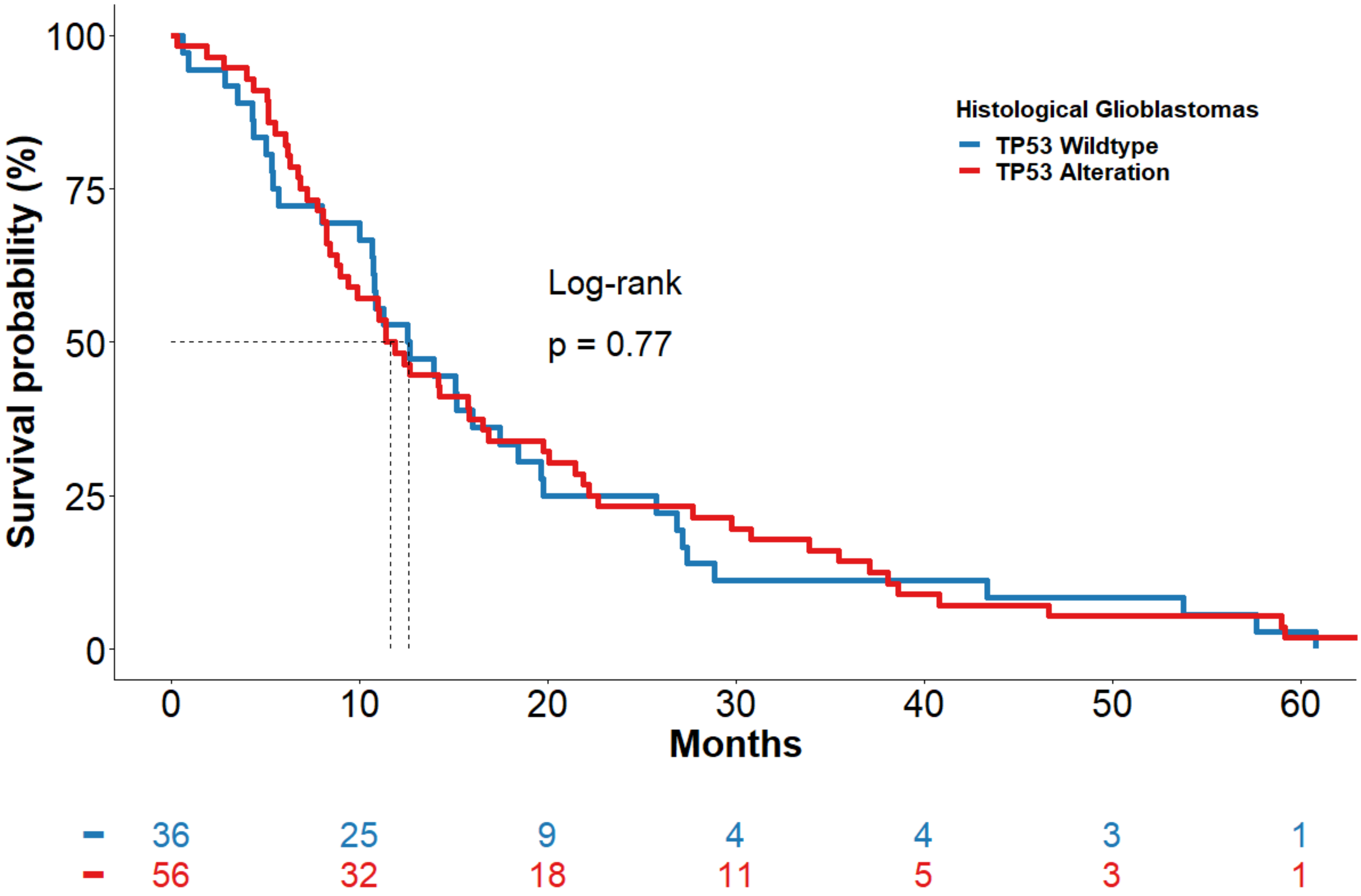

Survival probability (%)
100
75
50
25
0
Log-rank
p = 0.77
Histological Glioblastomas
TP53 Wildtype
TP53 Alteration
0
10
20
30
40
50
60
Months
36
25
9
4
4
3
1
56
32
18
11
5
3
1

(5)

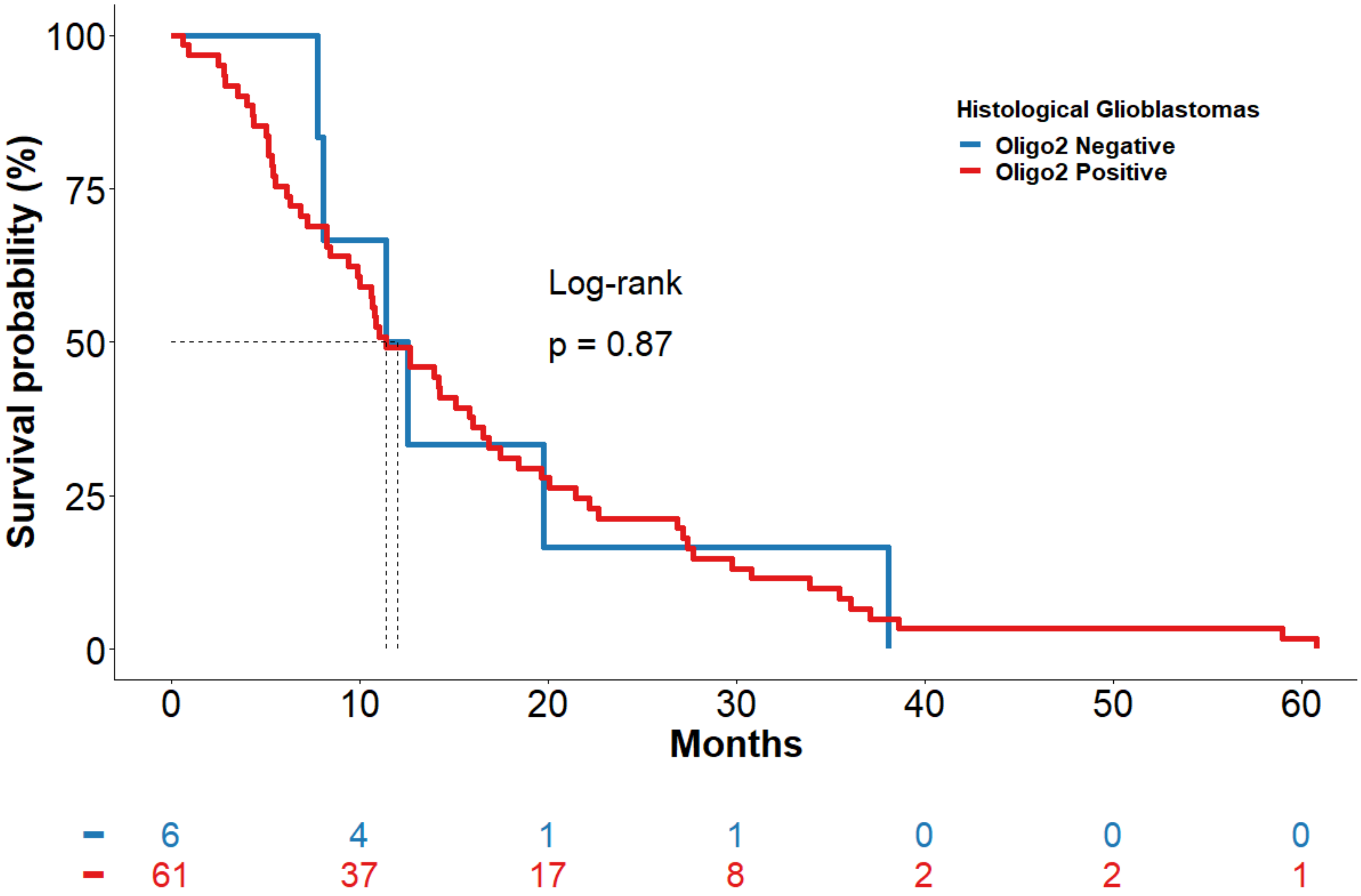
Survival probability (%)
100
75
50
25
0
Log-rank
p = 0.87
Histological Glioblastomas
Oligo2 Negative
Oligo2 Positive
0
10
20
30
40
50
60
Months
6 4 1 1 0 0 0
61 37 17 8 2 2 1

(6)

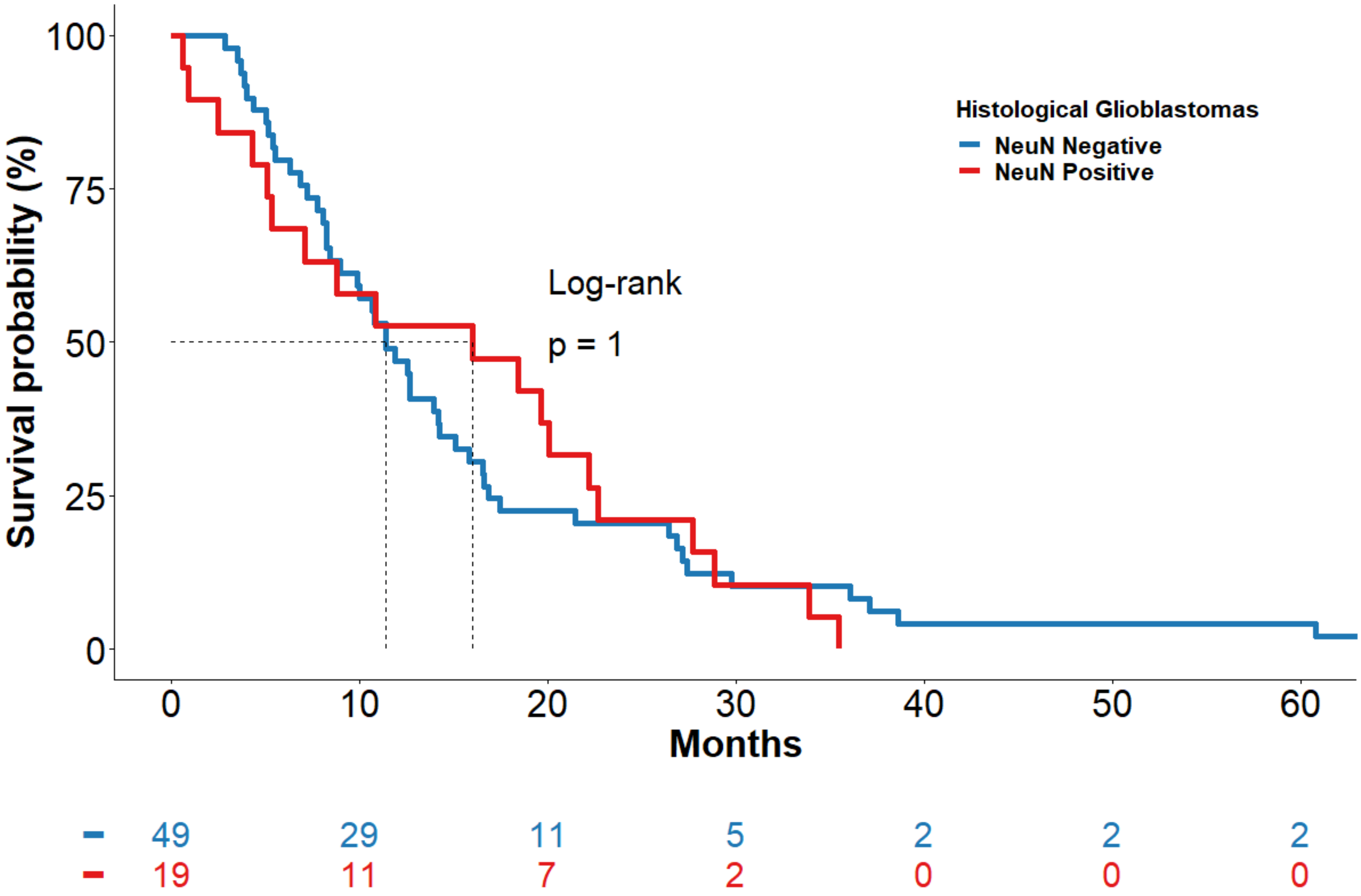

Histological Glioblastomas
NeuN Negative
NeuN Positive
Log-rank
p = 1
Survival probability (%)
100
75
50
25
0
0
10
20
30
40
50
60
Months
49 29 11 5 2 2 2
19 11 7 2 0 0 0

(7)

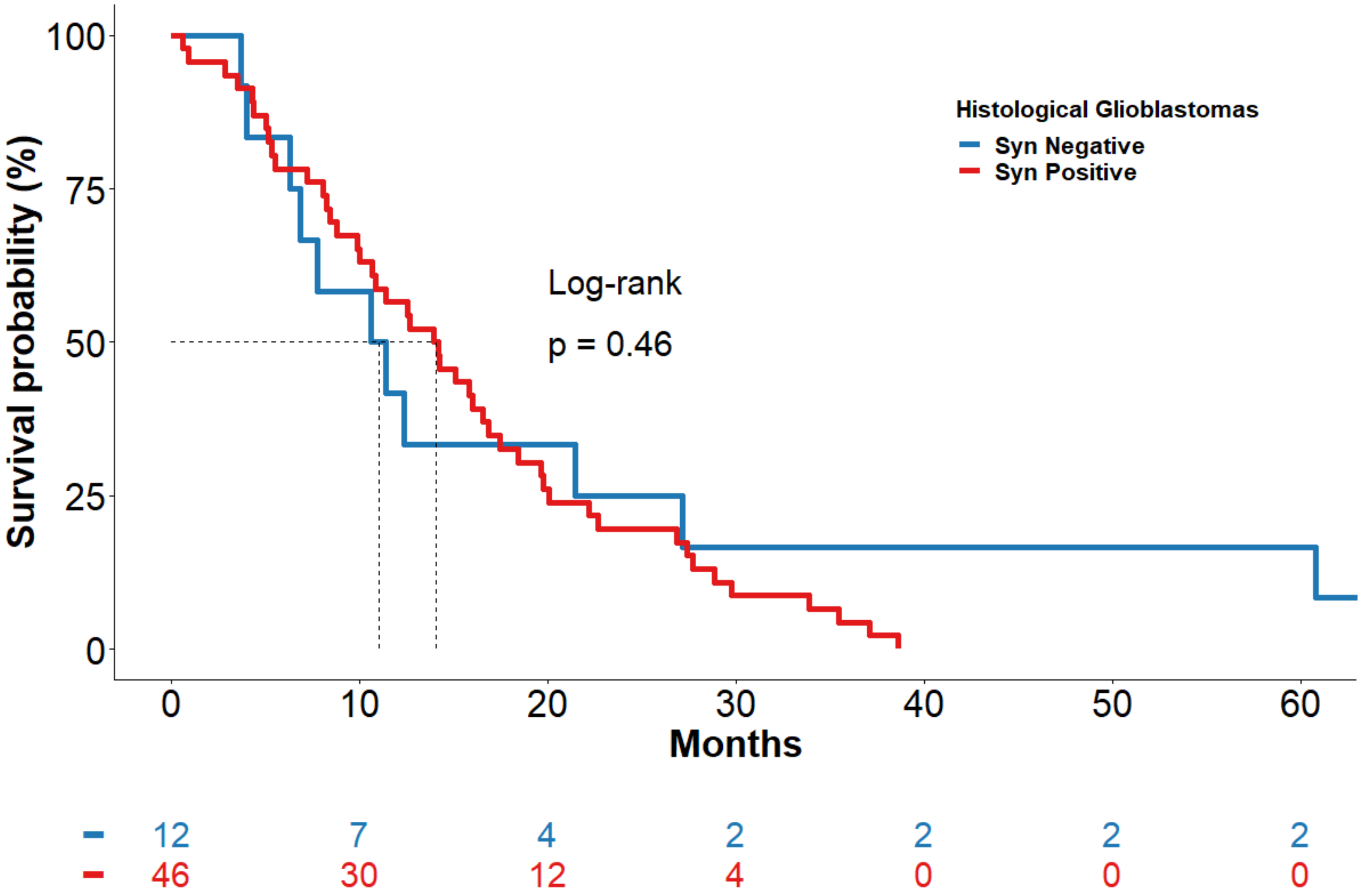

Survival probability (%)
100
75
50
25
0
0
10
20
30
40
50
60
Months
Histological Glioblastomas
Syn Negative
Syn Positive
Log-rank
p = 0.46
12
7
4
2
2
2
2
46
30
12
4
0
0
0

(8)

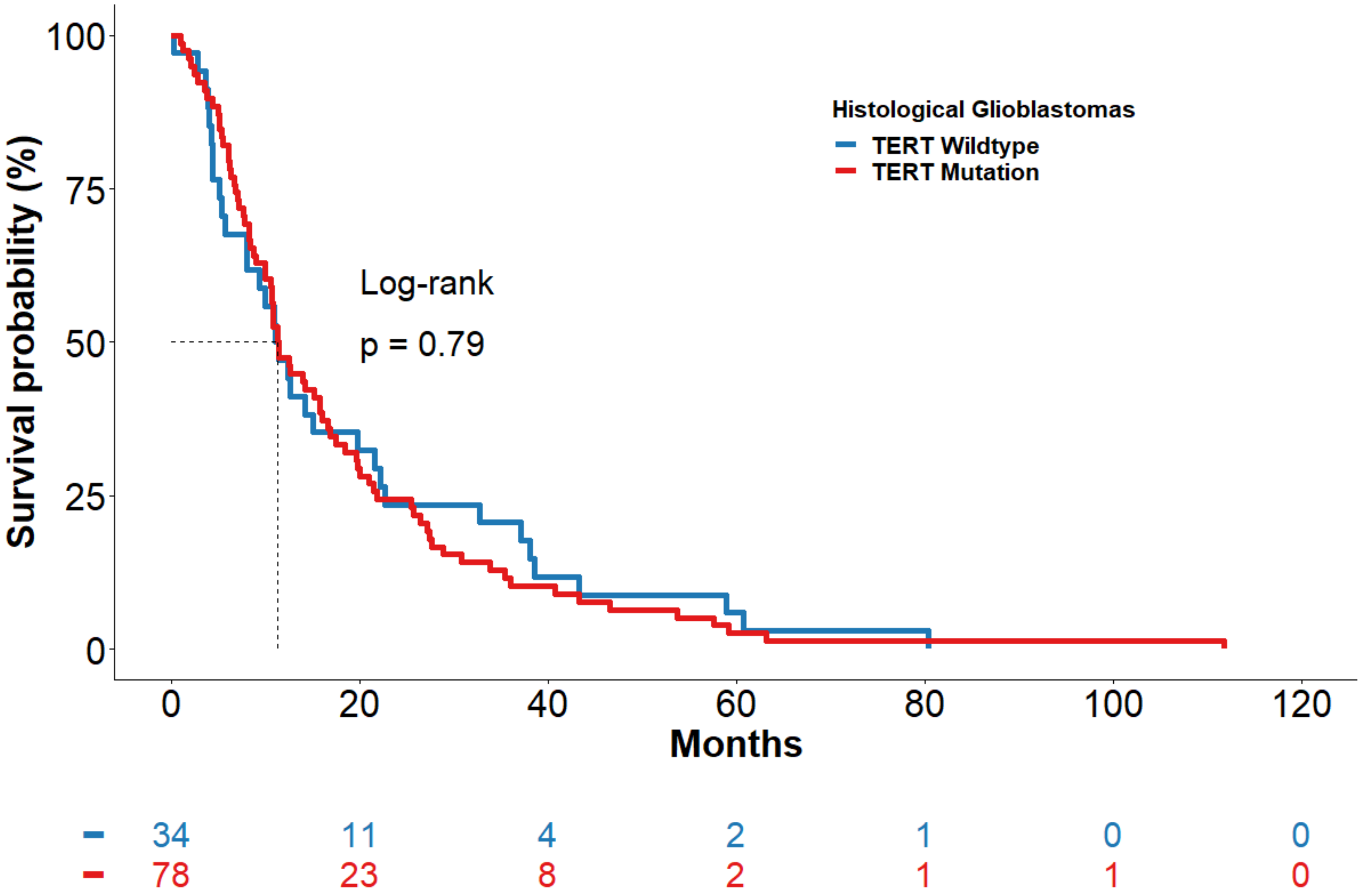
Survival probability (%)
100
75
50
25
0
Log-rank
p = 0.79
Histological Glioblastomas
TERT Wildtype
TERT Mutation
0
20
40
60
80
100
120
Months
34
11
4
2
1
0
0
78
23
8
2
1
1
0

(9)

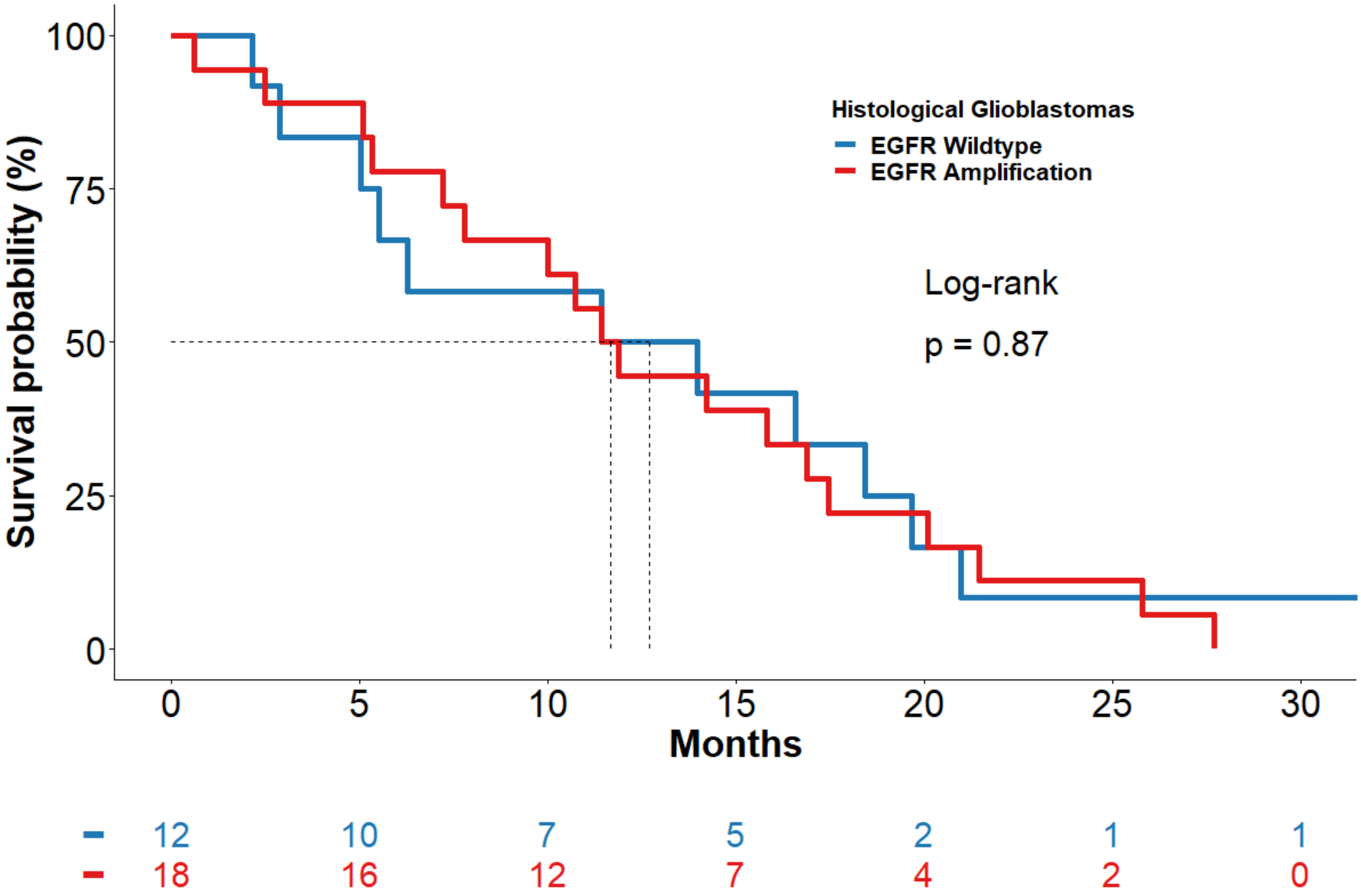
Histological Glioblastomas
EGFR Wildtype
EGFR Amplification
Log-rank
p = 0.87
Survival probability (%)
100
75
50
25
0
0
5
10
15
20
25
30
Months
12 10 7 5 2 1 1
18 16 12 7 4 2 0

(10)

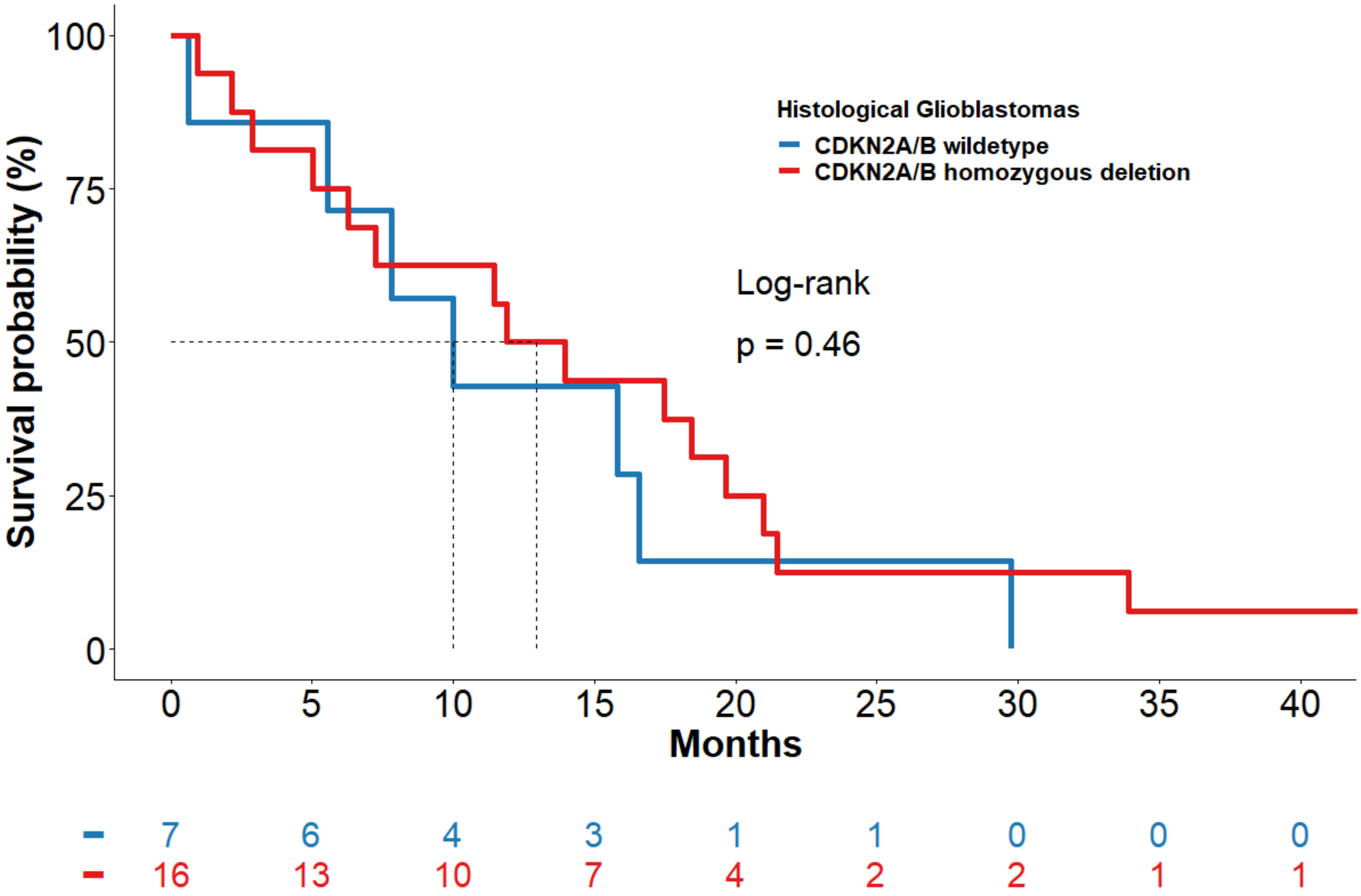

Survival probability (%)
100
75
50
25
0
Histological Glioblastomas
CDKN2A/B wildetype
CDKN2A/B homozygous deletion
Log-rank
p = 0.46
0
5
10
15
20
25
30
35
40
Months
7 6 4 3 1 1 0 0 0
16 13 10 7 4 2 2 1 1

(11)

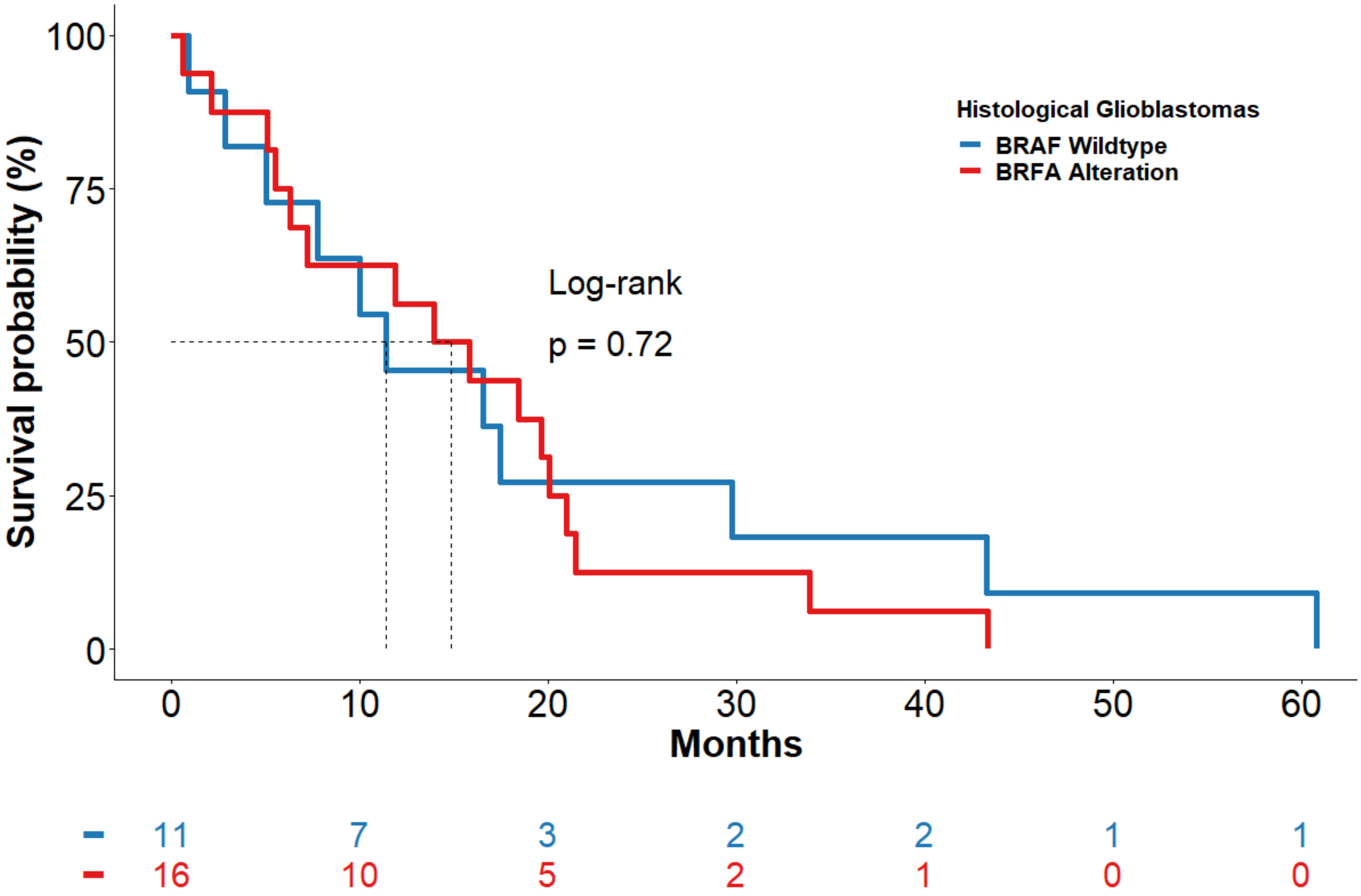

Survival probability (%)
100
75
50
25
0
Log-rank
p = 0.72
Histological Glioblastomas
BRAF Wildtype
BRFA Alteration
0
10
20
30
40
50
60
Months
11 7 3 2 2 1 1
16 10 5 2 1 0 0

(12)

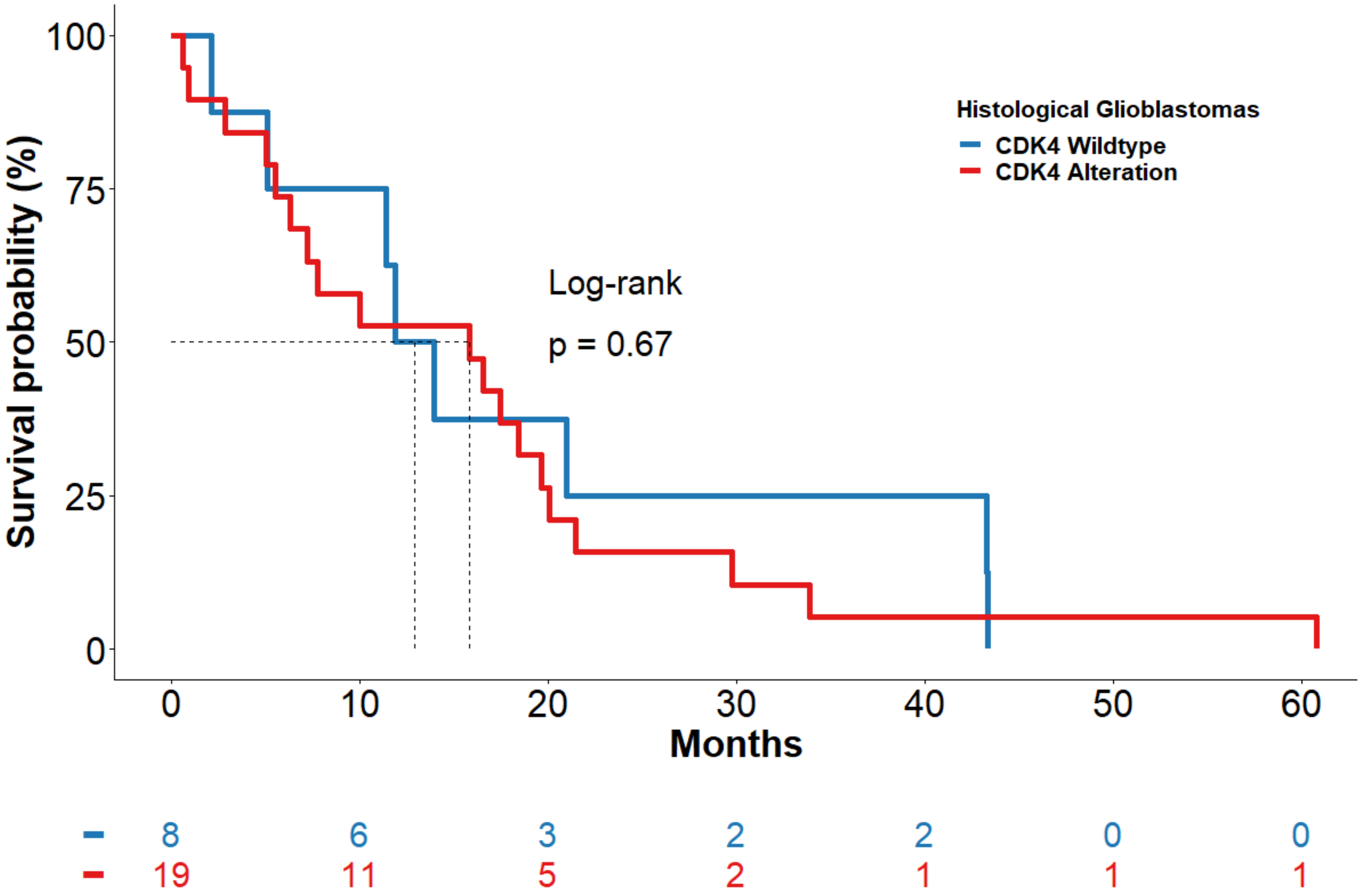

Survival probability (%)
100
75
50
25
0
Log-rank
p = 0.67
Histological Glioblastomas
CDK4 Wildtype
CDK4 Alteration
0
10
20
30
40
50
60
Months
8 6 3 2 2 0 0
19 11 5 2 1 1 1

(13)

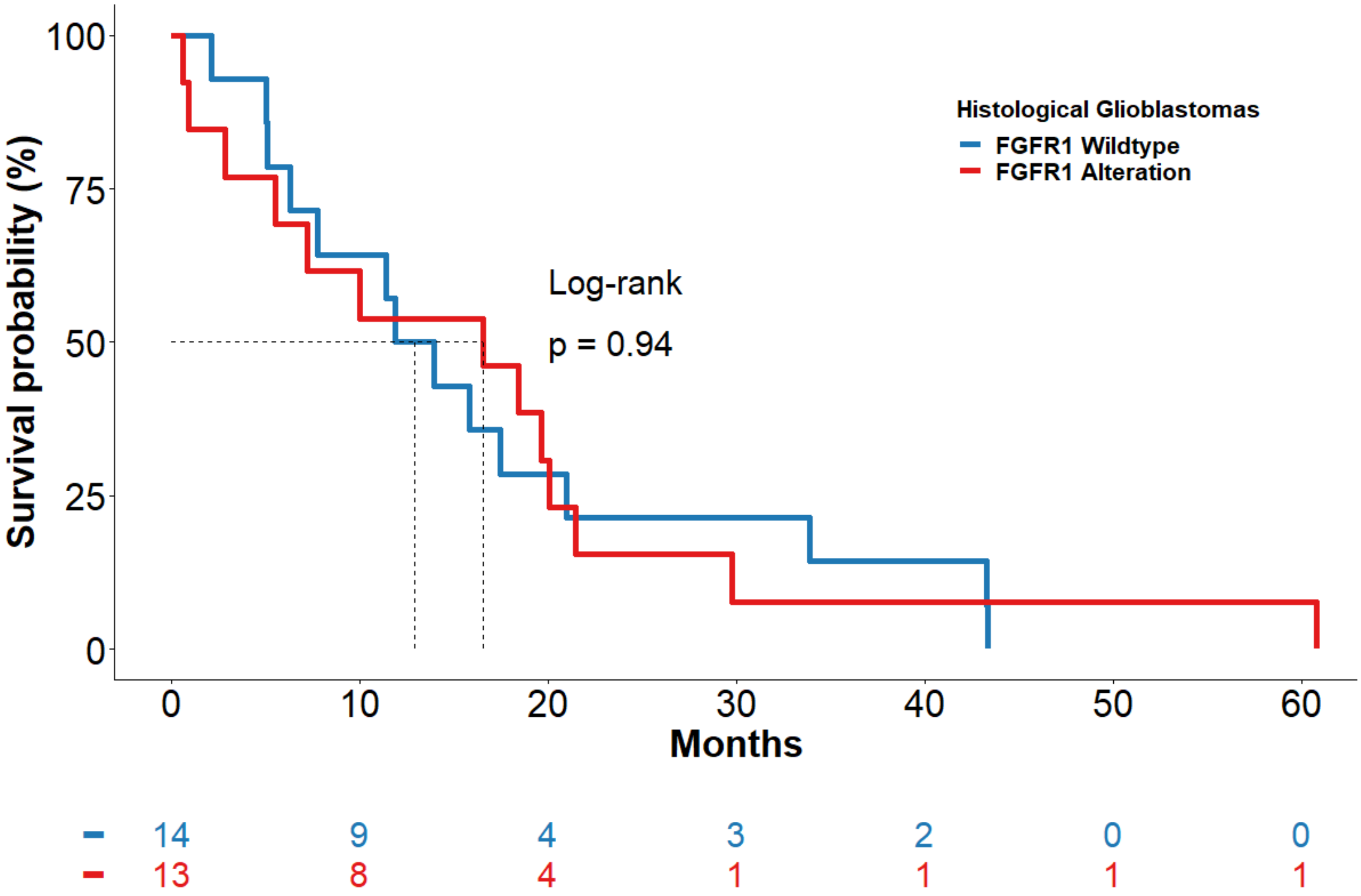
Survival probability (%)
100
75
50
25
0
Log-rank
p = 0.94
Histological Glioblastomas
FGFR1 Wildtype
FGFR1 Alteration
0
10
20
30
40
50
60
Months
14
9
4
3
2
0
0
13
8
4
1
1
1
1

(14)

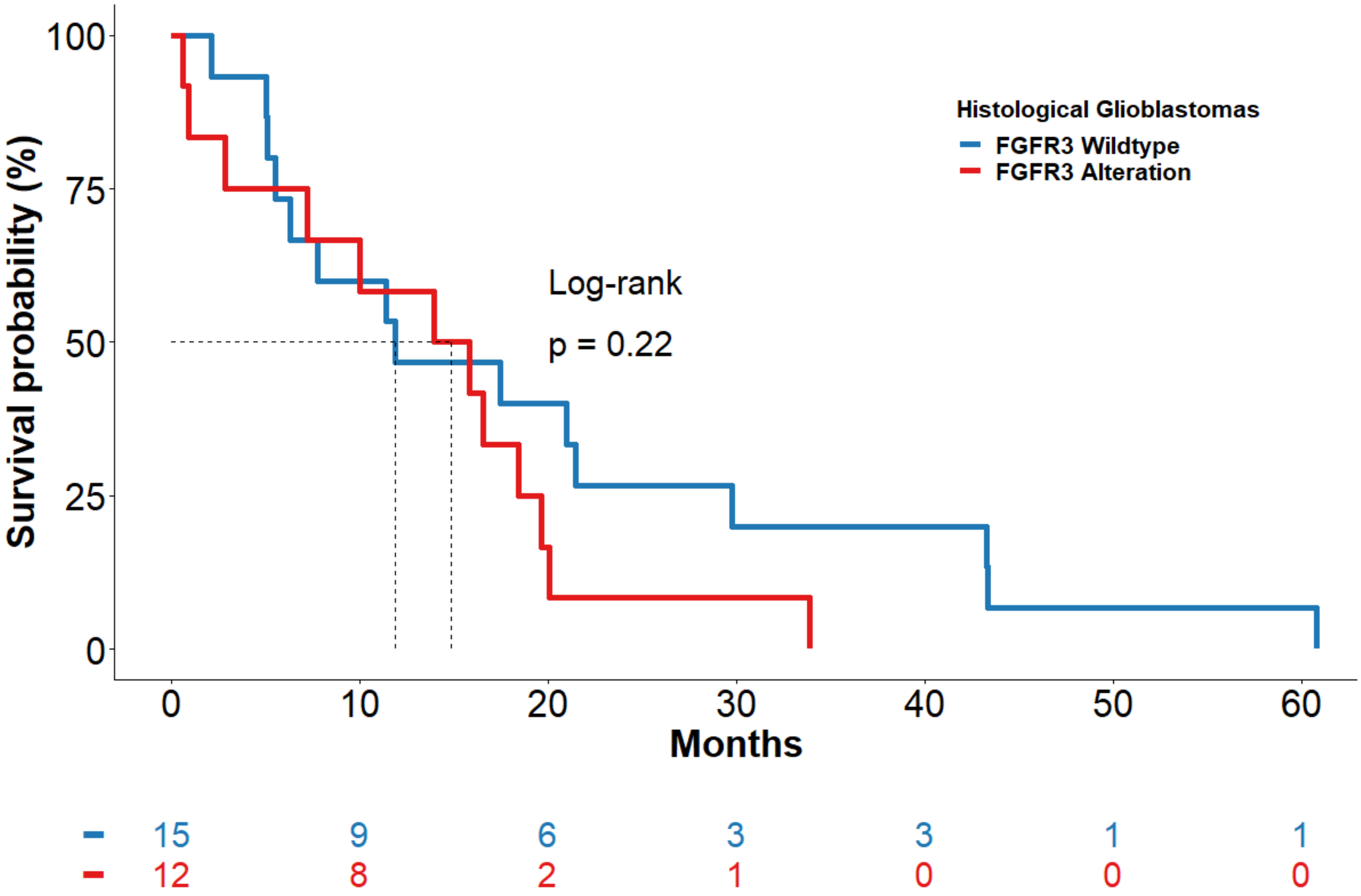

Survival probability (%)
100
75
50
25
0
Histological Glioblastomas
FGFR3 Wildtype
FGFR3 Alteration
Log-rank
p = 0.22
0
10
20
30
40
50
60
Months
15 9 6 3 3 1 1
12 8 2 1 0 0 0

(15)

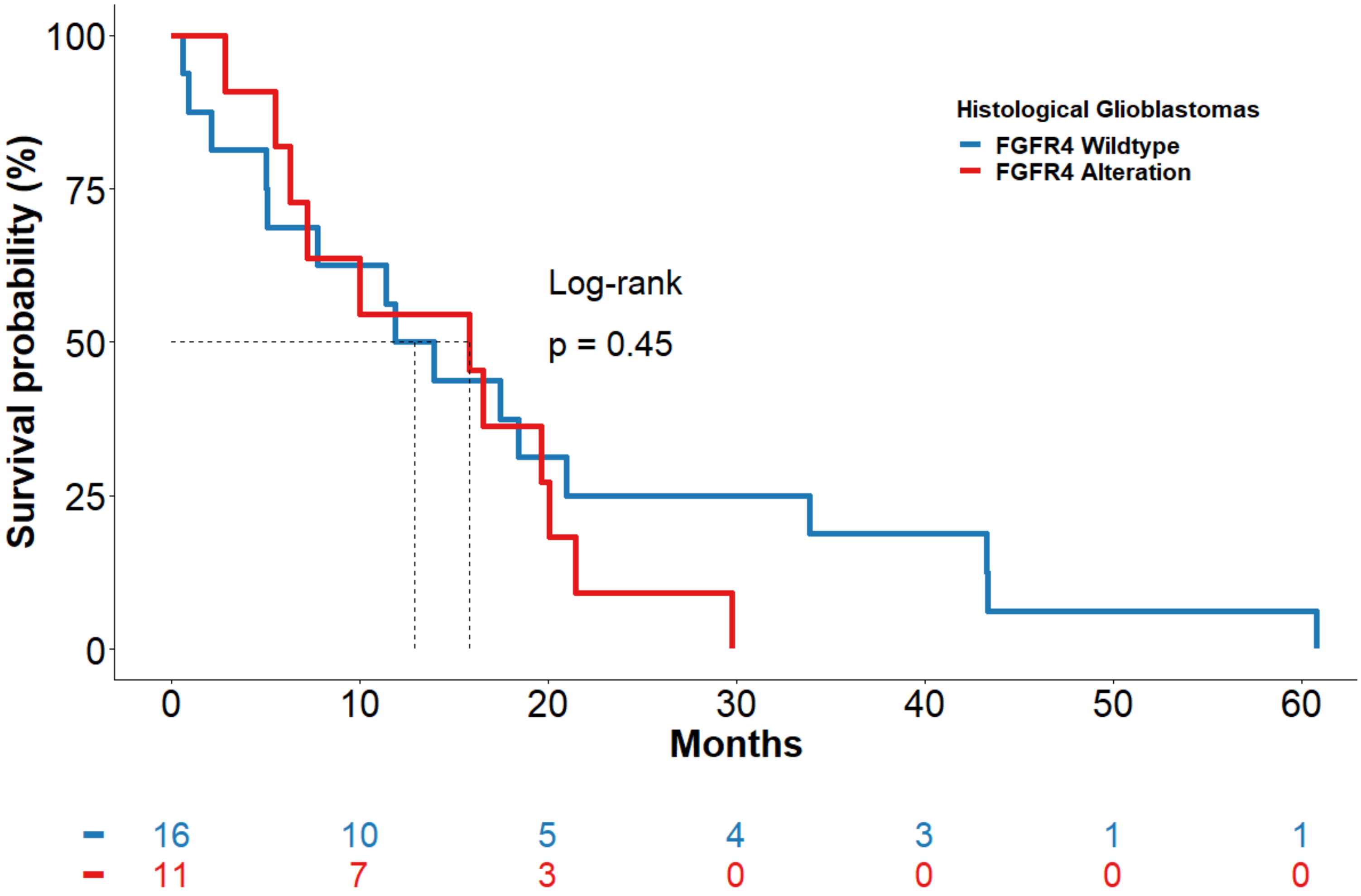
Survival probability (%)
100
75
50
25
0
Log-rank
p = 0.45
Histological Glioblastomas
FGFR4 Wildtype
FGFR4 Alteration
0
10
20
30
40
50
60
Months
16
10
5
4
3
1
1
11
7
3
0
0
0
0

(16)

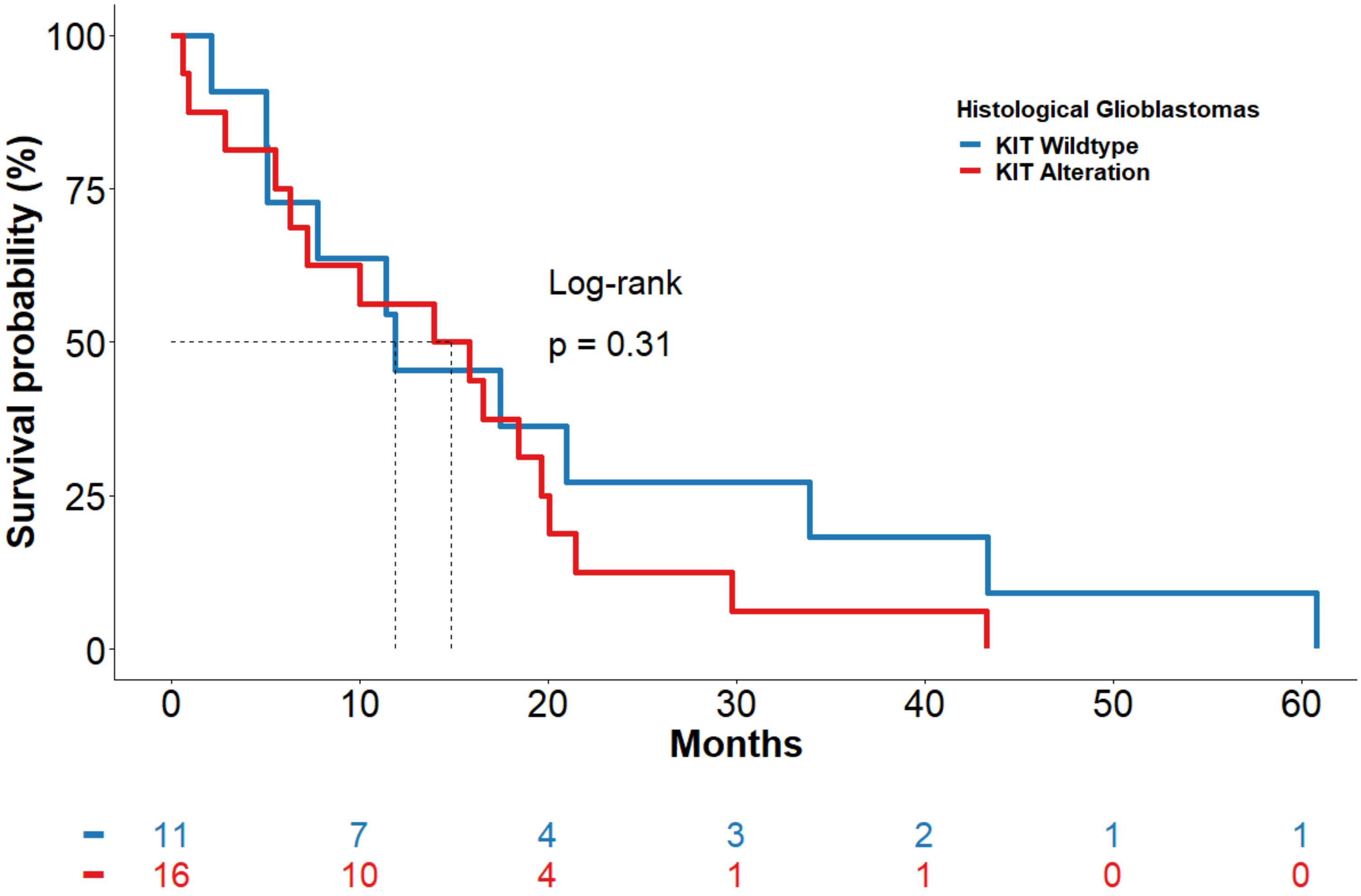

Survival probability (%)
100
75
50
25
0
Log-rank
p = 0.31
Histological Glioblastomas
KIT Wildtype
KIT Alteration
0
10
20
30
40
50
60
Months
11
7
4
3
2
1
1
16
10
4
1
1
0
0

(17)

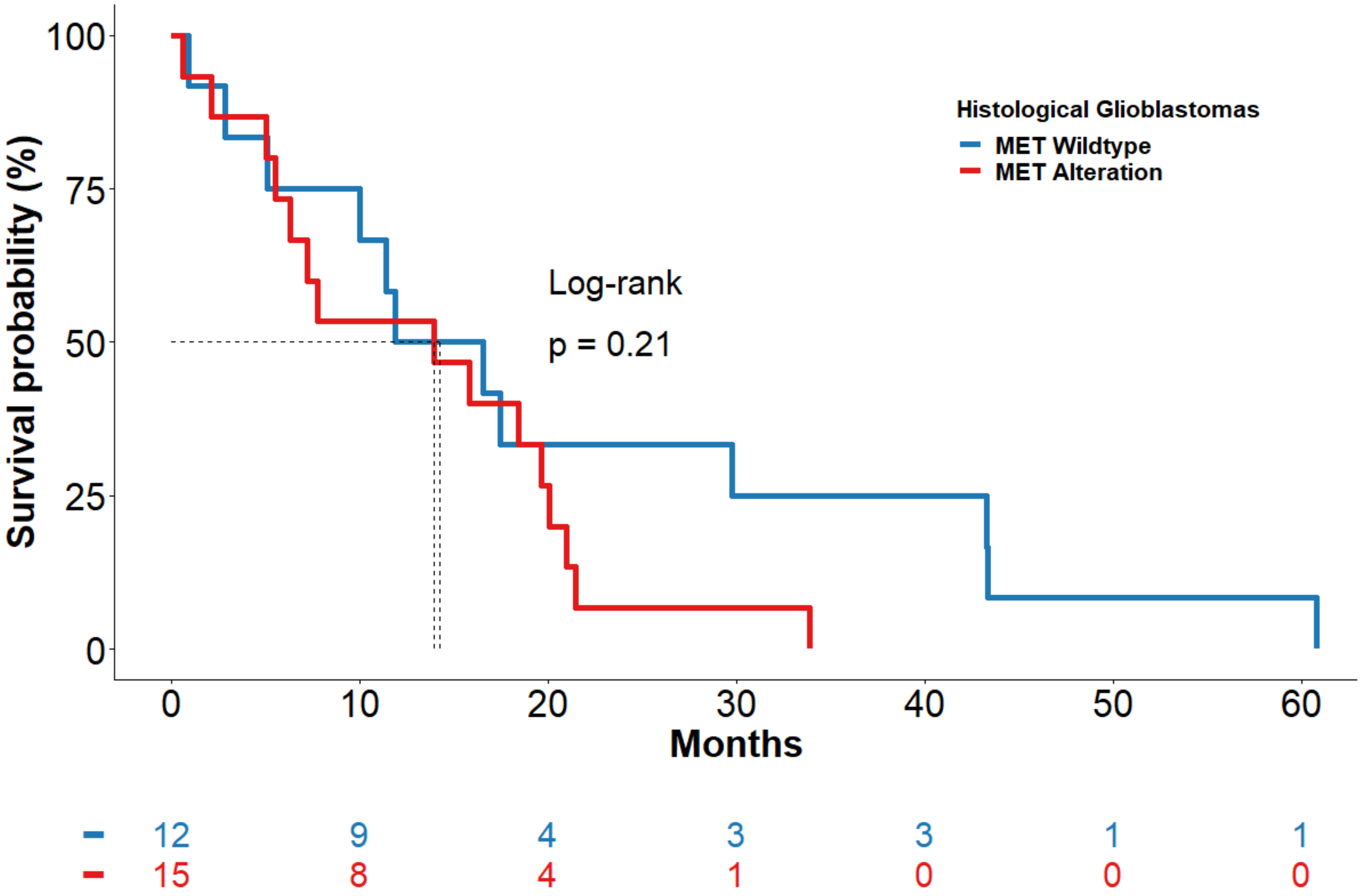

Survival probability (%)
100
75
50
25
0
Histological Glioblastomas
MET Wildtype
MET Alteration
Log-rank
p = 0.21
0
10
20
30
40
50
60
Months
12
9
4
3
3
1
1
15
8
4
1
0
0
0

(18)

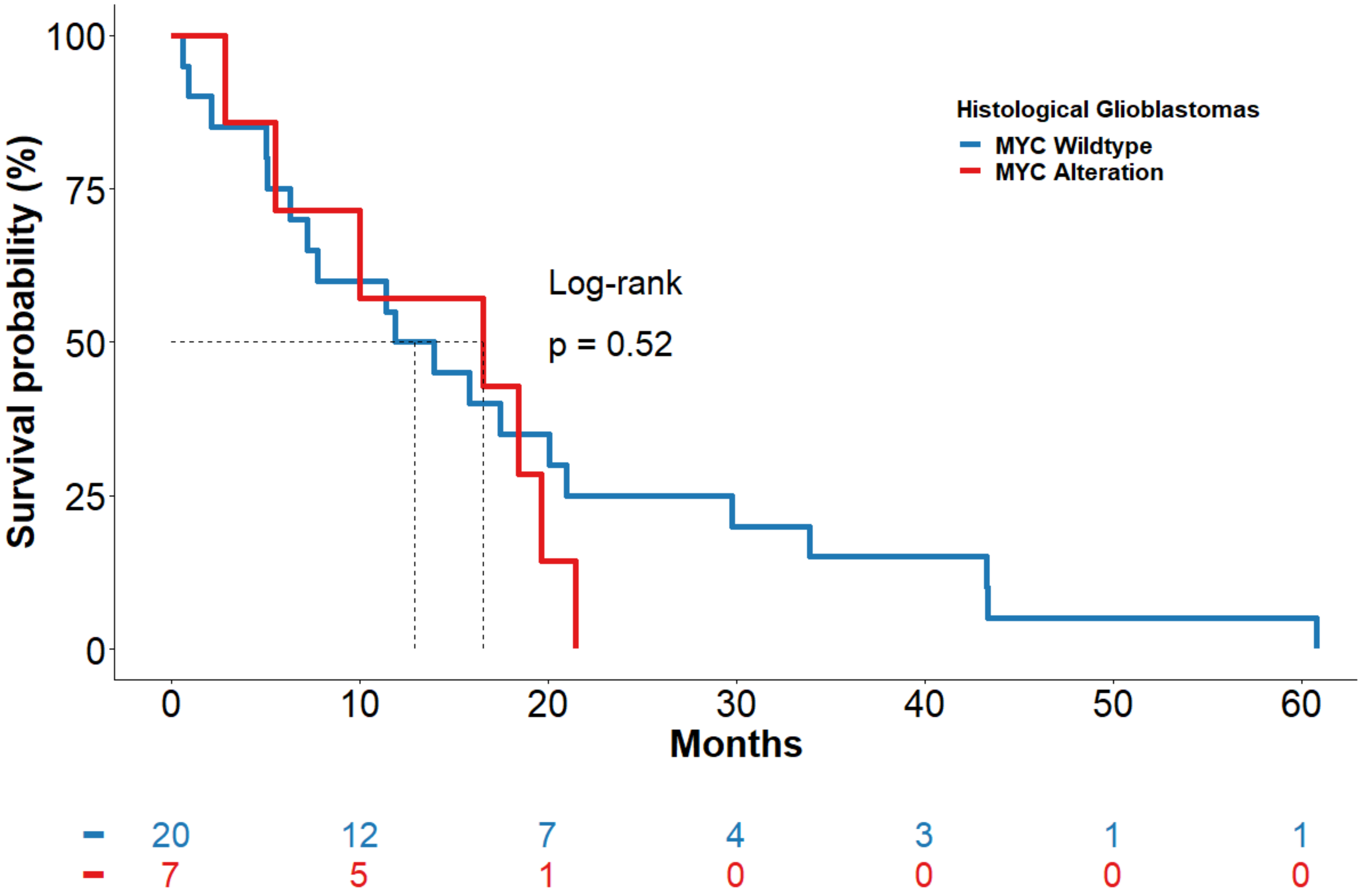

Survival probability (%)
100
75
50
25
0
Log-rank
p = 0.52
Histological Glioblastomas
MYC Wildtype
MYC Alteration
0
10
20
30
40
50
60
Months
20 12 7 4 3 1 1
7 5 1 0 0 0 0

(19)

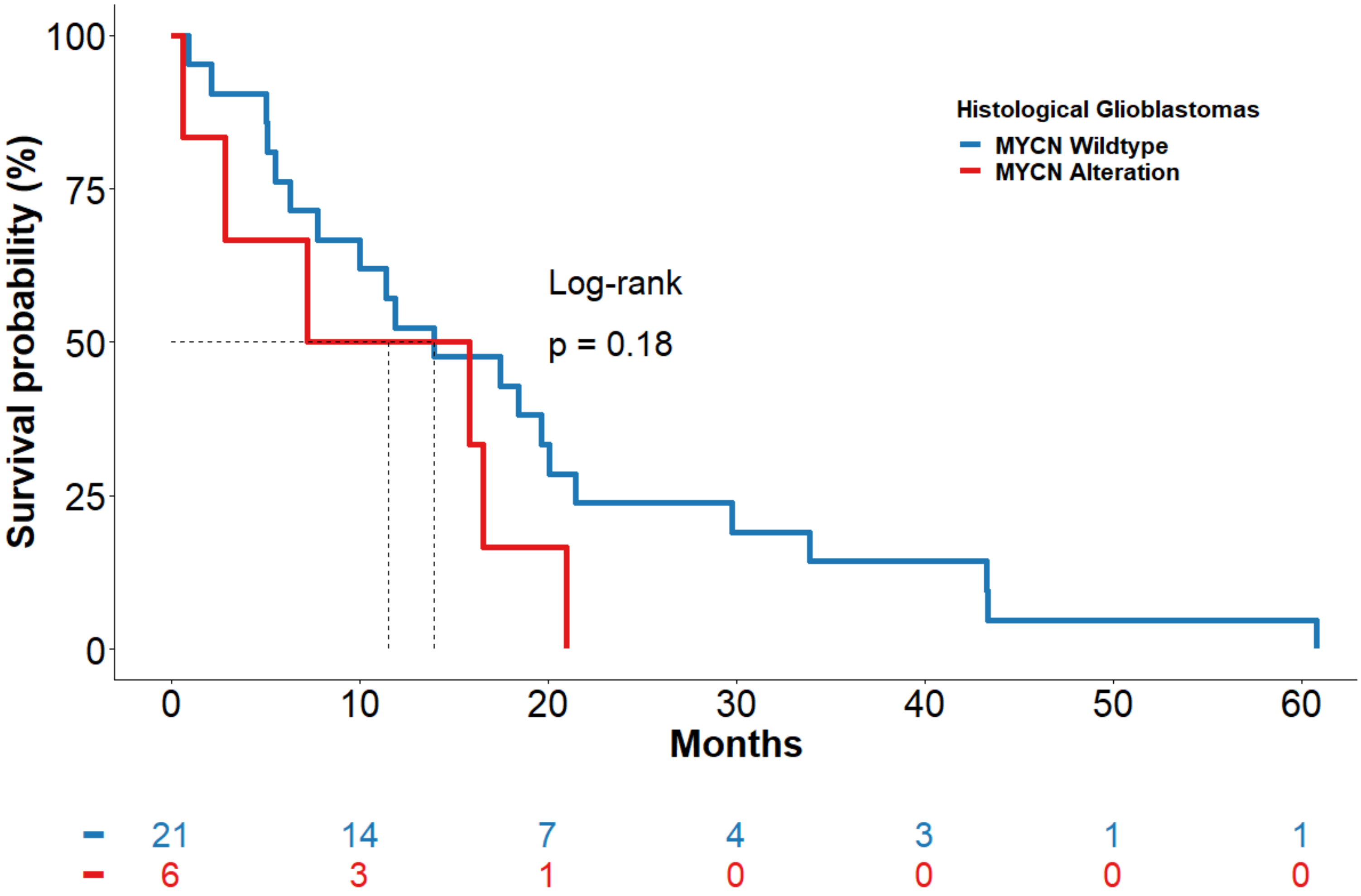

Survival probability (%)
100
75
50
25
0
Log-rank
p = 0.18
Histological Glioblastomas
MYCN Wildtype
MYCN Alteration
0
10
20
30
40
50
60
Months
21
14
7
4
3
1
1
6
3
1
0
0
0
0

(20)

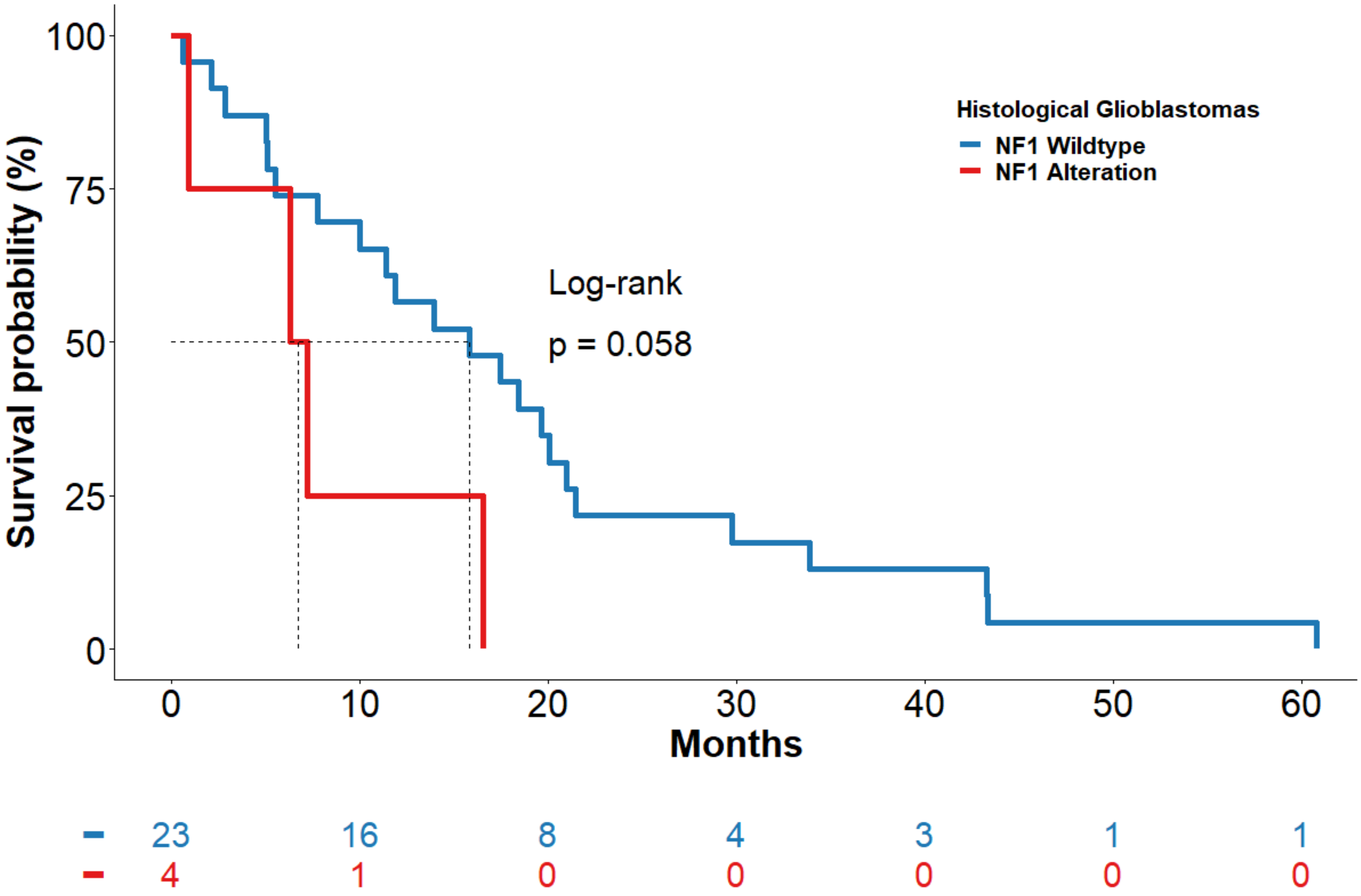
Survival probability (%)
100
75
50
25
0
Histological Glioblastomas
NF1 Wildtype
NF1 Alteration
Log-rank
p = 0.058
0
10
20
30
40
50
60
Months
23 16 8 4 3 1 1
4 1 0 0 0 0 0

(21)

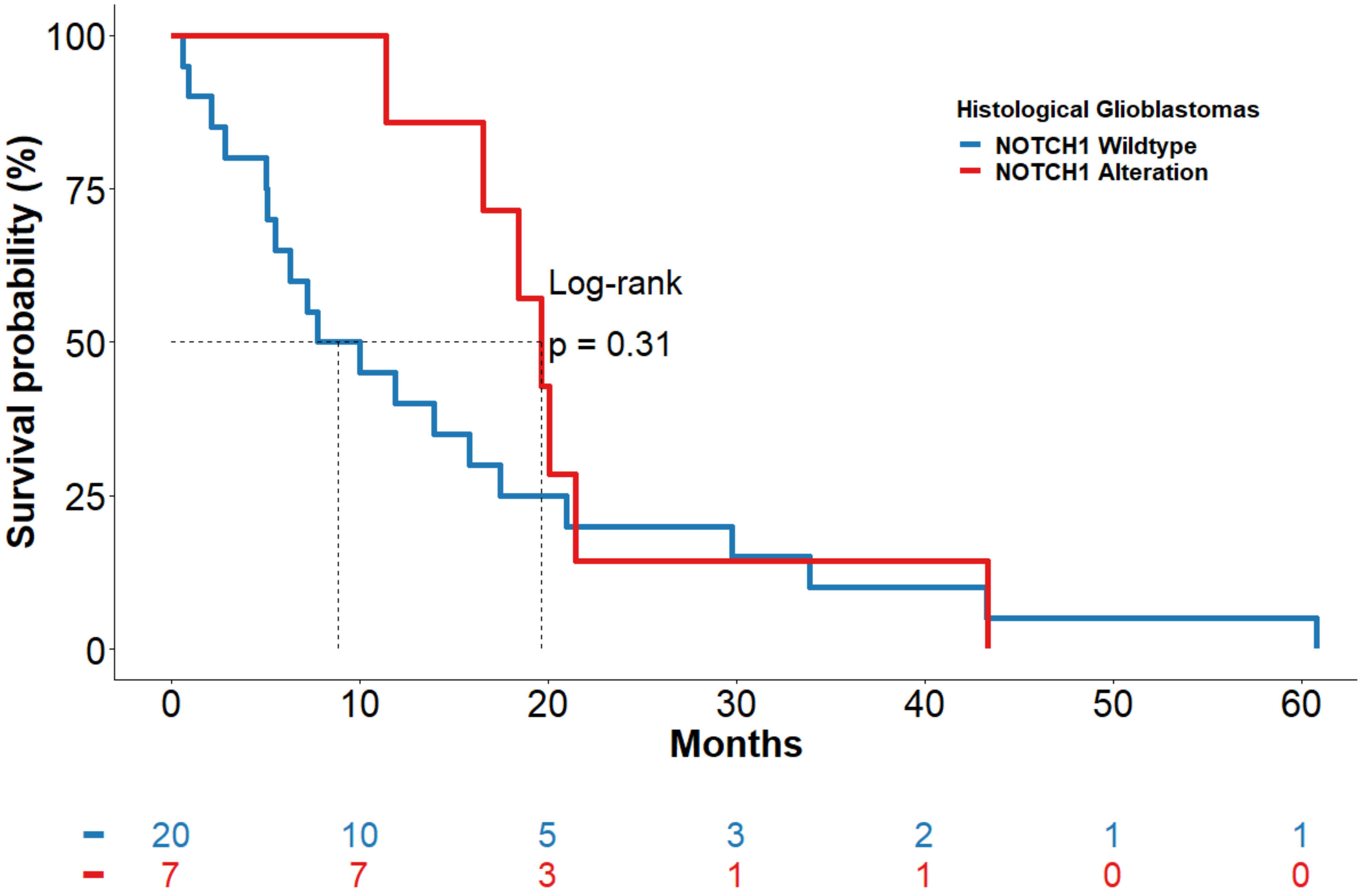

Survival probability (%)
100
75
50
25
0
Log-rank
p = 0.31
Histological Glioblastomas
NOTCH1 Wildtype
NOTCH1 Alteration
0
10
20
30
40
50
60
Months
20
10
5
3
2
1
1
7
7
3
1
1
0
0

(22)

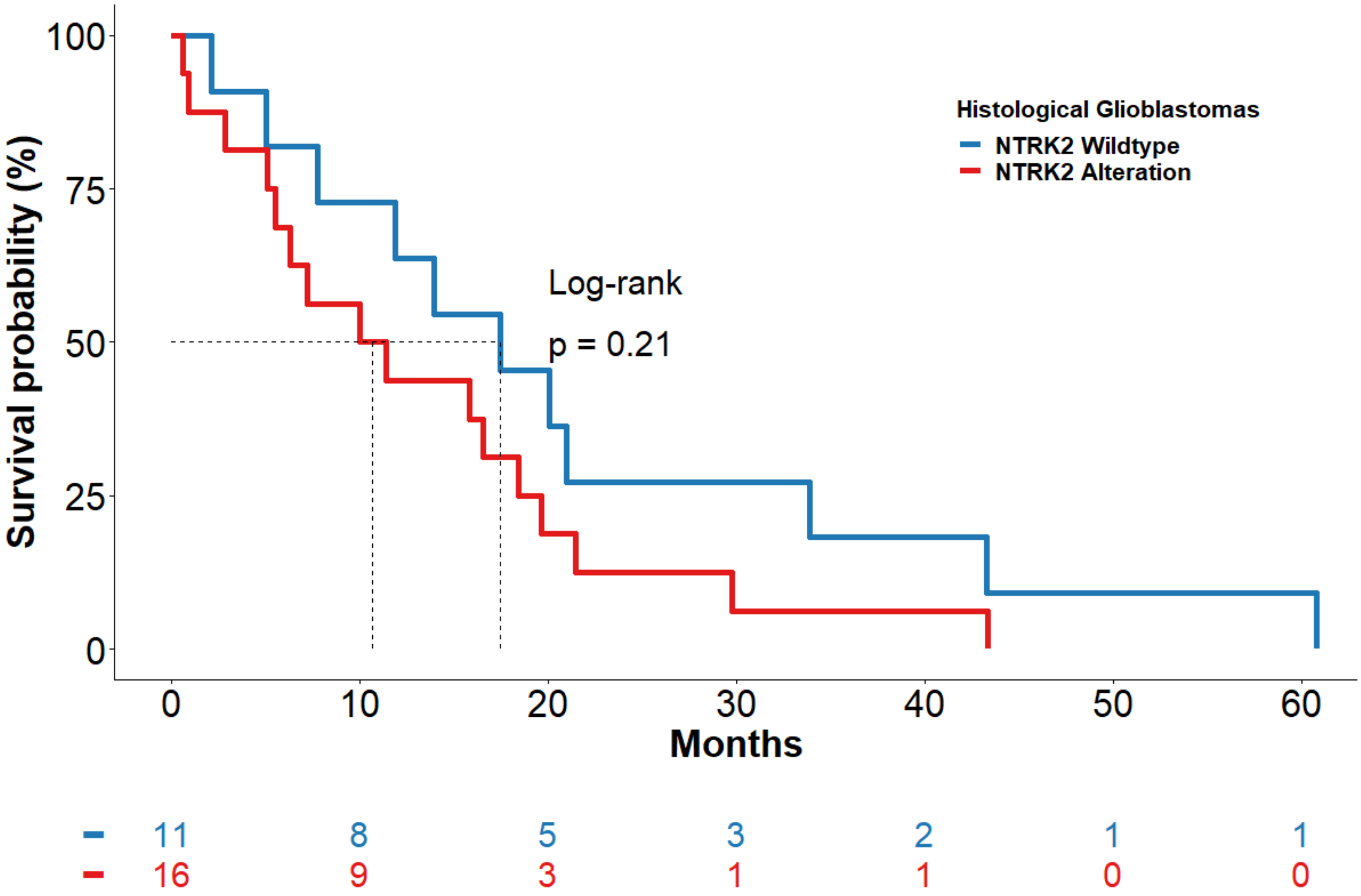
Survival probability (%)
100
75
50
25
0
Log-rank
p = 0.21
Histological Glioblastomas
NTRK2 Wildtype
NTRK2 Alteration
0
10
20
30
40
50
60
Months
11 8 5 3 2 1 1
16 9 3 1 1 0 0

(23)

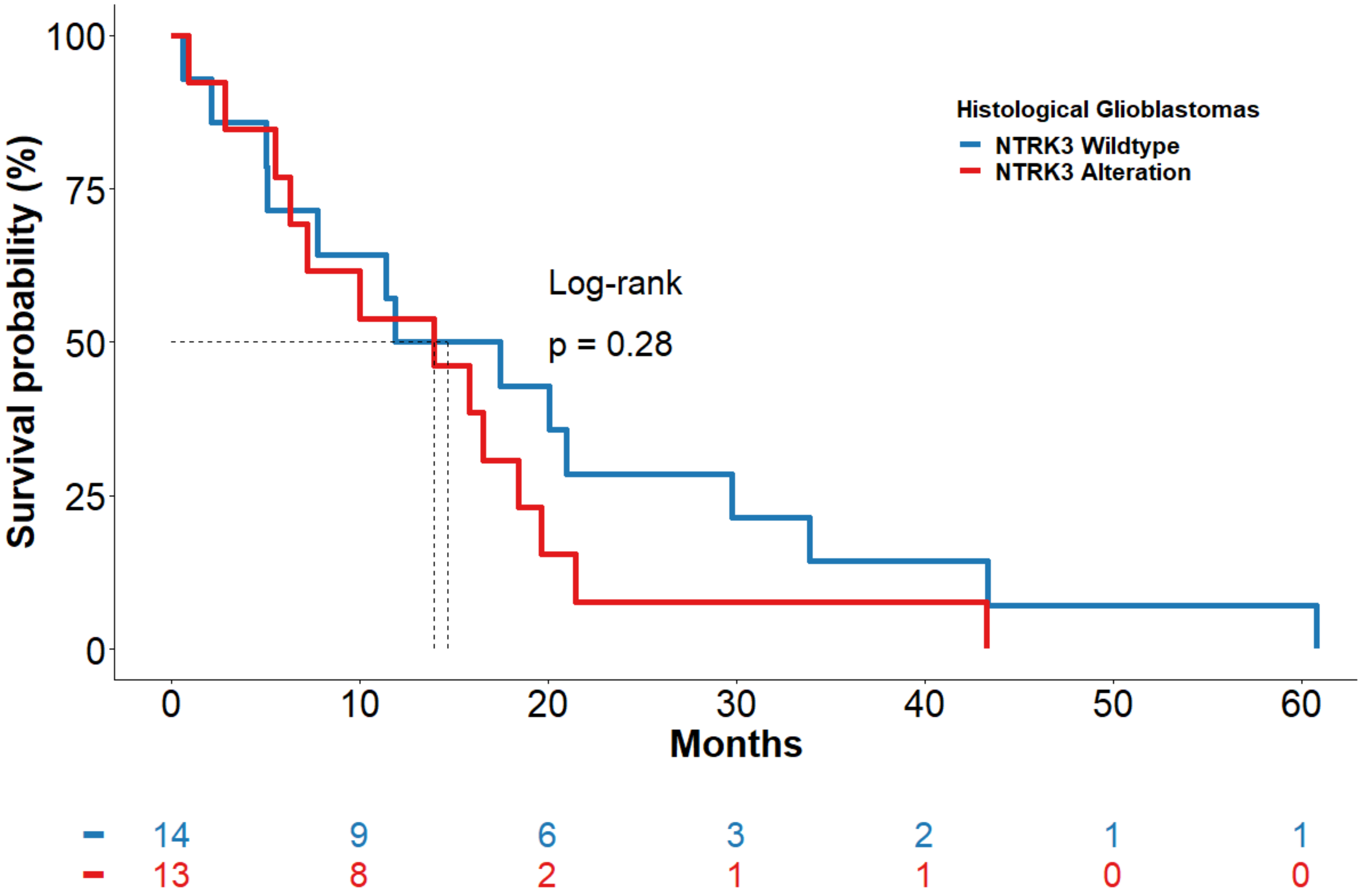

Histological Glioblastomas
NTRK3 Wildtype
NTRK3 Alteration
Survival probability (%)
100
75
50
25
0
Log-rank
p = 0.28
0
10
20
30
40
50
60
Months
14 9 6 3 2 1 1
13 8 2 1 1 0 0

(24)

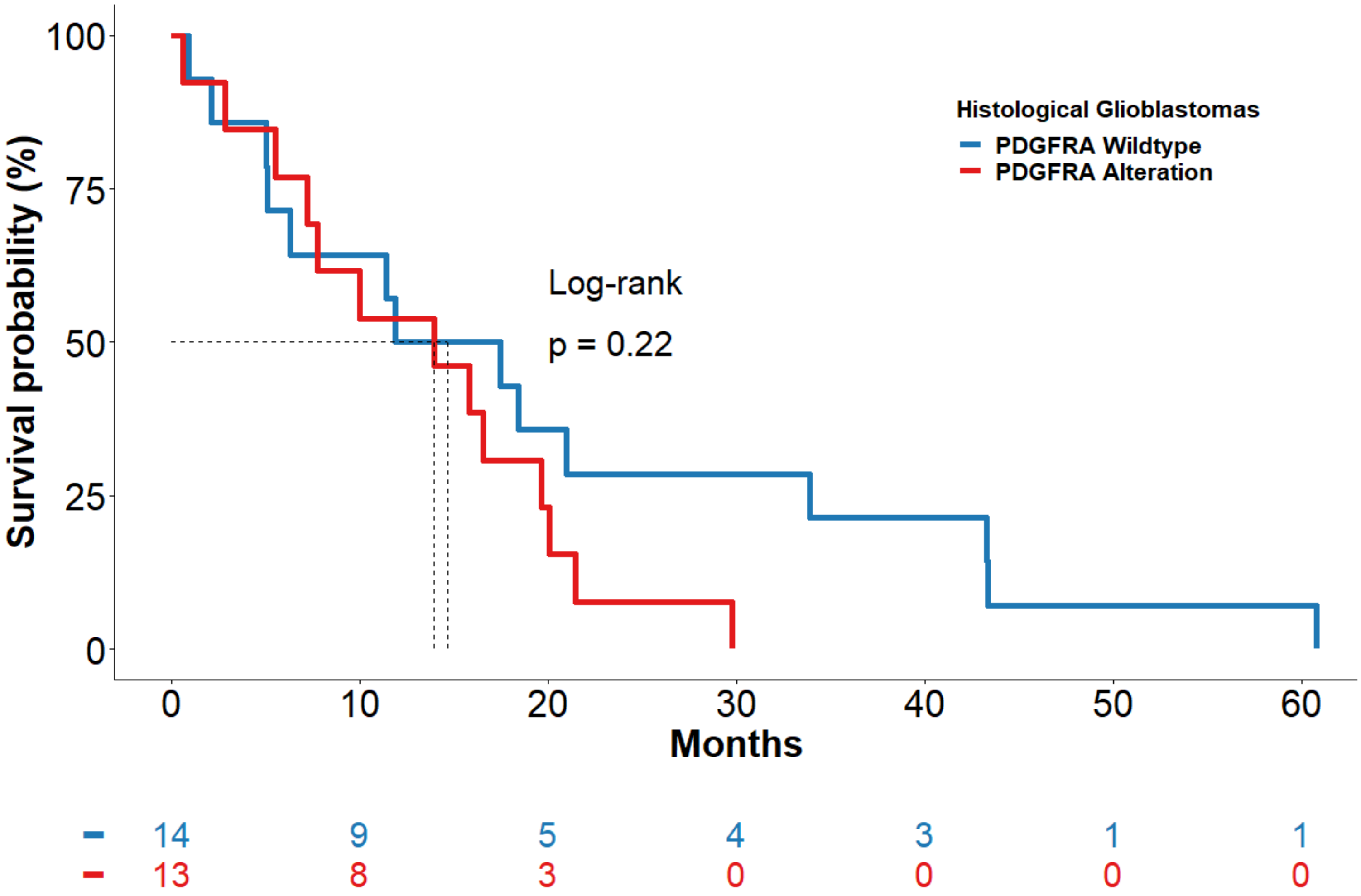

Survival probability (%)
100
75
50
25
0
Log-rank
p = 0.22
Histological Glioblastomas
PDGFRA Wildtype
PDGFRA Alteration
0
10
20
30
40
50
60
Months
14
9
5
4
3
1
1
13
8
3
0
0
0
0

(25)

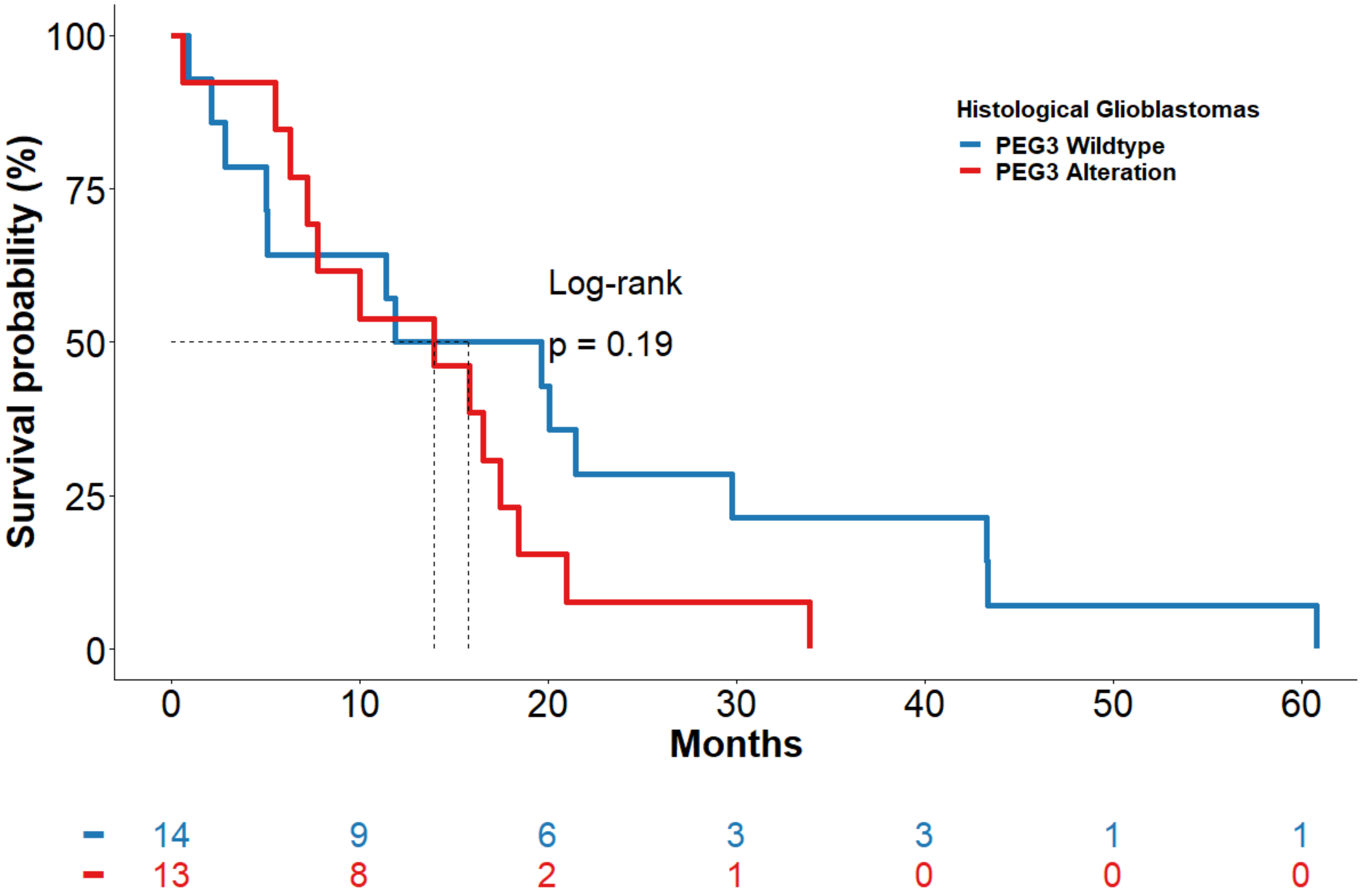

Survival probability (%)
100
75
50
25
0
0
10
20
30
40
50
60
Months
Log-rank
p = 0.19
Histological Glioblastomas
PEG3 Wildtype
PEG3 Alteration
14 9 6 3 3 1 1
13 8 2 1 0 0 0

(26)

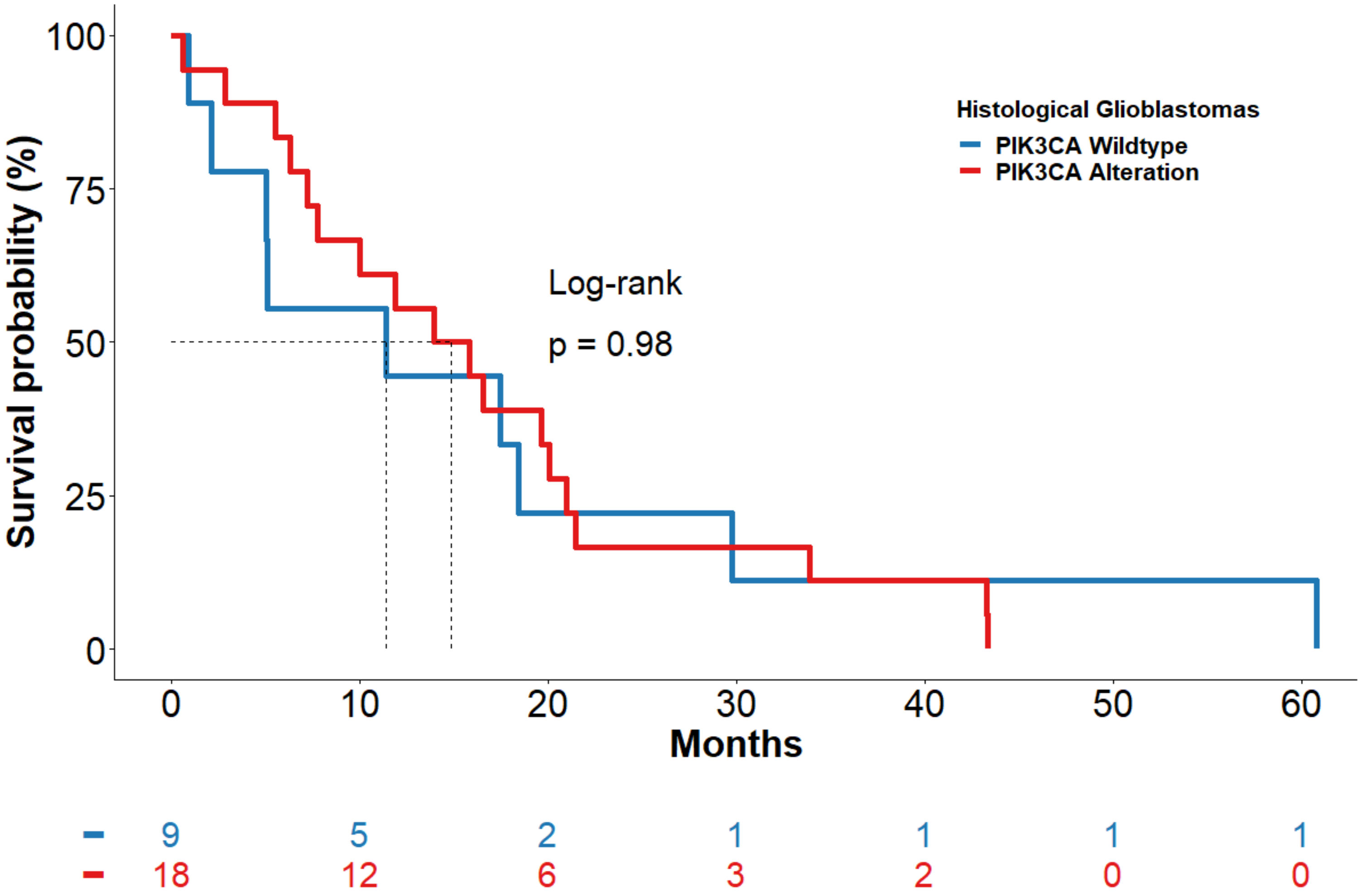

Survival probability (%)
100
75
50
25
0
Log-rank
p = 0.98
Histological Glioblastomas
PIK3CA Wildtype
PIK3CA Alteration
0
10
20
30
40
50
60
Months
9 5 2 1 1 1 1
18 12 6 3 2 0 0

(27)

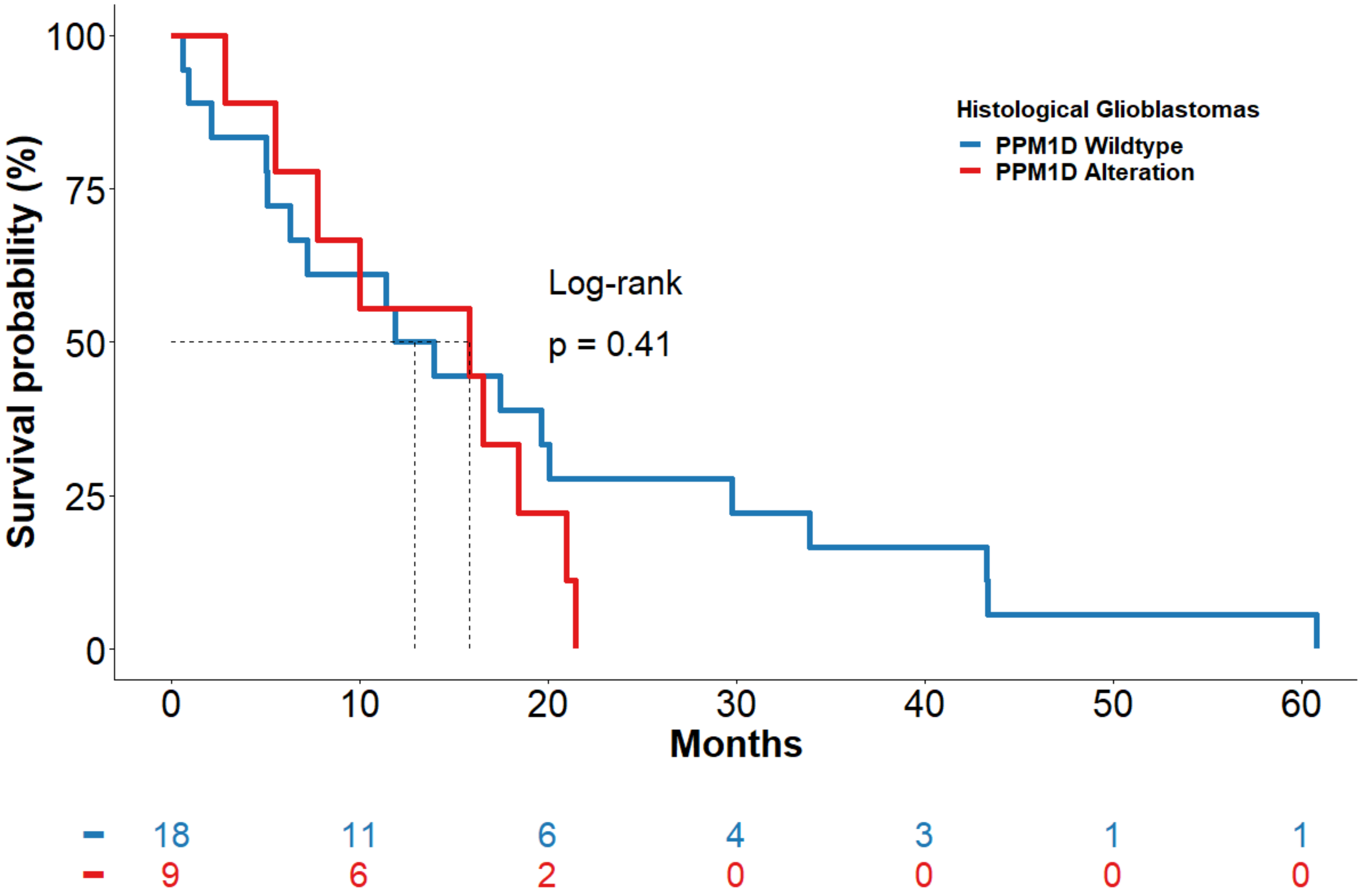
Survival probability (%)
100
75
50
25
0
Log-rank
p = 0.41
Histological Glioblastomas
PPM1D Wildtype
PPM1D Alteration
0
10
20
30
40
50
60
Months
18 11 6 4 3 1 1
9 6 2 0 0 0 0

(28)

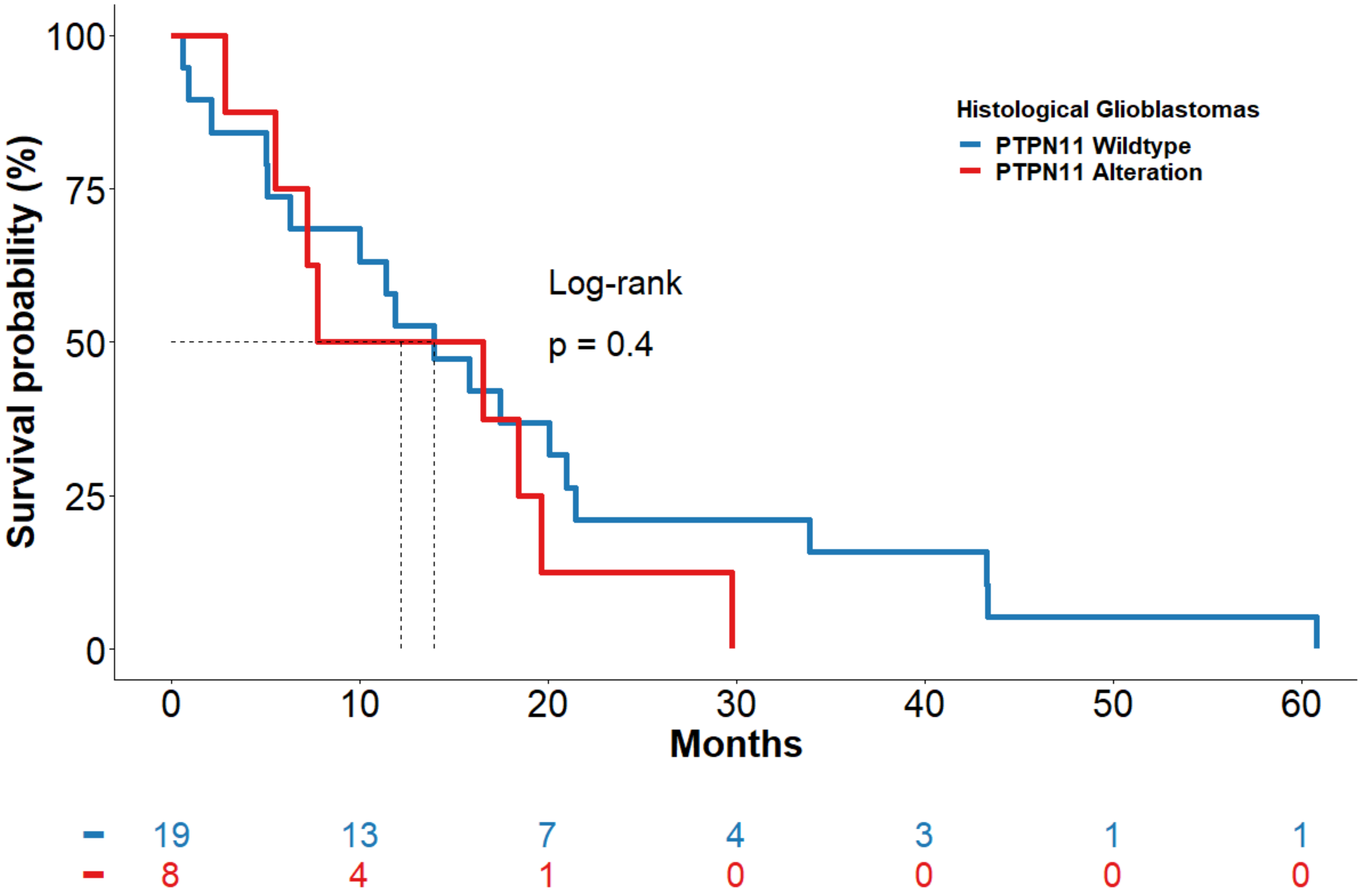

Histological Glioblastomas
PTPN11 Wildtype
PTPN11 Alteration
Survival probability (%)
100
75
50
25
0
Log-rank
p = 0.4
0
10
20
30
40
50
60
Months
19
13
7
4
3
1
1
8
4
1
0
0
0
0

(29)

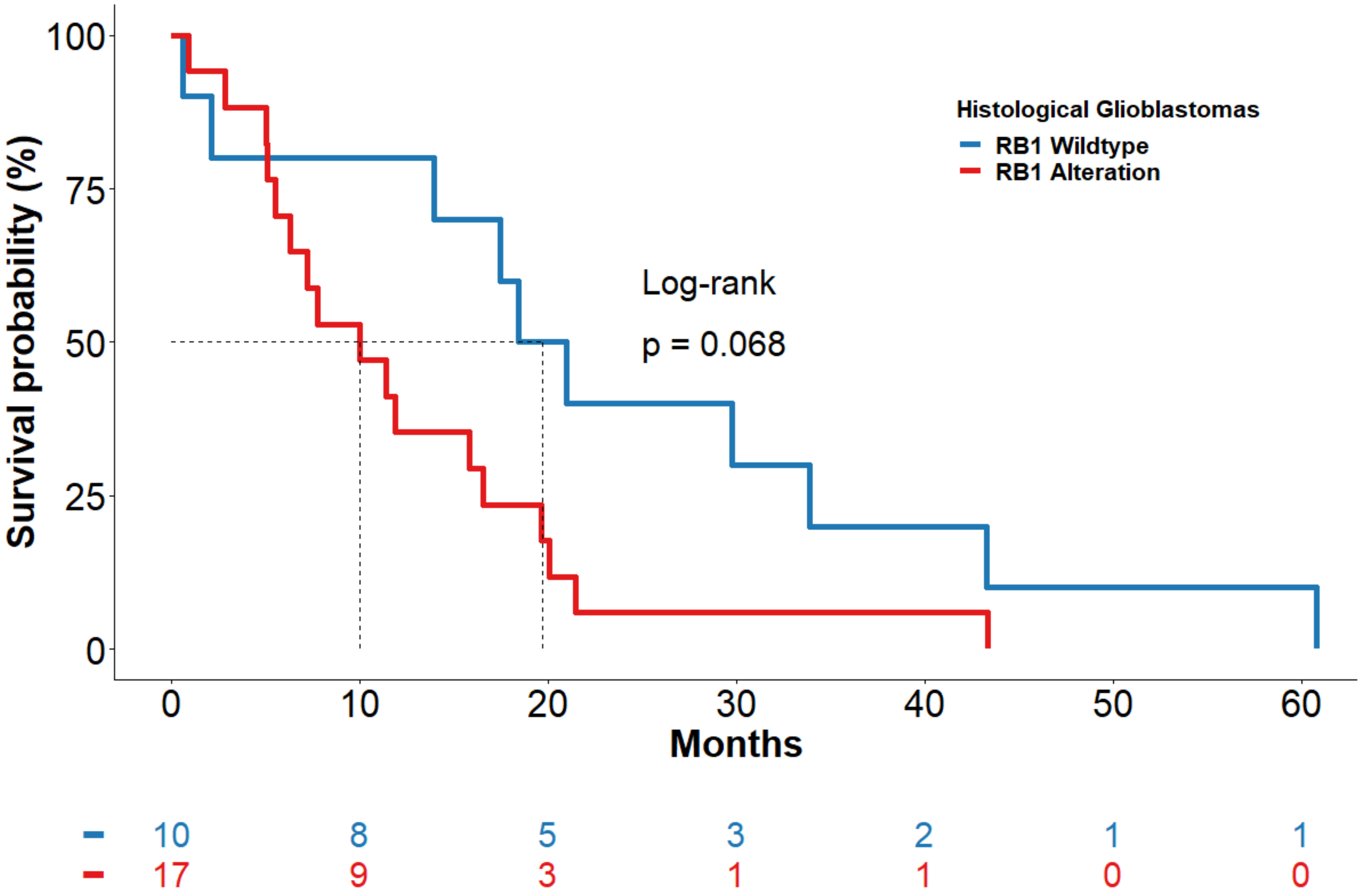

Survival probability (%)
100
75
50
25
0
Log-rank
p = 0.068
Histological Glioblastomas
RB1 Wildtype
RB1 Alteration
0
10
20
30
40
50
60
Months
10 8 5 3 2 1 1
17 9 3 1 1 0 0

(30)

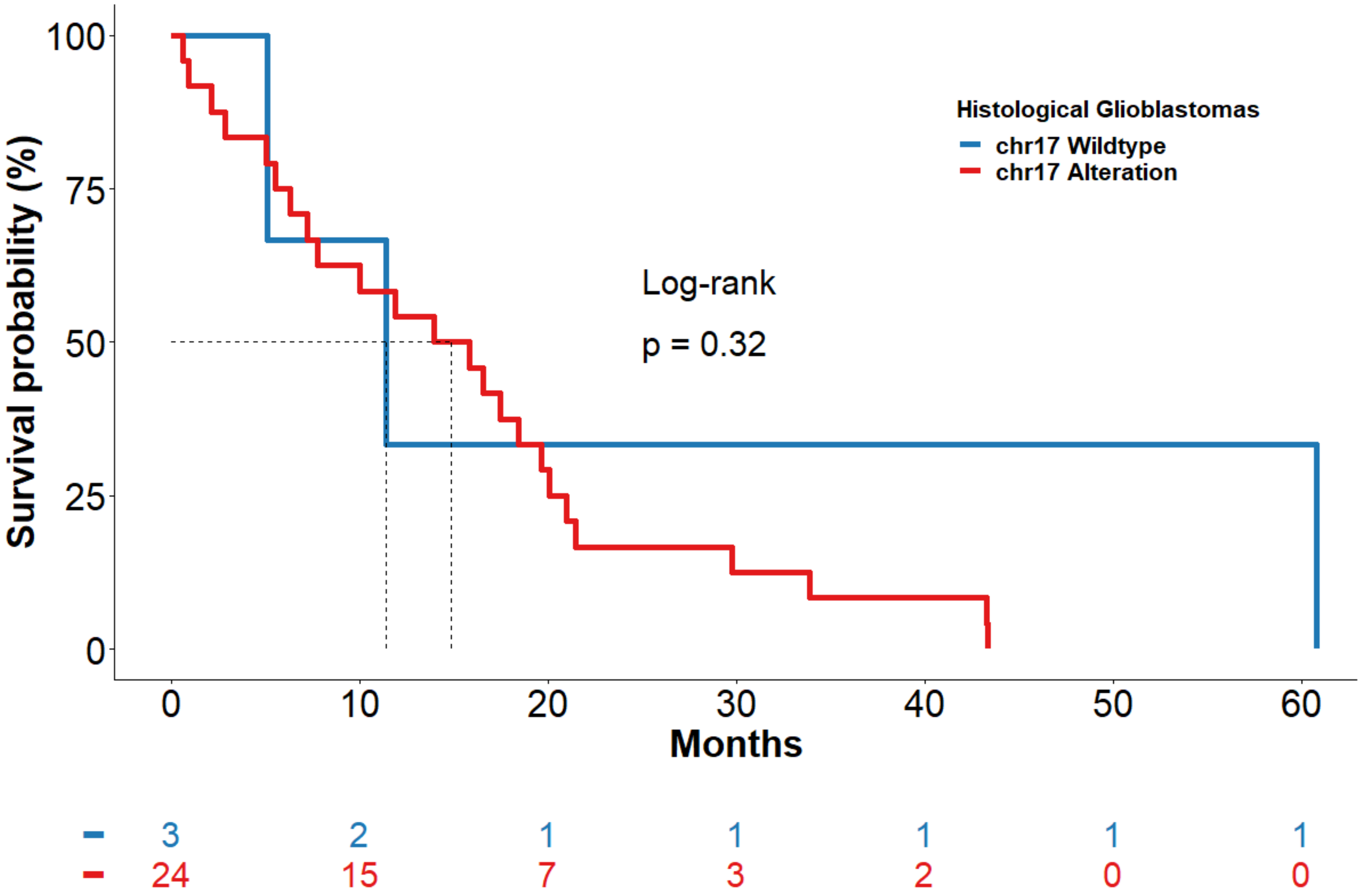

Survival probability (%)
100
75
50
25
0
Histological Glioblastomas
chr17 Wildtype
chr17 Alteration
Log-rank
p = 0.32
0
10
20
30
40
50
60
Months
3
2
1
1
1
1
1
24
15
7
3
2
0
0
