# Supplementary material for: Histological and molecular glioblastoma, IDH-wildtype: a real-world landscape using the 2021 WHO classification of central nervous system tumors
Source: Front Oncol. 2023 Jul 6;13:1200815. doi: 10.3389/fonc.2023.1200815 (PMC10358772; doi:10.3389/fonc.2023.1200815)
Supplement: Supplementary file 3 [file Image_3.pdf]

Supplementary Figure 3. Kaplan–Meier curves showing no significant effects of certain genetic and immunohistochemical alterations on the overall survival of patients with molecular glioblastomas (1-31). Since the numbers of patients divided into two groups (e.g. FGFR1 Wildtype/FGFR1 Alteration) using different parameters varied, we only enrolled the parameters using which the number of patients in either group was above 3. Parameters used in this section were shown as follows.

(1) Age 1
(2) Stupp protocol 2
(3) NeuN 3
(4) Syn 4
(5) MGMT promotor methylation 5
(6) TERT promotor mutation 6
(7) EGFR Amplification 7
(8) CDKN2A/B homozygous deletion 8
(9) BRAF 9
(10) FGFR1 10
(11) FGFR2 11
(12) FGFR4 12
(13) KIT 13
(14) KRAS 14
(15) MET 15
(16) MYB 16
(17) MYBL1 17
(18) MYC 18
(19) MYCN 19
(20) NF1 20
(21) NOTCH1 21
(22) NTRK2 22
(23) NTRK3 23
(24) PDGFRA 24
(25) PEG3 25
(26) PIK3CA 26
(27) PPM1D 27
(28) PTEN 28
(29) PTPN11 29
(30) RB1 30
(31) TOP3A 31

(1)

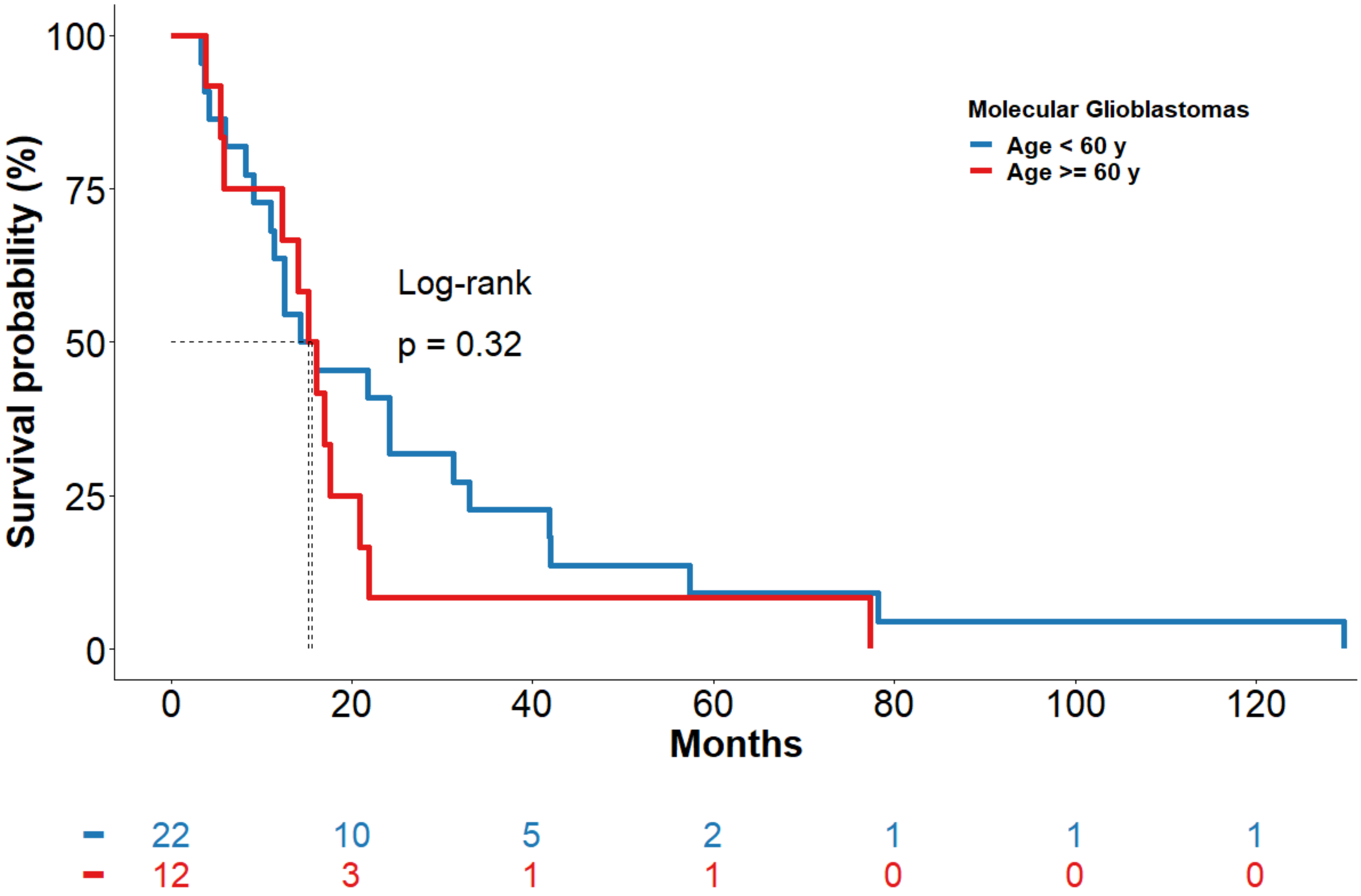

Survival probability (%)
100
75
50
25
0
Log-rank
p = 0.32
Molecular Glioblastomas
Age < 60 y
Age >= 60 y
0
20
40
60
80
100
120
Months
22 10 5 2 1 1 1
12 3 1 1 0 0 0

(2)

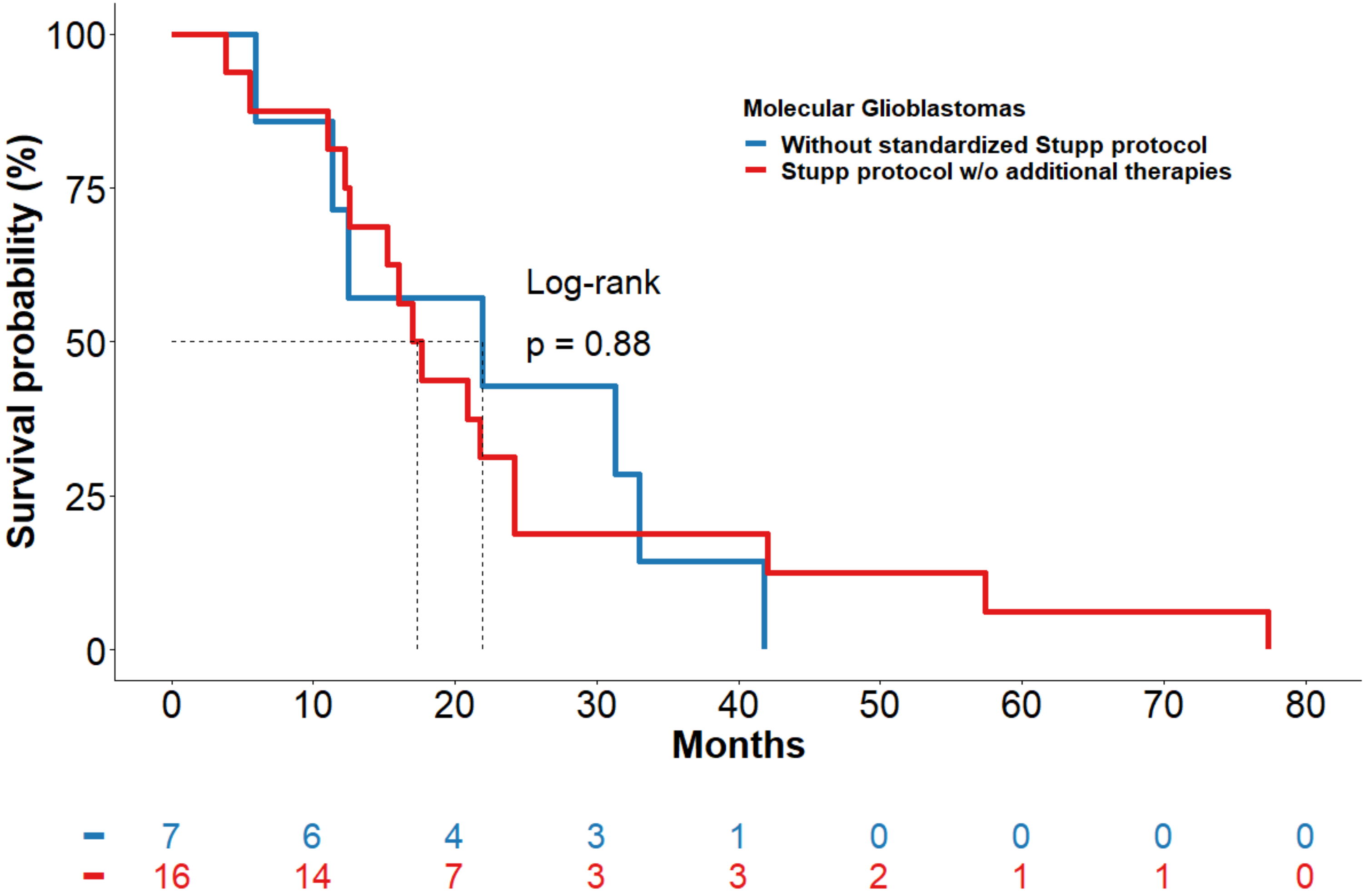

Molecular Glioblastomas
Without standardized Stupp protocol
Stupp protocol w/o additional therapies
Log-rank
p = 0.88
Survival probability (%)
100
75
50
25
0
0
10
20
30
40
50
60
70
80
Months
7 6 4 3 1 0 0 0 0
16 14 7 3 3 2 1 1 0

(3)

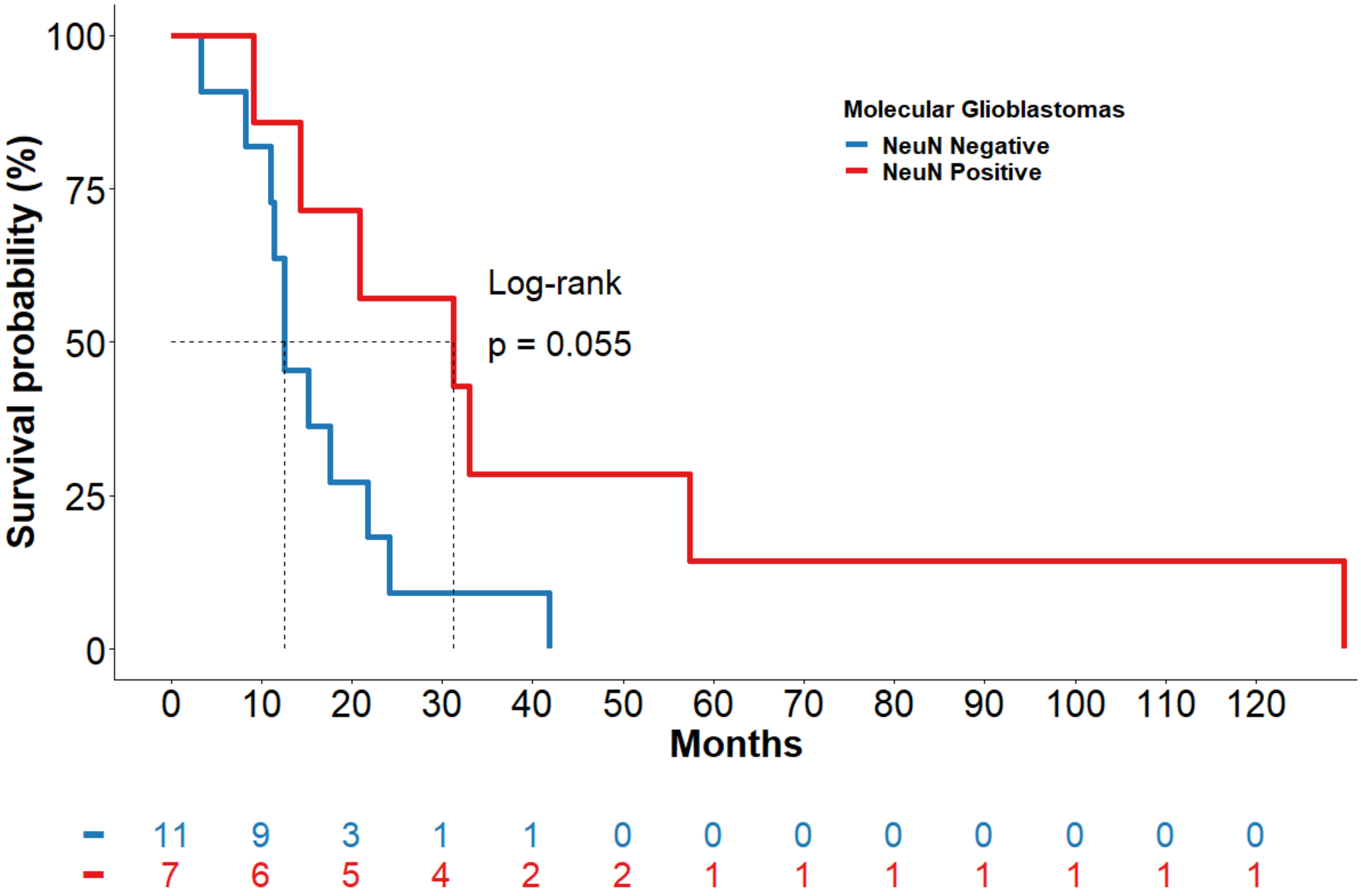

Survival probability (%)
100
75
50
25
0
Log-rank
p = 0.055
Molecular Glioblastomas
NeuN Negative
NeuN Positive
0
10
20
30
40
50
60
70
80
90
100
110
120
Months
11 9 3 1 1 0 0 0 0 0 0 0 0
7 6 5 4 2 2 1 1 1 1 1 1 1

(4)

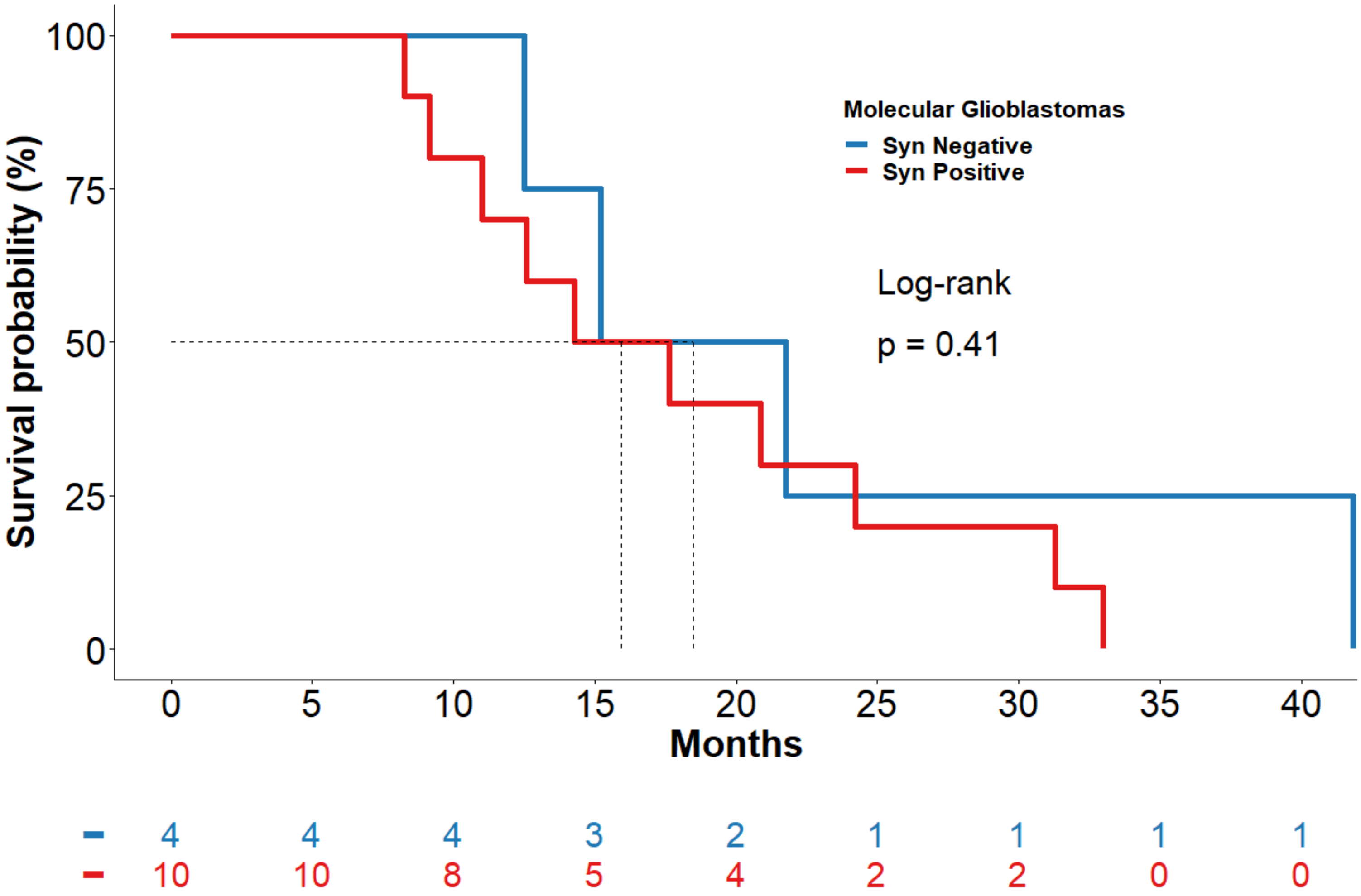

Survival probability (%)
100
75
50
25
0
Molecular Glioblastomas
Syn Negative
Syn Positive
Log-rank
p = 0.41
0
5
10
15
20
25
30
35
40
Months
4 4 4 3 2 1 1 1 1
10 10 8 5 4 2 2 0 0

(5)

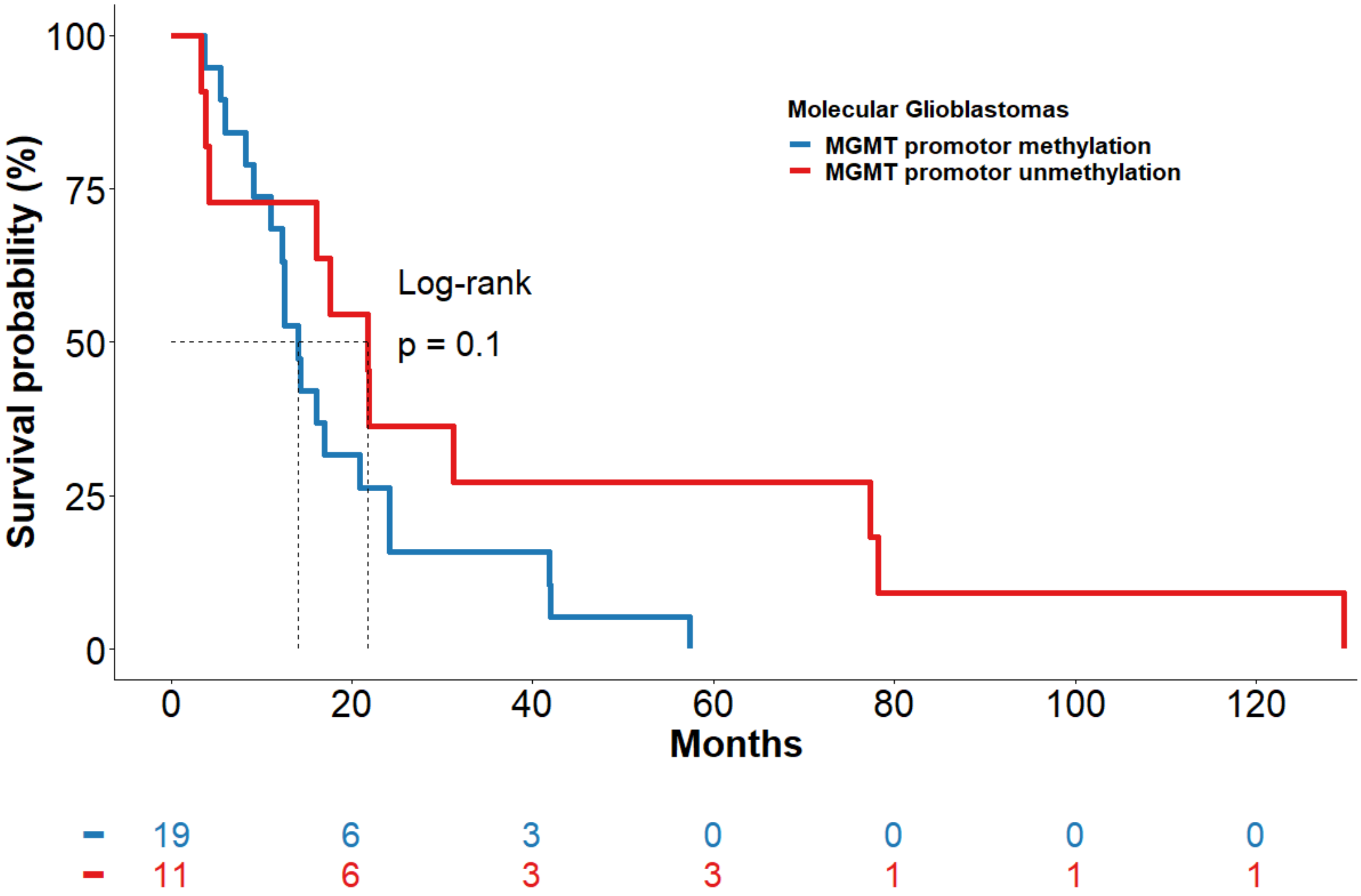

Survival probability (%)
100
75
50
25
0
Molecular Glioblastomas
MGMT promotor methylation
MGMT promotor unmethylation
Log-rank
p = 0.1
0
20
40
60
80
100
120
Months
19
6
3
0
0
0
0
11
6
3
3
1
1
1

(6)

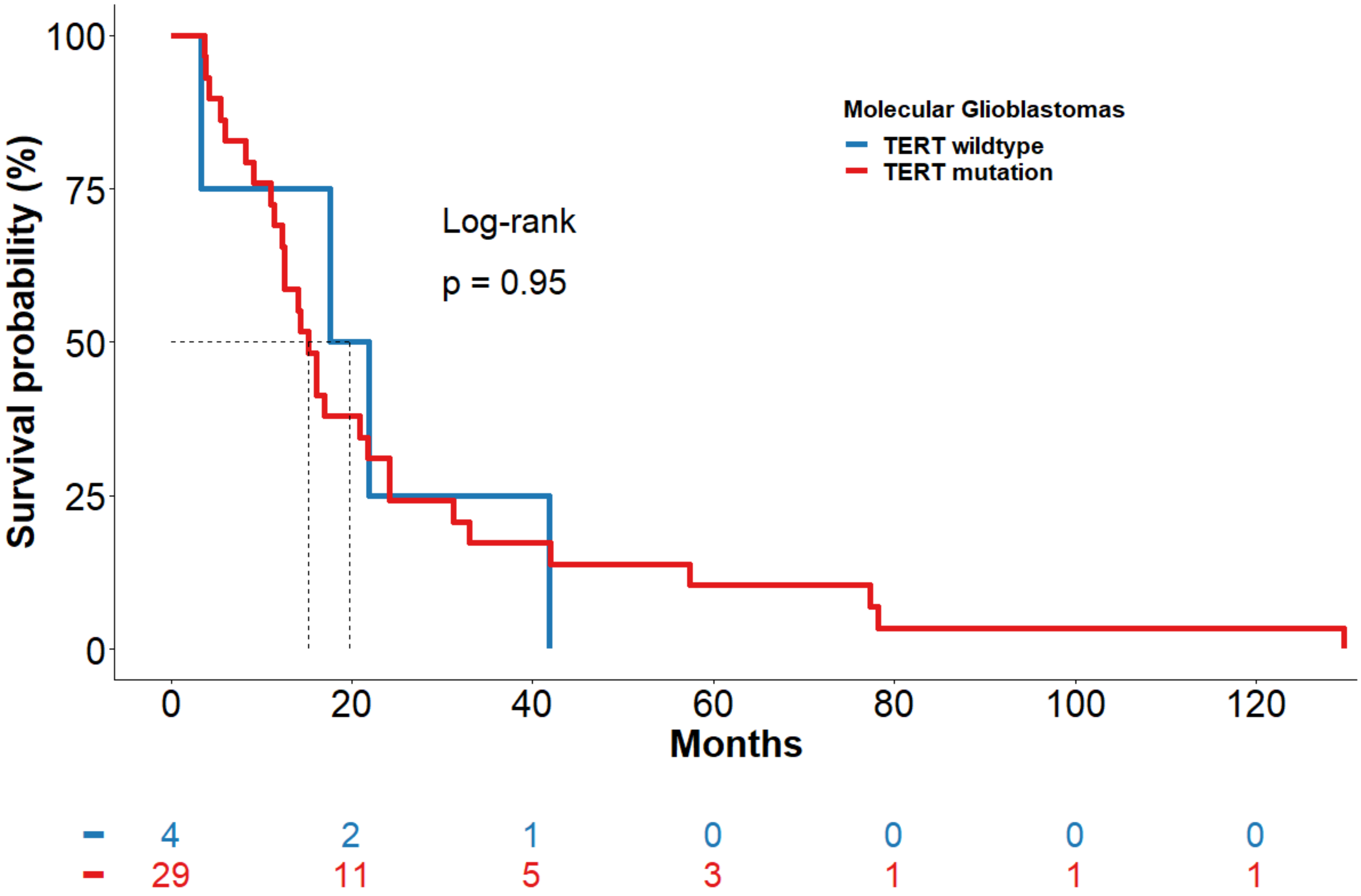
Molecular Glioblastomas
TERT wildtype
TERT mutation
Log-rank
p = 0.95
Survival probability (%)
100
75
50
25
0
0
20
40
60
80
100
120
Months
4 2 1 0 0 0 0
29 11 5 3 1 1 1

(7)

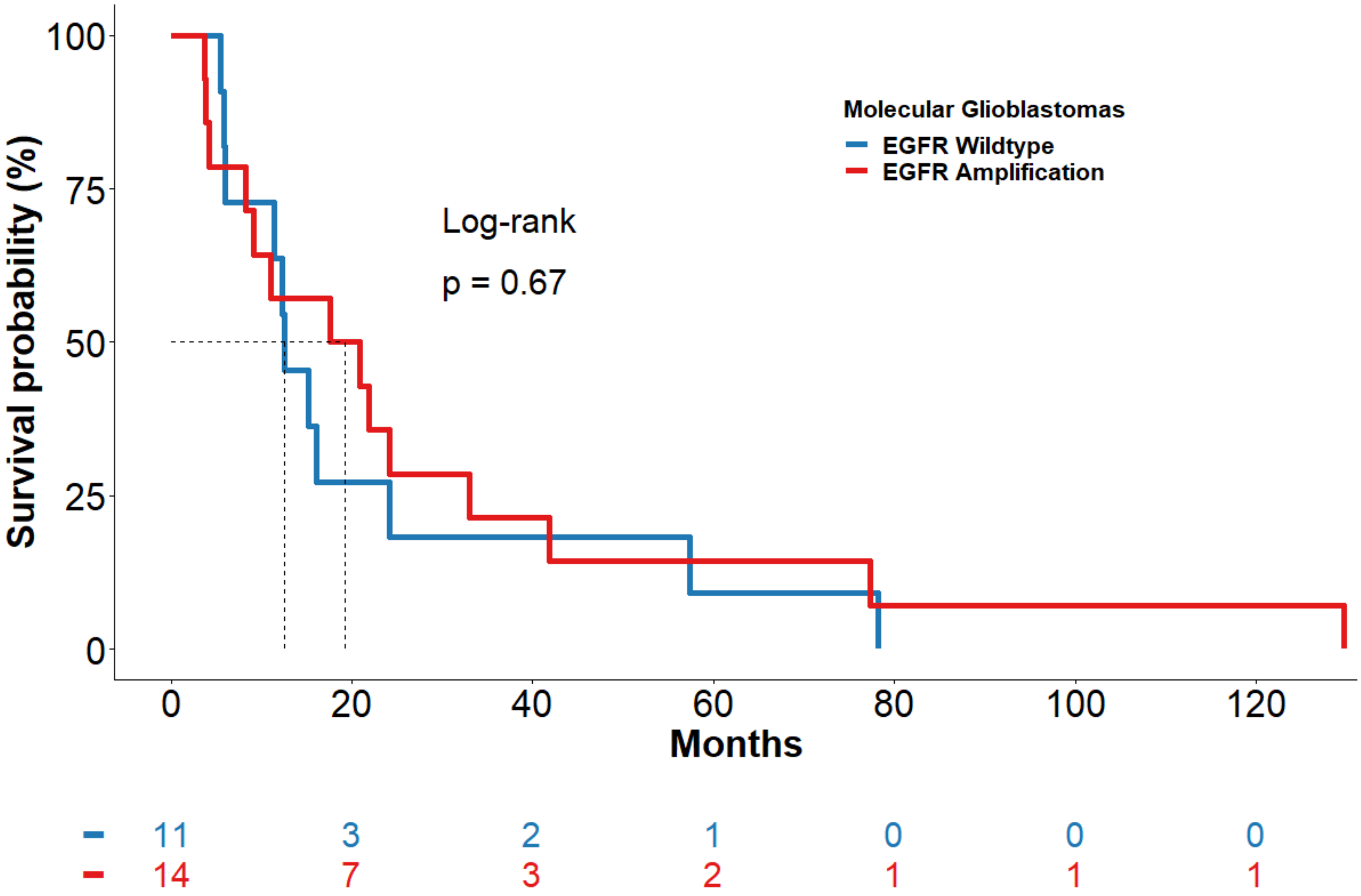

Survival probability (%)
100
75
50
25
0
Log-rank
p = 0.67
Molecular Glioblastomas
EGFR Wildtype
EGFR Amplification
0
20
40
60
80
100
120
Months
11 3 2 1 0 0 0
14 7 3 2 1 1 1

(8)

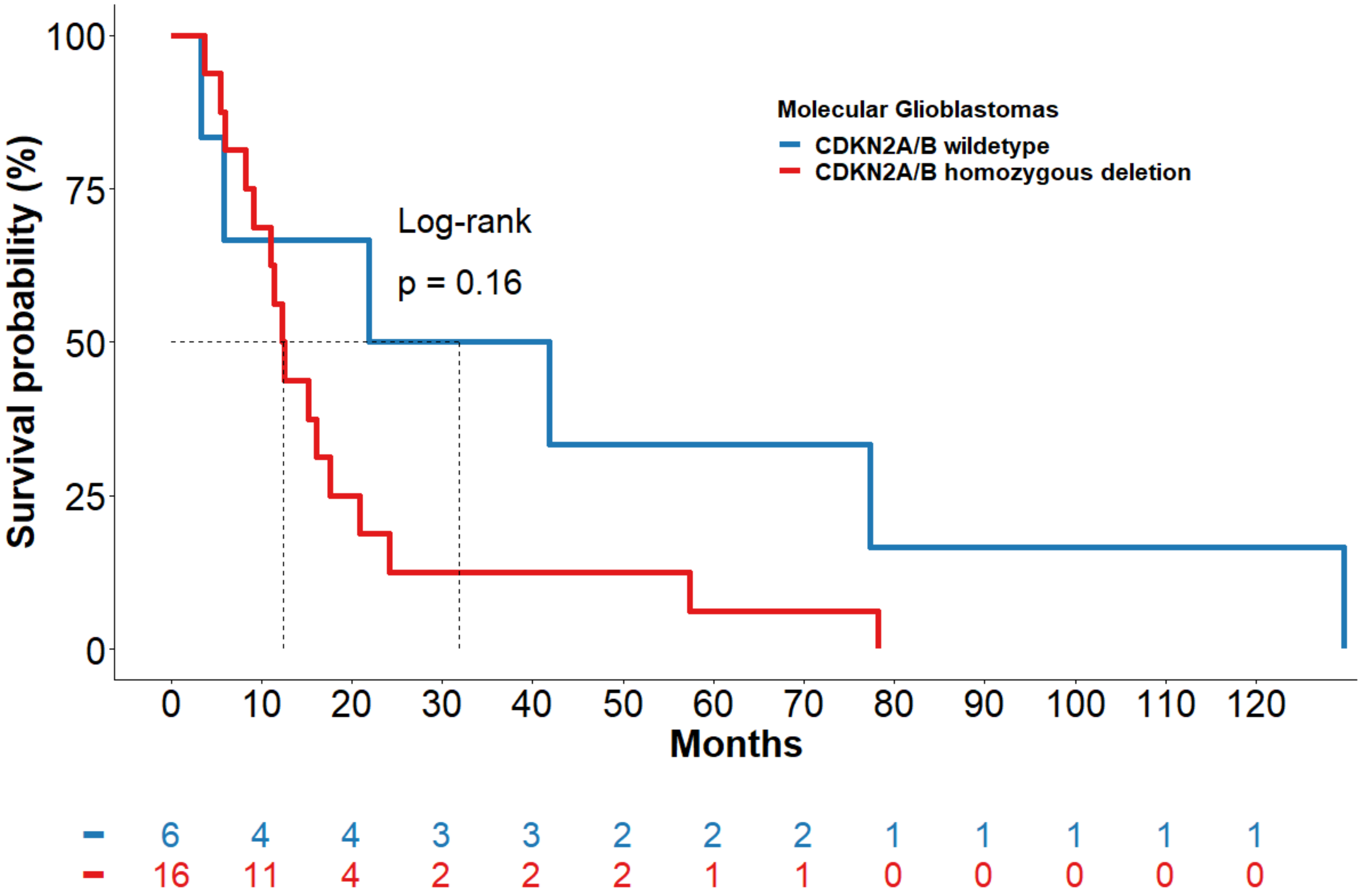

Survival probability (%)
100
75
50
25
0
Molecular Glioblastomas
CDKN2A/B wildetype
CDKN2A/B homozygous deletion
Log-rank
p = 0.16
0
10
20
30
40
50
60
70
80
90
100
110
120
Months
6 4 4 3 3 2 2 2 1 1 1 1 1
16 11 4 2 2 2 1 1 0 0 0 0 0

(9)

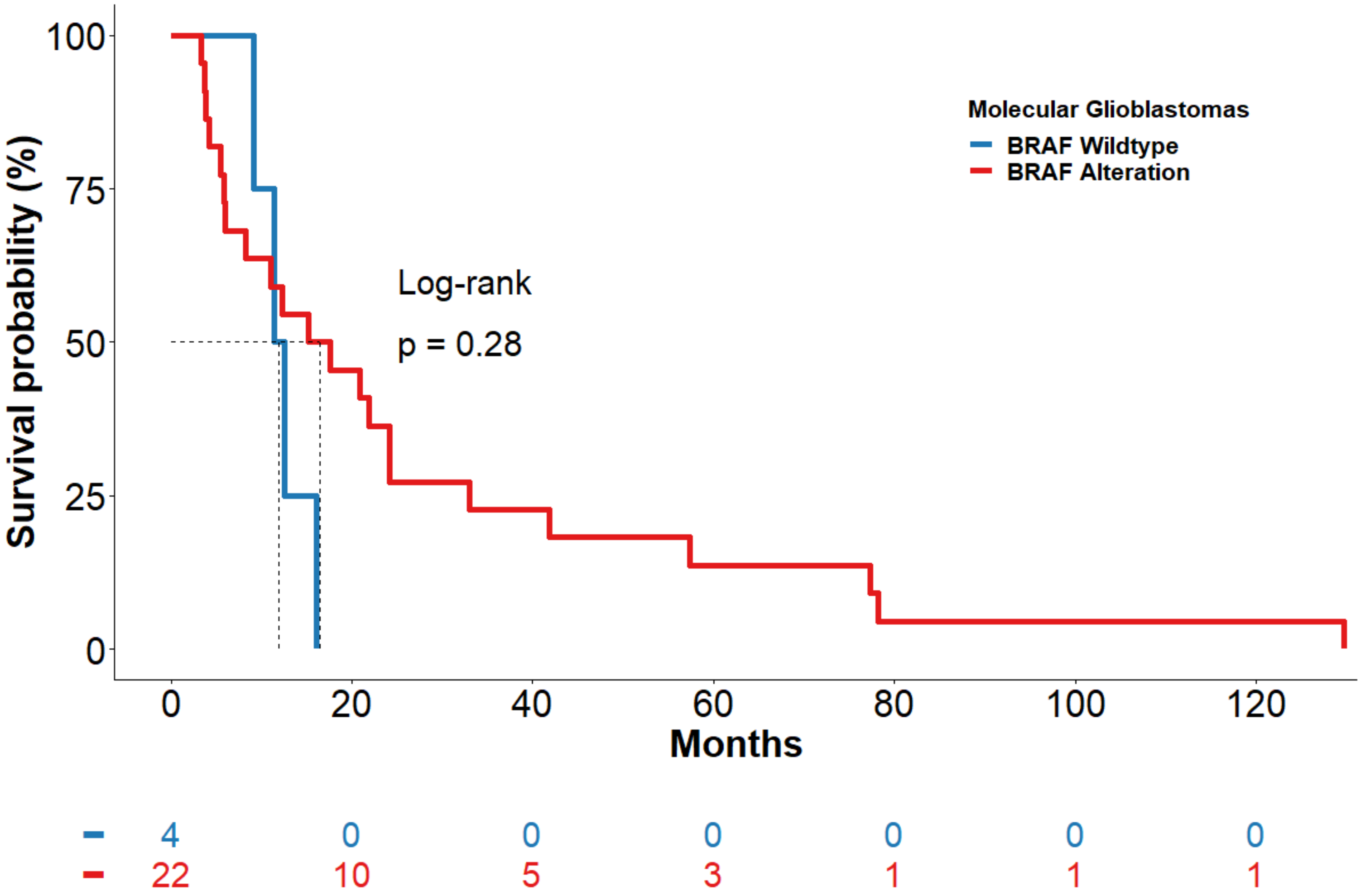

Survival probability (%)
100
75
50
25
0
Log-rank
p = 0.28
Molecular Glioblastomas
BRAF Wildtype
BRAF Alteration
0
20
40
60
80
100
120
Months
4
0
0
0
0
0
0
22
10
5
3
1
1
1

(10)

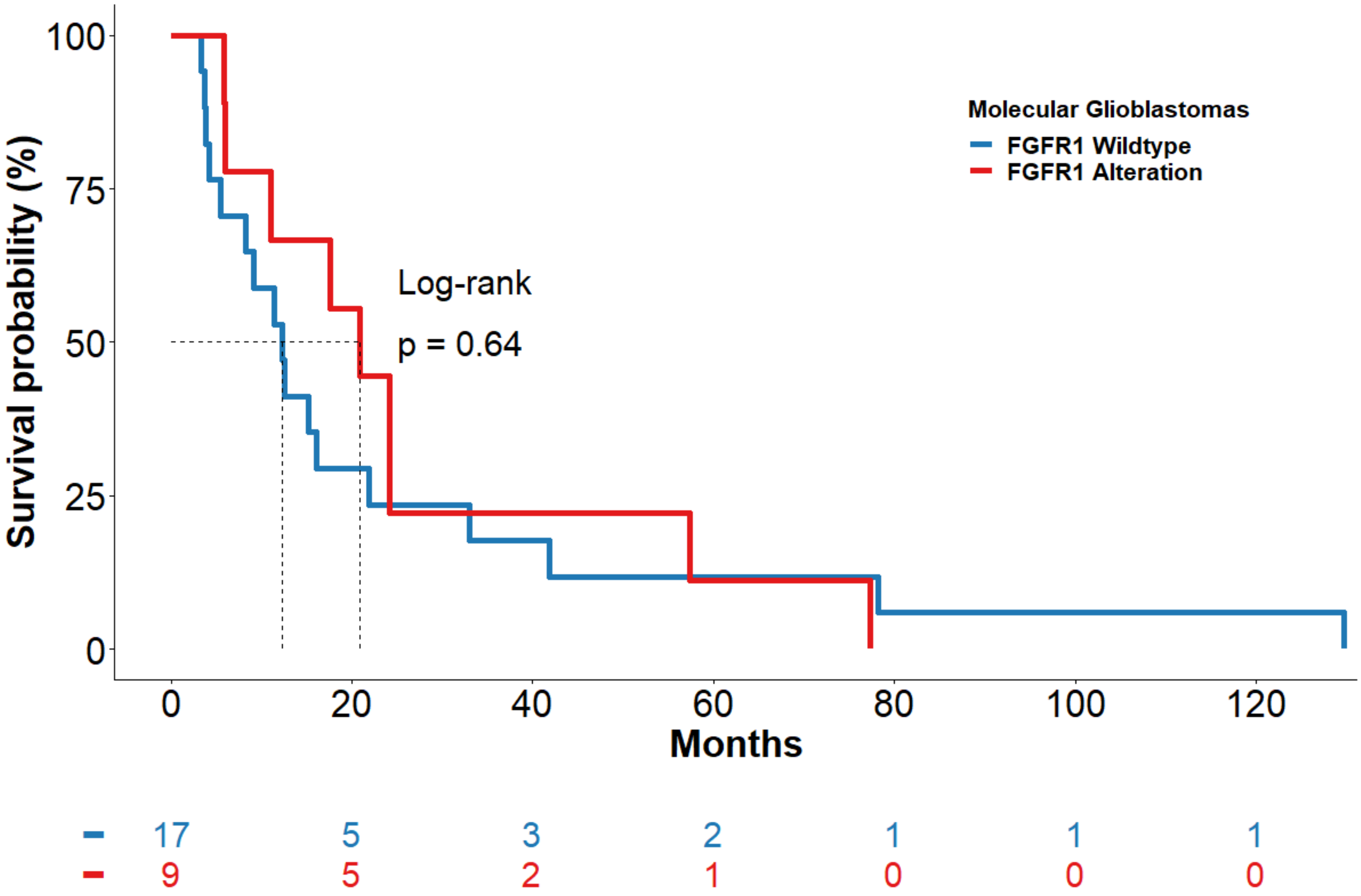

Survival probability (%)
100
75
50
25
0
Log-rank
p = 0.64
Molecular Glioblastomas
FGFR1 Wildtype
FGFR1 Alteration
0
20
40
60
80
100
120
Months
17 5 3 2 1 1 1
9 5 2 1 0 0 0

(11)

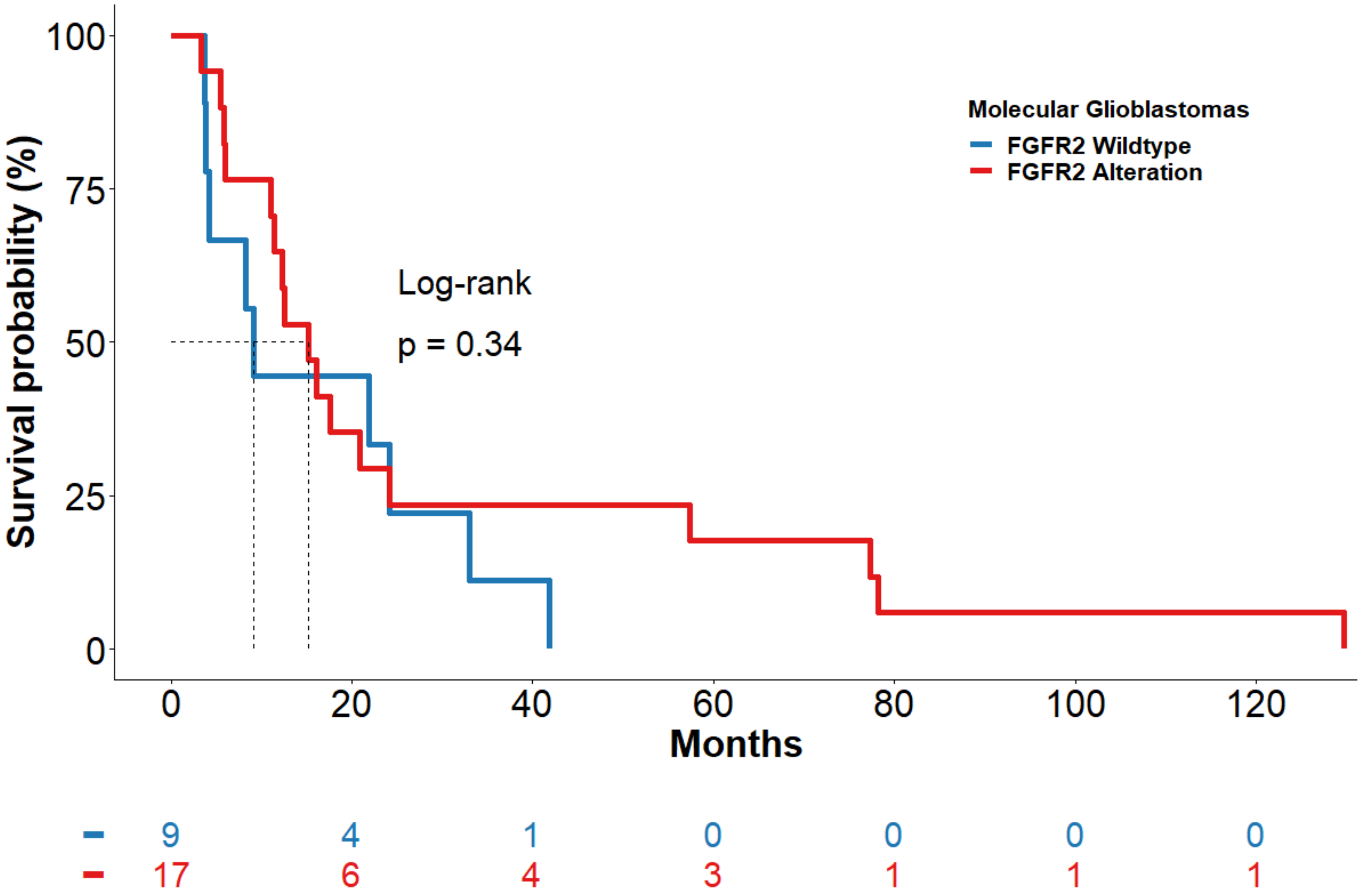

Molecular Glioblastomas
FGFR2 Wildtype
FGFR2 Alteration
Log-rank
p = 0.34
Survival probability (%)
100
75
50
25
0
0
20
40
60
80
100
120
Months
9
4
1
0
0
0
0
17
6
4
3
1
1
1

(12)

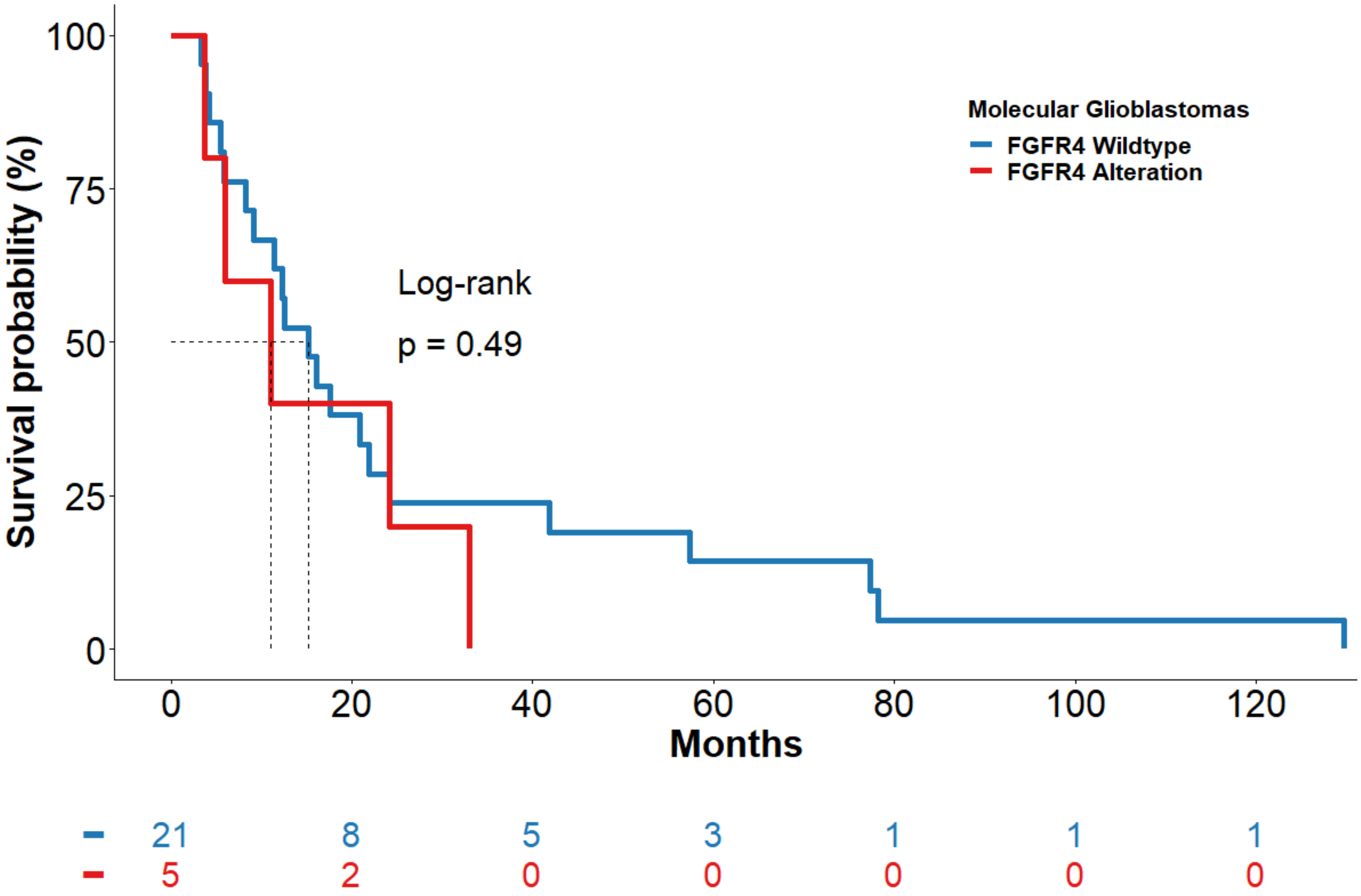

Survival probability (%)
100
75
50
25
0
Log-rank
p = 0.49
Molecular Glioblastomas
FGFR4 Wildtype
FGFR4 Alteration
0
20
40
60
80
100
120
Months
21 8 5 3 1 1 1
5 2 0 0 0 0 0

(13)

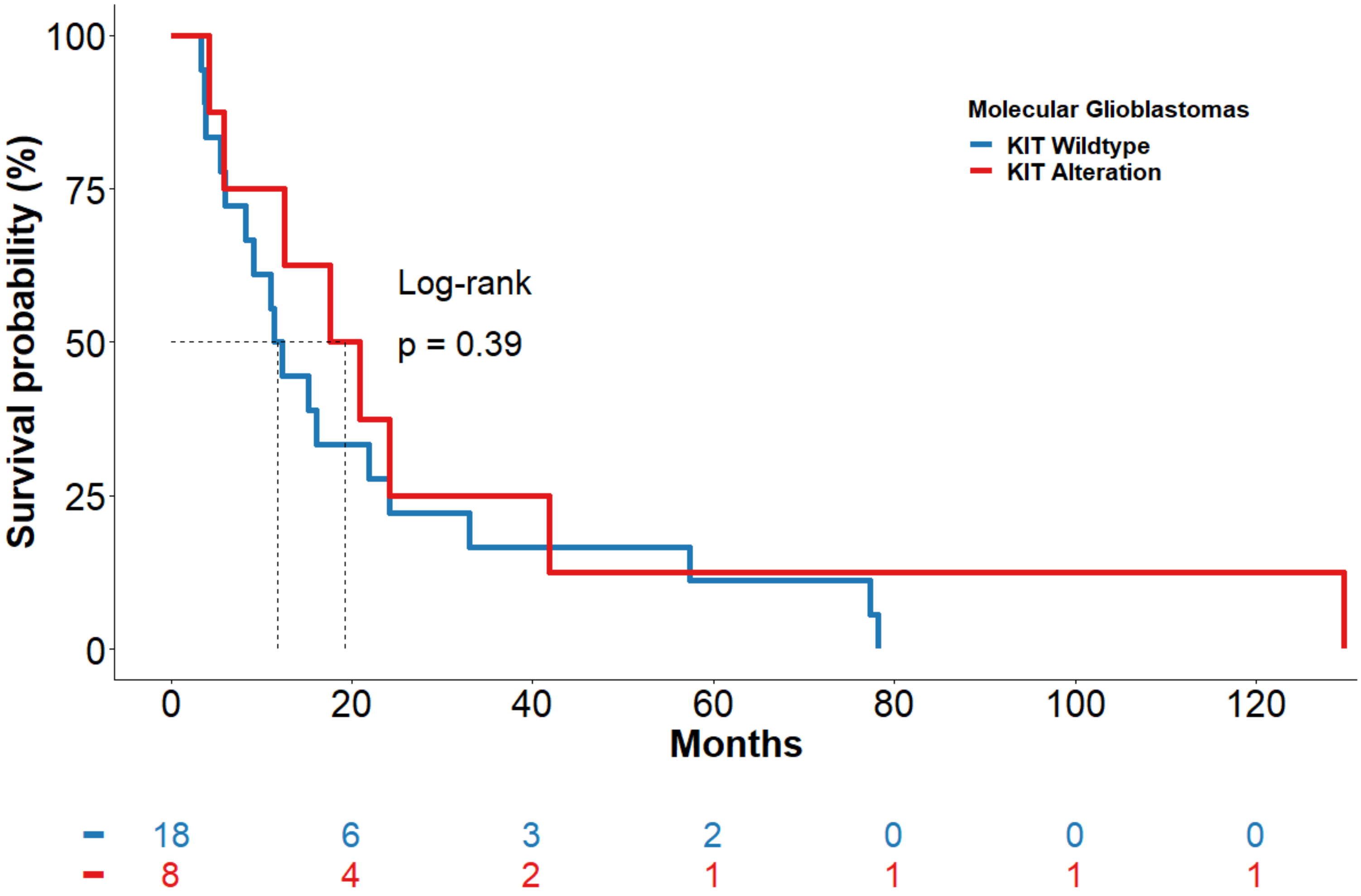

Survival probability (%)
100
75
50
25
0
Log-rank
p = 0.39
Molecular Glioblastomas
KIT Wildtype
KIT Alteration
0
20
40
60
80
100
120
Months
18
6
3
2
0
0
0
8
4
2
1
1
1
1

(14)

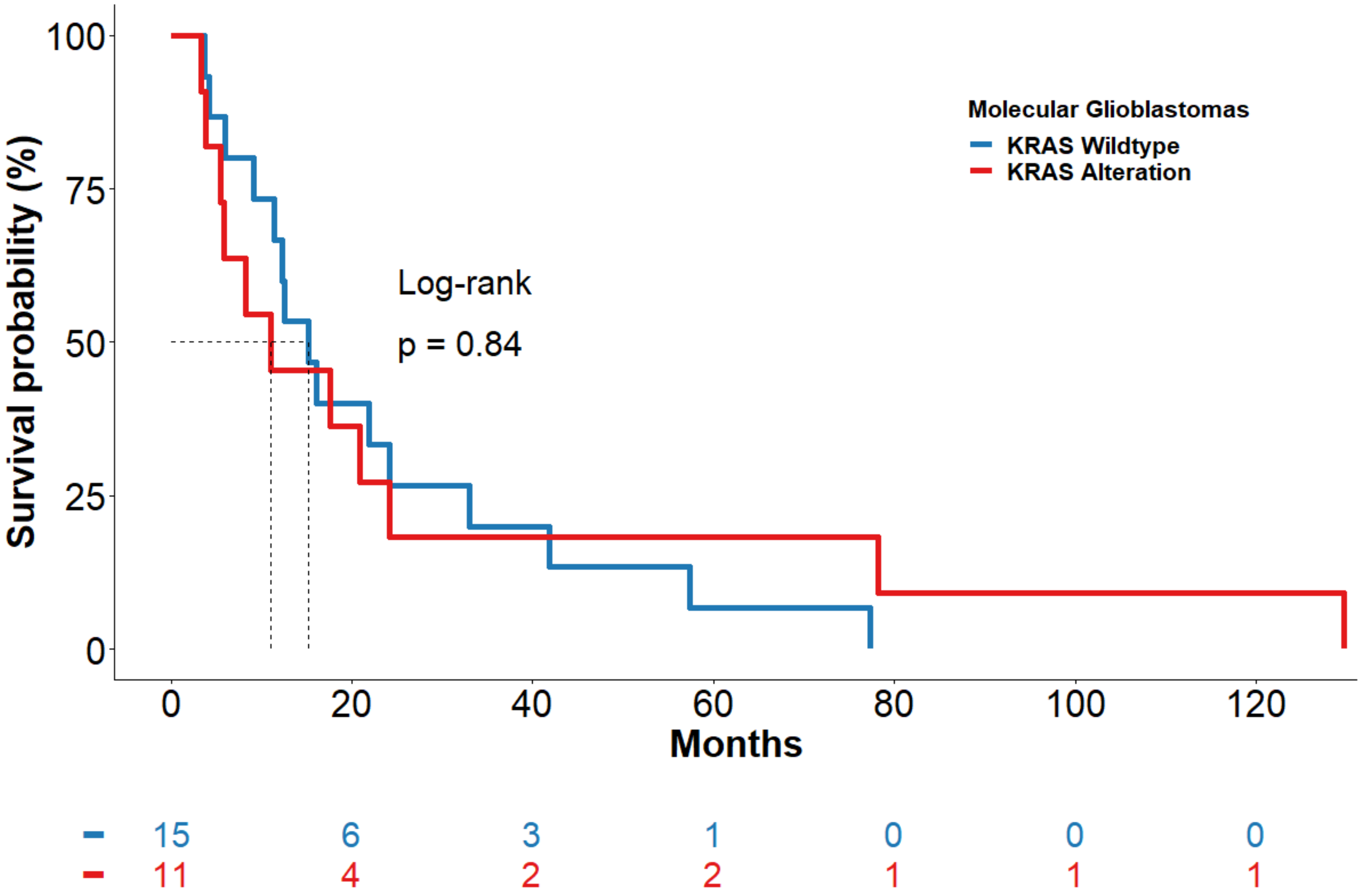
Survival probability (%)
100
75
50
25
0
Log-rank
p = 0.84
Molecular Glioblastomas
KRAS Wildtype
KRAS Alteration
0
20
40
60
80
100
120
Months
15 6 3 1 0 0 0
11 4 2 2 1 1 1

(15)

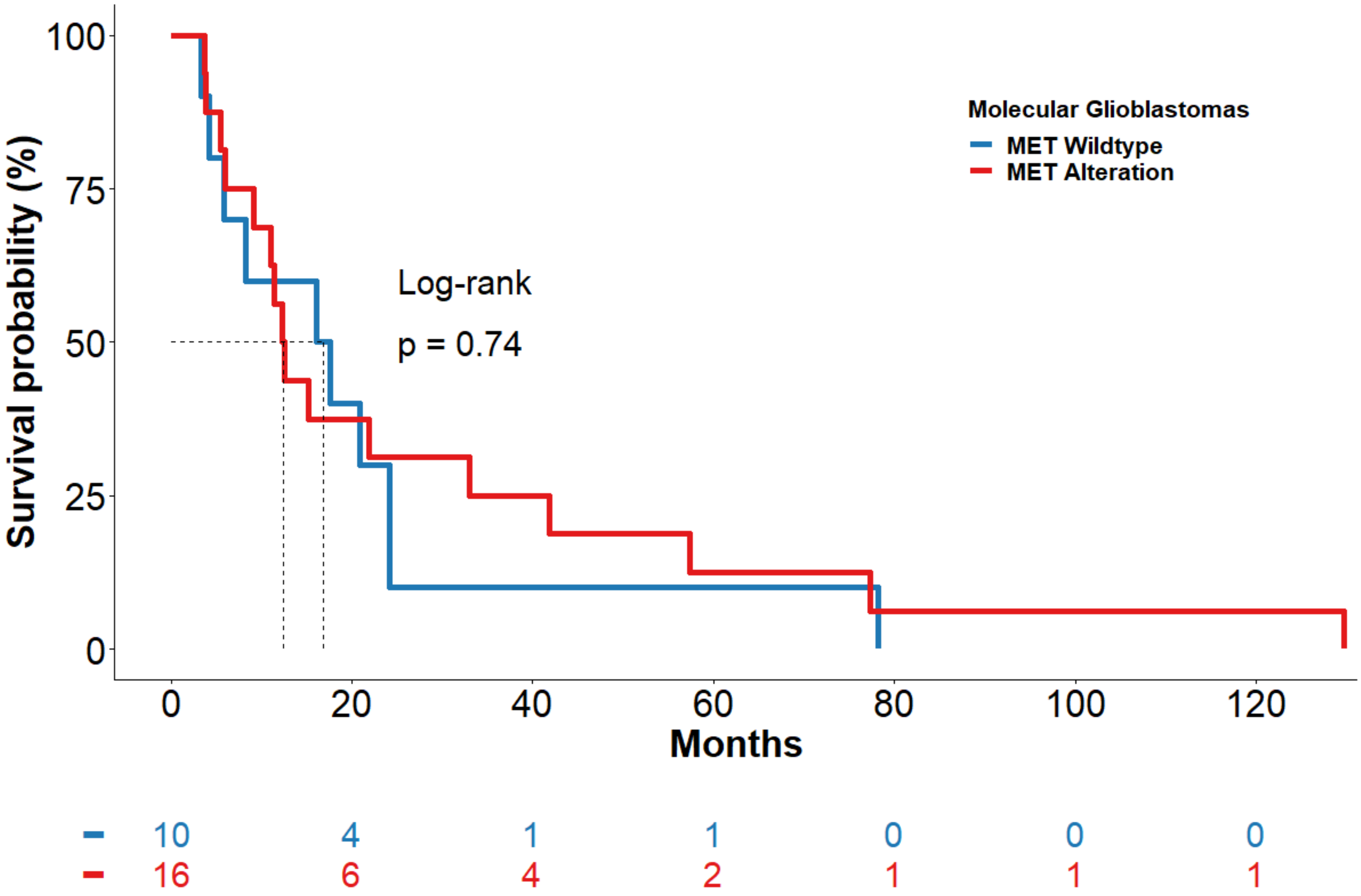

Survival probability (%)
100
75
50
25
0
Log-rank
p = 0.74
Molecular Glioblastomas
MET Wildtype
MET Alteration
0
20
40
60
80
100
120
Months
10 4 1 1 0 0 0
16 6 4 2 1 1 1

(16)

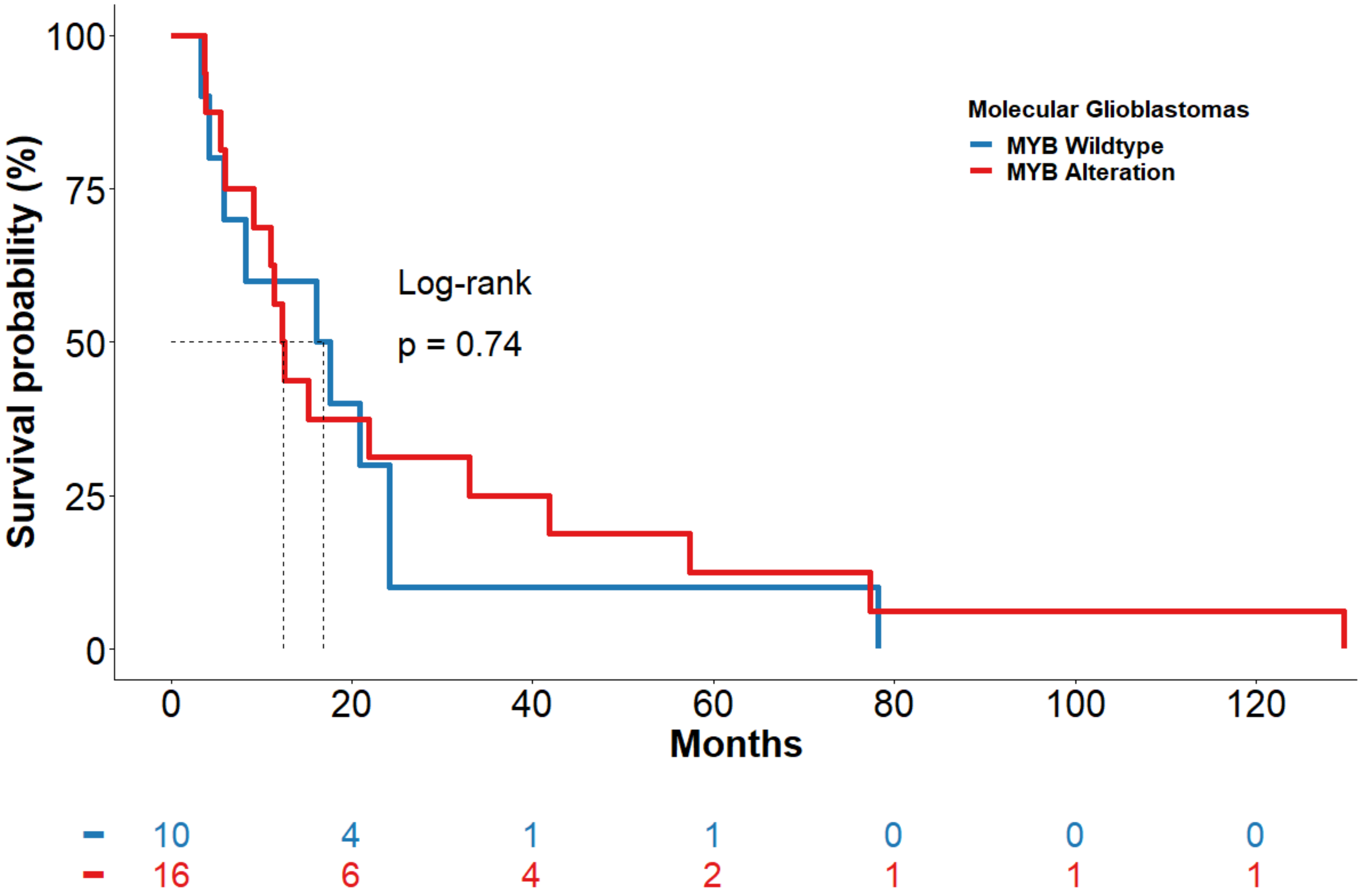

Survival probability (%)
100
75
50
25
0
Log-rank
p = 0.74
Molecular Glioblastomas
MYB Wildtype
MYB Alteration
0
20
40
60
80
100
120
Months
10 4 1 1 0 0 0
16 6 4 2 1 1 1

(17)

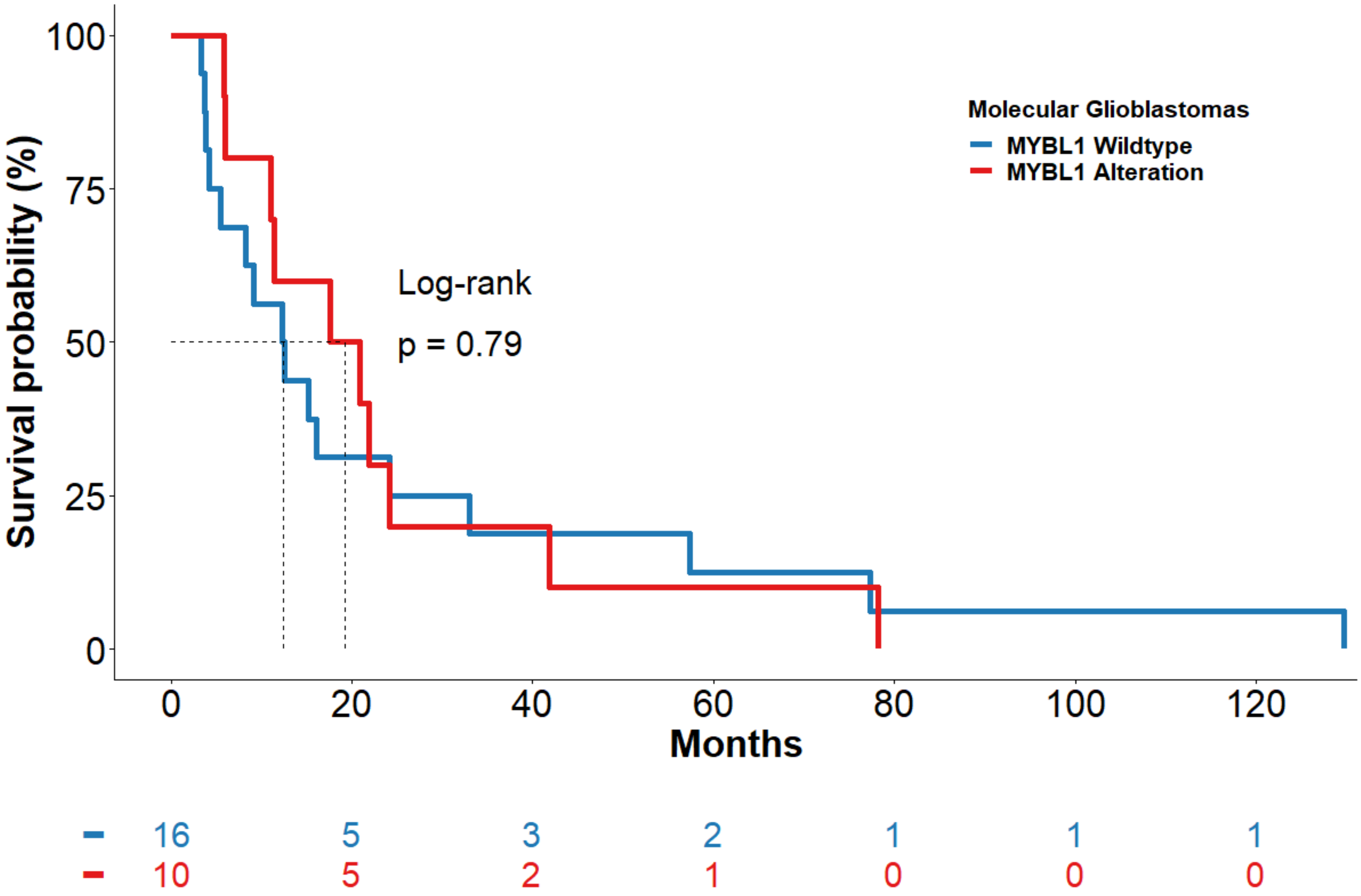

Survival probability (%)
100
75
50
25
0
Log-rank
p = 0.79
Molecular Glioblastomas
MYBL1 Wildtype
MYBL1 Alteration
0
20
40
60
80
100
120
Months
16 5 3 2 1 1 1
10 5 2 1 0 0 0

(18)

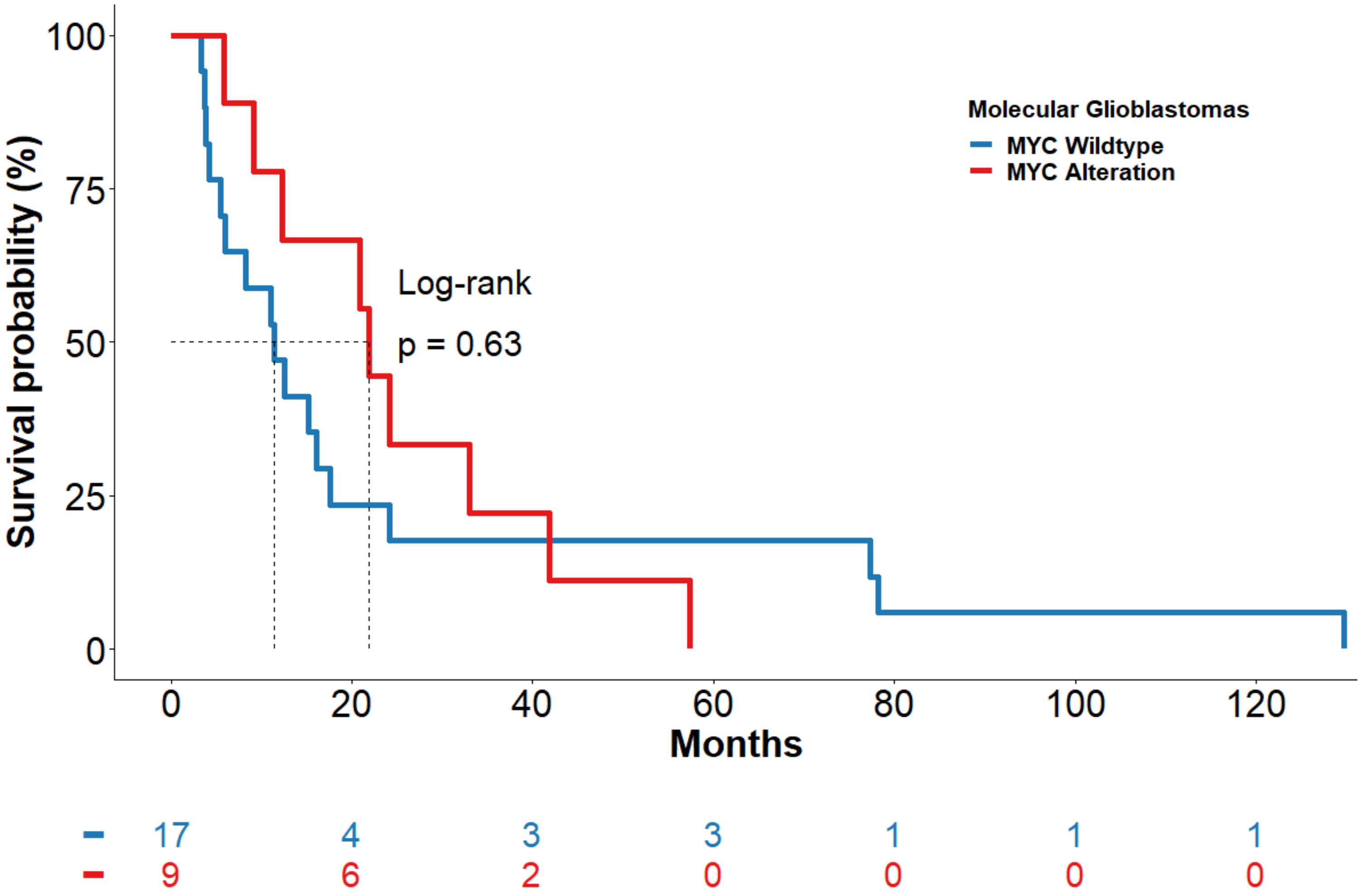

Survival probability (%)
100
75
50
25
0
Log-rank
p = 0.63
Molecular Glioblastomas
MYC Wildtype
MYC Alteration
0
20
40
60
80
100
120
Months
17 4 3 3 1 1 1
9 6 2 0 0 0 0

(19)

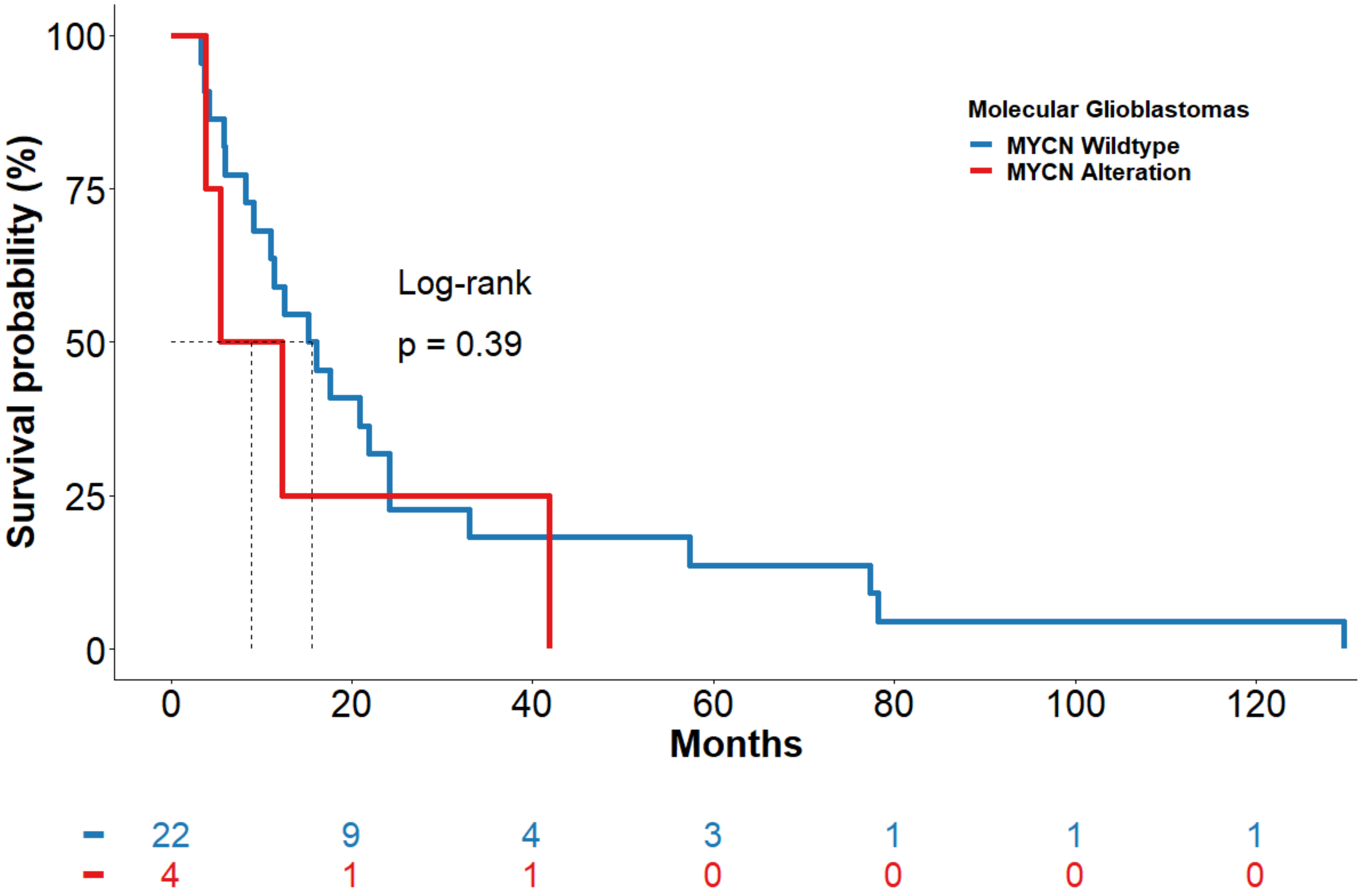

Survival probability (%)
100
75
50
25
0
Molecular Glioblastomas
MYCN Wildtype
MYCN Alteration
Log-rank
p = 0.39
0
20
40
60
80
100
120
Months
22 9 4 3 1 1 1
4 1 1 0 0 0 0

(20)

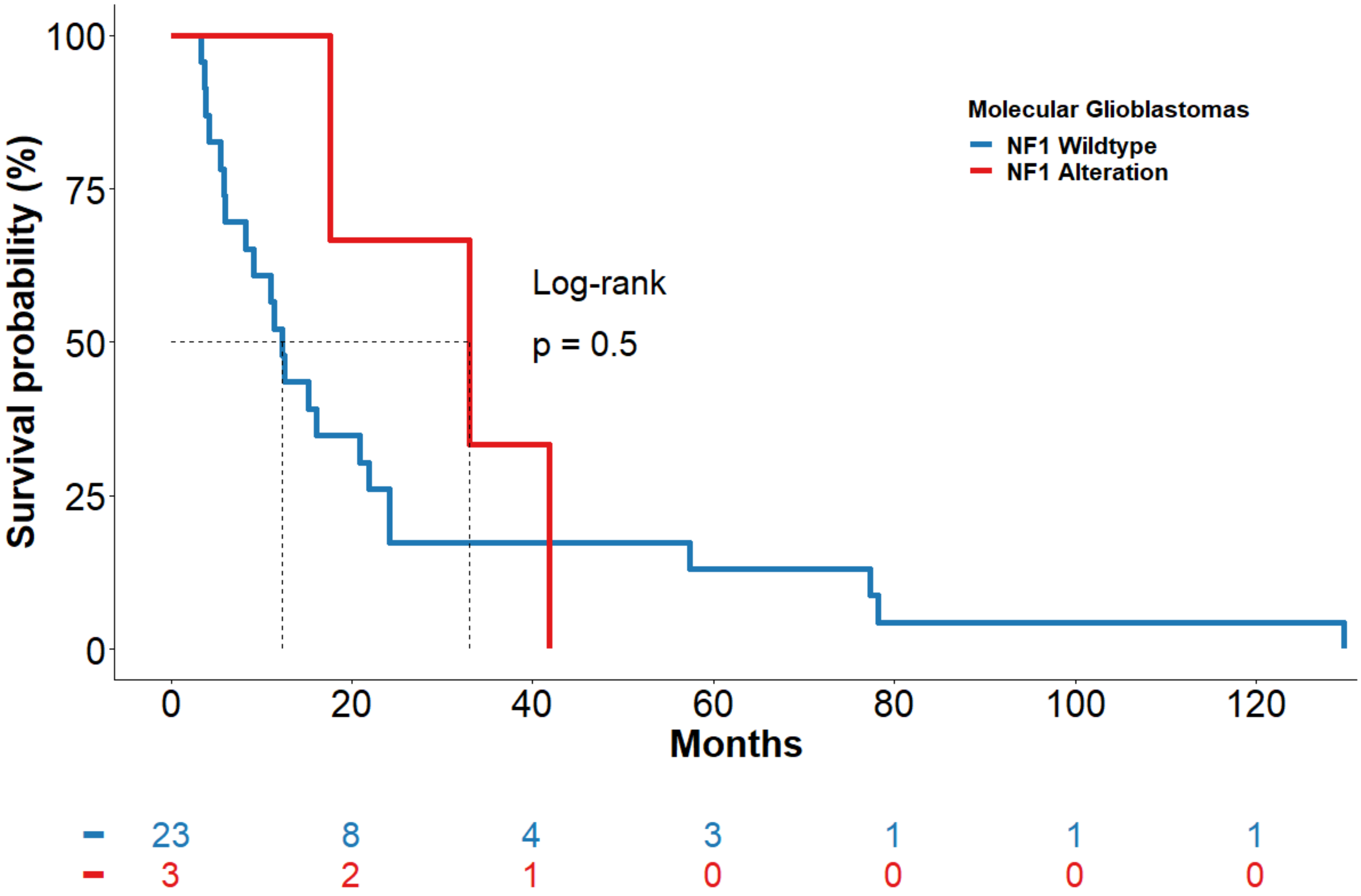

Survival probability (%)
100
75
50
25
0
Log-rank
p = 0.5
Molecular Glioblastomas
NF1 Wildtype
NF1 Alteration
0
20
40
60
80
100
120
Months
23
8
4
3
1
1
1
3
2
1
0
0
0
0

(21)

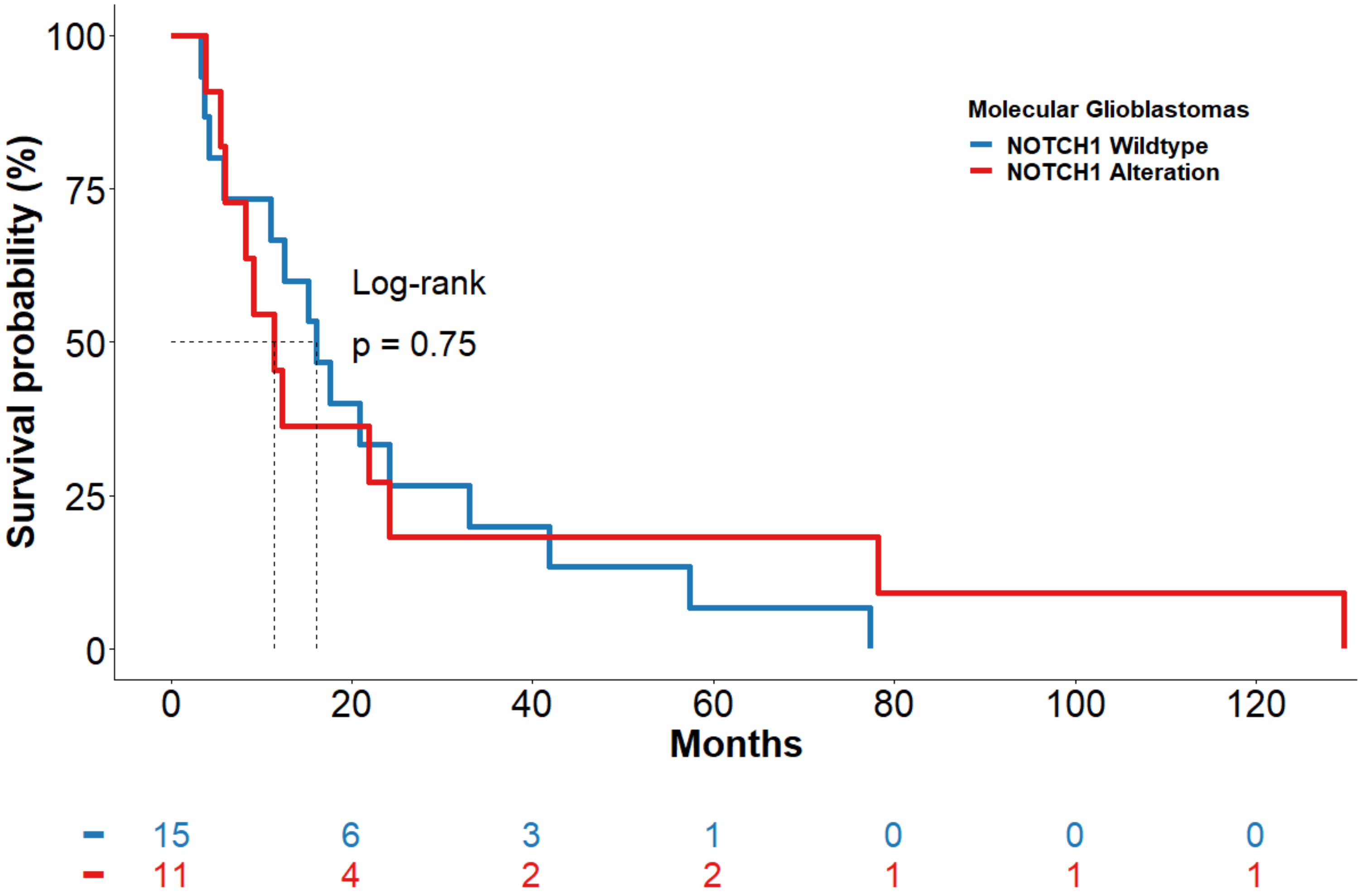
Survival probability (%)
100
75
50
25
0
Log-rank
p = 0.75
Molecular Glioblastomas
NOTCH1 Wildtype
NOTCH1 Alteration
0
20
40
60
80
100
120
Months
15 6 3 1 0 0 0
11 4 2 2 1 1 1

(22)

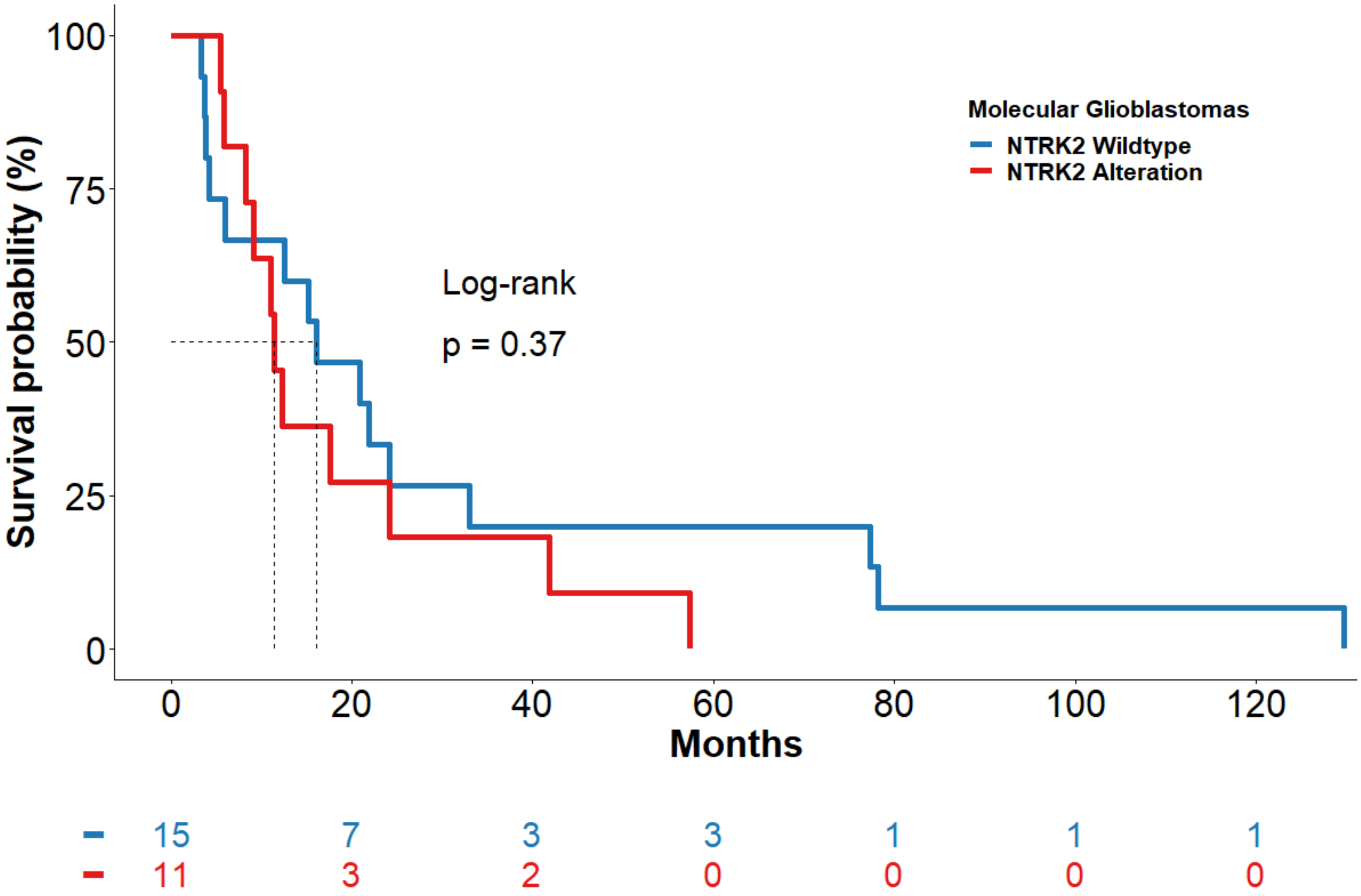

100
75
50
25
0
Survival probability (%)
Log-rank
p = 0.37
Molecular Glioblastomas
NTRK2 Wildtype
NTRK2 Alteration
0
20
40
60
80
100
120
Months
15 7 3 3 1 1 1
11 3 2 0 0 0 0

(23)

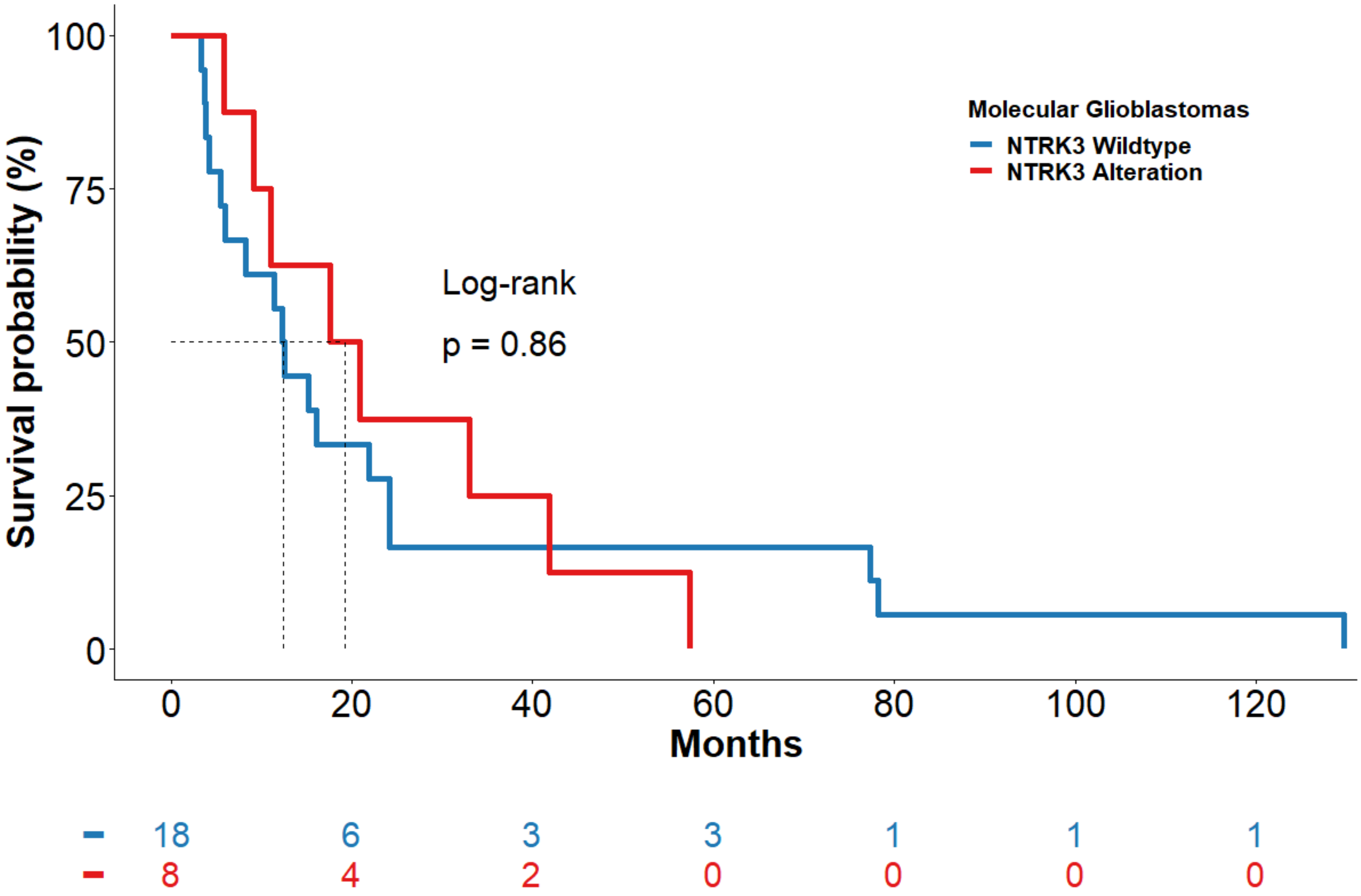

Survival probability (%)
100
75
50
25
0
Log-rank
p = 0.86
Molecular Glioblastomas
NTRK3 Wildtype
NTRK3 Alteration
0
20
40
60
80
100
120
Months
18 6 3 3 1 1 1
8 4 2 0 0 0 0

(24)

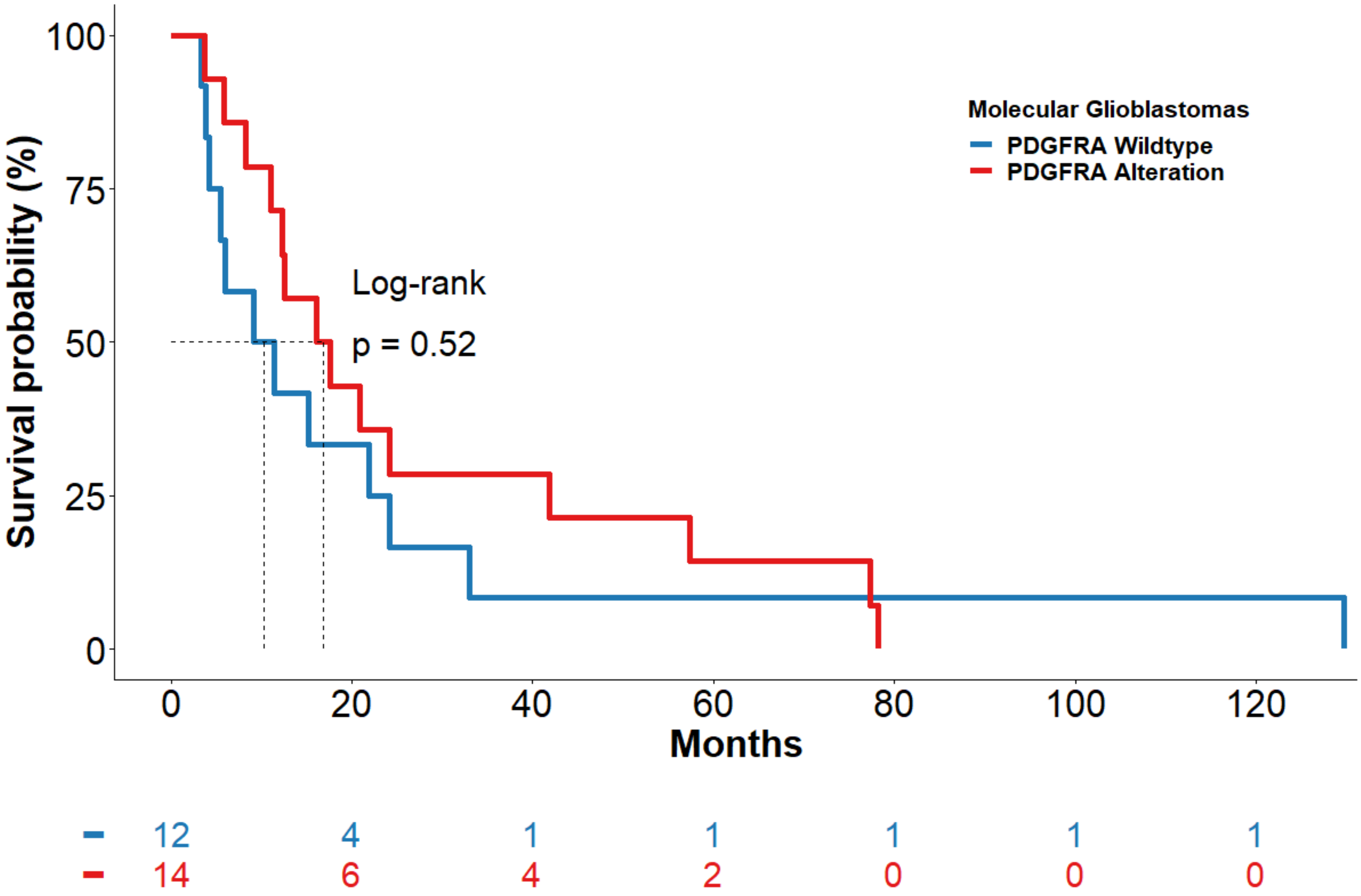

Survival probability (%)
100
75
50
25
0
Log-rank
p = 0.52
Molecular Glioblastomas
PDGFRA Wildtype
PDGFRA Alteration
0
20
40
60
80
100
120
Months
12 4 1 1 1 1 1
14 6 4 2 0 0 0

(25)

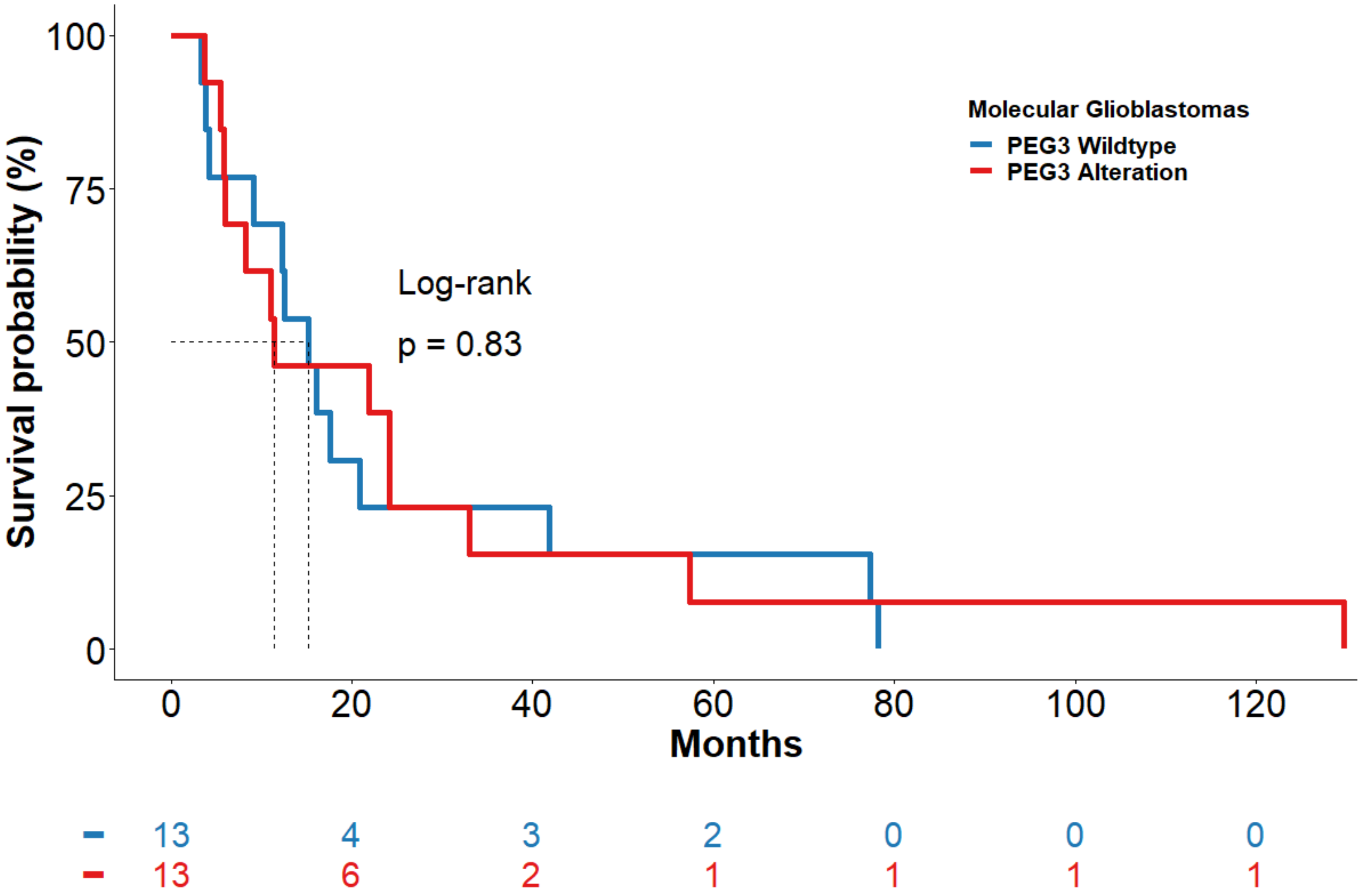

Survival probability (%)
100
75
50
25
0
Log-rank
p = 0.83
Molecular Glioblastomas
PEG3 Wildtype
PEG3 Alteration
0
20
40
60
80
100
120
Months
13
4
3
2
0
0
0
13
6
2
1
1
1
1

(26)

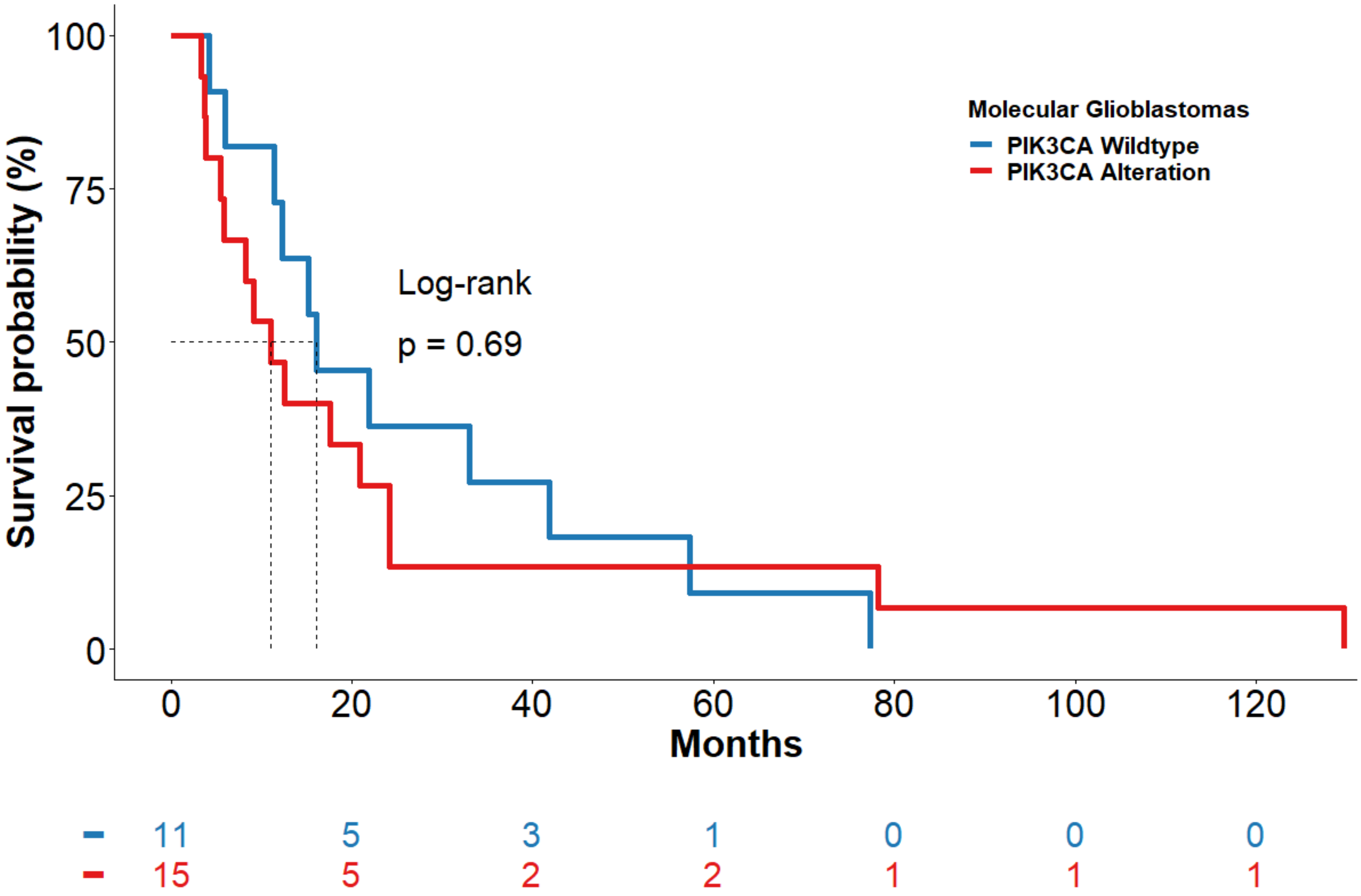

Survival probability (%)
100
75
50
25
0
Log-rank
p = 0.69
Molecular Glioblastomas
PIK3CA Wildtype
PIK3CA Alteration
0
20
40
60
80
100
120
Months
11 5 3 1 0 0 0
15 5 2 2 1 1 1

(27)

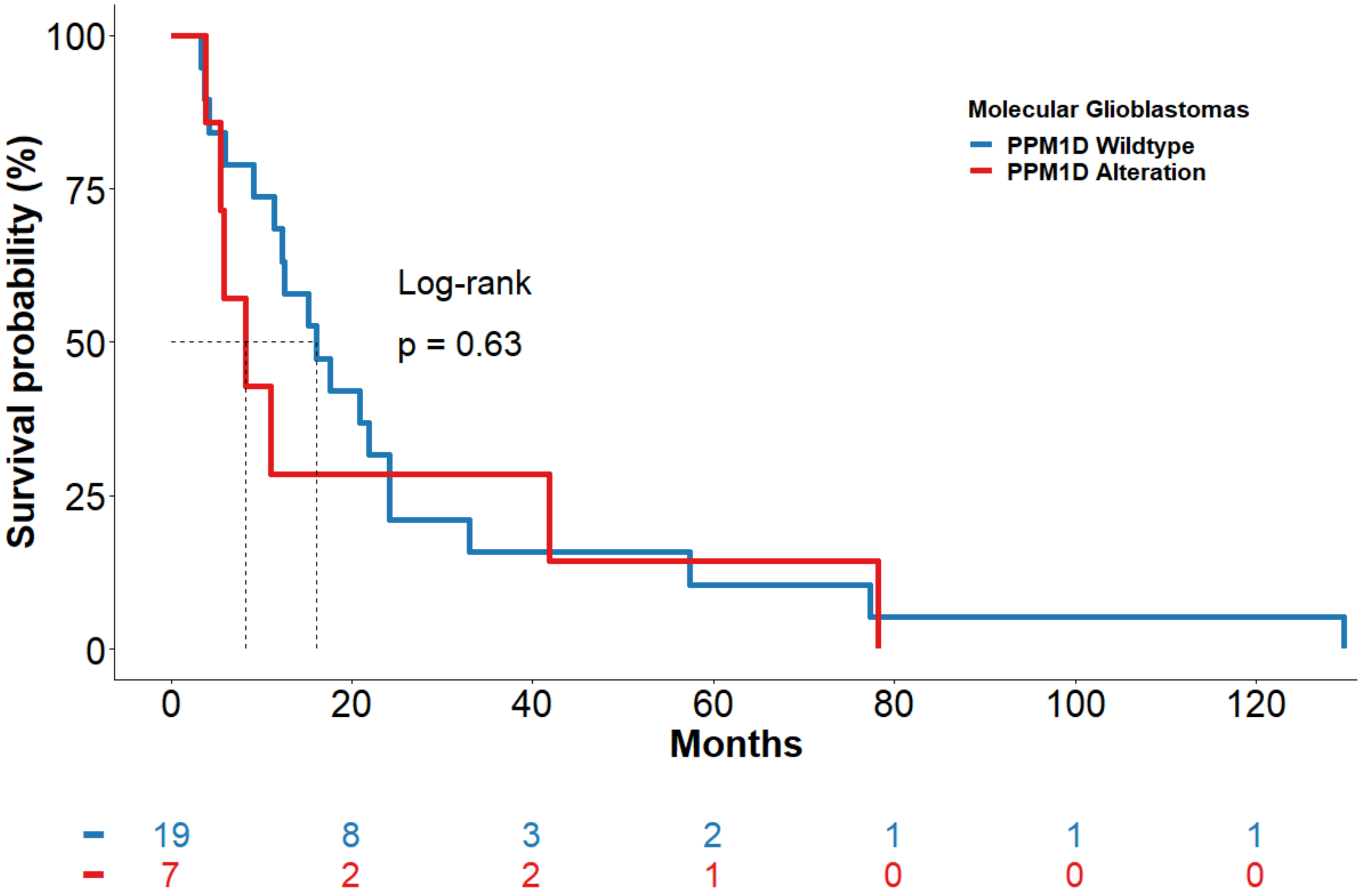
Survival probability (%)
100
75
50
25
0
Log-rank
p = 0.63
Molecular Glioblastomas
PPM1D Wildtype
PPM1D Alteration
0
20
40
60
80
100
120
Months
19 8 3 2 1 1 1
7 2 2 1 0 0 0

(28)

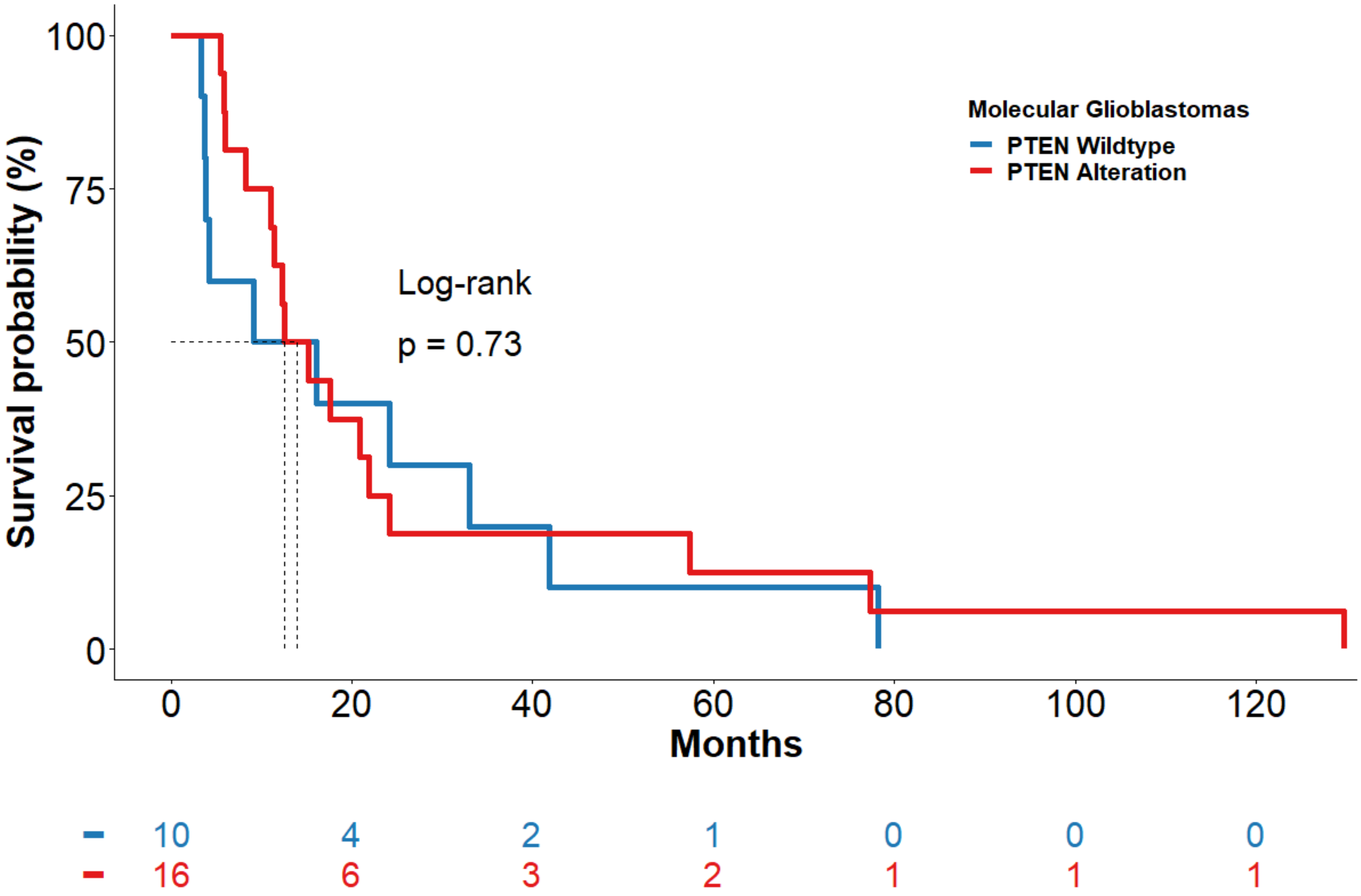

Survival probability (%)
100
75
50
25
0
Log-rank
p = 0.73
Molecular Glioblastomas
PTEN Wildtype
PTEN Alteration
0
20
40
60
80
100
120
Months
10
4
2
1
0
0
0
16
6
3
2
1
1
1

(29)

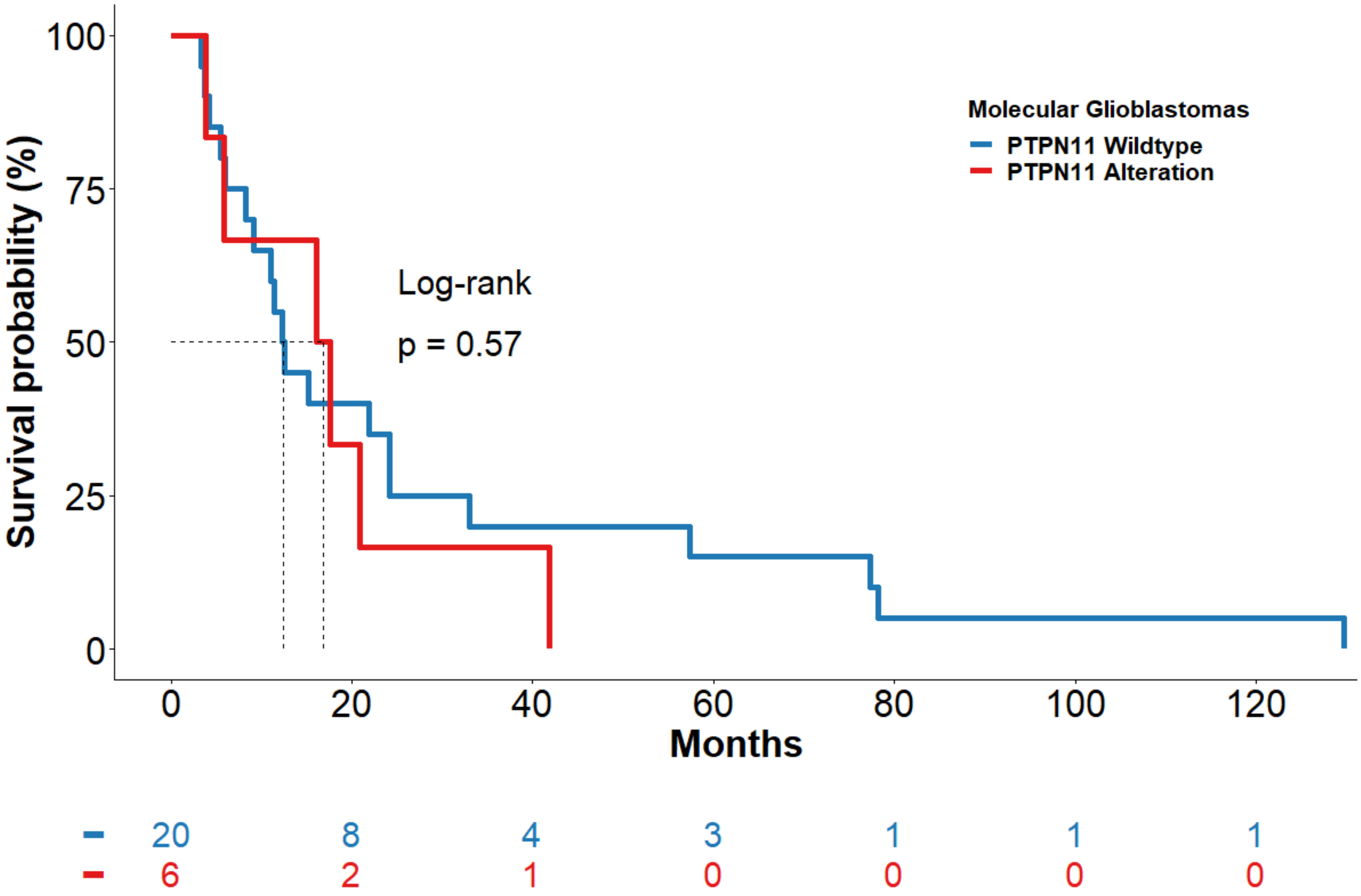

Survival probability (%)
100
75
50
25
0
Log-rank
p = 0.57
Molecular Glioblastomas
PTPN11 Wildtype
PTPN11 Alteration
0
20
40
60
80
100
120
Months
20 8 4 3 1 1 1
6 2 1 0 0 0 0

(30)

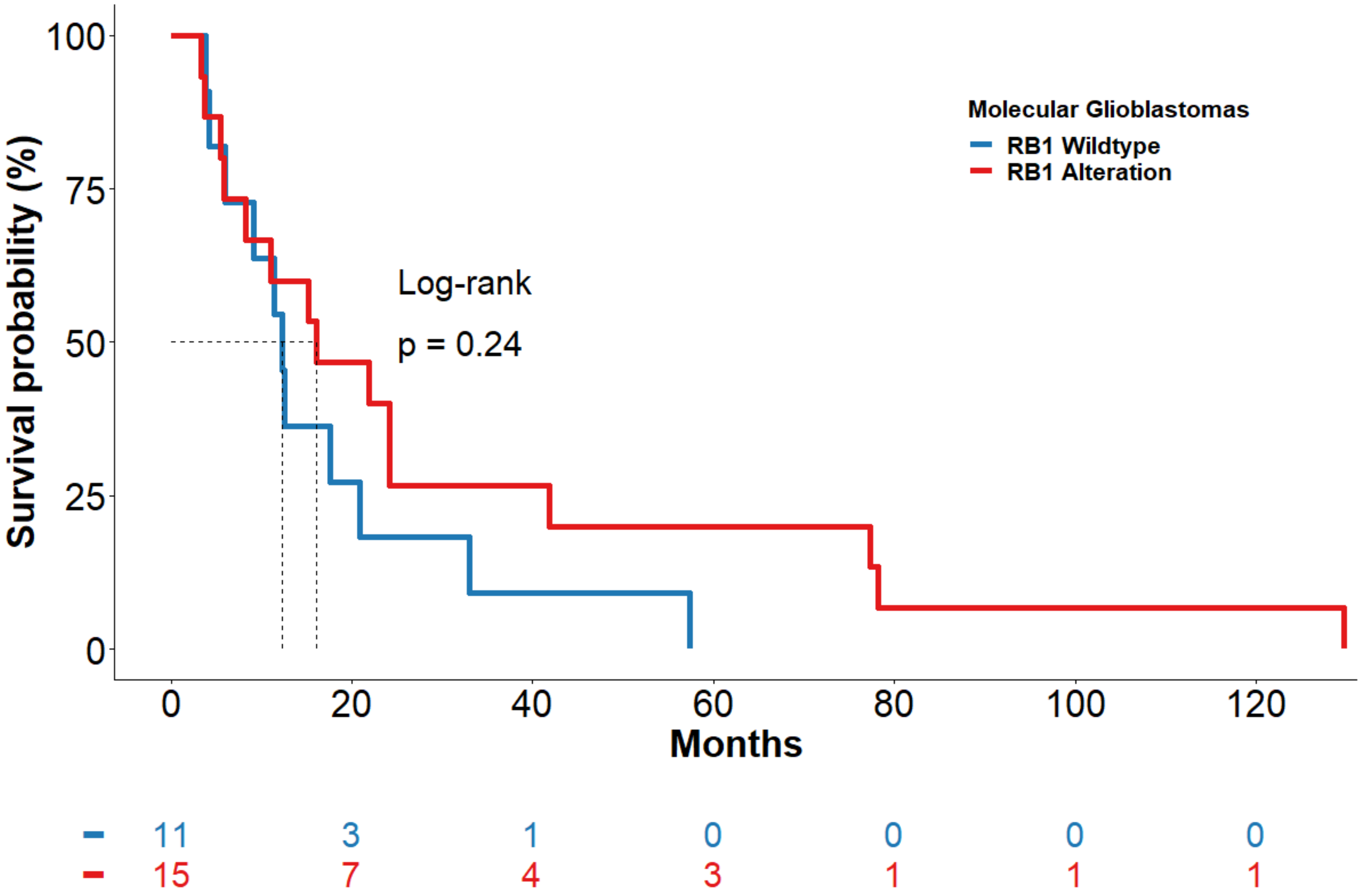

Survival probability (%)
100
75
50
25
0
Log-rank
p = 0.24
Molecular Glioblastomas
RB1 Wildtype
RB1 Alteration
0
20
40
60
80
100
120
Months
11 3 1 0 0 0 0
15 7 4 3 1 1 1

(31)

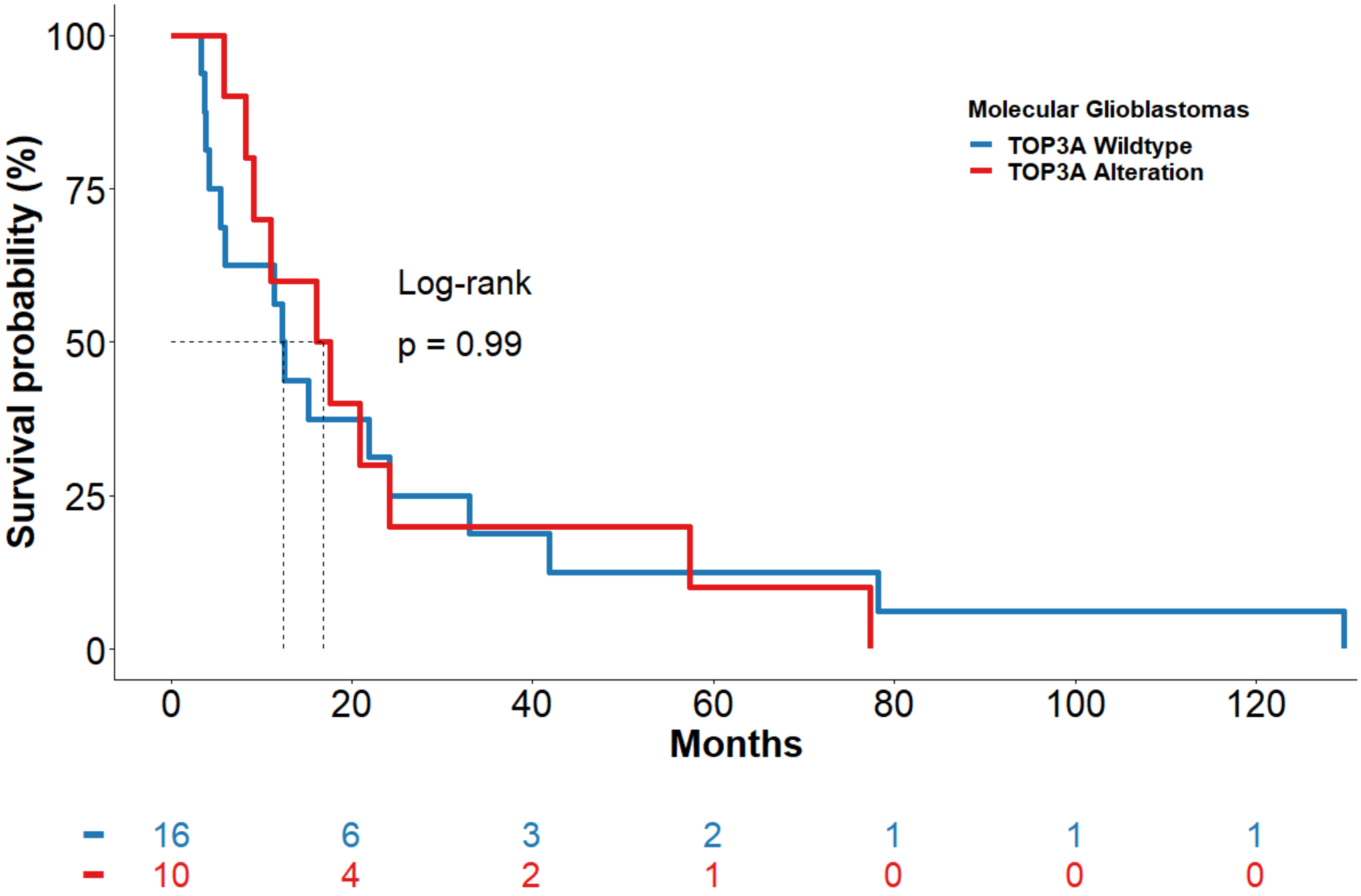

Survival probability (%)
100
75
50
25
0
Log-rank
p = 0.99
Molecular Glioblastomas
TOP3A Wildtype
TOP3A Alteration
0
20
40
60
80
100
120
Months
16
6
3
2
1
1
1
10
4
2
1
0
0
0
