# Supplementary material for: Histological and molecular glioblastoma, IDH-wildtype: a real-world landscape using the 2021 WHO classification of central nervous system tumors
Source: Front Oncol. 2023 Jul 6;13:1200815. doi: 10.3389/fonc.2023.1200815 (PMC10358772; doi:10.3389/fonc.2023.1200815)
Supplement: Supplementary file 4 [file Table_1.doc]

| **Supplementary Table 1. Clinical, radiological, histological, and molecular parameters analyzed in this study.** | | | | | |
| --- | --- | --- | --- | --- | --- |
| **Clinical** | **Radiological** | **Histological** | **Molecular** | | |
| Age at diagnosis | Tumor location | Ki-67 index | *ACVR1* | *IDH2* | *PPM1D* |
| Sex | Frontal lobe | WHO grade | *ATRX* | *KIT* | *PTEN* |
| Body mass index | Temporal lobe | ATRX expression | *BCOR* | *KMT5B* | *PTPN11* |
| Neurologic impairment at diagnosis | Parietal lobe | TP53 expression | *BRAF* | *KRAS* | *P53* |
| Motor dysfunction | Occipital lobe | GFAP expression | *CDK4* | *MAP2K1* | *RB1* |
| Sensory dysfunction | Insular lobe/thalamus/callosum | Olig2 expression | *CDK6* | *MET* | *SMARCA4* |
| Visual field defect | Subtentorial | S-100 expression | *CDKN2A* | *MYB* | *SMARCB1* |
| Aphasia | Tumor contact with functional areas | Syn expression | *CDKN2B* | *MYBL1* | *TERT* |
| Intracranial hypertension at diagnosis | Motor cortex and tract | NeuN expression | *CIC* | *MYC* | *TOP3A* |
| Epilepsy at diagnosis | Sensory cortex and tract |  | *EGFR* | *MYCN* | *TSC1* |
| Memory deterioration at diagnosis | Language area and tract |  | *FBXW7* | *NF1* | *TSC2* |
| Changes in personality at diagnosis | Visual pathway |  | *FGFR1* | *NOTCH1* | *YAP1* |
| Disease duration before admission | Signal on T1-weighted image |  | *FGFR2* | *NRAS* | chr1p |
| Baseline karnofsky performance scale | Signal on T2-weighted image |  | *FGFR3* | *NTRK2* | chr7p |
| Extent of surgical resection | Number of tumors |  | *FGFR4* | *NTRK3* | chr7q |
| Gross total resection | Presence of contrast enhancement |  | *FUBP1* | *PDGFRA* | chr9p |
| Subtotal resection | Intratumoral necrosis |  | *H3F3A* | *PEG3* | chr10p |
| Biopsy | Maximal tumor diameter |  | *HIST1H3B* | *PIK3CA* | chr10q |
| Postoperative treatment | Edema outside of the tumor margin |  | *HIST1H3C* | *PIK3CB* | chr17 |
| Overall survival | Diameter of the intratumoral necrosis |  | *IDH1* | *PIK3R1* | chr19q |
