# Supplementary material for: Histological and molecular glioblastoma, IDH-wildtype: a real-world landscape using the 2021 WHO classification of central nervous system tumors
Source: Front Oncol. 2023 Jul 6;13:1200815. doi: 10.3389/fonc.2023.1200815 (PMC10358772; doi:10.3389/fonc.2023.1200815)
Supplement: Supplementary file 5 [file Table_2.doc]

| **Supplementary Table 2. Molecular alterations and immunohistochemical expressions in histological and molecular glioblastomas.** | | | | | | | | | | |
| --- | --- | --- | --- | --- | --- | --- | --- | --- | --- | --- |
|  | **Histological Glioblastomas (n=42)** | | | |  | **Molecular Glioblastomas (n=36)** | | | | **P Value** |
|  | **Alteration/Positive** | **Mutation** | **Deletion** | **Amplification** |  | **Alteration/Positive** | **Mutation** | **Deletion** | **Amplification** |  |
| **Molecular alterations** | | | | | | | | | | |
| *PTEN* | 35, 83.3% | 12, 28.6% | 23, 54.8% | 0, 0.0% |  | 22, 61.1% | 2, 5.6% | 13, 36.1% | 7, 19.4% | **0.027** |
| *CDK4* | 33, 78.6% | 4, 9.5% | 19, 45.2% | 10, 23.8% |  | 14, 38.9% | 1, 2.8% | 2, 5.6% | 11, 30.6% | **<0.001** |
| *MYB* | 30, 71.4% | 5, 11.9% | 8, 19.0% | 17, 40.5% |  | 17, 47.2% | 5, 13.9% | 7, 19.4% | 5, 13.9% | **0.029** |
| *TOP3A* | 29, 69.0% | 13, 31.0% | 15, 35.7% | 1, 2.4% |  | 14, 38.9% | 8, 22.2% | 2, 5.6% | 4, 11.1% | **0.008** |
| *KIT* | 28, 66.7% | 4, 9.5% | 1, 2.4% | 23, 54.8% |  | 13, 36.1% | 2, 5.6% | 1, 2.8% | 10, 27.8% | **0.007** |
| *KRAS* | 26, 61.9% | 5, 11.9% | 7, 16.7% | 14, 33.3% |  | 14, 38.9% | 5, 13.9% | 0, 0.0% | 9, 25.0% | **0.043** |
| *NTRK3* | 24, 57.1% | 12, 28.6% | 3, 7.1% | 9, 21.4% |  | 11, 30.6% | 4, 11.1% | 1, 2.8% | 6, 16.7% | **0.019** |
| *ACVR1* | 0, 0.0% | 0, 0.0% | 0, 0.0% | 0, 0.0% |  | 0, 0.0% | 0, 0.0% | 0, 0.0% | 0, 0.0% | 1.000 |
| *ATRX* | 2, 4.8% | 2, 4.8% | 0, 0.0% | 0, 0.0% |  | 1, 2.8% | 1, 2.8% | 0, 0.0% | 0, 0.0% | 1.000 |
| *BCOR* | 1, 2.4% | 1, 2.4% | 0, 0.0% | 0, 0.0% |  | 1, 2.8% | 1, 2.8% | 0, 0.0% | 0, 0.0% | 1.000 |
| *BRAF* | 28, 66.7% | 13, 31.0% | 1, 2.4% | 14, 33.3% |  | 29, 80.6% | 13, 36.1% | 1, 2.8% | 15, 41.7% | 0.168 |
| *CDK6* | 36, 85.7% | 0, 0.0% | 0, 0.0% | 36, 85.7% |  | 32, 88.9% | 1, 2.8% | 0, 0.0% | 31, 86.1% | 0.938 |
| *CDKN2A* | 35, 83.3% | 1, 2.4% | 27, 64.3% | 7, 16.7% |  | 26, 72.2% | 3, 8.3% | 21, 58.3% | 2, 5.6% | 0.236 |
| *CDKN2B* | 35, 83.3% | 1, 2.4% | 26, 61.9% | 8, 19.0% |  | 31, 86.1% | 5, 13.9% | 22, 61.1% | 4, 11.1% | 0.735 |
| *CIC* | 5, 11.9% | 5, 11.9% | 0, 0.0% | 0, 0.0% |  | 1, 2.8% | 1, 2.8% | 0, 0.0% | 0, 0.0% | 0.209 |
| *EGFR* | 36, 85.7% | 2, 4.8% | 0, 0.0% | 34, 81.0% |  | 35, 97.2% | 2, 5.6% | 2, 5.6% | 31, 86.1% | 0.116 |
| *FBXW7* | 0, 0.0% | 0, 0.0% | 0, 0.0% | 0, 0.0% |  | 1, 2.8% | 0, 0.0% | 1, 2.8% | 0, 0.0% | 0.462 |
| *FGFR1* | 26, 61.9% | 4, 9.5% | 20, 47.6% | 2, 4.8% |  | 15, 41.7% | 2, 5.6% | 8, 22.2% | 5, 13.9% | 0.074 |
| *FGFR2* | 34, 81.0% | 3, 7.1% | 31, 73.8% | 0, 0.0% |  | 22, 61.1% | 5, 13.9% | 17, 47.2% | 0, 0.0% | 0.052 |
| *FGFR3* | 23, 54.8% | 2, 4.8% | 19, 45.2% | 2, 4.8% |  | 14, 38.9% | 1, 2.8% | 11, 30.6 | 2, 5.6% | 0.162 |
| *FGFR4* | 22, 52.4% | 5, 11.9% | 17, 40.5% | 0, 0.0% |  | 12, 33.3% | 3, 8.3% | 5, 13.9% | 4, 11.1% | 0.091 |
| *FUBP1* | 3, 7.1% | 2, 4.8% | 1, 2.4% | 0, 0.0% |  | 0, 0.0% | 0, 0.0% | 0, 0.0% | 0, 0.0% | 0.245 |
| *H3F3A* | 0, 0.0% | 0, 0.0% | 0, 0.0% | 0, 0.0% |  | 0, 0.0% | 0, 0.0% | 0, 0.0% | 0, 0.0% | 1.000 |
| *HIST1H3B* | 0, 0.0% | 0, 0.0% | 0, 0.0% | 0, 0.0% |  | 0, 0.0% | 0, 0.0% | 0, 0.0% | 0, 0.0% | 1.000 |
| *HIST1H3C* | 0, 0.0% | 0, 0.0% | 0, 0.0% | 0, 0.0% |  | 0, 0.0% | 0, 0.0% | 0, 0.0% | 0, 0.0% | 1.000 |
| *IDH1* | 0, 0.0% | 0, 0.0% | 0, 0.0% | 0, 0.0% |  | 0, 0.0% | 0, 0.0% | 0, 0.0% | 0, 0.0% | 1.000 |
| *IDH2* | 0, 0.0% | 0, 0.0% | 0, 0.0% | 0, 0.0% |  | 0, 0.0% | 0, 0.0% | 0, 0.0% | 0, 0.0% | 1.000 |
| *KMT5B* | 2, 4.8% | 2, 4.8% | 0, 0.0% | 0, 0.0% |  | 7, 19.4% | 7, 19.4% | 0, 0.0% | 0, 0.0% | 0.073 |
| *MAP2K1* | 0, 0.0% | 0, 0.0% | 0, 0.0% | 0, 0.0% |  | 0, 0.0% | 0, 0.0% | 0, 0.0% | 0, 0.0% | 1.000 |
| *MET* | 27, 64.3% | 1, 2.4% | 0, 0.0% | 26, 61.9% |  | 23, 63.9% | 8, 22.2% | 0, 0.0% | 15, 41.7% | 0.971 |
| *MYBL1* | 21, 50.0% | 1, 2.4% | 0, 0.0% | 20, 47.6% |  | 14, 38.9% | 3, 8.3% | 1, 2.8% | 10, 27.8% | 0.325 |
| *MYC* | 15, 35.7% | 6, 14.3% | 6, 14.3% | 3, 7.1% |  | 14, 38.9% | 2, 5.6% | 6, 16.7% | 6, 16.7% | 0.772 |
| *MYCN* | 10, 23.8% | 4, 9.5% | 2, 4.8% | 4, 9.5% |  | 6, 16.7% | 0, 0.0% | 3, 8.3% | 3, 8.3% | 0.436 |
| *NF1* | 5, 11.9% | 5, 11.9% | 0, 0.0% | 0, 0.0% |  | 5, 13.9% | 5, 13.9% | 0, 0.0% | 0, 0.0% | 1.000 |
| *NOTCH1* | 19, 45.2% | 7, 16.7% | 5, 11.9% | 7, 16.7% |  | 13, 36.1% | 1, 2.8% | 2, 5.6% | 10, 27.8% | 0.414 |
| *NRAS* | 0, 0.0% | 0, 0.0% | 0, 0.0% | 0, 0.0% |  | 0, 0.0% | 0, 0.0% | 0, 0.0% | 0, 0.0% | 1.000 |
| *NTRK2* | 26, 61.9% | 4, 9.5% | 4, 9.5% | 18, 42.9% |  | 18, 50.0% | 6, 16.7% | 3, 8.3% | 9, 25.0% | 0.291 |
| *PDGFRA* | 26, 61.9% | 10, 23.8% | 2, 4.8% | 14, 33.3% |  | 16, 44.4% | 7, 19.4% | 0, 0.0% | 9, 25.0% | 0.123 |
| *PEG3* | 25, 59.5% | 4, 9.5% | 0, 0.0% | 21, 50.0% |  | 17, 47.2% | 3, 8.3% | 3, 8.3% | 11, 30.6% | 0.277 |
| *PIK3CA* | 28, 66.7% | 1, 2.4% | 1, 2.4% | 26, 61.9% |  | 20, 55.6% | 3, 8.3% | 0, 0.0% | 17, 47.2% | 0.315 |
| *PIK3CB* | 1, 2.4% | 1, 2.4% | 0, 0.0% | 0, 0.0% |  | 1, 2.8% | 1, 2.8% | 0, 0.0% | 0, 0.0% | 1.000 |
| *PIK3R1* | 2, 4.8% | 2, 4.8% | 0, 0.0% | 0, 0.0% |  | 3, 8.3% | 1, 2.8% | 0, 0.0% | 2, 5.6% | 0.657 |
| *PPM1D* | 14, 33.3% | 6, 14.3% | 1, 2.4% | 7, 16.7% |  | 10, 27.8% | 2, 5.6% | 1, 2.8% | 7, 19.4% | 0.596 |
| *PTPN11* | 19, 45.2% | 3, 7.1% | 15, 35.7% | 1, 2.4% |  | 11, 30.6% | 4, 11.1% | 2, 5.6% | 5, 13.9% | 0.184 |
| *RB1* | 22, 52.4% | 5, 11.9% | 8, 19.0% | 9, 21.4% |  | 19, 52.8% | 4, 11.1% | 5, 13.9% | 10, 27.8% | 0.972 |
| *SMARCA4* | 0, 0.0% | 0, 0.0% | 0, 0.0% | 0, 0.0% |  | 2, 5.6% | 1, 2.8% | 1, 2.8% | 0, 0.0% | 0.210 |
| *SMARCB1* | 0, 0.0% | 0, 0.0% | 0, 0.0% | 0, 0.0% |  | 0, 0.0% | 0, 0.0% | 0, 0.0% | 0, 0.0% | 1.000 |
| *TERT* | 35, 83.3% | 35, 83.3% | 0, 0.0% | 0, 0.0% |  | 26, 72.2% | 26, 72.2% | 0, 0.0% | 0, 0.0% | 0.236 |
| *TP53* | 9, 21.4% | 9, 21.4% | 0, 0.0% | 0, 0.0% |  | 3, 8.3% | 3, 8.3% | 0, 0.0% | 0, 0.0% | 0.110 |
| *TSC1* | 0, 0.0% | 0, 0.0% | 0, 0.0% | 0, 0.0% |  | 1, 2.8% | 1, 2.8% | 0, 0.0% | 0, 0.0% | 0.462 |
| *TSC2* | 2, 4.8% | 2, 4.8% | 0, 0.0% | 0, 0.0% |  | 1, 2.8% | 1, 2.8% | 0, 0.0% | 0, 0.0% | 1.000 |
| *YAP1* | 0, 0.0% | 0, 0.0% | 0, 0.0% | 0, 0.0% |  | 0, 0.0% | 0, 0.0% | 0, 0.0% | 0, 0.0% | 1.000 |
| chr1p | 41, 97.6% | 30, 71.4% | 3, 7.1% | 8, 19.0% |  | 36, 100.0% | 30, 83.3% | 0, 0.0% | 6, 16,7% | 1.000 |
| chr7p | 41, 97.6% | 5, 11.9% | 0, 0.0% | 36, 85.7% |  | 36, 100.0% | 4, 11.1% | 1, 2.8% | 31, 86.1% | 1.000 |
| chr7q | 40, 95.2% | 12, 28.6% | 0, 0.0% | 28, 66.7% |  | 36, 100.0% | 9, 25.0% | 0, 0.0% | 27, 75.0% | 0.497 |
| chr9p | 41, 97.6% | 9, 21.4% | 24, 57.1% | 8, 19.0% |  | 36, 100.0% | 15, 41.7% | 20, 55.6% | 1, 2.8% | 1.000 |
| chr10p | 41, 97.6% | 9, 21.4% | 31, 73.8% | 1, 2.4% |  | 35, 97.2% | 11, 30.6% | 24, 66.7% | 0, 0.0% | 1.000 |
| chr10q | 41, 97.6% | 4, 9.5% | 36, 85.7% | 1, 2.4% |  | 36, 100.0% | 15, 41.7% | 21, 58.3% | 0, 0.0% | 1.000 |
| chr17 | 39, 92.9% | 30, 71.4% | 5, 11.9% | 4, 9.5% |  | 34, 94.4% | 29, 80.9% | 0, 0.0% | 5, 13.9% | 1.000 |
| chr19q | 37, 88.1% | 21, 50.0% | 4, 9.5% | 12, 28.6% |  | 30, 83.3% | 18, 50.0% | 1, 2.8% | 11, 30.6% | 0.547 |
